# Noggin contributes to brain metastatic colonization of lung cancer cells

**DOI:** 10.1186/s12935-023-03155-7

**Published:** 2023-11-28

**Authors:** Jung Eun Lee, Jihye Park, Eun Ju Kim, Yoon Ho Ko, Soon Auck Hong, Seung Ho Yang, Young-Ho Ahn

**Affiliations:** 1 Department of Neurosurgery, St. Vincent’s Hospital, College of Medicine, The Catholic University of Korea, Seoul, Republic of Korea; 2 Department of Molecular Medicine and Inflammation-Cancer Microenvironment Research Center, College of Medicine, Ewha Womans University, 25 Magokdong-ro 2-gil, Gangseo-gu, Seoul, 07804 Republic of Korea; 3 Department of Internal Medicine, Division of Oncology, College of Medicine, The Catholic University of Korea, Seoul, Republic of Korea; 4 Department of Pathology, College of Medicine, Chung-Ang University, Seoul, Republic of Korea; 5 Department of Neurosurgery, St. Vincent’s Hospital, College of Medicine, The Catholic University of Korea, 93 Jungbu-daero, Paldal-gu, Suwon, 16247 Republic of Korea

**Keywords:** Brain metastasis, Lung cancer, Mesenchymal–epithelial transition, Noggin

## Abstract

**Background:**

Brain metastasis is a common complication among patients with lung cancer, yet the underlying mechanisms remain unclear. In this study, we aimed to investigate the pathogenesis of brain metastasis in lung cancer.

**Methods:**

We established highly colonizing metastatic lung cancer cells, A549-M2, through multiple implantations of A549 human lung cancer cells in the carotid artery of athymic nude mice.

**Results:**

Compared to parental cells (M0), M2 cells demonstrated slower growth in culture plates and soft agar, as well as lower motility and higher adhesion, key characteristics of mesenchymal–epithelial transition (MET). Further analysis revealed that M2 cells exhibited decreased expression of epithelial–mesenchymal transition markers, including ZEB1 and Vimentin. M2 cells also demonstrated reduced invasiveness in co-culture systems. RNA sequencing and gene set enrichment analysis confirmed that M2 cells underwent MET. Intriguingly, depletion of Noggin, a BMP antagonist, was observed in M2 cells, and replenishment of Noggin restored suppressed migration and invasion of M2 cells. In addition, Noggin knockdown in control M0 cells promoted cell attachment and suppressed cell migration, suggesting that Noggin reduction during brain colonization causes inhibition of migration and invasion of metastatic lung cancer cells.

**Conclusions:**

Our results suggest that lung cancer cells undergo MET and lose their motility and invasiveness during brain metastatic colonization, which is dependent on Noggin.

**Supplementary Information:**

The online version contains supplementary material available at 10.1186/s12935-023-03155-7.

## Introduction

Lung cancer is the leading cause of cancer-related death in the world, with over 1.8 million new cases diagnosed annually. Five-year survival rate of patients with lung cancer ranges from 4 to 17% depending on the stage and regional difference [1, 2]. Despite advances in diagnosis and treatment, most lung cancer patients are still diagnosed at advanced stages with poor prognosis, with brain metastasis being a major contributor to this outcome [3, 4]. Metastasis, which accounts for up to 90% of cancer-related deaths [5], occurs when epithelial cancer cells within a primary tumor undergo an epithelial–mesenchymal transition (EMT), allowing them to acquire mobility and invasiveness, spread to distant sites via the circulatory system, and establish metastatic tumors after completing mesenchymal–epithelial transition (MET) [6]. Interestingly, each carcinoma has a preferred organotropism, with lung cancer commonly leading to brain metastasis in 40% of patients [7]. However, the underlying pathological mechanisms of brain metastasis in lung cancer remain elusive, emphasizing the necessity to identify new biomarkers and potential therapeutics to specifically target and alleviate brain metastasis.

Significant efforts have been conducted to determine genomic variability between primary lung tumors and brain metastases, with a focus on identifying the most frequent genetic alterations, including aberrations in *EGFR*, *ALK*, and *KRAS* [8, 9]. Recently, two independent studies have shown that presence of *RET* fusion in non-small cell lung cancer (NSCLC) patients is associated with higher occurrence of brain metastasis [10, 11]. Caveolin-1 expression levels are significantly increased in patients with lung cancer brain metastasis compared with those in patients with matched primary tumors [12]. Although several potential lung cancer brain metastasis biomarkers have been identified in recent years, most of them have not been clinically approved yet.

Direct injection of tumor cells into circulation via the carotid artery is a commonly utilized experimental approach to assess the development of brain metastasis. However, it is important to note that this method primarily evaluates brain colonization of tumor cells rather than full metastasis as it does not account for the potential of tumor cells to undergo EMT and intravasate into circulation. Furthermore, the quantity of cells introduced into circulation is several times greater than that of cells that typically escape from a primary tumor. Nevertheless, this technique enables the selection of highly metastatic cell populations with the capacity to cross the blood–brain barrier (BBB) and colonize the brain, resulting in the occurrence of brain metastasis [13, 14].

Noggin is a secreted protein that belongs to the BMP antagonist family, and it has been found to play a complex and context-dependent role in cancer development. Noggin has been shown to have anti-cancer effects by inhibiting the activity of BMPs, which are a group of proteins that belong to the TGF-β superfamily and are involved in various cellular processes such as cell proliferation, differentiation, and migration [15]. By blocking BMP activity, Noggin has been shown to inhibit the growth of osteolytic prostate cancer lesions [16]. In lung cancer cells, Noggin abrogated angiogenic activity enhanced by BMP-2 [17]. However, Noggin can also have pro-cancer effects in certain contexts. For example, in patients with gastric cancer, high Noggin expression is associated with a poor prognosis, and Noggin promotes the proliferation of gastric cancer cells by upregulating EGFR signaling [18]. Pro- and anti-cancer effects of Noggin are complex and dependent on the specific cancer type and context; therefore, further research is needed to fully understand the role of Noggin in cancer development and to explore its potential as a therapeutic target for cancer treatment.

The objective of this study was to establish lung cancer cells with optimized properties for brain metastasis through repeated implantation via the carotid artery and to uncover genetic changes associated with colonization of brain metastasis.

## Materials and methods

### Mouse experiments

Before initiation, all proposed mouse studies were submitted to and approved by the Institutional Animal Care and Use Committee (IACUC) of The Catholic University of Korea (CUMC-2016-0273-01). A549 was obtained from American Type Culture Collection (ATCC). The cell line has been authenticated using STR profiling with Powerplex 18D system (Promega; Madison, WI, USA) by COSMOgenetech (Daejeon, Korea) within the last 3 years. A total of 42 male homozygous asexual nude mice (BALB/c nude), preferably weighing 19–21 g and aged 8–10 weeks, were purchased from OrientBio (Seongnam, Korea). The mice used in the study were housed in standardized animal rooms with a room temperature maintained at 22 °C ± 2 °C and a light schedule of 7 am–7 pm. They were provided ad libitum access to both food and water throughout the duration of the experiment. Mice were cared for and euthanized according to the standards set forth by the IACUC. Briefly, anesthesia was induced with 4% isoflurane, followed by maintenance with 2.5% isoflurane (oxygen flow: ~ 400 ml/min) using a precision vaporizer, while monitoring vital signs. After respiratory anesthesia with isoflurane, mice were laid in a supine position. A 1.0 cm-long skin incision was made in the middle of the neck to perform blunt dissection of cervical tissue under a stereomicroscope, revealing the pulsating right common carotid artery. The proximal portion of the common carotid artery was ligated using a 7–0 suture. The distal part was wrapped without ligation. The right external carotid artery was also ligated. A 31-gauge needle was used to advance the tip of the cannula into the internal carotid artery for common carotid artery puncture. Following injection of 10 μL of A549 cell suspension (100 cells/μL), the syringe was removed, and the distal suture was used to ligate the right common carotid artery. The mouse's postoperative activity was monitored after the skin was sutured. Prior to the surgery, mice were monitored on a weekly basis, and the frequency of monitoring was subsequently increased to every 2 days. Mice were euthanized approximately 8 weeks after cell transplantation. The criterion for determining the endpoint was a 20% loss of body weight, as visual assessment of tumor size was not feasible; however, mice were euthanized if they exhibited an inability to carry out normal physiological activities such as eating, defecating, and urinating. The euthanasia procedure for mice was conducted in a dedicated acrylic chamber using CO_2_ gas. The filling rate was maintained at 30–70% per min to ensure a gradual and controlled process. Following an approximate exposure time of 5 min to 100% CO_2_ within the chamber, the gas flow was continued for an additional minimum of 1 min. This duration allowed for the confirmation of definitive veterinary death, specifically by observing evident signs such as respiratory arrest. The employed protocol aimed to ensure a humane and effective euthanasia procedure. The animal studies detailed here took place between the years 2020 and 2022. IACUC and Department of Laboratory Animal in The Catholic University of Korea accredited the Korea Excellence Animal Laboratory Facility from Korea Food and Drug Administration in 2017 and acquired AAALAC International full accreditation in 2018.

### Establishment of brain-colonized lung cancer cells

Initially, a group of mice underwent a single injection of parental A549 cells (A549-M0, 1 × 10^3^ cells) through the carotid artery, as described earlier, to establish A549-M1 cells. Subsequently, a distinct group of mice received a single injection of A549-M1 cells using the same injection technique to establish A549-M2 cells. After confirming brain metastasis with bioluminescence imaging, fresh brain tissues were aseptically cut into fine pieces (about 1 mm^3^) and were then digested with 2.5% trypsin (20 mL per 1 g of tissue, Welgene; Gyeongsan, Korea) with slow but constant mixing. To obtain a single cell suspension, the samples were gradually filtered through a cell strainer (SPL Life Sciences; Pocheon, Korea) with a pore size of 40 µm. After dilution with DMEM (Welgene) supplemented with 10% fetal bovine serum (Corning; Corning, NY, USA), isolated cancer cells (A549-M1) were placed in T25 culture flasks (SPL Life Sciences) and incubated at 37 °C and 5% CO_2_. A549-M2 cells that colonized in the brain were isolated though the repeated injection of A549-M1 cells into the mice via the carotid artery and were established as mentioned above.

### Bioluminescence imaging

The response to tumor growth was monitored using bioluminescence imaging with an IVIS spectral imaging system (PerkinElmer, Waltham, MA). Bioluminescence IVIS acquisitions were carried out throughout the duration of the experiment. To facilitate imaging, mice were treated with D-luciferin (150 mg/kg) via intraperitoneal injection. Mice were anesthetized using gas anesthesia with 2.5% isoflurane 15 min after luciferin administration. Imaging was conducted on black paper within the IVIS imaging system box.

### Cell culture

A549-M0 and A549-M2 human lung cancer cells were cultured in RPMI 1640 (Welgene). Immortalized human astrocytes (abm; Richmond, BC, Canada) and 293 T were cultured in DMEM (Welgene) supplemented with 10% fetal bovine serum at 37 °C and 5% CO_2_. For actin staining, cells were cultured on coverslips coated with collagen (0.1 mg/mL), fixed with 4% formaldehyde, and stained with Alexa Fluor 594-phalloidin (Invitrogen; Carlsbad, CA, USA) and DAPI (5 μg/mL) according to the manufacturer's protocol. Cell numbers were counted using a LUNA automated cell counter (Logos Biosystems; Anyang, Korea). For clonogenic assay, cells (200/well) were seeded into 6-well plates and stained with 0.1% crystal violet after 6 days. For soft agar colony assay, cells (5 × 10^4^/well) were suspended in 0.3% agarose and seeded into 6-well plates layered with 0.8% agarose. After 3 weeks, colonies were stained with nitro blue tetrazolium (0.5 mg/mL). For wound healing assay, cells (6 × 10^5^/well) were seeded into 6-well plates. Once reaching confluence, cell monolayer was scratched in a straight line using a pipette tip and incubated with mitomycin C (1 μg/mL) for 48 h. Wound area was measured using ImageJ (NIH; Bethesda, MD, USA).

### Gene overexpression and knockdown

For Noggin overexpression, human *NOG* cDNA (NM_005450) in pDNR-LIB vector (Clontech, Mountain View, CA, USA) was obtained from Korea Human Gene Bank (#hMU013799; Medical Genomics Research Center, KRIBB, Korea). *NOG* cDNA was cloned into pLVX-Neo vector and then transduced into A549-M2 cells by lentiviral infection. After the viral infection, A549-M2 cells transduced with *NOG* were stably established through a selection process using G418 (500 µg/mL; InvivoGen, San Diego, CA) for over 2 weeks. Human *NOG* siRNAs and non-targeting siRNA (AccuTarget Negative Control siRNA, #SN-1002) were purchased from Bioneer (Daejeon, Korea) and transiently transfected into lung cancer cells using a TransIT-X2 Dynamic Delivery System (Mirus Bio; Madison, WI, USA). The sequences and catalogue numbers of *NOG* siRNAs are as follows:

- #1 (9241-1): 5′-AGAGAGACUUAUUCUGGUU(TT)-3′, 5′-AACCAGAAUAAGUCUCUCU(TT)-3′
- #2 (9241-2): 5′-CAUUCUUCGGAAAGUGUUU(TT)-3′, 5′-AAACACUUUCCGAAGAAUG(TT)-3′
- #3 (9241-3): 5′-GAAGCUGCGGAGGAAGUUA(TT)-3′, 5′-UAACUUCCUCCGCAGCUUC(TT)-3′

Transfection efficiency was evaluated by using AccuTarget Fluorescein-labeled Negative Control siRNA (#SN-1022, Bioneer), which typically resulted in a transfection efficiency of 50–70%. The effectiveness of gene knockdown was also verified through qRT-PCR. For fluorescent labeling, A549-M0 and A549-M2 cells were transduced with a pLVX-Puro/EGFP vector (green fluorescence) and astrocytes were transduced with a pCDH-CMV-mCherry-EF1 Hygro vector (red fluorescence; a gift from Oskar Laur, Addgene plasmid #129440). After the viral infection, A549 cells overexpressing EGFP and astrocytes overexpressing mCherry were stably established through a selection process using puromycin (2 µg/mL; InvivoGen) or hygromycin (250 µg/mL; InvivoGen) for over 2 weeks, respectively. All experiments were performed with mycoplasma-free cells.

### Transwell migration assay

Cells (1 × 10^5^/well) were seeded onto transwell inserts (Falcon Cell Culture Inserts, 8 μm pore; Corning). Cells were incubated for 24 h and allowed to migrate toward 10% fetal bovine serum in bottom wells. Migrated cells were fixed with 90% ethanol, stained with 0.1% crystal violet, photographed, and counted. For co-culture with A549-M0/M2 and astrocytes, both EGFP-labelled A549-M0/M2 (5 × 10^4^/well) and mCherry-labelled astrocytes (5 × 10^4^/well) were cultured in transwell inserts for 24 h. Migrated cells were photographed under a fluorescence microscope and counted.

### Cell attachment assay

This assay is based on Humphries' method [19] with some modifications. Cells (3 × 10^5^/well) were seeded into collagen-coated 24-well plates and allowed to attach to the bottom for 30 min. After incubation, plates were washed twice with PBS to remove non-attached cells. The attached cells were then fixed with 90% ethanol and stained with 0.1% crystal violet solution. The dye was dissolved with 10% acetic acid and percentage of attached cells was measured spectrophotometrically at 595 nm.

### Spheroid overlay assay

To create spheroids, 25 μL droplets containing EGFP-labelled A549 cells (500 cells/droplet) in complete medium with 20% Methocel (Sigma-Aldrich; St. Louis, MO, USA) and 1% Matrigel (BD Biosciences; Franklin Lakes, NJ, USA) were hung on lids of 100-mm petri dishes and incubated at 37 °C for 2 days. Spheroids were then overlaid on top of a feeder cell layer of mCherry-labelled astrocytes. After 12 h, invasion of A549 spheroids was visualized with a fluorescence microscope.

### Western blotting

Cell lysates were prepared with a lysis buffer (50 mM Tris-Cl pH 7.4, 150 mM NaCl, 1 mM EDTA, 1% Triton X-100) containing protease inhibitors (Sigma-Aldrich). Afterwards, proteins (50–100 μg) were separated by SDS-PAGE, transferred to PVDF membranes, and incubated with primary antibodies and HRP-conjugated secondary antibodies (Bio-Rad; Hercules, CA, USA). Visualization of protein bands was performed using a Miracle-Star Western Blot Detection System (iNtRON Biotechnology, Seongnam, Korea). Antibodies against ZEB1 (1:1000 dilution, #NBP1-05987, Novus Biologicals; Centennial, CO, USA), Vimentin (1:1000 dilution, #sc-5565, Santa Cruz Biotechnology; Dallas, TX, USA), E-cadherin (1:1000 dilution, #sc-8426, Santa Cruz Biotechnology), N-cadherin (1:250 dilution, #sc-7939, Santa Cruz Biotechnology), Twist1 (1:1000 dilution, #90445, Cell Signaling Technology, Danvers, MA, USA), Noggin (1;1000 dilution, #sc-293439, Santa Cruz Biotechnology), and β-actin (1:5000 dilution, #BS6007M, Bioworld Technology; St. Louis Park, MN, USA) were purchased and used.

### RNA sequencing

Total RNA concentration was determined with a Quant-IT RiboGreen (Invitrogen). To assess the integrity of total RNA, samples were run on a TapeStation RNA ScreenTape (Agilent Technology; Santa Clara, CA, USA). Only high-quality RNA preparations with RIN greater than 7.0 were used for RNA library construction. A library was independently prepared using 1 μg of total RNA for each sample and Illumina TruSeq Stranded mRNA Sample Prep Kit (Illumina; San Diego, CA, USA). The first step in the workflow involved purifying poly‐A containing mRNA molecules using poly‐T‐attached magnetic beads. Following purification, the mRNA was fragmented into small pieces using divalent cations under elevated temperature. Cleaved RNA fragments were copied into first strand cDNA using SuperScript II reverse transcriptase (Invitrogen) and random primers, followed by second strand cDNA synthesis using DNA Polymerase I, RNase H, and dUTP. These cDNA fragments then underwent an end repair process, addition of a single ‘A’ base, and ligation of adapters. Products were then purified and enriched by PCR to create the final cDNA library. Libraries were quantified using KAPA Library Quantification kits for Illumina Sequencing platforms (Kapa Biosystems; Wilmington, MA, USA) and qualified using the TapeStation D1000 ScreenTape (Agilent). Indexed libraries were then submitted to Macrogen (Seoul, Korea) for paired-end (2 × 100 bp) sequencing using Illumina NovaSeq (Illumina). Differentially expressed genes between M0 and M2 cells (fold-change > 3, *P*-value < 0.05) are listed in Additional file 1: Table S1.

### Quantitative real-time reverse transcription PCR (qRT-PCR)

Total RNA was isolated from A549-M0 and A549-M2 cells with AccuPrep Universal RNA Extraction Kit (Bioneer). Reverse transcription was performed with ELPIS RT Prime Kit (Elpis-Biotech, Daejeon, Korea). qRT-PCR assays were performed using a BioFACT A-Star Real-time PCR Kit including SFCgreen I (BioFACT, Daejeon, Korea). Real-time PCR was conducted on the AriaMx Real-time PCR system (Agilent Technologies) using a two-step PCR condition consisting of 40 cycles of 95 °C for 20 s followed by 60 °C for 40 s. mRNA levels were normalized to ribosomal protein L32 (*RPL32*) mRNA [20]. Quantification data were calculated using the 2^−ΔΔCt^ method. PCR primers used in this study are listed in Additional file 2: Table S2.

### Statistics

Data were analyzed with unpaired two-tailed Student’s *t*-tests and two-way ANOVA with Bonferroni post hoc tests using GraphPad Prism (La Jolla, CA, USA) unless otherwise noted. *P*-values < 0.05 were considered statistically significant.

## Result

### Repeated implantation potentiates brain colonization ability of lung cancer cells

**Fig. 1:**
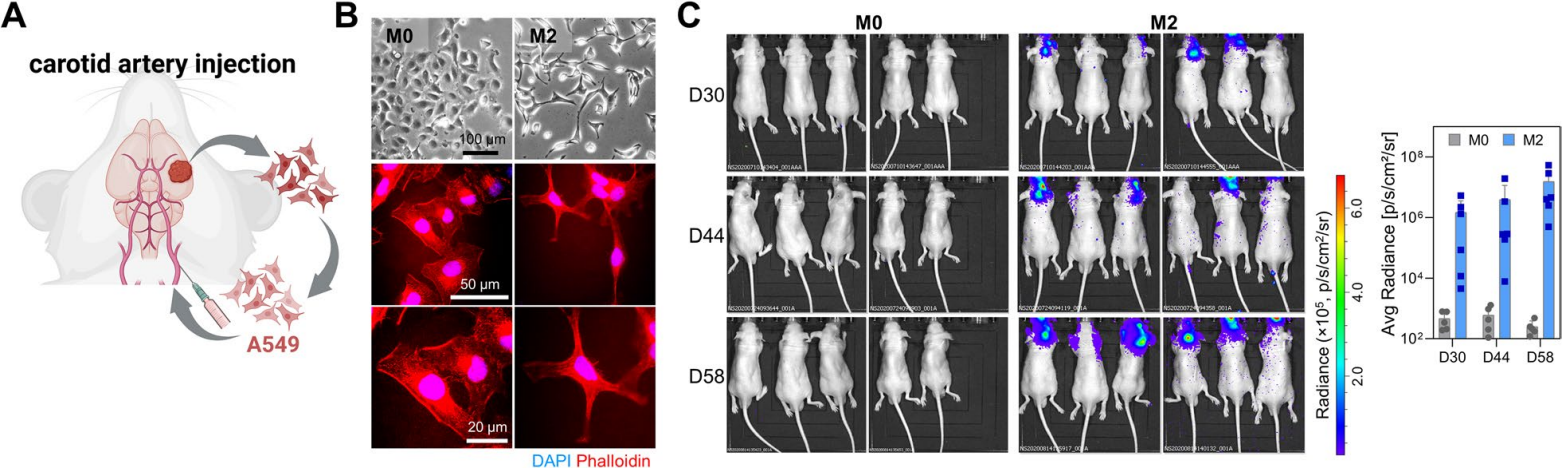
Repeated implantation of lung cancer cells potentiates their brain colonization ability. **A** Establishment of brain-colonized lung cancer cells. A549 human lung cancer cells (M0 cells) were injected into the brain of a mouse via the carotid artery. After 60 days, brain-colonized tumor was isolated and dissociated into single cells (M1 cells). Cultured and expanded M1 cells were re-injected into the brain. The second-round brain-colonized tumor was isolated and dissociated into single cells, which were expanded to establish M2 cells. **B** Cell morphology of M0 and M2 cells. Representative phase-contrast and fluorescence microscopic images of M0 and M2 cells. Cells were stained with phalloidin conjugated to Alexa 594 (red) and DAPI (blue). **C** In vivo bioluminescence images of athymic nude mice injected with M0 and M2 cells. M0 and M2 cells were injected into brains of BALB/c nude mice via carotid arteries. Brain-colonized tumors were visualized by bioluminescence imaging at 30, 44, and 58 days after injection. The graph denotes average radiance measured by using bioluminescence imaging

To experimentally recapitulate metastatic brain colonization of lung cancer cells, A549 human lung adenocarcinoma cells were injected into the brain cerebrum via the carotid artery of an athymic nude mouse. After 8 weeks, tumor formation in the brain was confirmed using in vivo bioluminescent imaging (data not shown). Brain-colonized tumors were isolated, dissociated into single-cell suspension, and then re-implanted into the brain of another mouse using the same method (Fig. 1a). After two cycles of brain implantation and isolation, A549-M2 cells were established. Parental A549 cells were named A549-M0. A549-M0, M1, and M2 cells were slightly different in cellular morphology. M1 and M2 cells were more pointed and elongated than M0 cells (Fig. 1b; Additional file 3: Fig. S1A). When these two cancer cells were injected again into the brain via the carotid artery, M2 cells colonized in the brain much better than M0 cells (Fig. 1c). These results suggest that through repeated implantation into the brain, lung cancer cells with high brain colonization potential could be selectively isolated and established.

### Brain-colonized lung cancer cells have lower growth and migration activities than parental cells

**Fig. 2:**
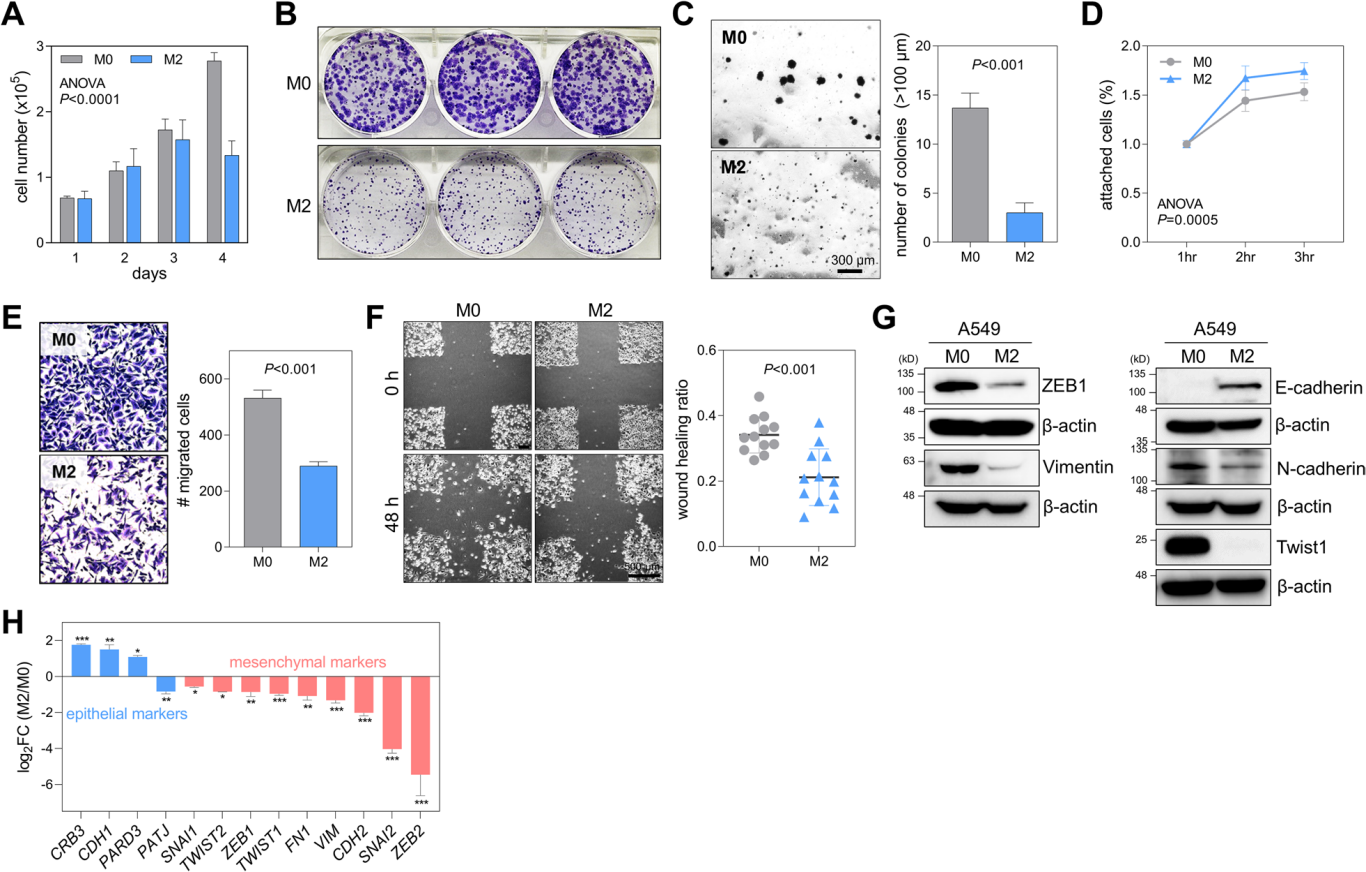
Brain colonization diminishes the growth and migration of metastatic lung cancer cells. **A** Cell growth of A549-M0 and M2 cells. Cells were counted using an automated cell counter (n = 3). Mean + SD. *P*, two-way ANOVA with Bonferroni post hoc tests. The adjusted *P*-value for the 4-day time point is < 0.0001. **B** Colonogenic assays of M0 and M2 cells. Colonies were stained with crystal violet at 6 days after seeding. **C** Soft agar colony assays of M0 and M2 cells. Colonies over 100 μm in diameter were counted at 3 weeks after seeding. Mean + SD (n = 3). *P*, unpaired two-tailed Student’s *t*-test. **D** Cell attachment assays of M0 and M2 cells. Cells (3 × 10^5^/well) were seeded into collagen-coated 24-well plates. Non-adherent cells were removed by washing with PBS after 30 min. Attached cells were measured by crystal violet staining. Mean ± SD (n = 3). *P*, two-way ANOVA with Bonferroni post hoc tests. The adjusted *P*-values for the 2-h and 3-h time points are 0.0038 and 0.0079, respectively. **E** Transwell migration assays of M0 and M2 cells. Cells (1 × 10^5^/insert) were cultured in upper wells for 24 h. Migrated cells were counted after staining with crystal violet. Mean ± SD (n = 3). *P*, unpaired two-tailed Student’s *t*-test. **F** Wound healing assays of M0 and M2 cells. Confluent cell cultures were subjected to scratch wounds and then incubated for 48 h in the presence of mitomycin C (1 μg/mL) to attenuate the proliferation-caused effect. Wound healing ratio (1 − [wound area ratio of 48–0 h]) was measured using ImageJ (http://imagej.nih.gov/ij). Mean ± SD (n = 12). *P*, unpaired two-tailed Student’s *t*-test. **G** Western blot of ZEB1, Vimentin, E-cadherin, N-cadherin, and Twist1 in M0 and M2 cells. β-actin was used as a loading control. **H** qRT-PCR of epithelial and mesenchymal markers in M0 and M2 cells. Expression levels were normalized to *RPL32* mRNA levels. *P < 0.05, **P < 0.01, ***P < 0.001; unpaired two-tailed Student’s *t*-test

First, we compared growth rates of A549-M0 and M2 cells. Cell counting assay revealed that M2 cells grew slower than M0 cells (Fig. 2a). M1 and M2 cells formed much fewer and smaller colonies than M0 cells in the clonogenic assay (Fig. 2b; Additional file 3: Fig. S1B). In soft agar colony assay, M2 cells could not form as many colonies as M0 cells (Fig. 2c). These data meant that proliferative capacity of metastatic lung cancer cells might be reduced after brain colonization. The poor colony formation of M2 cells on soft agar indicates that these cells are highly dependent on anchorage or adherence to the bottom. Supporting this, M2 cells attached to the collagen-coated plate better than M0 cells in the cell attachment assay (Fig. 2d). M1 and M2 cells were less motile than M0 cells in the transwell migration assay, conducted 24 h after cell seeding (Fig. 2e; Additional file 3: Fig. S1C). The same result was obtained in the wound-healing assay, conducted 48 h after cell seeding (Fig. 2f). High adherence and low motility are main indicators of mesenchymal-to-epithelial transition (MET) [6]. Therefore, we checked expression levels of mesenchymal and epithelial markers in M0, M1, and M2 cells by Western blotting and qRT-PCR. Results showed that protein expression levels of mesenchymal markers such as ZEB1, Vimentin, N-cadherin, and Twist1 were relatively lower in M1 and M2 cells than in M0 cells. On the contrary, protein expression of E-cadherin, an epithelial marker, was higher in M2 cells than in M0 cells (Fig. 2g; Additional file 3: Fig. S1D). qRT-PCR results were similar to Western blotting results: mRNA levels of mesenchymal markers were lower, and those of epithelial markers were higher in M2 cells than in M0 cells (Fig. 2h). These results imply that metastatic lung cancer cells can lose their proliferative and migratory activities, gain adhesion activity, and undergo MET after brain colonization.

### Brain-colonized lung cancer cells are less invasive than parental cells

**Fig. 3:**
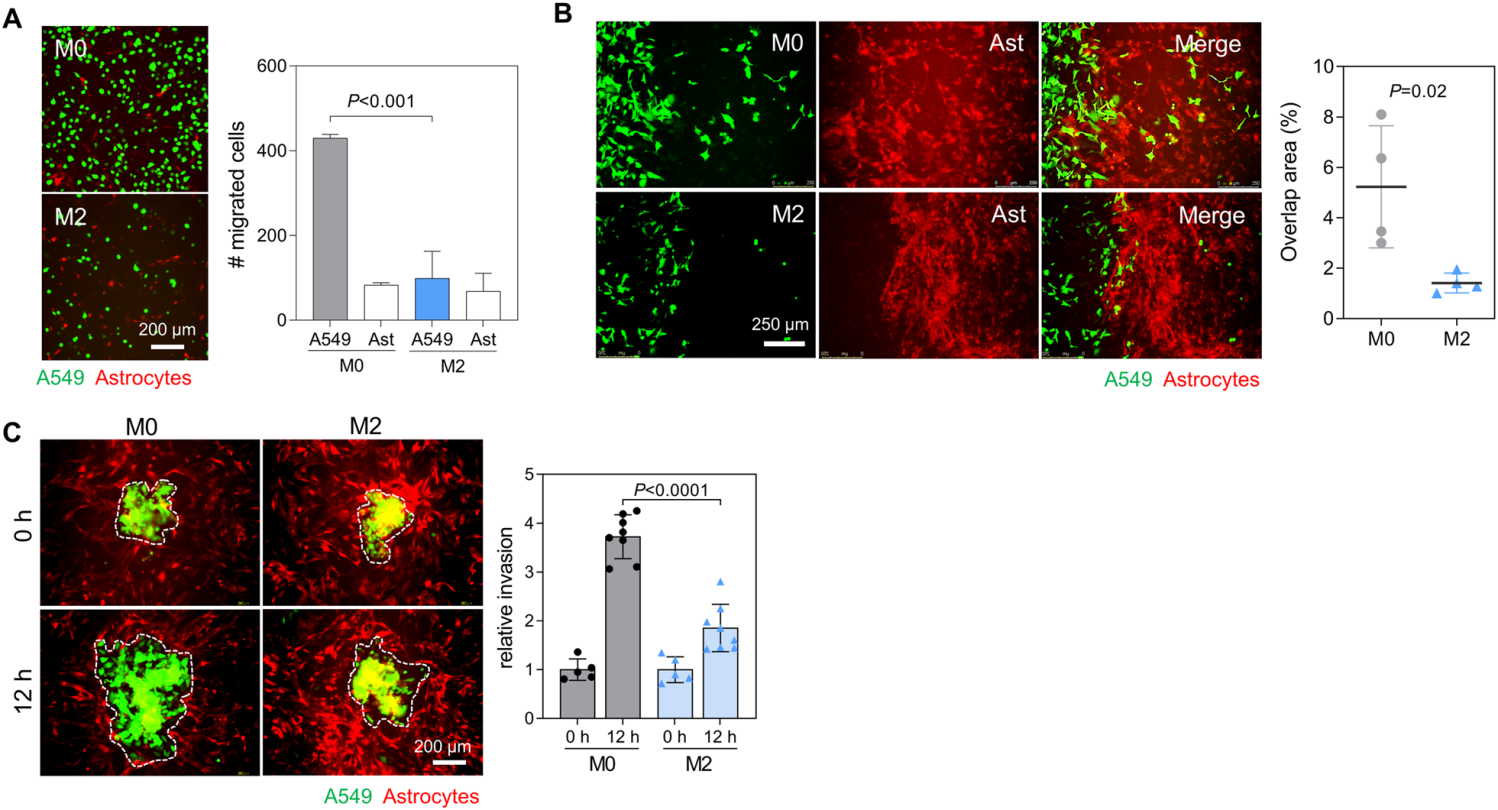
Brain colonization suppresses invasiveness of metastatic lung cancer cells. **A** Transwell migration assay of A549-M0 and M2 cells co-cultured with astrocytes. GFP-labelled M0 and M2 cells (5 × 10^4^/insert) and mCherry-labelled astrocytes (5 × 10^4^/insert) were cultured in the upper wells for 24 h. Migrated A549 cells and astrocytes were counted under a fluorescence microscope. Mean ± SD (n = 3). *P*, unpaired two-tailed Student’s *t*-test. **B** Three-well migration assay of M0 and M2 cells co-cultured with astrocytes. Lung cancer cells and astrocytes were seeded onto either side of the Culture-Insert 2 Well in a 35-mm dish. Cells were then allowed to migrate toward the center and close the wound for 48 h. Overlap areas between two types of cells were measured using ImageJ. Mean ± SD (n = 4). *P*, unpaired two-tailed Student’s *t*-test. **C** Co-culture of A549 spheroids on astrocyte feeder layers. A549-M0 or M2 spheroids (green) were overlaid on top of confluent astrocyte feeder layers (red). Invasion of A549 spheroids was observed after 12 h. Spheroid invasion area was measured by ImageJ

We next investigated the interaction between lung cancer cells and astrocytes during brain colonization using co-culture systems. Both GFP-labelled lung cancer cells and mCherry-labelled astrocytes were seeded onto upper inserts of the transwell system. Migrated cells were then observed under a fluorescence microscope. Motility of astrocytes was independent of co-cultured lung cancer cells. However, M0 cells migrated much better than M2 cells when co-cultured with astrocytes (Fig. 3a). Lung cancer cells and astrocytes were then seeded onto either side of a Culture-Insert 2 Well in a 35-mm dish. Cells were allowed to migrate toward the center and close the wound. M0 cells interacted and mixed well with astrocytes. However, M2 cells could not (Fig. 3b). When spheroids generated with M0 or M2 cells were overlaid on astrocyte feeder layers, M0 spheroids invaded and expanded into feeder astrocytes better than M2 spheroids (Fig. 3c). These data suggest that metastatic lung cancer cells become less invasive after brain colonization. In cocultures with astrocytes, M0 cells demonstrated migratory and invasive properties, whereas M2 cells exhibited a tendency to adhere to or remain in contact with astrocytes, which facilitated their colonization in the brain microenvironment.

### Reduction of Noggin during brain colonization suppresses lung cancer cell migration and invasion

**Fig. 4:**
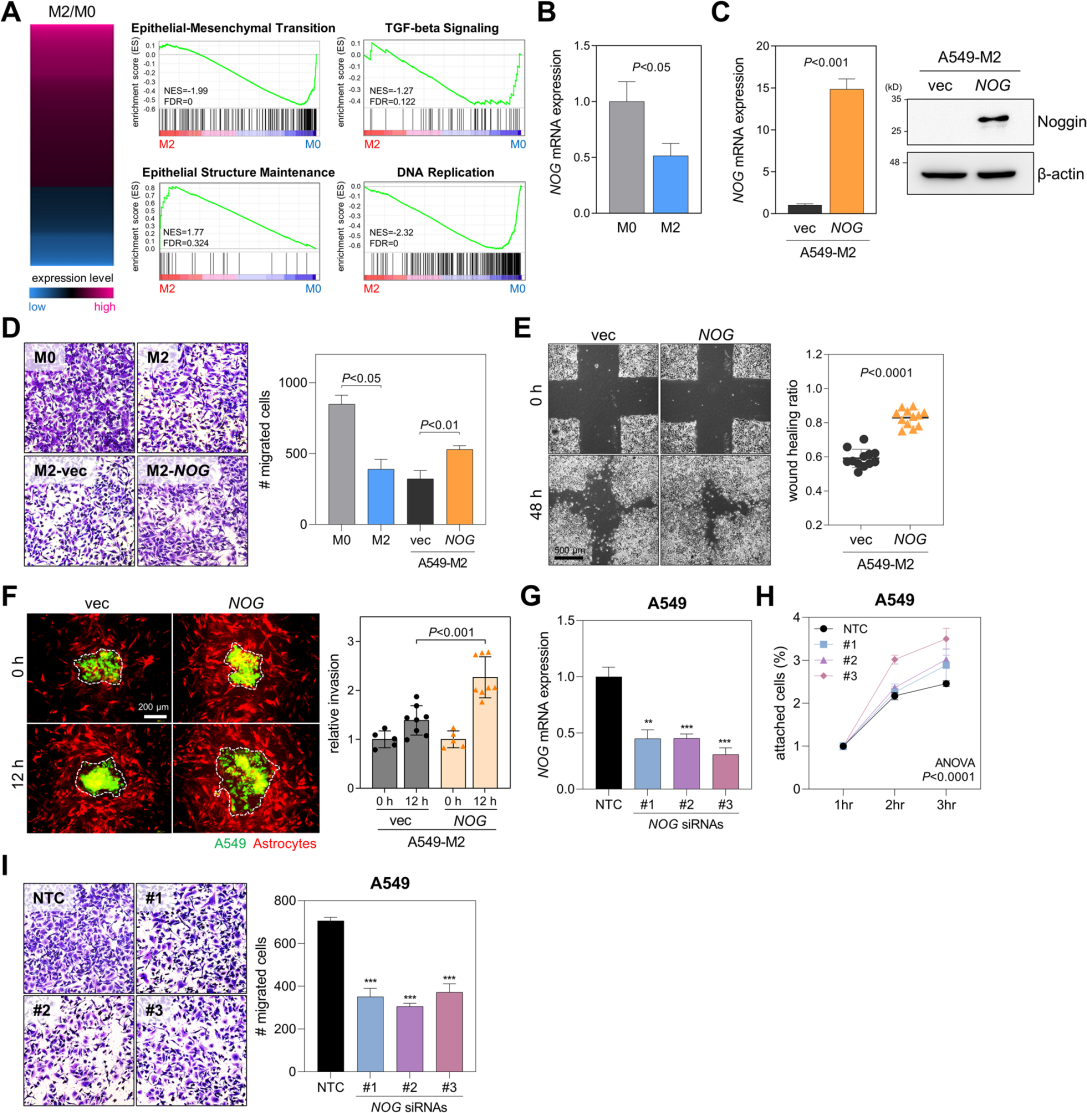
Depletion of Noggin after brain colonization causes reduced motility and invasiveness of lung cancer cells. **A** Heatmap showing RNA sequencing data obtained from M0 and M2 cells (left). Red: up-regulated; Blue: down-regulated in M2 cells. Gene set enrichment analysis of genes differentially expressed between A549-M0 and M2 cells (right). Normalized enrichment score (NES) and false discovery rate (FDR) are shown in the graph. **B** qRT-PCR of *NOG* mRNA in M0 and M2 cells. Expression levels were normalized to *RPL32* mRNA levels. *P*, unpaired two-tailed Student’s *t*-test. **C** Overexpression of Noggin in M2 cells. qRT-PCR analysis of *NOG* mRNA in M2 cells transfected with *NOG* cDNA or an empty vector (right, pLVX-Neo). Expression levels were normalized to *RPL32* mRNA levels. *P*, two-tailed Student’s *t*-test. Western blot results of Noggin in M2 cells transfected with *NOG* cDNA (right). β-actin was used as a loading control. **D** Transwell migration assay of M0, M2, and Noggin-transfected M2 cells. Cells (1 × 10^5^/insert) were cultured in upper wells for 24 h. Migrated cells were counted after staining with crystal violet. Mean ± SD (n = 3). *P*, unpaired two-tailed Student’s *t*-test. **E** Wound healing assay of Noggin-transfected M2 cells. Confluent cell cultures were subjected to scratch wounds and then incubated for 48 h in the presence of mitomycin C (1 μg/mL) to attenuate the proliferation-caused effect. Mean ± SD (n = 12). *P*, unpaired two-tailed Student’s *t*-test. **F** Co-cultures of Noggin-transfected M2 cells on astrocyte feeder layers. Spheroids generated with Noggin-transfected M2 cells or control (green) were overlaid on top of confluent astrocyte feeder layers (red). Invasion of spheroids was observed after 12 h. Spheroid invasion area was measured by ImageJ. **G** qRT-PCR of *NOG* mRNA in A549 cells transfected with nontargeting control (NTC) or Noggin siRNAs (#1, #2, and #3). Expression levels were normalized to *RPL32* mRNA levels. **P < 0.01, ***P < 0.001; unpaired two-tailed Student’s *t*-test. **H** Cell attachment assays of A549 cells transfected with NTC or Noggin siRNAs. Mean ± SD (n = 3). *P*, two-way ANOVA with Bonferroni post hoc tests. The adjusted *P*-values for NTC versus #1, NTC versus #2, and NTC versus #3 are 0.082, 0.0064, and < 0.0001, respectively. **I** Transwell migration assay of A549 cells transfected with NTC or Noggin siRNAs. Mean ± SD (n = 3). *P*, unpaired two-tailed Student’s *t*-test

To detect changes at the gene expression level, we performed RNA sequencing using M0 and M2 cells (Fig. 4a). Gene set enrichment analysis (GSEA) revealed that gene signatures including epithelial–mesenchymal transition (normalized enrichment score, NES =  − 1.99), TGF-beta signaling (NES =  − 1.27), and DNA replication (NES =  − 2.32) were depleted in M2 cells. However, epithelial structure maintenance (NES = 1.77) gene signature was enriched in M2 cells, consistent with mesenchymal–epithelial transition induction and growth inhibition in M2 cells as described above. GSEA revealed that gene signatures associated with EMT and TGFβ signal were downregulated in A549-M2 cells compared to those in parental cells, consistent with the data obtained from cell migration and invasion assays. This means that M2 cells undergo MET, which is essential for late stage of metastatic colonization [26]. Cancer cells undergoing MET show an increase in cell–cell and cell–matrix adhesion and become less able to migrate and invade surrounding tissue. Early stages of metastasis benefit from the EMT. However, the colonization potential is greatly improved in cells undergoing MET. Given that the mouse model used in this study was optimized for metastatic colonization, M2 cells known to undergo MET favorable colonization were selected though this technique. Among downregulated genes in M2 cells, Noggin, a BMP antagonist [16, 21], was selected for further studies (Fig. 4b). We overexpressed Noggin in M2 cells (Fig. 4c) and checked its effects on cancer cell migration. As expected, Noggin overexpression restored suppressed migration of M2 cells in transwell (Fig. 4d) and wound-healing assays (Fig. 4e). In spheroid overlay assay, Noggin-overexpressed M2 cells invaded and expanded into feeder astrocytes better than control cells (Fig. 4f). In addition, Noggin knockdown by siRNAs in control A549 cells promoted cell attachment (Fig. 4h) and suppressed cell migration (Fig. 4i). To evaluate clinical relevance of these findings, we performed immunohistochemical staining for Noggin using tumor samples obtained from two patients whose surgical tissues were obtained from pairs of primary lung tumors and brain-metastatic tumors. The result revealed that Noggin levels were notably reduced in brain-metastatic tumors as compared to their primary lung tumor counterparts (Additional file 3: Fig. S2A). Kaplan–Meier analysis using public data (http://kmplot.com) revealed that reduced Noggin expression is linked to poorer overall and post-progression survival in patients with lung adenocarcinoma (Additional file 3: Fig. S2B). These results suggest that Noggin reduction during brain colonization can cause inhibition of migration and invasion of metastatic lung cancer cells.

## Discussion

In the present study, a brain metastasis-optimized cell line was acquired through repetitive injection of A549 cells into the carotid artery of mouse. Systemic inoculation recapitulates several steps of the metastatic cascade including survival in circulation and extravasation through the BBB as well as metastatic colonization. However, intracarotid administration neglects earlier steps of the cascade such as impact of primary tumors on the development of the pre-metastatic niche [22]. Generally, systemic administration of cancer cell lines (parental cell lines) into mice does not exhibit a significant propensity to produce brain metastasis [23, 24]. However, parental cancer cell lines are heterogeneous. They comprise subclones with intrinsic or acquired ability to target the brain. To identify subclones exhibiting a propensity for brain tropism, parental cancer cells can be administered systemically through intracarotid injection. Rare clones located in the brain are subsequently isolated. Cancer cells recovered from these brains are then expanded in vitro, established as a cell line, and re-injected into mice. Typically, repeating the in vivo selection process (3–5 times) can enhance the ability of selected cancer cells to target the brain. Consequently, the final cancer cell line obtained through in vivo selection in the brain is designated as a brain metastasis-optimized cell line. It can consistently metastasize to the brain in a significant proportion of inoculated mice [24–26].

Brain-colonized metastatic lung cancer cells, A549-M2, grew slower and were less motile than parental M0 cells. Metastatic lung cancer cells lose their proliferative and migratory activities and undergo MET after brain colonization. Parental M0 cells possess a strong capability to migrate and invade, enabling them to penetrate the brain. However, in order to effectively colonize in the brain, they must undergo MET [27]. M2 cells, which have been repeatedly implanted in the brain, have already undergone MET and as a result, appear to possess the ability to initiate metastatic colonization rather than migration and invasion capacity.

The RNA sequencing data of M0 and M2 cells were analyzed using GSEA, which supported the findings from the cell-based experiments. GSEA revealed that a gene signature related to DNA replication was depleted in M2 cells, consistent with the observation that M2 cells grow slower than M0 cells. In addition, the analysis showed that M2 cells had a depleted signature of EMT and an enriched signature of epithelial structure maintenance, indicating that M2 cells had undergone MET. Moreover, TGF-beta signaling pathway, which involves the activity of Noggin and BMP proteins, was differentially regulated in M2 cells compared to M0 cells. This observation prompted further investigation of the role of Noggin in the context of M2 cells.

Ectopic expression of Noggin, which was downregulated in M2 cells, resulted in recovery of motility and invasiveness of M2 cells, implying that Noggin could mediate the MET process and subsequent metastatic colonization. Noggin functions as a secreted inhibitor of bone morphogenetic protein signaling, which is involved in various cellular processes including cell differentiation, survival, migration, and angiogenesis [15]. Noggin expression is downregulated in various types of cancer, including stomach, prostate, and lung cancers. Its loss can contribute to tumorigenesis and metastasis [16, 21, 28]. For example, low levels of Noggin have been associated with a more aggressive phenotype and increased invasion and metastasis of lung cancer cells [17, 28]. In addition, Noggin has been found to be able to inhibit angiogenesis in lung cancer models [17], suggesting that its loss or downregulation in lung cancer cells may contribute to angiogenic switch that occurs in tumor progression. Here, we found that Noggin downregulation also facilitated brain metastatic colonization of lung cancer cells. All these studies suggest that Noggin plays an important role in regulating lung cancer progression and metastasis.

Treatment strategies for brain metastasis are currently aiming at minimizing neurological problems. However, there is a shortage of effective methods to prevent the occurrence of brain metastases. Recently, systemic therapies have become a part of the treatment paradigm, particularly for patients with ALK-rearranged non-small cell lung cancer. Second-generation ALK tyrosine kinase inhibitors such as alectinib and brigatinib have been specifically developed to overcome the BBB, which is a significant weakness of the first-generation drug crizotinib. These newer drugs have shown clear superiority over crizotinib, delivering higher intracranial response rates and longer progression-free survival [29, 30]. Intracranial activity is not limited to ALK-rearranged or EGFR-mutant non-small cell lung cancer. In the FLAURA study, patients with measurable intracranial metastases showed a response rate of 91% (20 out of 22) to osimertinib [31]. It might be necessary to evaluate the impact of traditional chemotherapy and radiation therapy on M0 and M2 cells, respectively. If the current treatment is ineffective against M2, a change in the target of treatment might need to be considered. In cases of lung cancer with brain metastasis that do not have targetable genetic alterations, Noggin could be an alternative target for preventing the progression of brain metastasis. Studies have reported that treatment with recombinant Noggin protein can effectively normalize hypoxia-induced pulmonary arterial smooth muscle cell proliferation and that restoring Noggin levels might be a successful way of inhibiting cell proliferation by suppressing calcium entry [32].

Astrocytes are the most plentiful type of mesenchymal cells found in the brain environment. They play a crucial role in supporting and maintaining homeostasis within the central nervous system. Through co-culture experiments with astrocytes, we found that metastatic lung cancer cells became less invasive after brain colonization. Astrocytes play a crucial role in the context of lung cancer cell metastasis to the brain, particularly within the brain's environment. Their interactions with cancer cells create a supportive environment for tumor growth and survival [33]. Astrocytes can generate growth factors, cytokines, and extracellular matrix (ECM) proteins that enhance the proliferation and survival of tumor cells [24]. They also contribute to the remodeling of the ECM, aiding cancer cells in invading and colonizing themselves in brain tissue [34]. Regarding their interaction with astrocytes, A549-M2 cells exhibited reduced mobility but displayed a tendency to remain in contact with astrocytes. This behavior suggests that A549-M2 cells may have developed an affinity for settling within brain tissue.

Lung cancer cells that interact with astrocytes show increased expression of endothelin-1, which can activate intracellular signaling pathways including PI3K/AKT and MAPK pathways through endothelin-1 receptors, ultimately contributing to colonization and hemoresistance of metastatic lung cancer cells [35]. One interesting aspect of brain TME is reprogramming of microglia and astrocytes. Changes in M1/M2 microglia and macrophage polarization can alter the environment for brain metastasis, leading to fibrosis, immune suppression, angiogenesis, and changes in synaptic plasticity [36]. Despite the limitation of not including a comprehensive examination of the immune microenvironment in this study, we intend to explore this further in follow-up studies.

## Conclusions

Our findings demonstrate that during brain metastatic colonization, lung cancer cells undergo MET and exhibit decreased motility and invasiveness, which is dependent on Noggin. Thus, Noggin might provide a potential therapeutic target for inhibiting brain metastasis progression in a subset of patients with lung cancer.

### Supplementary Information

**Additional file 1: Table S1.** Differentially expressed genes (DEGs) between M0 and M2 cells analyzed by RNA sequencing.

**Additional file 2: Table S2.** qRT-PCR primer sequences used in this study.

**Additional file 3.** Method and figures.

## Data Availability

The data generated in the present study was deposited in Korean Nucleotide Archive (KoNA, https://kobic.re.kr/kona) with the accession ID, PRJKA230587.

## Abbreviations

ATCC: American Type Culture Collection
BBB: Blood–brain barrier
EMT: Epithelial–mesenchymal transition
GSEA: Gene set enrichment analysis
MET: Mesenchymal–epithelial transition
NSCLC: Non-small cell lung cancer
qRT-PCR: Quantitative real-time reverse transcription PCR
