# Supplementary material for: Noggin contributes to brain metastatic colonization of lung cancer cells
Source: Cancer Cell Int. 2023 Nov 28;23:299. doi: 10.1186/s12935-023-03155-7 (PMC10683317; doi:10.1186/s12935-023-03155-7)
Supplement: Supplementary file 1 — Additional file 1: Table S1. Differentially expressed genes (DEGs) between M0 and M2 cells analyzed by RNA sequencing. [file 12935_2023_3155_MOESM1_ESM.docx]

**Supplementary Table 1**. Differentially expressed genes (DEGs) between M0 and M2 cells analyzed by RNA sequencing

| Gene_ID | Transcript_ID | Gene_Symbol | Description | AM2/AM0.fc | AM2/AM0.logCPM | AM2/AM0.raw.pval | AM2/AM0.bh.pval |
| --- | --- | --- | --- | --- | --- | --- | --- |
| 3853 | NM_005554 | KRT6A | keratin 6A | -23767.486888 | 3.277947 | 2.86201E-16 | 1.18401E-12 |
| 56479 | NM_001160130KCNQ5 | | potassium voltage-gated channel subfamily Q member 5 | -12656.911824 | 2.376411 | 1.44606E-14 | 2.99117E-11 |
| 1301 | NM_001190709COL11A1 | | collagen type XI alpha 1 chain | -7759.383016 | 1.669769 | 3.00862E-13 | 3.95711E-10 |
| 1009 | NM_001308392CDH11 | | cadherin 11 | -6681.609686 | 1.462600 | 7.60635E-13 | 8.28091E-10 |
| 10891 | NM_001330751PPARGC1A | | PPARG coactivator 1 alpha | -5199.671357 | 1.097148 | 3.5719E-12 | 2.73647E-09 |
| 5789 | NM_001040712PTPRD | | protein tyrosine phosphatase, receptor type D | -3535.462538 | 0.611526 | 3.82454E-11 | 1.88358E-08 |
| 1961 | NM_001965 EGR4 | | early growth response 4 | -3297.718421 | 0.448639 | 5.88133E-11 | 2.70345E-08 |
| 728215 | NM_001080396FAM155A | | family with sequence similarity 155 member A | -2861.854207 | 0.247594 | 1.3874E-10 | 5.40596E-08 |
| 25859 | NR_024617,NR PART1 | | prostate androgen-regulated transcript 1 (non-protein codi | -2853.929403 | 0.243666 | 1.41127E-10 | 5.40596E-08 |
| 282618 | NM_172140 IFNL1 | | interferon lambda 1 | -2663.734110 | 0.146038 | 2.15662E-10 | 7.31308E-08 |
| 94115 | NM_033183 CGB8 | | chorionic gonadotropin beta subunit 8 | -2592.410874 | 0.151327 | 2.54794E-10 | 8.36575E-08 |
| 143425 | NM_175733 SYT9 | | synaptotagmin 9 | -2354.666758 | 0.088696 | 4.59789E-10 | 1.30284E-07 |
| 84648 | NM_032563 LCE3D | | late cornified envelope 3D | -2212.020287 | -0.116355 | 6.74329E-10 | 1.78827E-07 |
| 3627 | NM_001565 CXCL10 | | C-X-C motif chemokine ligand 10 | -2124.847445 | -0.141052 | 8.62509E-10 | 2.2256E-07 |
| 374918 | NM_198541 IGFL1 | | IGF like family member 1 | -2069.373817 | -0.150715 | 9.90586E-10 | 2.41062E-07 |
| 387590 | NR_001591 TPTEP1 | | transmembrane phosphatase with tensin homology pseudo | -2045.599406 | -0.226486 | 1.06283E-09 | 2.55635E-07 |
| 6373 | NM_001302123CXCL11 | | C-X-C motif chemokine ligand 11 | -1847.479308 | -0.333083 | 1.97505E-09 | 4.48943E-07 |
| 91133 | NM_001330559L3MBTL4 | | l(3)mbt-like 4 (Drosophila) | -1530.487152 | -0.596536 | 6.17697E-09 | 1.20538E-06 |
| 81285 | NM_030774 OR51E2 | | olfactory receptor family 51 subfamily E member 2 | -1459.163917 | -0.699602 | 8.23844E-09 | 1.48185E-06 |
| 26253 | NM_014358 CLEC4E | | C-type lectin domain family 4 member E | -1387.840682 | -0.737237 | 1.11419E-08 | 1.95313E-06 |
| 353145 | NM_178435 LCE3E | | late cornified envelope 3E | -1237.269408 | -0.928824 | 2.13729E-08 | 3.42713E-06 |
| 200504 | NM_182536 GKN2 | | gastrokine 2 | -1165.946173 | -1.010991 | 3.04232E-08 | 4.59346E-06 |
| 401509 | NR_003528,NR ZNF658B | | zinc finger protein 658B (pseudogene) | -1086.698134 | 0.038361 | 4.61748E-08 | 6.3675E-06 |
| 115361 | NM_052941 GBP4 | | guanylate binding protein 4 | -1047.074114 | -0.810989 | 5.75113E-08 | 7.62577E-06 |
| 282616 | NM_172138 IFNL2 | | interferon lambda 2 | -951.976468 | -1.290042 | 1.00754E-07 | 1.21877E-05 |
| 6352 | NM_001278736CCL5 | | C-C motif chemokine ligand 5 | -911.976960 | 4.828280 | 2.68085E-15 | 7.46881E-12 |
| 6348 | NM_002983 CCL3 | | C-C motif chemokine ligand 3 | -904.427644 | -1.360153 | 1.3606E-07 | 1.54638E-05 |
| 55531 | NM_001130037ELMOD1 | | ELMO domain containing 1 | -880.653233 | -1.144111 | 1.59E-07 | 1.73101E-05 |
| 401014 | NR_033870 TEX41 | | testis expressed 41 (non-protein coding) | -856.878821 | -1.433857 | 1.86549E-07 | 1.94887E-05 |
| 154064 | NM_130900 RAET1L | | retinoic acid early transcript 1L | -825.179605 | -1.070698 | 2.32365E-07 | 2.33324E-05 |
| 57451 | NM_001080428TENM2 | | teneurin transmembrane protein 2 | -801.405194 | -1.524907 | 2.75404E-07 | 2.63738E-05 |
| 143503 | NM_152430 OR51E1 | | olfactory receptor family 51 subfamily E member 1 | -801.405194 | -1.524907 | 2.75404E-07 | 2.63738E-05 |
| 282617 | NM_001346937IFNL3 | | interferon lambda 3 | -793.480390 | -1.538398 | 2.91758E-07 | 2.75572E-05 |
| 728695 | NM_032461,N SPANXB1 | | SPANX family member B1 | -706.307547 | -1.181965 | 5.71537E-07 | 4.86512E-05 |
| 4685 | NM_004540 NCAM2 | | neural cell adhesion molecule 2 | -690.457939 | -1.726368 | 6.51161E-07 | 5.47531E-05 |
| 6288 | NM_000331,N SAA1 | | serum amyloid A1 | -674.608331 | 2.002200 | 7.43944E-07 | 6.10654E-05 |
| 4843 | NM_000625 NOS2 | | nitric oxide synthase 2 | -650.833920 | -1.191260 | 9.13479E-07 | 7.26743E-05 |
| 27128 | NM_001318024CYTH4 | | cytohesin 4 | -619.134704 | -0.688845 | 1.2143E-06 | 9.30287E-05 |
| 3785 | NM_004518,N KCNQ2 | | potassium voltage-gated channel subfamily Q member 2 | -611.209900 | -1.661994 | 1.30657E-06 | 9.82778E-05 |
| 102723692 | NR_135179 LOC102723692 | | uncharacterized LOC102723692 | -611.209900 | -1.755295 | 1.30657E-06 | 9.82778E-05 |

| 89886 | NM_001146172SLAMF9 | SLAM family member 9 | -595.360292 | -1.646459 | 1.51666E-06 | 0.000109693 |
| --- | --- | --- | --- | --- | --- | --- |
| 55511 | NM_018666 SAGE1 | sarcoma antigen 1 | -579.510685 | -1.960728 | 1.76696E-06 | 0.00012224 |
| 176 | NM_001135,N ACAN | aggrecan | -571.585881 | -1.836396 | 1.90992E-06 | 0.00012953 |
| 1565 | NM_000106,N CYP2D6 | cytochrome P450 family 2 subfamily D member 6 | -571.585881 | -1.104454 | 1.90992E-06 | 0.00012953 |
| 3861 | NM_000526 KRT14 | keratin 14 | -571.585881 | -1.979026 | 1.90992E-06 | 0.00012953 |
| 55301 | NM_001039702OLAH | oleoyl-ACP hydrolase | -571.585881 | -1.870767 | 1.90992E-06 | 0.00012953 |
| 8549 | NM_001277226LGR5 | leucine rich repeat containing G protein-coupled receptor 5 | -563.661077 | -1.960103 | 2.06647E-06 | 0.000137003 |
| 109504726 | NM_001348050ERV3-1-ZNF117 | ERV3-1-ZNF117 readthrough | -563.661077 | -0.257527 | 2.06647E-06 | 0.000137003 |
| 27253 | NM_001040429PCDH17 | protocadherin 17 | -547.811469 | -2.035361 | 2.4265E-06 | 0.000158335 |
| 1082 | NM_000737 CGB3 | chorionic gonadotropin beta subunit 3 | -516.112253 | -2.114083 | 3.38925E-06 | 0.00021053 |
| 5800 | NM_002848,N PTPRO | protein tyrosine phosphatase, receptor type O | -508.187449 | -2.033752 | 3.69514E-06 | 0.000225599 |
| 55879 | NM_018558 GABRQ | gamma-aminobutyric acid type A receptor theta subunit | -500.262646 | -1.688511 | 4.03352E-06 | 0.000242539 |
| 115362 | NM_001134486GBP5 | guanylate binding protein 5 | -500.262646 | -2.072877 | 4.03352E-06 | 0.000242539 |
| 6588 | NM_003063 SLN | sarcolipin | -484.413038 | -2.197370 | 4.82427E-06 | 0.000281099 |
| 387700 | NM_213606 SLC16A12 | solute carrier family 16 member 12 | -484.413038 | -2.072224 | 4.82427E-06 | 0.000281099 |
| 3456 | NM_002176 IFNB1 | interferon beta 1 | -460.638626 | -2.263165 | 6.37378E-06 | 0.000356811 |
| 6317 | NM_006919 SERPINB3 | serpin family B member 3 | -460.638626 | -1.782231 | 6.37378E-06 | 0.000356811 |
| 245972 | NM_152565 ATP6V0D2 | ATPase H+ transporting V0 subunit d2 | -452.713822 | -2.174395 | 7.01372E-06 | 0.000385943 |
| 440896 | NR_015361,NR LOC440896 | uncharacterized LOC440896 | -452.713822 | -2.031500 | 7.01372E-06 | 0.000385943 |
| 4103 | NM_001011548MAGEA4 | MAGE family member A4 | -444.789018 | -2.308765 | 7.72941E-06 | 0.000416361 |
| 100288695 | NM_001205288LIMS4 | LIM zinc finger domain containing 4 | -413.089803 | -1.685429 | 1.04431E-05 | 0.000530748 |
| 27033 | NM_001316902ZBTB32 | zinc finger and BTB domain containing 32 | -405.164999 | -2.429531 | 1.15831E-05 | 0.000574573 |
| 56704 | NM_001317830JPH1 | junctophilin 1 | -405.164999 | -2.283666 | 1.15831E-05 | 0.000574573 |
| 251 | NM_031313 ALPPL2 | alkaline phosphatase, placental like 2 | -389.315391 | -2.480832 | 1.4326E-05 | 0.000679665 |
| 390732 | NR_033740 CES1P2 | carboxylesterase 1 pseudogene 2 | -381.390587 | -2.237076 | 1.59765E-05 | 0.000749374 |
| 100526772 | NM_001198974TMEM110-MUST | TMEM110-MUSTN1 readthrough | -381.390587 | -2.507186 | 1.59765E-05 | 0.000749374 |
| 9369 | NM_001105250NRXN3 | neurexin 3 | -380.283890 | 1.424815 | 2.57434E-11 | 1.40132E-08 |
| 139065 | NM_001184749SLITRK4 | SLIT and NTRK like family member 4 | -373.465783 | -2.353328 | 1.78517E-05 | 0.00082241 |
| 60676 | NM_020318,N PAPPA2 | pappalysin 2 | -365.540979 | -2.479700 | 1.9987E-05 | 0.000910643 |
| 1437 | NM_000758 CSF2 | colony stimulating factor 2 | -357.616175 | 1.267410 | 2.24245E-05 | 0.000995387 |
| 9248 | NM_004224 GPR50 | G protein-coupled receptor 50 | -349.691371 | -2.505664 | 2.52136E-05 | 0.001095679 |
| 51676 | NM_001202429ASB2 | ankyrin repeat and SOCS box containing 2 | -341.766568 | -1.118849 | 2.84133E-05 | 0.001211813 |
| 3363 | NM_000872,N HTR7 | 5-hydroxytryptamine receptor 7 | -333.841764 | -2.375987 | 3.20938E-05 | 0.001357587 |
| 2555 | NM_000807,N GABRA2 | gamma-aminobutyric acid type A receptor alpha2 subunit | -317.992156 | -2.737491 | 4.12497E-05 | 0.001637714 |
| 6507 | NM_001166695SLC1A3 | solute carrier family 1 member 3 | -317.992156 | -2.737491 | 4.12497E-05 | 0.001637714 |
| 9075 | NM_001171092CLDN2 | claudin 2 | -311.144790 | 5.326961 | 6.42967E-13 | 7.38876E-10 |
| 6351 | NM_002984 CCL4 | C-C motif chemokine ligand 4 | -310.067352 | -2.769061 | 4.69471E-05 | 0.001818542 |
| 100126784 | NR_015384 LOC100126784 | uncharacterized LOC100126784 | -310.067352 | -2.326379 | 4.69471E-05 | 0.001818542 |
| 88 | NM_001103,N ACTN2 | actinin alpha 2 | -302.142548 | -2.801342 | 5.35781E-05 | 0.001996869 |
| 23349 | NM_001304333PHF24 | PHD finger protein 24 | -302.142548 | -2.644753 | 5.35781E-05 | 0.001996869 |
| 53836 | NM_023915 GPR87 | G protein-coupled receptor 87 | -302.142548 | -2.558293 | 5.35781E-05 | 0.001996869 |
| 100134444 | NM_001194958KCNJ18 | potassium voltage-gated channel subfamily J member 18 | -302.142548 | -2.801342 | 5.35781E-05 | 0.001996869 |
| 102723631 | NM_001291527CT45A10 | cancer/testis antigen family 45 member A10 | -302.142548 | -2.801342 | 5.35781E-05 | 0.001996869 |

| 9023 | NM_003956 | CH25H | cholesterol 25-hydroxylase | -294.217744 | -2.233256 | 6.13206E-05 | 0.002217511 |
| --- | --- | --- | --- | --- | --- | --- | --- |
| 283807 | NM_203373 | FBXL22 | F-box and leucine rich repeat protein 22 | -294.217744 | -2.211044 | 6.13206E-05 | 0.002217511 |
| 1117 | NM_001025197CHI3L2 | | chitinase 3 like 2 | -286.292940 | -2.868165 | 7.03917E-05 | 0.002430804 |
| 6367 | NM_002990 CCL22 | | C-C motif chemokine ligand 22 | -286.292940 | -2.704573 | 7.03917E-05 | 0.002430804 |
| 7146 | NR_001284 TNXA | | tenascin XA (pseudogene) | -286.292940 | -2.868165 | 7.03917E-05 | 0.002430804 |
| 90592 | NM_001271848ZNF700 | | zinc finger protein 700 | -286.292940 | -2.868165 | 7.03917E-05 | 0.002430804 |
| 147906 | NM_001301046DACT3 | | dishevelled binding antagonist of beta catenin 3 | -286.292940 | -2.104471 | 7.03917E-05 | 0.002430804 |
| 284415 | NM_001288791VSTM1 | | V-set and transmembrane domain containing 1 | -286.292940 | -2.643957 | 7.03917E-05 | 0.002430804 |
| 100133093 | NM_001137549FAM25G | | family with sequence similarity 25 member G | -286.292940 | -2.673949 | 7.03917E-05 | 0.002430804 |
| 57575 | NM_020815,N PCDH10 | | protocadherin 10 | -278.368136 | -2.145699 | 8.10571E-05 | 0.002744133 |
| 63982 | NM_001313726ANO3 | | anoctamin 3 | -270.443333 | -2.556743 | 9.36436E-05 | 0.003104194 |
| 84675 | NM_033058,N TRIM55 | | tripartite motif containing 55 | -270.443333 | -2.277666 | 9.36436E-05 | 0.003104194 |
| 8900 | NM_001111045CCNA1 | | cyclin A1 | -262.518529 | -2.832680 | 0.000108555 | 0.003497601 |
| 123041 | NM_153646,N SLC24A4 | | solute carrier family 24 member 4 | -262.518529 | -2.023782 | 0.000108555 | 0.003497601 |
| 54674 | NM_001099658LRRN3 | | leucine rich repeat neuronal 3 | -254.593725 | -3.011938 | 0.000126293 | 0.00394617 |
| 137868 | NM_001322879SGCZ | | sarcoglycan zeta | -254.593725 | -3.011938 | 0.000126293 | 0.00394617 |
| 152573 | NM_001080505SHISA3 | | shisa family member 3 | -254.593725 | -3.011938 | 0.000126293 | 0.00394617 |
| 1634 | NM_001920,N DCN | | decorin | -246.668921 | -2.866039 | 0.000147483 | 0.004440584 |
| 1436 | NM_001288705CSF1R | | colony stimulating factor 1 receptor | -238.744117 | -2.641568 | 0.000172911 | 0.005044328 |
| 2250 | NM_001291812FGF5 | | fibroblast growth factor 5 | -238.744117 | -2.865613 | 0.000172911 | 0.005044328 |
| 161357 | NM_001113498MDGA2 | | MAM domain containing glycosylphosphatidylinositol anch | -238.744117 | -3.089613 | 0.000172911 | 0.005044328 |
| 104355135 | NR_126378 LINC00431 | | long intergenic non-protein coding RNA 431 | -238.744117 | -2.527504 | 0.000172911 | 0.005044328 |
| 1620 | NM_014618 BRINP1 | | BMP/retinoic acid inducible neural specific 1 | -230.819313 | -2.900204 | 0.000203569 | 0.005721223 |
| 1842 | NM_001197295ECM2 | | extracellular matrix protein 2 | -230.819313 | -2.527120 | 0.000203569 | 0.005721223 |
| 4314 | NM_002422 MMP3 | | matrix metallopeptidase 3 | -222.894509 | -3.088710 | 0.000240716 | 0.006534407 |
| 8685 | NM_006770 MARCO | | macrophage receptor with collagenous structure | -222.894509 | -3.010168 | 0.000240716 | 0.006534407 |
| 22806 | NM_001257408IKZF3 | | IKAROS family zinc finger 3 | -222.894509 | -3.171734 | 0.000240716 | 0.006534407 |
| 9037 | NM_003966 SEMA5A | | semaphorin 5A | -214.969705 | -3.214626 | 0.000285964 | 0.007393951 |
| 10044 | NM_001142531SH2D3C | | SH2 domain containing 3C | -214.969705 | -3.048462 | 0.000285964 | 0.007393951 |
| 81832 | NM_001201465NETO1 | | neuropilin and tolloid like 1 | -214.969705 | -3.214626 | 0.000285964 | 0.007393951 |
| 388372 | NM_207007,N CCL4L1 | | C-C motif chemokine ligand 4 like 1 | -214.969705 | -3.214626 | 0.000285964 | 0.007393951 |
| 388555 | NM_207393 IGFL3 | | IGF like family member 3 | -214.969705 | -3.214626 | 0.000285964 | 0.007393951 |
| 100506409 | NR_038962,NR ELOVL2-AS1 | | ELOVL2 antisense RNA 1 | -214.969705 | -2.499191 | 0.000285964 | 0.007393951 |
| 100507431 | NR_125383 LOC100507431 | | uncharacterized LOC100507431 | -214.969705 | -3.088258 | 0.000285964 | 0.007393951 |
| 1589 | NM_000500,N CYP21A2 | | cytochrome P450 family 21 subfamily A member 2 | -207.044901 | -3.128717 | 0.000341383 | 0.008456898 |
| 85235 | NM_080596 HIST1H2AH | | histone cluster 1 H2A family member h | -207.044901 | -2.394987 | 0.000341383 | 0.008456898 |
| 404203 | NM_001195290SPINK6 | | serine peptidase inhibitor, Kazal type 6 | -207.044901 | -3.258840 | 0.000341383 | 0.008456898 |
| 439936 | NR_131245 C5orf17 | | chromosome 5 open reading frame 17 | -207.044901 | -3.258840 | 0.000341383 | 0.008456898 |
| 101929099 | NR_110786 LOC101929099 | | uncharacterized LOC101929099 | -207.044901 | -2.321715 | 0.000341383 | 0.008456898 |
| 6361 | NM_002987 CCL17 | | C-C motif chemokine ligand 17 | -199.120097 | -3.304460 | 0.00040966 | 0.009751222 |
| 91543 | NM_080657 RSAD2 | | radical S-adenosyl methionine domain containing 2 | -193.709373 | 1.423438 | 2.12112E-10 | 7.31258E-08 |
| 4066 | NM_005583 LYL1 | | LYL1, basic helix-loop-helix family member | -191.195294 | -3.351577 | 0.0004943 | 0.01131223 |
| 101927854 | NR_121667 LOC101927854 | | uncharacterized LOC101927854 | -191.195294 | -3.351577 | 0.0004943 | 0.01131223 |

| 101928602 | NR_134931 | LINC01775 | long intergenic non-protein coding RNA 1775 | -191.195294 | -2.525200 | 0.0004943 | 0.01131223 |
| --- | --- | --- | --- | --- | --- | --- | --- |
| 103752587 | NR_125795 | FOXC2-AS1 | FOXC2 antisense RNA 1 | -191.195294 | -3.351577 | 0.0004943 | 0.01131223 |
| 945 | NM_001082618CD33 | | CD33 molecule | -183.270490 | -2.609440 | 0.00059992 | 0.013229577 |
| 1908 | NM_001302455EDN3 | | endothelin 3 | -175.345686 | -3.450724 | 0.000732646 | 0.01532333 |
| 3598 | NM_000640 IL13RA2 | | interleukin 13 receptor subunit alpha 2 | -175.345686 | -3.450724 | 0.000732646 | 0.01532333 |
| 6349 | NM_021006,N CCL3L1 | | C-C motif chemokine ligand 3 like 1 | -175.345686 | -3.450724 | 0.000732646 | 0.01532333 |
| 140894 | NM_001207076CNBD2 | | cyclic nucleotide binding domain containing 2 | -175.345686 | -2.969802 | 0.000732646 | 0.01532333 |
| 643161 | NM_001146157FAM25A | | family with sequence similarity 25 member A | -175.345686 | -3.168965 | 0.000732646 | 0.01532333 |
| 644524 | NM_033176 NKX2-4 | | NK2 homeobox 4 | -175.345686 | -3.450724 | 0.000732646 | 0.01532333 |
| 102724434 | NR_130921,NR LOC102724434 | | uncharacterized LOC102724434 | -175.345686 | -3.450724 | 0.000732646 | 0.01532333 |
| 247 | NM_001039130ALOX15B | | arachidonate 15-lipoxygenase, type B | -167.420882 | -3.502990 | 0.000900684 | 0.018158517 |
| 943 | NM_001243,N TNFRSF8 | | TNF receptor superfamily member 8 | -167.420882 | -2.896767 | 0.000900684 | 0.018158517 |
| 1496 | NM_001164883CTNNA2 | | catenin alpha 2 | -167.420882 | -3.502990 | 0.000900684 | 0.018158517 |
| 6279 | NM_001319196S100A8 | | S100 calcium binding protein A8 | -167.420882 | -3.168503 | 0.000900684 | 0.018158517 |
| 6318 | NM_002974,N SERPINB4 | | serpin family B member 4 | -167.420882 | -2.637984 | 0.000900684 | 0.018158517 |
| 65267 | NM_001002838WNK3 | | WNK lysine deficient protein kinase 3 | -167.420882 | -2.039413 | 0.000900684 | 0.018158517 |
| 80380 | NM_025239 PDCD1LG2 | | programmed cell death 1 ligand 2 | -167.420882 | -3.302553 | 0.000900684 | 0.018158517 |
| 85480 | NM_033035,N TSLP | | thymic stromal lymphopoietin | -167.420882 | -3.399320 | 0.000900684 | 0.018158517 |
| 729083 | NR_122070 LINC02158 | | long intergenic non-protein coding RNA 2158 | -167.420882 | -3.502990 | 0.000900684 | 0.018158517 |
| 23136 | NM_001281533EPB41L3 | | erythrocyte membrane protein band 4.1 like 3 | -159.496078 | -3.557232 | 0.001115132 | 0.021383887 |
| 57631 | NM_001243963LRCH2 | | leucine rich repeats and calponin homology domain contai | -159.496078 | -2.418030 | 0.001115132 | 0.021383887 |
| 92241 | NM_001322923RCSD1 | | RCSD domain containing 1 | -159.496078 | -3.557232 | 0.001115132 | 0.021383887 |
| 100506433 | NR_039996 LINC00648 | | long intergenic non-protein coding RNA 648 | -159.496078 | -3.557232 | 0.001115132 | 0.021383887 |
| 102724895 | NR_146458 LIVAR | | liver cell viability associated lncRNA | -159.496078 | -1.545112 | 0.001115132 | 0.021383887 |
| 9173 | NM_001282408IL1RL1 | | interleukin 1 receptor like 1 | -158.062128 | 0.269431 | 5.43636E-09 | 1.09176E-06 |
| 79160 | NR_125359 LINC01711 | | long intergenic non-protein coding RNA 1711 | -151.571274 | -3.501993 | 0.001391155 | 0.025331018 |
| 85479 | NM_001349432DNAJC5B | | DnaJ heat shock protein family (Hsp40) member C5 beta | -151.571274 | -3.255540 | 0.001391155 | 0.025331018 |
| 92591 | NM_080863 ASB16 | | ankyrin repeat and SOCS box containing 16 | -151.571274 | -3.613604 | 0.001391155 | 0.025331018 |
| 284293 | NM_001123366HMSD | | histocompatibility minor serpin domain containing | -151.571274 | -3.167580 | 0.001391155 | 0.025331018 |
| 284467 | NM_001004440FAM19A3 | | family with sequence similarity 19 member A3, C-C motif c | -151.571274 | -2.895907 | 0.001391155 | 0.025331018 |
| 100130520 | NM_001324073LOC100130520 | | CD300c molecule-like | -151.571274 | -3.501993 | 0.001391155 | 0.025331018 |
| 912 | NM_001319145CD1D | | CD1d molecule | -143.646470 | -3.397858 | 0.0017497 | 0.030261321 |
| 2313 | NM_001167681FLI1 | | Fli-1 proto-oncogene, ETS transcription factor | -143.646470 | -3.044435 | 0.0017497 | 0.030261321 |
| 2769 | NM_002068 GNA15 | | G protein subunit alpha 15 | -143.646470 | -3.501494 | 0.0017497 | 0.030261321 |
| 9421 | NM_004821 HAND1 | | heart and neural crest derivatives expressed 1 | -143.646470 | -2.607467 | 0.0017497 | 0.030261321 |
| 57010 | NM_001300895CABP4 | | calcium binding protein 4 | -143.646470 | -3.672282 | 0.0017497 | 0.030261321 |
| 80183 | NM_001286761RUBCNL | | RUN and cysteine rich domain containing beclin 1 interacti | -143.646470 | -3.672282 | 0.0017497 | 0.030261321 |
| 148014 | NM_152479 TTC9B | | tetratricopeptide repeat domain 9B | -143.646470 | -3.672282 | 0.0017497 | 0.030261321 |
| 4070 | NM_002353 TACSTD2 | | tumor associated calcium signal transducer 2 | -135.721666 | -3.733462 | 0.002220049 | 0.036130384 |
| 26628 | NR_120437,NR OR7E47P | | olfactory receptor family 7 subfamily E member 47 pseudog | -135.721666 | -3.733462 | 0.002220049 | 0.036130384 |
| 54538 | NM_001301088ROBO4 | | roundabout guidance receptor 4 | -135.721666 | -3.448259 | 0.002220049 | 0.036130384 |
| 64123 | NM_022159 ADGRL4 | | adhesion G protein-coupled receptor L4 | -135.721666 | -3.733462 | 0.002220049 | 0.036130384 |
| 92736 | NM_178160 OTOP2 | | otopetrin 2 | -135.721666 | -3.733462 | 0.002220049 | 0.036130384 |

| 134466 | NR_026867 | ZNF300P1 | zinc finger protein 300 pseudogene 1 | -135.721666 | -3.733462 | 0.002220049 | 0.036130384 |
| --- | --- | --- | --- | --- | --- | --- | --- |
| 374860 | NM_001145029ANKRD30B | | ankyrin repeat domain 30B | -135.721666 | -3.733462 | 0.002220049 | 0.036130384 |
| 440829 | NM_001207020SHISA8 | | shisa family member 8 | -135.721666 | -3.043988 | 0.002220049 | 0.036130384 |
| 793 | NM_004929 CALB1 | | calbindin 1 | -127.796862 | -3.797365 | 0.002843659 | 0.0427381 |
| 3574 | NM_000880,N IL7 | | interleukin 7 | -127.796862 | -3.043540 | 0.002843659 | 0.0427381 |
| 3854 | NM_005555 KRT6B | | keratin 6B | -127.796862 | -3.797365 | 0.002843659 | 0.0427381 |
| 11262 | NM_001005176SP140 | | SP140 nuclear body protein | -127.796862 | -3.555214 | 0.002843659 | 0.0427381 |
| 51176 | NM_001130713LEF1 | | lymphoid enhancer binding factor 1 | -127.796862 | -3.612073 | 0.002843659 | 0.0427381 |
| 54210 | NM_001242589TREM1 | | triggering receptor expressed on myeloid cells 1 | -127.796862 | -3.447766 | 0.002843659 | 0.0427381 |
| 56659 | NM_022054 KCNK13 | | potassium two pore domain channel subfamily K member 1 | -127.796862 | -3.209494 | 0.002843659 | 0.0427381 |
| 91227 | NM_001282879GGTLC2 | | gamma-glutamyltransferase light chain 2 | -127.796862 | -3.396882 | 0.002843659 | 0.0427381 |
| 284424 | NR_027148 MIR7-3HG | | MIR7-3 host gene | -127.796862 | -3.797365 | 0.002843659 | 0.0427381 |
| 57718 | NM_001348142PPP4R4 | | protein phosphatase 4 regulatory subunit 4 | -123.168219 | 1.286099 | 7.2143E-09 | 1.36906E-06 |
| 54600 | NM_021027 UGT1A9 | | UDP glucuronosyltransferase family 1 member A9 | -122.135263 | 4.412217 | 1.66135E-10 | 6.19801E-08 |
| 1761 | NM_021951 DMRT1 | | doublesex and mab-3 related transcription factor 1 | -119.872058 | -3.864247 | 0.003680029 | 0.051642736 |
| 1780 | NM_001135556DYNC1I1 | | dynein cytoplasmic 1 intermediate chain 1 | -119.872058 | -3.864247 | 0.003680029 | 0.051642736 |
| 5309 | NM_005029 PITX3 | | paired like homeodomain 3 | -119.872058 | -2.577552 | 0.003680029 | 0.051642736 |
| 5673 | NM_001130014PSG5 | | pregnancy specific beta-1-glycoprotein 5 | -119.872058 | -3.864247 | 0.003680029 | 0.051642736 |
| 64850 | NM_001146590ETNPPL | | ethanolamine-phosphate phospho-lyase | -119.872058 | -3.670733 | 0.003680029 | 0.051642736 |
| 85449 | NM_001029864KIAA1755 | | KIAA1755 | -119.872058 | -3.082833 | 0.003680029 | 0.051642736 |
| 91646 | NM_001110822TDRD12 | | tudor domain containing 12 | -119.872058 | -3.082833 | 0.003680029 | 0.051642736 |
| 101928043 | NR_110692 LINC01635 | | long intergenic non-protein coding RNA 1635 | -119.872058 | -3.209028 | 0.003680029 | 0.051642736 |
| 1747 | NM_005220 DLX3 | | distal-less homeobox 3 | -111.947255 | -3.003968 | 0.00481584 | 0.062415821 |
| 6886 | NM_001287347TAL1 | | TAL bHLH transcription factor 1, erythroid differentiation fa | -111.947255 | -3.611052 | 0.00481584 | 0.062415821 |
| 54752 | NM_017559 FNDC8 | | fibronectin type III domain containing 8 | -111.947255 | -3.611052 | 0.00481584 | 0.062415821 |
| 154664 | NM_152701 ABCA13 | | ATP binding cassette subfamily A member 13 | -111.947255 | -3.934399 | 0.00481584 | 0.062415821 |
| 284217 | NM_005559 LAMA1 | | laminin subunit alpha 1 | -111.947255 | -2.858805 | 0.00481584 | 0.062415821 |
| 338328 | NM_001301772GPIHBP1 | | glycosylphosphatidylinositol anchored high density lipoprot | -111.947255 | -3.934399 | 0.00481584 | 0.062415821 |
| 440087 | NM_001013698SMCO3 | | single-pass membrane protein with coiled-coil domains 3 | -111.947255 | -3.934399 | 0.00481584 | 0.062415821 |
| 342 | NR_028412,NR APOC1P1 | | apolipoprotein C1 pseudogene 1 | -104.022451 | -4.008155 | 0.006379424 | 0.076320644 |
| 395 | NM_001287242ARHGAP6 | | Rho GTPase activating protein 6 | -104.022451 | -4.008155 | 0.006379424 | 0.076320644 |
| 653 | NM_001329754BMP5 | | bone morphogenetic protein 5 | -104.022451 | -3.298738 | 0.006379424 | 0.076320644 |
| 972 | NM_001025158CD74 | | CD74 molecule | -104.022451 | -3.731371 | 0.006379424 | 0.076320644 |
| 4063 | NM_001033667LY9 | | lymphocyte antigen 9 | -104.022451 | -3.499000 | 0.006379424 | 0.076320644 |
| 4880 | NM_024409 NPPC | | natriuretic peptide C | -104.022451 | -4.008155 | 0.006379424 | 0.076320644 |
| 6703 | NM_006945 SPRR2D | | small proline rich protein 2D | -104.022451 | -4.008155 | 0.006379424 | 0.076320644 |
| 9478 | NM_001033677CABP1 | | calcium binding protein 1 | -104.022451 | -3.446286 | 0.006379424 | 0.076320644 |
| 22871 | NM_014932 NLGN1 | | neuroligin 1 | -104.022451 | -4.008155 | 0.006379424 | 0.076320644 |
| 50674 | NM_020999 NEUROG3 | | neurogenin 3 | -104.022451 | -4.008155 | 0.006379424 | 0.076320644 |
| 63973 | NM_024019 NEUROG2 | | neurogenin 2 | -104.022451 | -3.499000 | 0.006379424 | 0.076320644 |
| 64109 | NM_001012288CRLF2 | | cytokine receptor-like factor 2 | -104.022451 | -2.664705 | 0.006379424 | 0.076320644 |
| 126755 | NM_001010847LRRC38 | | leucine rich repeat containing 38 | -104.022451 | -4.008155 | 0.006379424 | 0.076320644 |
| 285141 | NM_001289947ERICH2 | | glutamate rich 2 | -104.022451 | -3.346274 | 0.006379424 | 0.076320644 |

| 100128385 | NR_024376 | FAM225B | family with sequence similarity 225 member B (non-protein | -104.022451 | -4.008155 | 0.006379424 | 0.076320644 |
| --- | --- | --- | --- | --- | --- | --- | --- |
| 100506791 | NR_125810 | LINC01511 | long intergenic non-protein coding RNA 1511 | -104.022451 | -4.008155 | 0.006379424 | 0.076320644 |
| 101929769 | NR_132101 | LINC01063 | long intergenic non-protein coding RNA 1063 | -104.022451 | -3.208094 | 0.006379424 | 0.076320644 |
| 695 | NM_000061,N | BTK | Bruton tyrosine kinase | -96.097647 | -2.965417 | 0.008564159 | 0.094530216 |
| 2259 | NM_001321931FGF14 | | fibroblast growth factor 14 | -96.097647 | -4.085908 | 0.008564159 | 0.094530216 |
| 3824 | NM_001114396KLRD1 | | killer cell lectin like receptor D1 | -96.097647 | -4.085908 | 0.008564159 | 0.094530216 |
| 5055 | NM_001143818SERPINB2 | | serpin family B member 2 | -96.097647 | -4.085908 | 0.008564159 | 0.094530216 |
| 5653 | NM_001012964KLK6 | | kallikrein related peptidase 6 | -96.097647 | -4.085908 | 0.008564159 | 0.094530216 |
| 5697 | NM_004160 PYY | | peptide YY | -96.097647 | -3.730848 | 0.008564159 | 0.094530216 |
| 8360 | NM_003539 HIST1H4D | | histone cluster 1 H4 family member d | -96.097647 | -3.730848 | 0.008564159 | 0.094530216 |
| 10777 | NM_001025068ARPP21 | | cAMP regulated phosphoprotein 21 | -96.097647 | -4.085908 | 0.008564159 | 0.094530216 |
| 55784 | NM_001159643MCTP2 | | multiple C2 and transmembrane domain containing 2 | -96.097647 | -3.933314 | 0.008564159 | 0.094530216 |
| 63827 | NM_021948,N BCAN | | brevican | -96.097647 | -4.085908 | 0.008564159 | 0.094530216 |
| 81551 | NM_001283053STMN4 | | stathmin 4 | -96.097647 | -3.933314 | 0.008564159 | 0.094530216 |
| 148709 | NR_002929 LOC148709 | | actin pseudogene | -96.097647 | -2.757901 | 0.008564159 | 0.094530216 |
| 149934 | NR_003678 NCOR1P1 | | nuclear receptor corepressor 1 pseudogene 1 | -96.097647 | -4.085908 | 0.008564159 | 0.094530216 |
| 151121 | NR_122040,NR LINC01854 | | long intergenic non-protein coding RNA 1854 | -96.097647 | -3.933314 | 0.008564159 | 0.094530216 |
| 202151 | NM_001161429RANBP3L | | RAN binding protein 3 like | -96.097647 | -4.085908 | 0.008564159 | 0.094530216 |
| 340286 | NR_028347 FAM183BP | | family with sequence similarity 183 member B, pseudogene | -96.097647 | -3.933314 | 0.008564159 | 0.094530216 |
| 345651 | NM_001017992ACTBL2 | | actin, beta like 2 | -96.097647 | -3.669183 | 0.008564159 | 0.094530216 |
| 101060211 | NM_001291543CT45A7 | | cancer/testis antigen family 45 member A7 | -96.097647 | -4.085908 | 0.008564159 | 0.094530216 |
| 101929407 | NR_121599,NR PTPRD-AS1 | | PTPRD antisense RNA 1 | -96.097647 | -4.085908 | 0.008564159 | 0.094530216 |
| 5099 | NM_001173523PCDH7 | | protocadherin 7 | -92.071970 | 0.038741 | 4.13374E-08 | 5.73868E-06 |
| 4312 | NM_001145938MMP1 | | matrix metallopeptidase 1 | -91.670122 | 0.966625 | 8.04088E-09 | 1.459E-06 |
| 1137 | NM_000744,N CHRNA4 | | cholinergic receptor nicotinic alpha 4 subunit | -88.172843 | -4.168113 | 0.011667115 | 0.118301114 |
| 6457 | NM_001301108SH3GL3 | | SH3 domain containing GRB2 like 3, endophilin A3 | -88.172843 | -2.604704 | 0.011667115 | 0.118301114 |
| 9899 | NM_001167580SV2B | | synaptic vesicle glycoprotein 2B | -88.172843 | -4.168113 | 0.011667115 | 0.118301114 |
| 51298 | NM_016585,N THEG | | theg spermatid protein | -88.172843 | -3.932772 | 0.011667115 | 0.118301114 |
| 51438 | NM_016249 MAGEC2 | | MAGE family member C2 | -88.172843 | -3.498002 | 0.011667115 | 0.118301114 |
| 54544 | NM_019060 CRCT1 | | cysteine rich C-terminal 1 | -88.172843 | -4.168113 | 0.011667115 | 0.118301114 |
| 64881 | NM_022843 PCDH20 | | protocadherin 20 | -88.172843 | -3.794720 | 0.011667115 | 0.118301114 |
| 120939 | NM_001079815TMEM52B | | transmembrane protein 52B | -88.172843 | -2.016710 | 0.011667115 | 0.118301114 |
| 121256 | NM_133448 TMEM132D | | transmembrane protein 132D | -88.172843 | -4.168113 | 0.011667115 | 0.118301114 |
| 150622 | NR_026832 LINC01105 | | long intergenic non-protein coding RNA 1105 | -88.172843 | -4.168113 | 0.011667115 | 0.118301114 |
| 162967 | NM_001351773ZNF320 | | zinc finger protein 320 | -88.172843 | -4.007057 | 0.011667115 | 0.118301114 |
| 222553 | NM_001029858SLC35F1 | | solute carrier family 35 member F1 | -88.172843 | -4.007057 | 0.011667115 | 0.118301114 |
| 347404 | NM_001170331LANCL3 | | LanC like 3 | -88.172843 | -3.345309 | 0.011667115 | 0.118301114 |
| 390212 | NM_206997 GPR152 | | G protein-coupled receptor 152 | -88.172843 | -4.168113 | 0.011667115 | 0.118301114 |
| 730755 | NM_001165252KRTAP2-3 | | keratin associated protein 2-3 | -88.172843 | -3.668666 | 0.011667115 | 0.118301114 |
| 100506436 | NR_040012 SPAG5-AS1 | | SPAG5 antisense RNA 1 | -88.172843 | -2.634001 | 0.011667115 | 0.118301114 |
| 100874043 | NR_046873 TPRG1-AS1 | | TPRG1 antisense RNA 1 | -88.172843 | -2.414691 | 0.011667115 | 0.118301114 |
| 100874107 | NR_046378 MTUS2-AS1 | | MTUS2 antisense RNA 1 | -88.172843 | -4.168113 | 0.011667115 | 0.118301114 |
| 101926933 | NR_110002 LOC101926933 | | uncharacterized LOC101926933 | -88.172843 | -4.168113 | 0.011667115 | 0.118301114 |

| 101929467 | NR_125775 | LURAP1L-AS1 | LURAP1L antisense RNA 1 | -88.172843 | -3.794720 | 0.011667115 | 0.118301114 |
| --- | --- | --- | --- | --- | --- | --- | --- |
| 101929690 | NR_120367 | LINC01583 | long intergenic non-protein coding RNA 1583 | -88.172843 | -3.297783 | 0.011667115 | 0.118301114 |
| 105379514 | NR_135513 | LOC105379514 | uncharacterized LOC105379514 | -88.172843 | -4.168113 | 0.011667115 | 0.118301114 |
| 106144537 | NR_132376 | LINC01173 | long intergenic non-protein coding RNA 1173 | -88.172843 | -4.168113 | 0.011667115 | 0.118301114 |
| 6364 | NM_001130046CCL20 | | C-C motif chemokine ligand 20 | -88.030266 | 3.841950 | 7.68539E-09 | 1.42557E-06 |
| 9358 | NM_001271754ITGBL1 | | integrin subunit beta like 1 | -87.828156 | 0.373790 | 2.55352E-08 | 3.9819E-06 |
| 117 | NM_001118,N ADCYAP1R1 | | ADCYAP receptor type I | -80.248039 | -3.609010 | 0.016154467 | 0.146302603 |
| 1295 | NM_001850,N COL8A1 | | collagen type VIII alpha 1 chain | -80.248039 | -3.794190 | 0.016154467 | 0.146302603 |
| 1555 | NM_000767 CYP2B6 | | cytochrome P450 family 2 subfamily B member 6 | -80.248039 | -4.255312 | 0.016154467 | 0.146302603 |
| 2914 | NM_000841,N GRM4 | | glutamate metabotropic receptor 4 | -80.248039 | -3.668149 | 0.016154467 | 0.146302603 |
| 3670 | NM_002202 ISL1 | | ISL LIM homeobox 1 | -80.248039 | -3.497503 | 0.016154467 | 0.146302603 |
| 4741 | NM_001105541NEFM | | neurofilament medium | -80.248039 | -3.163423 | 0.016154467 | 0.146302603 |
| 5408 | NM_005396,NRPNLIPRP2 | | pancreatic lipase related protein 2 (gene/pseudogene) | -80.248039 | -4.255312 | 0.016154467 | 0.146302603 |
| 6263 | NM_001036,N RYR3 | | ryanodine receptor 3 | -80.248039 | -4.255312 | 0.016154467 | 0.146302603 |
| 6456 | NM_003026 SH3GL2 | | SH3 domain containing GRB2 like 2, endophilin A1 | -80.248039 | -3.668149 | 0.016154467 | 0.146302603 |
| 6578 | NM_005630 SLCO2A1 | | solute carrier organic anion transporter family member 2A1 | -80.248039 | -4.255312 | 0.016154467 | 0.146302603 |
| 6698 | NM_001199828SPRR1A | | small proline rich protein 1A | -80.248039 | -4.255312 | 0.016154467 | 0.146302603 |
| 7757 | NM_001329971ZNF208 | | zinc finger protein 208 | -80.248039 | -4.255312 | 0.016154467 | 0.146302603 |
| 8359 | NM_003538 HIST1H4A | | histone cluster 1 H4 family member a | -80.248039 | -3.668149 | 0.016154467 | 0.146302603 |
| 9332 | NM_004244,N CD163 | | CD163 molecule | -80.248039 | -4.084795 | 0.016154467 | 0.146302603 |
| 10003 | NM_001300930NAALAD2 | | N-acetylated alpha-linked acidic dipeptidase 2 | -80.248039 | -4.255312 | 0.016154467 | 0.146302603 |
| 29906 | NM_001307986ST8SIA5 | | ST8 alpha-N-acetyl-neuraminide alpha-2,8-sialyltransferase | -80.248039 | -4.006508 | 0.016154467 | 0.146302603 |
| 54857 | NM_001171191GDPD2 | | glycerophosphodiester phosphodiesterase domain containi | -80.248039 | -3.344827 | 0.016154467 | 0.146302603 |
| 56253 | NM_001304782CRTAM | | cytotoxic and regulatory T-cell molecule | -80.248039 | -3.932229 | 0.016154467 | 0.146302603 |
| 57569 | NM_001258415ARHGAP20 | | Rho GTPase activating protein 20 | -80.248039 | -2.725207 | 0.016154467 | 0.146302603 |
| 79927 | NM_024869 FAM110D | | family with sequence similarity 110 member D | -80.248039 | -4.255312 | 0.016154467 | 0.146302603 |
| 83482 | NM_031309 SCRT1 | | scratch family transcriptional repressor 1 | -80.248039 | -4.255312 | 0.016154467 | 0.146302603 |
| 123745 | NM_001206670PLA2G4E | | phospholipase A2 group IVE | -80.248039 | -3.861569 | 0.016154467 | 0.146302603 |
| 135644 | NM_001286633TRIM40 | | tripartite motif containing 40 | -80.248039 | -4.084795 | 0.016154467 | 0.146302603 |
| 147710 | NM_001205280IGSF23 | | immunoglobulin superfamily member 23 | -80.248039 | -4.084795 | 0.016154467 | 0.146302603 |
| 257358 | NR_026945,NR LINC01366 | | long intergenic non-protein coding RNA 1366 | -80.248039 | -3.393956 | 0.016154467 | 0.146302603 |
| 389458 | NR_015343 RBAKDN | | RBAK downstream neighbor (non-protein coding) | -80.248039 | -3.668149 | 0.016154467 | 0.146302603 |
| 399947 | NM_207645 C11orf87 | | chromosome 11 open reading frame 87 | -80.248039 | -3.497503 | 0.016154467 | 0.146302603 |
| 494197 | NM_001009616SPANXN5 | | SPANX family member N5 | -80.248039 | -4.255312 | 0.016154467 | 0.146302603 |
| 643160 | NR_003599 CYMP | | chymosin pseudogene | -80.248039 | -4.255312 | 0.016154467 | 0.146302603 |
| 728597 | NM_001287444DCDC2C | | doublecortin domain containing 2C | -80.248039 | -4.255312 | 0.016154467 | 0.146302603 |
| 728712 | NM_145662,N SPANXA2 | | SPANX family member A2 | -80.248039 | -4.006508 | 0.016154467 | 0.146302603 |
| 100287639 | NR_077228 MTHFD2P1 | | methylenetetrahydrofolate dehydrogenase (NADP+ depend | -80.248039 | -4.084795 | 0.016154467 | 0.146302603 |
| 104326193 | NR_126376 DLX2-AS1 | | DLX2 antisense RNA 1 (head to head) | -80.248039 | -3.794190 | 0.016154467 | 0.146302603 |
| 80760 | NM_001001851ITIH5 | | inter-alpha-trypsin inhibitor heavy chain family member 5 | -75.857439 | 0.425583 | 3.70633E-08 | 5.32398E-06 |
| 118611 | NM_001004298C10orf90 | | chromosome 10 open reading frame 90 | -75.624039 | 1.125362 | 1.52583E-08 | 2.63015E-06 |
| 7425 | NM_003378 VGF | | VGF nerve growth factor inducible | -74.050026 | 3.824626 | 3.46948E-09 | 7.46717E-07 |
| 1818 | NR_111001 DRD5P2 | | dopamine receptor D5 pseudogene 2 | -72.323235 | -4.348151 | 0.02277515 | 0.184819135 |

| 3662 | NM_001195286IRF4 | interferon regulatory factor 4 | -72.323235 | -4.005958 | 0.02277515 | 0.184819135 |
| --- | --- | --- | --- | --- | --- | --- |
| 3753 | NM_000219,N KCNE1 | potassium voltage-gated channel subfamily E regulatory su | -72.323235 | -4.348151 | 0.02277515 | 0.184819135 |
| 4321 | NM_002426 MMP12 | matrix metallopeptidase 12 | -72.323235 | -3.344344 | 0.02277515 | 0.184819135 |
| 5047 | NM_001018048PAEP | progestagen associated endometrial protein | -72.323235 | -3.793661 | 0.02277515 | 0.184819135 |
| 6123 | NM_005061 RPL3L | ribosomal protein L3 like | -72.323235 | -4.348151 | 0.02277515 | 0.184819135 |
| 6707 | NM_001097589SPRR3 | small proline rich protein 3 | -72.323235 | -4.348151 | 0.02277515 | 0.184819135 |
| 7730 | NM_001172651ZNF177 | zinc finger protein 177 | -72.323235 | -1.542206 | 0.02277515 | 0.184819135 |
| 8128 | NM_001330416ST8SIA2 | ST8 alpha-N-acetyl-neuraminide alpha-2,8-sialyltransferase | -72.323235 | -4.084239 | 0.02277515 | 0.184819135 |
| 8424 | NM_003986 BBOX1 | gamma-butyrobetaine hydroxylase 1 | -72.323235 | -4.348151 | 0.02277515 | 0.184819135 |
| 8647 | NM_003742 ABCB11 | ATP binding cassette subfamily B member 11 | -72.323235 | -3.608499 | 0.02277515 | 0.184819135 |
| 55170 | NM_018137 PRMT6 | protein arginine methyltransferase 6 | -72.323235 | -4.348151 | 0.02277515 | 0.184819135 |
| 56287 | NM_019617 GKN1 | gastrokine 1 | -72.323235 | -4.348151 | 0.02277515 | 0.184819135 |
| 57111 | NM_020387,NRRAB25 | RAB25, member RAS oncogene family | -72.323235 | -4.084239 | 0.02277515 | 0.184819135 |
| 79750 | NM_024697 ZNF385D | zinc finger protein 385D | -72.323235 | -4.348151 | 0.02277515 | 0.184819135 |
| 84109 | NM_198179 QRFPR | pyroglutamylated RFamide peptide receptor | -72.323235 | -4.005958 | 0.02277515 | 0.184819135 |
| 84891 | NM_001282415ZSCAN10 | zinc finger and SCAN domain containing 10 | -72.323235 | -4.166986 | 0.02277515 | 0.184819135 |
| 115749 | NM_001099676C12orf56 | chromosome 12 open reading frame 56 | -72.323235 | -3.393468 | 0.02277515 | 0.184819135 |
| 117195 | NM_054031 MRGPRX3 | MAS related GPR family member X3 | -72.323235 | -4.005958 | 0.02277515 | 0.184819135 |
| 145241 | NR_003951 ADAM21P1 | ADAM metallopeptidase domain 21 pseudogene 1 | -72.323235 | -4.348151 | 0.02277515 | 0.184819135 |
| 375057 | NM_001003665STUM | stum, mechanosensory transduction mediator homolog | -72.323235 | -4.084239 | 0.02277515 | 0.184819135 |
| 390940 | NM_001193621PINLYP | phospholipase A2 inhibitor and LY6/PLAUR domain contain | -72.323235 | -3.497004 | 0.02277515 | 0.184819135 |
| 644919 | NR_109757,NR LINC02315 | long intergenic non-protein coding RNA 2315 | -72.323235 | -4.348151 | 0.02277515 | 0.184819135 |
| 692148 | NR_004387 SCARNA10 | small Cajal body-specific RNA 10 | -72.323235 | -3.040406 | 0.02277515 | 0.184819135 |
| 729786 | NR_027411 GOLGA8CP | golgin A8 family member C, pseudogene | -72.323235 | -4.348151 | 0.02277515 | 0.184819135 |
| 729987 | NR_046088 LINC01776 | long intergenic non-protein coding RNA 1776 | -72.323235 | -3.729279 | 0.02277515 | 0.184819135 |
| 100130086 | NM_001164415HSFX2 | heat shock transcription factor family, X-linked 2 | -72.323235 | -2.519437 | 0.02277515 | 0.184819135 |
| 100506564 | NM_001256475THEGL | theg spermatid protein like | -72.323235 | -3.344344 | 0.02277515 | 0.184819135 |
| 5266 | NM_002638 PI3 | peptidase inhibitor 3 | -67.048523 | 0.562069 | 7.66975E-08 | 9.79314E-06 |
| 495 | NM_000704 ATP4A | ATPase H+/K+ transporting alpha subunit | -64.398431 | -4.447408 | 0.032764251 | 0.233618934 |
| 814 | NM_001323374CAMK4 | calcium/calmodulin dependent protein kinase IV | -64.398431 | -3.343861 | 0.032764251 | 0.233618934 |
| 869 | NM_004352 CBLN1 | cerebellin 1 precursor | -64.398431 | -4.083682 | 0.032764251 | 0.233618934 |
| 1521 | NM_001335 CTSW | cathepsin W | -64.398431 | -4.447408 | 0.032764251 | 0.233618934 |
| 1750 | NM_005222 DLX6 | distal-less homeobox 6 | -64.398431 | -4.005409 | 0.032764251 | 0.233618934 |
| 2042 | NM_005233,N EPHA3 | EPH receptor A3 | -64.398431 | -4.447408 | 0.032764251 | 0.233618934 |
| 2167 | NM_001442 FABP4 | fatty acid binding protein 4 | -64.398431 | -4.447408 | 0.032764251 | 0.233618934 |
| 2253 | NM_001206389FGF8 | fibroblast growth factor 8 | -64.398431 | -4.447408 | 0.032764251 | 0.233618934 |
| 2357 | NM_001193306FPR1 | formyl peptide receptor 1 | -64.398431 | -4.447408 | 0.032764251 | 0.233618934 |
| 2780 | NM_005272 GNAT2 | G protein subunit alpha transducin 2 | -64.398431 | -3.667115 | 0.032764251 | 0.233618934 |
| 3018 | NM_021062 HIST1H2BB | histone cluster 1 H2B family member b | -64.398431 | -4.005409 | 0.032764251 | 0.233618934 |
| 3815 | NM_000222,N KIT | KIT proto-oncogene receptor tyrosine kinase | -64.398431 | -4.447408 | 0.032764251 | 0.233618934 |
| 4210 | NM_000243,N MEFV | MEFV, pyrin innate immunity regulator | -64.398431 | -3.496505 | 0.032764251 | 0.233618934 |
| 5454 | NM_005604 POU3F2 | POU class 3 homeobox 2 | -64.398431 | -4.447408 | 0.032764251 | 0.233618934 |
| 6332 | NM_002976,NRSCN7A | sodium voltage-gated channel alpha subunit 7 | -64.398431 | -4.447408 | 0.032764251 | 0.233618934 |

| 6406 | NM_003007 | SEMG1 | semenogelin I | -64.398431 | -4.447408 | 0.032764251 | 0.233618934 |
| --- | --- | --- | --- | --- | --- | --- | --- |
| 7754 | NR_002722,NR | ZNF204P | zinc finger protein 204, pseudogene | -64.398431 | -4.447408 | 0.032764251 | 0.233618934 |
| 10324 | NM_006063 | KLHL41 | kelch like family member 41 | -64.398431 | -3.607988 | 0.032764251 | 0.233618934 |
| 10633 | NM_006477 | RASL10A | RAS like family 10 member A | -64.398431 | -4.447408 | 0.032764251 | 0.233618934 |
| 10655 | NM_001130865DMRT2 | | doublesex and mab-3 related transcription factor 2 | -64.398431 | -3.793131 | 0.032764251 | 0.233618934 |
| 51513 | NM_001207035ETV7 | | ETS variant 7 | -64.398431 | -3.667115 | 0.032764251 | 0.233618934 |
| 124590 | NM_001282489USH1G | | USH1 protein network component sans | -64.398431 | -3.551176 | 0.032764251 | 0.233618934 |
| 134829 | NM_001010852CLVS2 | | clavesin 2 | -64.398431 | -4.447408 | 0.032764251 | 0.233618934 |
| 148979 | NM_147193 GLIS1 | | GLIS family zinc finger 1 | -64.398431 | -4.447408 | 0.032764251 | 0.233618934 |
| 163115 | NM_152605 ZNF781 | | zinc finger protein 781 | -64.398431 | -4.447408 | 0.032764251 | 0.233618934 |
| 168090 | NM_144980 C6orf118 | | chromosome 6 open reading frame 118 | -64.398431 | -3.001309 | 0.032764251 | 0.233618934 |
| 204219 | NM_001290341CERS3 | | ceramide synthase 3 | -64.398431 | -4.447408 | 0.032764251 | 0.233618934 |
| 221692 | NM_001242648PHACTR1 | | phosphatase and actin regulator 1 | -64.398431 | -4.447408 | 0.032764251 | 0.233618934 |
| 257313 | NM_198152 UTS2B | | urotensin 2B | -64.398431 | -4.447408 | 0.032764251 | 0.233618934 |
| 286530 | NM_178129_ch P2RY8 | | purinergic receptor P2Y8 | -64.398431 | -4.447408 | 0.032764251 | 0.233618934 |
| 339685 | NR_144462 LOC339685 | | uncharacterized LOC339685 | -64.398431 | -3.607988 | 0.032764251 | 0.233618934 |
| 339751 | NR_033882 MAP3K20-AS1 | | MAP3K20 antisense RNA 1 | -64.398431 | -4.166422 | 0.032764251 | 0.233618934 |
| 387758 | NM_203371 FIBIN | | fin bud initiation factor homolog (zebrafish) | -64.398431 | -4.447408 | 0.032764251 | 0.233618934 |
| 100129520 | NM_001195272TEX13C | | TEX13 family member C | -64.398431 | -3.793131 | 0.032764251 | 0.233618934 |
| 100132979 | NR_027407 GOLGA8DP | | golgin A8 family member D, pseudogene | -64.398431 | -4.447408 | 0.032764251 | 0.233618934 |
| 100190940 | NR_024457 LINC02418 | | long intergenic non-protein coding RNA 2418 | -64.398431 | -4.447408 | 0.032764251 | 0.233618934 |
| 100500918 | NR_037424 MIR3651 | | microRNA 3651 | -64.398431 | -4.347572 | 0.032764251 | 0.233618934 |
| 100505785 | NR_145471 KIRREL-IT1 | | KIRREL intronic transcript 1 | -64.398431 | -3.860497 | 0.032764251 | 0.233618934 |
| 100507392 | NR_038908 SENCR | | smooth muscle and endothelial cell enriched migration/diff | -64.398431 | -4.447408 | 0.032764251 | 0.233618934 |
| 100528018 | NR_037650 ARL2-SNX15 | | ARL2-SNX15 readthrough (NMD candidate) | -64.398431 | -4.254170 | 0.032764251 | 0.233618934 |
| 101927124 | NR_110045 LOC101927124 | | uncharacterized LOC101927124 | -64.398431 | -4.347572 | 0.032764251 | 0.233618934 |
| 105375475 | NR_131960,NR LINC02476 | | long intergenic non-protein coding RNA 2476 | -64.398431 | -4.447408 | 0.032764251 | 0.233618934 |
| 105377623 | NR_136203 LOC105377623 | | uncharacterized LOC105377623 | -64.398431 | -3.728756 | 0.032764251 | 0.233618934 |
| 109729128 | NR_146275 LINC01930 | | long intergenic non-protein coding RNA 1930 | -64.398431 | -4.447408 | 0.032764251 | 0.233618934 |
| 431707 | NM_001001933LHX8 | | LIM homeobox 8 | -64.302165 | 1.709130 | 1.72875E-08 | 2.86073E-06 |
| 80161 | NR_026710_chr ASMTL-AS1 | | ASMTL antisense RNA 1 | -63.558754 | 0.387134 | 1.74108E-07 | 1.86579E-05 |
| 3624 | NM_002192 INHBA | | inhibin beta A subunit | -63.410058 | 0.193105 | 1.07155E-07 | 1.26545E-05 |
| 54577 | NM_019077 UGT1A7 | | UDP glucuronosyltransferase family 1 member A7 | -63.253433 | 4.922119 | 6.8384E-09 | 1.30974E-06 |
| 100885850 | NM_001136042PTGES3L-AARSD1 | | PTGES3L-AARSD1 readthrough | -61.966231 | -0.062718 | 1.3502E-06 | 0.000101036 |
| 5455 | NM_006236 POU3F3 | | POU class 3 homeobox 3 | -59.656234 | -1.106590 | 1.68013E-06 | 0.00011741 |
| 3575 | NM_002185,NRIL7R | | interleukin 7 receptor | -59.194234 | -1.055973 | 1.75686E-06 | 0.000121948 |
| 57574 | NM_020814 MARCH4 | | membrane associated ring-CH-type finger 4 | -58.149065 | 5.717665 | 1.07489E-08 | 1.90034E-06 |
| 4923 | NM_002531 NTSR1 | | neurotensin receptor 1 | -57.134500 | 0.112741 | 3.2739E-07 | 3.05048E-05 |
| 881 | NM_005893 CCIN | | calicin | -56.473627 | -4.554038 | 0.048219086 | 0.298715722 |
| 1012 | NM_001220488CDH13 | | cadherin 13 | -56.473627 | -4.554038 | 0.048219086 | 0.298715722 |
| 1240 | NM_001142343CMKLR1 | | chemerin chemokine-like receptor 1 | -56.473627 | -4.554038 | 0.048219086 | 0.298715722 |
| 2254 | NM_002010 FGF9 | | fibroblast growth factor 9 | -56.473627 | -4.554038 | 0.048219086 | 0.298715722 |
| 2328 | NM_001002294FMO3 | | flavin containing monooxygenase 3 | -56.473627 | -4.554038 | 0.048219086 | 0.298715722 |

| 2813 | NM_001007240GP2 | glycoprotein 2 | -56.473627 | -4.554038 | 0.048219086 | 0.298715722 |
| --- | --- | --- | --- | --- | --- | --- |
| 2982 | NM_000856,N GUCY1A3 | guanylate cyclase 1 soluble subunit alpha | -56.473627 | -4.346993 | 0.048219086 | 0.298715722 |
| 3040 | NM_000517 HBA2 | hemoglobin subunit alpha 2 | -56.473627 | -4.554038 | 0.048219086 | 0.298715722 |
| 3135 | NM_002127 HLA-G | major histocompatibility complex, class I, G | -56.473627 | -4.554038 | 0.048219086 | 0.298715722 |
| 4915 | NM_001007097NTRK2 | neurotrophic receptor tyrosine kinase 2 | -56.473627 | -3.859961 | 0.048219086 | 0.298715722 |
| 5320 | NM_000300,N PLA2G2A | phospholipase A2 group IIA | -56.473627 | -4.554038 | 0.048219086 | 0.298715722 |
| 6005 | NM_000324 RHAG | Rh-associated glycoprotein | -56.473627 | -4.554038 | 0.048219086 | 0.298715722 |
| 6354 | NM_006273 CCL7 | C-C motif chemokine ligand 7 | -56.473627 | -4.083125 | 0.048219086 | 0.298715722 |
| 7356 | NM_003357 SCGB1A1 | secretoglobin family 1A member 1 | -56.473627 | -4.554038 | 0.048219086 | 0.298715722 |
| 7447 | NM_003385 VSNL1 | visinin like 1 | -56.473627 | -4.554038 | 0.048219086 | 0.298715722 |
| 8342 | NM_003521 HIST1H2BM | histone cluster 1 H2B family member m | -56.473627 | -3.728232 | 0.048219086 | 0.298715722 |
| 8346 | NM_003525 HIST1H2BI | histone cluster 1 H2B family member i | -56.473627 | -4.253599 | 0.048219086 | 0.298715722 |
| 9955 | NM_006042 HS3ST3A1 | heparan sulfate-glucosamine 3-sulfotransferase 3A1 | -56.473627 | -4.554038 | 0.048219086 | 0.298715722 |
| 11026 | NM_001172654LILRA3 | leukocyte immunoglobulin like receptor A3 | -56.473627 | -4.554038 | 0.048219086 | 0.298715722 |
| 26290 | NM_017417 GALNT8 | polypeptide N-acetylgalactosaminyltransferase 8 | -56.473627 | -4.554038 | 0.048219086 | 0.298715722 |
| 27290 | NM_014471 SPINK4 | serine peptidase inhibitor, Kazal type 4 | -56.473627 | -4.554038 | 0.048219086 | 0.298715722 |
| 51294 | NM_016580 PCDH12 | protocadherin 12 | -56.473627 | -3.079214 | 0.048219086 | 0.298715722 |
| 58494 | NM_001270407JAM2 | junctional adhesion molecule 2 | -56.473627 | -4.554038 | 0.048219086 | 0.298715722 |
| 63876 | NM_022062 PKNOX2 | PBX/knotted 1 homeobox 2 | -56.473627 | -4.554038 | 0.048219086 | 0.298715722 |
| 63923 | NM_022093 TNN | tenascin N | -56.473627 | -4.554038 | 0.048219086 | 0.298715722 |
| 126206 | NM_153447 NLRP5 | NLR family pyrin domain containing 5 | -56.473627 | -4.554038 | 0.048219086 | 0.298715722 |
| 151475 | NR_040038 LINC01907 | long intergenic non-protein coding RNA 1907 | -56.473627 | -4.554038 | 0.048219086 | 0.298715722 |
| 284266 | NM_213602 SIGLEC15 | sialic acid binding Ig like lectin 15 | -56.473627 | -4.554038 | 0.048219086 | 0.298715722 |
| 285782 | NM_001170692CAGE1 | cancer antigen 1 | -56.473627 | -3.120034 | 0.048219086 | 0.298715722 |
| 340581 | NR_134925 LINC02243 | long intergenic non-protein coding RNA 2243 | -56.473627 | -4.554038 | 0.048219086 | 0.298715722 |
| 348645 | NM_001289922C22orf34 | chromosome 22 open reading frame 34 | -56.473627 | -4.554038 | 0.048219086 | 0.298715722 |
| 374907 | NM_198540 B3GNT8 | UDP-GlcNAc:betaGal beta-1,3-N-acetylglucosaminyltransfer | -56.473627 | -3.792602 | 0.048219086 | 0.298715722 |
| 401138 | NM_001286731AMTN | amelotin | -56.473627 | -4.554038 | 0.048219086 | 0.298715722 |
| 415117 | NM_001001850STX19 | syntaxin 19 | -56.473627 | -3.205292 | 0.048219086 | 0.298715722 |
| 647309 | NM_001146686GMNC | geminin coiled-coil domain containing | -56.473627 | -3.930601 | 0.048219086 | 0.298715722 |
| 654790 | NM_001102566PCP4L1 | Purkinje cell protein 4 like 1 | -56.473627 | -4.554038 | 0.048219086 | 0.298715722 |
| 727982 | NR_034134 LINC01249 | long intergenic non-protein coding RNA 1249 | -56.473627 | -4.554038 | 0.048219086 | 0.298715722 |
| 729420 | NM_001257995LMO7DN | LMO7 downstream neighbor | -56.473627 | -4.554038 | 0.048219086 | 0.298715722 |
| 100131094 | NM_001242901DPP9-AS1 | DPP9 antisense RNA 1 | -56.473627 | -4.554038 | 0.048219086 | 0.298715722 |
| 100506068 | NR_040041,NR DACT3-AS1 | DACT3 antisense RNA 1 | -56.473627 | -4.554038 | 0.048219086 | 0.298715722 |
| 100873979 | NR_109804 RBMS3-AS3 | RBMS3 antisense RNA 3 | -56.473627 | -4.165858 | 0.048219086 | 0.298715722 |
| 101927780 | NR_110416,NR LOC101927780 | uncharacterized LOC101927780 | -56.473627 | -3.343379 | 0.048219086 | 0.298715722 |
| 101929025 | NR_120649 LINC01516 | long intergenic non-protein coding RNA 1516 | -56.473627 | -4.554038 | 0.048219086 | 0.298715722 |
| 101929159 | NR_135550 LOC101929159 | uncharacterized LOC101929159 | -56.473627 | -4.253599 | 0.048219086 | 0.298715722 |
| 105369980 | NR_135001,NR LOC105369980 | uncharacterized LOC105369980 | -56.473627 | -4.554038 | 0.048219086 | 0.298715722 |
| 59344 | NM_001165960ALOXE3 | arachidonate lipoxygenase 3 | -56.143537 | 2.450701 | 2.66313E-08 | 4.11096E-06 |
| 5243 | NM_000927,N ABCB1 | ATP binding cassette subfamily B member 1 | -55.950064 | -0.601462 | 7.78193E-07 | 6.33737E-05 |
| 597 | NM_001114735BCL2A1 | BCL2 related protein A1 | -55.080684 | 2.621744 | 4.12706E-08 | 5.73868E-06 |

| 1066 | NM_001025194CES1 | carboxylesterase 1 | -54.054158 | 5.303112 | 1.70839E-08 | 2.84985E-06 |
| --- | --- | --- | --- | --- | --- | --- |
| 137970 | NM_001322818UNC5D | unc-5 netrin receptor D | -52.950789 | 1.527299 | 5.20489E-08 | 7.03681E-06 |
| 4747 | NM_006158 NEFL | neurofilament light | -51.051370 | 4.572295 | 2.56028E-08 | 3.9819E-06 |
| 11199 | NM_007193 ANXA10 | annexin A10 | -49.802161 | 3.241777 | 3.86999E-08 | 5.48293E-06 |
| 8638 | NM_001261825OASL | 2'-5'-oligoadenylate synthetase like | -49.512497 | 4.201768 | 3.19766E-08 | 4.72454E-06 |
| 8710 | NM_001040147SERPINB7 | serpin family B member 7 | -48.650169 | 1.779148 | 1.19663E-07 | 1.39844E-05 |
| 7130 | NM_007115 TNFAIP6 | TNF alpha induced protein 6 | -42.961083 | 0.003879 | 7.39937E-07 | 6.10654E-05 |
| 1305 | NM_001130103COL13A1 | collagen type XIII alpha 1 chain | -42.443612 | -0.215639 | 3.69954E-06 | 0.000225599 |
| 3310 | NM_002155 HSPA6 | heat shock protein family A (Hsp70) member 6 | -40.851085 | 1.045035 | 3.72971E-07 | 3.38373E-05 |
| 6098 | NM_002944 ROS1 | ROS proto-oncogene 1, receptor tyrosine kinase | -40.233272 | 0.758813 | 3.68758E-07 | 3.36025E-05 |
| 28999 | NM_014079 KLF15 | Kruppel like factor 15 | -38.866256 | -1.741538 | 1.87643E-05 | 0.000860622 |
| 10202 | NM_001318835DHRS2 | dehydrogenase/reductase 2 | -35.316035 | 2.803733 | 2.9135E-07 | 2.75572E-05 |
| 2793 | NM_001198754GNGT2 | G protein subunit gamma transducin 2 | -35.170260 | -1.872197 | 3.24104E-05 | 0.00136029 |
| 147372 | NM_133459 CCBE1 | collagen and calcium binding EGF domains 1 | -34.935202 | 1.803539 | 4.15685E-07 | 3.70622E-05 |
| 11151 | NM_001193333CORO1A | coronin 1A | -34.104655 | 1.077128 | 1.41919E-06 | 0.000104843 |
| 7078 | NM_000362 TIMP3 | TIMP metallopeptidase inhibitor 3 | -31.421039 | -0.302990 | 3.36413E-06 | 0.00021053 |
| 100874309 | NR_046824 EPN2-IT1 | EPN2 intronic transcript 1 | -28.922445 | -2.004640 | 0.000600631 | 0.013231154 |
| 101927551 | NR_147506 LOC101927551 | uncharacterized LOC101927551 | -27.176247 | -2.187241 | 0.000804374 | 0.016655139 |
| 101928687 | NR_120641,NR LINC01468 | long intergenic non-protein coding RNA 1468 | -26.774498 | 3.421654 | 1.25392E-06 | 9.50085E-05 |
| 2983 | NM_000857,N GUCY1B3 | guanylate cyclase 1 soluble subunit beta | -25.816782 | -0.208196 | 1.3137E-05 | 0.000637887 |
| 6620 | NM_001001502SNCB | synuclein beta | -25.468271 | -1.554566 | 0.000163744 | 0.004859452 |
| 26585 | NM_001191322GREM1 | gremlin 1, DAN family BMP antagonist | -25.214372 | 5.055809 | 1.56387E-06 | 0.000111933 |
| 6280 | NM_002965 S100A9 | S100 calcium binding protein A9 | -24.392107 | 0.878570 | 3.91759E-06 | 0.00023764 |
| 50509 | NM_015719 COL5A3 | collagen type V alpha 3 chain | -24.378245 | 1.448487 | 3.00793E-06 | 0.000191443 |
| 6695 | NM_004598 SPOCK1 | SPARC/osteonectin, cwcv and kazal like domains proteoglyc | -23.860881 | 4.081173 | 2.27141E-06 | 0.000149631 |
| 7503 | NR_001564 XIST | X inactive specific transcript (non-protein coding) | -23.823556 | -1.332929 | 4.61944E-05 | 0.001809717 |
| 65055 | NM_001164730REEP1 | receptor accessory protein 1 | -23.611335 | 0.892605 | 5.33393E-06 | 0.00030563 |
| 7092 | NM_001204760TLL1 | tolloid like 1 | -23.485621 | 0.239820 | 7.55433E-06 | 0.000412299 |
| 101928841 | NM_001304433LOC101928841 | collagen alpha-1(II) chain-like | -22.837271 | 1.635412 | 4.5359E-06 | 0.00026884 |
| 1794 | NM_004946 DOCK2 | dedicator of cytokinesis 2 | -22.706736 | 1.141351 | 5.51832E-06 | 0.000315322 |
| 5924 | NM_006909 RASGRF2 | Ras protein specific guanine nucleotide releasing factor 2 | -21.879565 | 1.602789 | 6.01661E-06 | 0.000340969 |
| 1306 | NM_001855 COL15A1 | collagen type XV alpha 1 chain | -21.682138 | -1.379533 | 7.67798E-05 | 0.002625106 |
| 221833 | NM_182700,N SP8 | Sp8 transcription factor | -21.682138 | -1.607137 | 7.67798E-05 | 0.002625106 |
| 131578 | NM_001135057LRRC15 | leucine rich repeat containing 15 | -21.398675 | -1.398765 | 0.000157876 | 0.004692055 |
| 2045 | NM_001288629EPHA7 | EPH receptor A7 | -21.275400 | 1.006133 | 7.23995E-06 | 0.000397237 |
| 27012 | NM_014379 KCNV1 | potassium voltage-gated channel modifier subfamily V mem | -21.242673 | 0.172534 | 1.3379E-05 | 0.000645286 |
| 57007 | NM_020311 ACKR3 | atypical chemokine receptor 3 | -20.871337 | 1.408242 | 6.97751E-06 | 0.000385943 |
| 338442 | NM_177551 HCAR2 | hydroxycarboxylic acid receptor 2 | -20.770468 | -1.398272 | 0.000184391 | 0.005297386 |
| 81792 | NM_001324511ADAMTS12 | ADAM metallopeptidase with thrombospondin type 1 motif | -20.517147 | 2.598245 | 6.40724E-06 | 0.000357234 |
| 3885 | NM_021013 KRT34 | keratin 34 | -20.386276 | -2.191811 | 0.000496089 | 0.011332395 |
| 1672 | NM_005218 DEFB1 | defensin beta 1 | -20.016591 | -1.548453 | 0.000117521 | 0.003745634 |
| 56154 | NM_001350162TEX15 | testis expressed 15, meiosis and synapsis associated | -19.390756 | 1.672174 | 1.0642E-05 | 0.000538213 |
| 54742 | NM_001160354LY6K | lymphocyte antigen 6 family member K | -19.358131 | 1.035463 | 1.23032E-05 | 0.000605935 |

| 4922 | NM_006183 | NTS | neurotensin | -19.009888 | 5.263185 | 7.77377E-06 | 0.000417663 |
| --- | --- | --- | --- | --- | --- | --- | --- |
| 101927884 | NR_110281 | LOC101927884 | uncharacterized LOC101927884 | -19.000278 | -2.169218 | 0.000696625 | 0.014824777 |
| 84443 | NM_032428 | FRMPD3 | FERM and PDZ domain containing 3 | -18.885847 | -0.465023 | 0.000301013 | 0.007686975 |
| 100533179 | NR_037904 | UBE2F-SCLY | UBE2F-SCLY readthrough (NMD candidate) | -18.750757 | 1.311760 | 2.17751E-05 | 0.000985597 |
| 9308 | NM_001040280CD83 | | CD83 molecule | -18.666451 | 1.761601 | 1.37763E-05 | 0.000659634 |
| 387763 | NM_001145033C11orf96 | | chromosome 11 open reading frame 96 | -18.599487 | -1.030097 | 0.000110866 | 0.003555439 |
| 3311 | NR_024151 HSPA7 | | heat shock protein family A (Hsp70) member 7 | -18.571744 | -1.867179 | 0.000327888 | 0.008231018 |
| 93659 | NM_033043 CGB5 | | chorionic gonadotropin beta subunit 5 | -18.446335 | -1.166709 | 8.32268E-05 | 0.002808396 |
| 6241 | NM_001034,N RRM2 | | ribonucleotide reductase regulatory subunit M2 | -18.387816 | 7.744529 | 9.09812E-06 | 0.000471666 |
| 81569 | NM_030812 ACTL8 | | actin like 8 | -18.351044 | -1.116160 | 0.000185523 | 0.005322524 |
| 102723471 | NR_135668,NR LOC102723471 | | uncharacterized LOC102723471 | -17.865684 | 0.712286 | 5.45857E-05 | 0.002027118 |
| 3918 | NM_005562,N LAMC2 | | laminin subunit gamma 2 | -17.755853 | 7.924108 | 1.1092E-05 | 0.000556889 |
| 2998 | NM_021957 GYS2 | | glycogen synthase 2 | -17.629433 | -1.831965 | 0.000426839 | 0.010113593 |
| 105371828 | NR_146897 LINC02073 | | long intergenic non-protein coding RNA 2073 | -17.528461 | -0.856487 | 6.92105E-05 | 0.002430804 |
| 10395 | NM_001164271DLC1 | | DLC1 Rho GTPase activating protein | -17.447158 | 2.702336 | 1.46775E-05 | 0.000694747 |
| 92949 | NM_001040272ADAMTSL1 | | ADAMTS like 1 | -17.152280 | -2.768540 | 0.00112921 | 0.021547706 |
| 9201 | NM_001195415DCLK1 | | doublecortin like kinase 1 | -17.137162 | 2.813989 | 1.68261E-05 | 0.000783892 |
| 3569 | NM_000600,N IL6 | | interleukin 6 | -17.098728 | 1.103858 | 4.0746E-05 | 0.001637714 |
| 43 | NM_000665,N ACHE | | acetylcholinesterase (Cartwright blood group) | -16.681363 | 0.724808 | 3.24865E-05 | 0.00136029 |
| 4139 | NM_001286124MARK1 | | microtubule affinity regulating kinase 1 | -16.630204 | 0.370156 | 3.65225E-05 | 0.001487141 |
| 64388 | NM_022469 GREM2 | | gremlin 2, DAN family BMP antagonist | -16.567039 | 0.159052 | 4.10784E-05 | 0.001637714 |
| 1746 | NM_004405 DLX2 | | distal-less homeobox 2 | -16.560007 | 0.244031 | 4.35528E-05 | 0.001722542 |
| 100616394 | NR_039646_chr MIR4444-1 | | microRNA 4444-1 | -16.438299 | 0.721535 | 4.53687E-05 | 0.001784128 |
| 79822 | NM_001010000ARHGAP28 | | Rho GTPase activating protein 28 | -16.373019 | -2.194672 | 0.000562075 | 0.012582804 |
| 220164 | NM_152721 DOK6 | | docking protein 6 | -16.373019 | -2.331440 | 0.000562075 | 0.012582804 |
| 652995 | NR_015379 UCA1 | | urothelial cancer associated 1 (non-protein coding) | -15.744812 | -2.172396 | 0.000679941 | 0.014589822 |
| 55203 | NM_018176 LGI2 | | leucine rich repeat LGI family member 2 | -15.733755 | -1.674484 | 0.000409502 | 0.009751222 |
| 9839 | NM_001171653ZEB2 | | zinc finger E-box binding homeobox 2 | -15.620687 | 3.330862 | 2.53591E-05 | 0.001099694 |
| 90952 | NM_138961 ESAM | | endothelial cell adhesion molecule | -15.566859 | 2.839507 | 2.72664E-05 | 0.001170134 |
| 80086 | NR_003063 TUBA4B | | tubulin alpha 4b | -15.495820 | -1.869101 | 0.000442347 | 0.010445154 |
| 9509 | NM_014244,N ADAMTS2 | | ADAM metallopeptidase with thrombospondin type 1 motif | -15.304282 | -2.673040 | 0.001904897 | 0.032297378 |
| 162466 | NM_001143804PHOSPHO1 | | phosphoethanolamine/phosphocholine phosphatase | -15.225374 | 0.292432 | 9.08227E-05 | 0.003039914 |
| 269 | NM_001164690AMHR2 | | anti-Mullerian hormone receptor type 2 | -15.112844 | -0.941526 | 0.000153086 | 0.004582618 |
| 4804 | NM_002507 NGFR | | nerve growth factor receptor | -15.077229 | 0.686777 | 6.76503E-05 | 0.002392045 |
| 83858 | NM_001317238ATAD3B | | ATPase family, AAA domain containing 3B | -14.861131 | 1.465772 | 4.61396E-05 | 0.001809717 |
| 55086 | NM_001184782CXorf57 | | chromosome X open reading frame 57 | -14.795237 | -0.037074 | 7.91624E-05 | 0.002697653 |
| 4688 | NM_000433,N NCF2 | | neutrophil cytosolic factor 2 | -14.791740 | 1.964369 | 4.07908E-05 | 0.001637714 |
| 467 | NM_001030287ATF3 | | activating transcription factor 3 | -14.711604 | 5.312282 | 3.26861E-05 | 0.001363127 |
| 250 | NM_001632 ALPP | | alkaline phosphatase, placental | -14.578025 | -1.755540 | 0.000394426 | 0.009587201 |
| 8061 | NM_001300844FOSL1 | | FOS like 1, AP-1 transcription factor subunit | -14.467720 | 6.234512 | 3.52258E-05 | 0.001445727 |
| 10871 | NM_006678 CD300C | | CD300c molecule | -14.380283 | -2.832148 | 0.002516721 | 0.039830432 |
| 33 | NM_001608 ACADL | | acyl-CoA dehydrogenase, long chain | -14.316770 | -1.008809 | 0.000173441 | 0.005045891 |
| 51440 | NM_001282396HPCAL4 | | hippocalcin like 4 | -14.195029 | -0.584391 | 0.000451574 | 0.010626636 |

| 57172 | NM_020439 | CAMK1G | calcium/calmodulin dependent protein kinase IG | -13.918283 | -2.165488 | 0.002906428 | 0.043497646 |
| --- | --- | --- | --- | --- | --- | --- | --- |
| 3772 | NM_001276435KCNJ15 | | potassium voltage-gated channel subfamily J member 15 | -13.830273 | -2.032297 | 0.000780726 | 0.016230464 |
| 164781 | NM_001330004DAW1 | | dynein assembly factor with WD repeats 1 | -13.814010 | 1.196359 | 6.42397E-05 | 0.002302944 |
| 4613 | NM_001293228MYCN | | MYCN proto-oncogene, bHLH transcription factor | -13.592337 | -2.196508 | 0.0008504 | 0.01753791 |
| 2825 | NM_001098199GPR1 | | G protein-coupled receptor 1 | -13.479295 | -1.390443 | 0.000430265 | 0.010183103 |
| 26047 | NM_014141 CNTNAP2 | | contactin associated protein-like 2 | -13.435472 | 1.944299 | 6.63951E-05 | 0.002359764 |
| 81796 | NM_001146008SLCO5A1 | | solute carrier organic anion transporter family member 5A1 | -13.365900 | 0.223334 | 0.000116264 | 0.003717032 |
| 7474 | NM_001256105WNT5A | | Wnt family member 5A | -13.237538 | -1.544599 | 0.000641766 | 0.013929631 |
| 348013 | NM_001348663TMEM255B | | transmembrane protein 255B | -13.231984 | -1.698340 | 0.001550588 | 0.027726851 |
| 23630 | NM_012282 KCNE5 | | potassium voltage-gated channel subfamily E regulatory su | -13.206660 | -3.123860 | 0.016225581 | 0.146504993 |
| 9636 | NM_005101 ISG15 | | ISG15 ubiquitin-like modifier | -12.966798 | 3.469248 | 6.93493E-05 | 0.002430804 |
| 51129 | NM_001039667ANGPTL4 | | angiopoietin like 4 | -12.916211 | 5.371765 | 6.73569E-05 | 0.002385749 |
| 760 | NM_000067,N CA2 | | carbonic anhydrase 2 | -12.848472 | 2.606223 | 7.86218E-05 | 0.002683651 |
| 1004 | NM_004932 CDH6 | | cadherin 6 | -12.846651 | 0.329357 | 0.000117213 | 0.00374159 |
| 81706 | NM_030949 PPP1R14C | | protein phosphatase 1 regulatory inhibitor subunit 14C | -12.812576 | 0.875661 | 0.000121694 | 0.00385488 |
| 7074 | NM_003253 TIAM1 | | T-cell lymphoma invasion and metastasis 1 | -12.736734 | 1.407760 | 0.000128818 | 0.004001664 |
| 54739 | NM_017523,N XAF1 | | XIAP associated factor 1 | -12.603777 | -2.531771 | 0.001939802 | 0.032808511 |
| 100129083 | NM_001256795LOC100129083 | | uncharacterized LOC100129083 | -12.532285 | -2.472807 | 0.004569388 | 0.060705064 |
| 374897 | NM_001166034SBSN | | suprabasin | -12.403317 | 1.084972 | 0.000114733 | 0.003673766 |
| 2303 | NM_005251 FOXC2 | | forkhead box C2 (MFH-1, mesenchyme forkhead 1) | -12.399862 | -0.395357 | 0.000216884 | 0.006005693 |
| 8843 | NM_006018 HCAR3 | | hydroxycarboxylic acid receptor 3 | -12.164725 | -1.217613 | 0.00132779 | 0.02456649 |
| 81553 | NM_030797 FAM49A | | family with sequence similarity 49 member A | -12.088549 | -1.767923 | 0.001005703 | 0.019934819 |
| 3603 | NM_001172128IL16 | | interleukin 16 | -12.070285 | -3.171151 | 0.005352624 | 0.067925788 |
| 342931 | NM_001145014RFPL4A | | ret finger protein like 4A | -12.070285 | -3.171151 | 0.005352624 | 0.067925788 |
| 100303453 | NR_028393,NR TSNAX-DISC1 | | TSNAX-DISC1 readthrough (NMD candidate) | -11.971822 | 0.076557 | 0.000290485 | 0.007492135 |
| 105369486 | NR_135100 LOC105369486 | | uncharacterized LOC105369486 | -11.745310 | -0.472072 | 0.000348038 | 0.008589436 |
| 26166 | NM_001286692RGS22 | | regulator of G protein signaling 22 | -11.705553 | -1.102489 | 0.001176763 | 0.022311039 |
| 3290 | NM_001206741HSD11B1 | | hydroxysteroid 11-beta dehydrogenase 1 | -11.661466 | -2.558334 | 0.002756531 | 0.042710739 |
| 4889 | NM_001317091NPY5R | | neuropeptide Y receptor Y5 | -11.608286 | -3.214037 | 0.006295194 | 0.076320644 |
| 6853 | NM_006950,N SYN1 | | synapsin I | -11.608286 | -2.898802 | 0.006295194 | 0.076320644 |
| 283768 | NM_001350919GOLGA8G | | golgin A8 family member G | -11.608286 | -3.009166 | 0.006295194 | 0.076320644 |
| 90226 | NM_033199 UCN2 | | urocortin 2 | -11.581302 | 2.152906 | 0.000146899 | 0.004440584 |
| 84465 | NM_032445 MEGF11 | | multiple EGF like domains 11 | -11.514054 | -0.946784 | 0.001274794 | 0.023820337 |
| 1756 | NM_000109,N DMD | | dystrophin | -11.489420 | -1.497003 | 0.000631192 | 0.013772371 |
| 91752 | NM_194250 ZNF804A | | zinc finger protein 804A | -11.489420 | -1.470384 | 0.000631192 | 0.013772371 |
| 105370526 | NR_144368 LOC105370526 | | uncharacterized LOC105370526 | -11.460462 | -2.605639 | 0.026706022 | 0.207532496 |
| 56944 | NM_001286352OLFML3 | | olfactomedin like 3 | -11.458646 | 1.993061 | 0.000153687 | 0.004593941 |
| 618 | NR_001568 BCYRN1 | | brain cytoplasmic RNA 1 | -11.396343 | -1.233506 | 0.000996744 | 0.019854122 |
| 8862 | NM_017413 APLN | | apelin | -11.386109 | 0.061962 | 0.000258585 | 0.006883963 |
| 11178 | NM_021020 LZTS1 | | leucine zipper tumor suppressor 1 | -11.146286 | -2.934234 | 0.007434817 | 0.086278476 |
| 101927528 | NR_104644,NR LINC01204 | | long intergenic non-protein coding RNA 1204 | -11.146286 | -2.581361 | 0.007434817 | 0.086278476 |
| 1674 | NM_001927 DES | | desmin | -11.033259 | -2.800552 | 0.003523399 | 0.050788504 |
| 64409 | NM_022479 WBSCR17 | | Williams-Beuren syndrome chromosome region 17 | -11.033259 | -2.800552 | 0.003523399 | 0.050788504 |

| 316 | NM_001159 | AOX1 | aldehyde oxidase 1 | -11.030663 | 2.479225 | 0.000174014 | 0.005052482 |
| --- | --- | --- | --- | --- | --- | --- | --- |
| 5054 | NM_000602 | SERPINE1 | serpin family E member 1 | -11.025483 | 9.062439 | 0.000154312 | 0.00460597 |
| 25907 | NM_015444 | TMEM158 | transmembrane protein 158 (gene/pseudogene) | -10.790615 | 2.977641 | 0.000210421 | 0.005851824 |
| 100533195 | NR_037915 | FAM24B-CUZD1 | FAM24B-CUZD1 readthrough | -10.737113 | -1.368309 | 0.002369975 | 0.038071568 |
| 90161 | NM_001077188HS6ST2 heparan sulfate 6-O-sulfotransferase 2 | | | -10.618030 | -0.091339 | 0.000418001 | 0.009915548 |
| 50614 | NM_001122636GALNT9 polypeptide N-acetylgalactosaminyltransferase 9 | | | -10.588170 | 1.860124 | 0.000250631 | 0.006741612 |
| 5798 | NM_001199763PTPRN protein tyrosine phosphatase, receptor type N | | | -10.450403 | -0.729848 | 0.000632613 | 0.013788822 |
| 51286 | NM_016564 CEND1 cell cycle exit and neuronal differentiation 1 | | | -10.434980 | -1.145075 | 0.001530156 | 0.027498935 |
| 79589 | NM_024539,N RNF128 ring finger protein 128, E3 ubiquitin protein ligase | | | -10.299065 | 0.876661 | 0.000297328 | 0.007621112 |
| 692086 | NR_003045 SNORD17 small nucleolar RNA, C/D box 17 | | | -10.299065 | 1.631212 | 0.000297328 | 0.007621112 |
| 4605 | NM_001278610MYBL2 MYB proto-oncogene like 2 | | | -10.268638 | 6.557880 | 0.000226778 | 0.006246214 |
| 4319 | NM_002425 MMP10 matrix metallopeptidase 10 | | | -10.237238 | 0.003753 | 0.000402386 | 0.009700885 |
| 23316 | NM_015267 CUX2 cut like homeobox 2 | | | -10.222287 | -2.933366 | 0.010510413 | 0.110415384 |
| 102724360 | NR_134912,NR LOC102724360 uncharacterized LOC102724360 | | | -10.222287 | -2.794974 | 0.010510413 | 0.110415384 |
| 389119 | NM_203370 FAM212A family with sequence similarity 212 member A | | | -10.173567 | -1.582624 | 0.002106798 | 0.035172821 |
| 991 | NM_001255 CDC20 cell division cycle 20 | | | -10.094932 | 6.697745 | 0.00024807 | 0.006685785 |
| 797 | NM_000728 CALCB calcitonin related polypeptide beta | | | -10.090948 | -2.324265 | 0.005186874 | 0.066228702 |
| 8291 | NM_001130455DYSF dysferlin | | | -10.048614 | -0.385305 | 0.000611338 | 0.01342413 |
| 8676 | NM_003764 STX11 syntaxin 11 | | | -10.003797 | 0.971289 | 0.000416654 | 0.009894946 |
| 9242 | NM_005098 MSC musculin | | | -9.938069 | 3.570284 | 0.000285066 | 0.007393951 |
| 55388 | NM_018518,N MCM10 minichromosome maintenance 10 replication initiation fact | | | -9.913830 | 5.770255 | 0.000274534 | 0.007215675 |
| 4900 | NM_001126181NRGN neurogranin | | | -9.879282 | 1.879830 | 0.000385488 | 0.009391279 |
| 26059 | NM_015576,NRERC2 ELKS/RAB6-interacting/CAST family member 2 | | | -9.760287 | -2.794556 | 0.012586629 | 0.125356208 |
| 54065 | NM_058182 SMIM11A small integral membrane protein 11A | | | -9.714264 | -3.862302 | 0.045799632 | 0.294945637 |
| 373861 | NR_024192,NR HILS1 histone linker H1 domain, spermatid-specific 1 (pseudogen | | | -9.714264 | -4.085556 | 0.045799632 | 0.294945637 |
| 100507127 | NR_038291 LINC00707 long intergenic non-protein coding RNA 707 | | | -9.595577 | 4.364412 | 0.000334963 | 0.008398431 |
| 1462 | NM_001126336VCAN versican | | | -9.587269 | 6.930816 | 0.000324691 | 0.008160663 |
| 4803 | NM_002506 NGF nerve growth factor | | | -9.537513 | -1.082466 | 0.001092825 | 0.021383887 |
| 8682 | NM_001297576PEA15 phosphoprotein enriched in astrocytes 15 | | | -9.530576 | 6.442549 | 0.000335784 | 0.008408839 |
| 3434 | NM_001270927IFIT1 interferon induced protein with tetratricopeptide repeats 1 | | | -9.488074 | 4.325316 | 0.00035418 | 0.008721689 |
| 100529067 | NR_037673 SERF2-C15ORF63 SERF2-C15orf63 readthrough | | | -9.483242 | 3.712320 | 0.000365766 | 0.008974928 |
| 56999 | NM_001318781ADAMTS9 ADAM metallopeptidase with thrombospondin type 1 motif | | | -9.456307 | 3.348388 | 0.0003734 | 0.009151409 |
| 7277 | NM_001278552TUBA4A tubulin alpha 4a | | | -9.310574 | 7.508656 | 0.000377943 | 0.009251765 |
| 101927279 | NR_110514 EP300-AS1 EP300 antisense RNA 1 | | | -9.309502 | -1.622202 | 0.004481104 | 0.05982482 |
| 63967 | NM_001190481CLSPN claspin | | | -9.268911 | 5.360875 | 0.000396923 | 0.00962527 |
| 993 | NM_001789,N CDC25A cell division cycle 25A | | | -9.256939 | 4.511407 | 0.000402202 | 0.009700885 |
| 22989 | NM_014981 MYH15 myosin heavy chain 15 | | | -9.250172 | 1.593565 | 0.000578901 | 0.012917555 |
| 10391 | NM_001190456CORO2B coronin 2B | | | -9.182626 | 1.773080 | 0.000506218 | 0.011494087 |
| 5618 | NM_000949,N PRLR prolactin receptor | | | -9.153163 | -2.032214 | 0.002845074 | 0.0427381 |
| 105371964 | NR_146506 LINC01895 long intergenic non-protein coding RNA 1895 | | | -9.153163 | -2.071642 | 0.002845074 | 0.0427381 |
| 84141 | NM_001135032EVA1A eva-1 homolog A, regulator of programmed cell death | | | -9.016601 | 1.972824 | 0.0005294 | 0.011915814 |
| 51200 | NM_001163446CPA4 carboxypeptidase A4 | | | -9.013774 | 3.258789 | 0.000487942 | 0.01131223 |
| 25819 | NM_012118 NOCT nocturnin | | | -8.932460 | 3.237838 | 0.00050184 | 0.011432334 |

| 241 | NM_001204406ALOX5AP | arachidonate 5-lipoxygenase activating protein | -8.928878 | -1.140768 | 0.001670126 | 0.029276743 |
| --- | --- | --- | --- | --- | --- | --- |
| 64386 | NM_022468 MMP25 | matrix metallopeptidase 25 | -8.920062 | -1.012012 | 0.00151661 | 0.027350547 |
| 11217 | NM_001004065AKAP2 | A-kinase anchoring protein 2 | -8.849828 | 6.160203 | 0.00049438 | 0.01131223 |
| 57094 | NM_020361 CPA6 | carboxypeptidase A6 | -8.836288 | -3.502360 | 0.018334706 | 0.159820227 |
| 1013 | NM_004933 CDH15 | cadherin 15 | -8.832241 | -1.820680 | 0.002282946 | 0.036950501 |
| 64219 | NM_001032396PJA1 | praja ring finger ubiquitin ligase 1 | -8.818150 | 4.109297 | 0.000527171 | 0.011891521 |
| 100526760 | NM_001316331ABHD14A-ACY1 | ABHD14A-ACY1 readthrough | -8.793383 | 1.609416 | 0.000713281 | 0.015148068 |
| 93082 | NM_001285485NEURL3 | neuralized E3 ubiquitin protein ligase 3 | -8.749944 | -0.000178 | 0.000920307 | 0.018500043 |
| 65072 | NR_040030 CFLAR-AS1 | CFLAR antisense RNA 1 | -8.672482 | -1.955484 | 0.003355072 | 0.048769965 |
| 8989 | NM_007332 TRPA1 | transient receptor potential cation channel subfamily A me | -8.636455 | 2.980702 | 0.000595546 | 0.013229577 |
| 2290 | NM_005249 FOXG1 | forkhead box G1 | -8.595696 | -2.644544 | 0.006319303 | 0.076320644 |
| 389058 | NM_001003845SP5 | Sp5 transcription factor | -8.512255 | -1.420673 | 0.003649256 | 0.051642736 |
| 220042 | NM_145018 DDIAS | DNA damage induced apoptosis suppressor | -8.506228 | 4.896399 | 0.000618636 | 0.013541261 |
| 10381 | NM_001197181TUBB3 | tubulin beta 3 class III | -8.496255 | 7.762580 | 0.000606619 | 0.013334657 |
| 5218 | NM_001287135CDK14 | cyclin dependent kinase 14 | -8.469899 | -1.531316 | 0.002780495 | 0.0427381 |
| 10060 | NM_005691,N ABCC9 | ATP binding cassette subfamily C member 9 | -8.450084 | -2.479641 | 0.004860852 | 0.062881001 |
| 53335 | NM_018014,N BCL11A | B-cell CLL/lymphoma 11A | -8.374289 | -2.968371 | 0.022314819 | 0.184819135 |
| 2069 | NM_001432 EREG | epiregulin | -8.366878 | 8.280467 | 0.000656065 | 0.014225056 |
| 9088 | NM_001258450PKMYT1 | protein kinase, membrane associated tyrosine/threonine 1 | -8.344803 | 5.226655 | 0.000676435 | 0.014544766 |
| 51330 | NM_016639 TNFRSF12A | TNF receptor superfamily member 12A | -8.324923 | 7.160533 | 0.000673994 | 0.014507344 |
| 654 | NM_001718 BMP6 | bone morphogenetic protein 6 | -8.286244 | 3.621226 | 0.000717259 | 0.015216936 |
| 221687 | NM_001165032RNF182 | ring finger protein 182 | -8.236601 | 1.647927 | 0.000965429 | 0.019313252 |
| 100126791 | NR_004428 EGOT | eosinophil granule ontogeny transcript (non-protein coding | -8.119825 | -2.210519 | 0.008030972 | 0.091880895 |
| 129607 | NM_001256477CMPK2 | cytidine/uridine monophosphate kinase 2 | -8.080062 | -0.119823 | 0.001307761 | 0.02427302 |
| 338761 | NM_001008223C1QL4 | complement C1q like 4 | -8.063331 | 1.578165 | 0.001115456 | 0.021383887 |
| 202 | NM_001624 CRYBG1 | crystallin beta-gamma domain containing 1 | -8.032452 | 1.812765 | 0.001048425 | 0.020634325 |
| 374393 | NM_001142703FAM111B | family with sequence similarity 111 member B | -8.003239 | 4.833103 | 0.000842751 | 0.017414891 |
| 677768 | NR_003002 SCARNA13 | small Cajal body-specific RNA 13 | -7.912289 | -3.501363 | 0.027322057 | 0.210330017 |
| 100422781 | NR_147505 LOC100422781 | uncharacterized LOC100422781 | -7.912289 | -2.636685 | 0.027322057 | 0.210330017 |
| 5187 | NM_002616 PER1 | period circadian clock 1 | -7.907136 | 5.273024 | 0.000880882 | 0.018022792 |
| 7476 | NM_004625 WNT7A | Wnt family member 7A | -7.881890 | -2.833301 | 0.0090842 | 0.098608124 |
| 9134 | NM_057749 CCNE2 | cyclin E2 | -7.814412 | 3.488831 | 0.000981028 | 0.019606333 |
| 4856 | NM_002514 NOV | nephroblastoma overexpressed | -7.771980 | 1.611655 | 0.001196659 | 0.022626034 |
| 26038 | NM_015557 CHD5 | chromodomain helicase DNA binding protein 5 | -7.755470 | 1.225748 | 0.00127864 | 0.023870636 |
| 3433 | NM_001547 IFIT2 | interferon induced protein with tetratricopeptide repeats 2 | -7.724171 | 4.438513 | 0.001007439 | 0.019934819 |
| 105416157 | NR_131157 NKILA | NF-kappaB interacting lncRNA | -7.699347 | 2.457885 | 0.001144435 | 0.021757947 |
| 10170 | NM_001142270DHRS9 | dehydrogenase/reductase 9 | -7.652375 | 0.766826 | 0.001439522 | 0.026074 |
| 2810 | NM_006142 SFN | stratifin | -7.648322 | 6.824698 | 0.001035537 | 0.020419522 |
| 83700 | NM_001205329JAM3 | junctional adhesion molecule 3 | -7.642358 | 2.596530 | 0.001138034 | 0.021676096 |
| 133396 | NM_001242636IL31RA | interleukin 31 receptor A | -7.638736 | 1.948860 | 0.001261388 | 0.023633884 |
| 4973 | NM_001172632OLR1 | oxidized low density lipoprotein receptor 1 | -7.617793 | 2.208912 | 0.002959829 | 0.044077797 |
| 146760 | NM_178568 RTN4RL1 | reticulon 4 receptor like 1 | -7.609898 | 1.289000 | 0.001323757 | 0.024513803 |
| 8877 | NM_001142601SPHK1 | sphingosine kinase 1 | -7.600958 | 3.399975 | 0.001127625 | 0.021547706 |

| 340206 | NR_027256 | TREML3P | triggering receptor expressed on myeloid cells like 3, pseud | -7.592824 | -2.174594 | 0.00499616 | 0.064269639 |
| --- | --- | --- | --- | --- | --- | --- | --- |
| 130574 | NM_001195685LYPD6 | | LY6/PLAUR domain containing 6 | -7.571997 | -0.950736 | 0.003640076 | 0.051642736 |
| 2491 | NM_001318521CENPI | | centromere protein I | -7.568195 | 4.678639 | 0.001124851 | 0.021524096 |
| 56062 | NM_019117,N KLHL4 | | kelch like family member 4 | -7.540546 | 3.534986 | 0.001175705 | 0.022311039 |
| 283455 | NM_173598 KSR2 | | kinase suppressor of ras 2 | -7.536911 | 1.687952 | 0.001285204 | 0.023949959 |
| 9260 | NM_005451,N PDLIM7 | | PDZ and LIM domain 7 | -7.536395 | 5.722721 | 0.001120838 | 0.021467162 |
| 148281 | NM_001270805SYT6 | | synaptotagmin 6 | -7.518516 | 0.350192 | 0.001587416 | 0.028282264 |
| 94274 | NM_001243947PPP1R14A | | protein phosphatase 1 regulatory inhibitor subunit 14A | -7.512777 | 5.196760 | 0.001141155 | 0.021715539 |
| 23284 | NM_001322246ADGRL3 | | adhesion G protein-coupled receptor L3 | -7.505759 | 0.607079 | 0.00146759 | 0.026512757 |
| 2151 | NM_001256566F2RL2 | | coagulation factor II thrombin receptor like 2 | -7.486679 | 4.233528 | 0.001183382 | 0.022415995 |
| 374987 | NR_103535 NEXN-AS1 | | NEXN antisense RNA 1 | -7.455089 | -1.686715 | 0.005415811 | 0.068358604 |
| 7123 | NM_001308394CLEC3B | | C-type lectin domain family 3 member B | -7.450290 | -3.555585 | 0.033664757 | 0.237988891 |
| 27201 | NM_080819,NRGPR78 | | G protein-coupled receptor 78 | -7.450290 | -3.671629 | 0.033664757 | 0.237988891 |
| 26499 | NM_016445 PLEK2 | | pleckstrin 2 | -7.441788 | 4.398239 | 0.001226527 | 0.02308527 |
| 9244 | NM_004750 CRLF1 | | cytokine receptor like factor 1 | -7.434027 | -1.178252 | 0.004431119 | 0.059517988 |
| 699 | NM_001278616BUB1 | | BUB1 mitotic checkpoint serine/threonine kinase | -7.419770 | 6.730745 | 0.001207036 | 0.022801399 |
| 64151 | NM_022346,NRNCAPG | | non-SMC condensin I complex subunit G | -7.414441 | 6.166481 | 0.001214865 | 0.022886603 |
| 54821 | NM_001009954ERCC6L | | ERCC excision repair 6 like, spindle assembly checkpoint he | -7.343792 | 5.068722 | 0.001295677 | 0.024101684 |
| 115572 | NM_052943 FAM46B | | family with sequence similarity 46 member B | -7.315565 | -0.344111 | 0.002587337 | 0.040760895 |
| 79733 | NM_001256371E2F8 | | E2F transcription factor 8 | -7.298741 | 3.702224 | 0.00137088 | 0.025273301 |
| 51309 | NM_016608 ARMCX1 | | armadillo repeat containing, X-linked 1 | -7.281237 | -1.677609 | 0.004357651 | 0.058913728 |
| 79801 | NM_001324318SHCBP1 | | SHC binding and spindle associated 1 | -7.225466 | 6.244106 | 0.001380371 | 0.025331018 |
| 6299 | NM_001127892SALL1 | | spalt like transcription factor 1 | -7.214298 | 0.577826 | 0.001809505 | 0.030959154 |
| 9241 | NM_005450 NOG | | noggin | -7.186867 | -0.484491 | 0.003616011 | 0.051642736 |
| 9568 | NM_005458 GABBR2 | | gamma-aminobutyric acid type B receptor subunit 2 | -7.179618 | -1.918528 | 0.006383312 | 0.076323009 |
| 57452 | NM_001168368GALNT16 | | polypeptide N-acetylgalactosaminyltransferase 16 | -7.164324 | 1.812809 | 0.001756755 | 0.030310349 |
| 10656 | NM_006558 KHDRBS3 | | KH RNA binding domain containing, signal transduction ass | -7.103212 | 1.603529 | 0.0018693 | 0.031834193 |
| 8318 | NM_001178010CDC45 | | cell division cycle 45 | -7.098743 | 4.842157 | 0.0015281 | 0.027485864 |
| 55723 | NM_018154 ASF1B | | anti-silencing function 1B histone chaperone | -7.092194 | 4.917995 | 0.001539907 | 0.027626168 |
| 64866 | NM_022842,N CDCP1 | | CUB domain containing protein 1 | -7.087317 | 6.517270 | 0.001526239 | 0.027476289 |
| 84561 | NM_001195483SLC12A8 | | solute carrier family 12 member 8 | -7.076092 | -0.105502 | 0.002826714 | 0.0427381 |
| 84660 | NM_201435,NRCCDC62 | | coiled-coil domain containing 62 | -7.070211 | -2.170737 | 0.008202272 | 0.093581908 |
| 55247 | NM_018248 NEIL3 | | nei like DNA glycosylase 3 | -7.005015 | 4.952221 | 0.001633285 | 0.028949879 |
| 284207 | NM_001004431METRNL | | meteorin like, glial cell differentiation regulator | -7.004239 | 2.588772 | 0.001756931 | 0.030310349 |
| 54443 | NM_001284301ANLN | | anillin actin binding protein | -6.988501 | 7.801392 | 0.001619804 | 0.028735548 |
| 3113 | NM_001242524HLA-DPA1 | | major histocompatibility complex, class II, DP alpha 1 | -6.988290 | -3.732801 | 0.041756881 | 0.278177479 |
| 990 | NM_001254 CDC6 | | cell division cycle 6 | -6.968219 | 6.503529 | 0.001647584 | 0.029089147 |
| 79412 | NM_001253725KREMEN2 | | kringle containing transmembrane protein 2 | -6.953486 | 1.440172 | 0.002120482 | 0.035344222 |
| 78986 | NM_001305115DUSP26 | | dual specificity phosphatase 26 | -6.949913 | -2.668768 | 0.022910824 | 0.18519359 |
| 6563 | NM_001128588SLC14A1 | | solute carrier family 14 member 1 (Kidd blood group) | -6.930148 | -2.973516 | 0.015240223 | 0.144078619 |
| 145270 | NM_178013 PRIMA1 | | proline rich membrane anchor 1 | -6.909984 | -1.698935 | 0.009033387 | 0.098345056 |
| 157313 | NM_001317906CDCA2 | | cell division cycle associated 2 | -6.877624 | 6.017500 | 0.001762288 | 0.030352691 |
| 8193 | NM_001135155DPF1 | | double PHD fingers 1 | -6.833823 | 2.350686 | 0.002072007 | 0.034704018 |

| 205860 | NM_001303419TRIML2 | tripartite motif family like 2 | -6.819998 | 5.099867 | 0.0018427 | 0.031423123 |
| --- | --- | --- | --- | --- | --- | --- |
| 11065 | NM_001281741UBE2C | ubiquitin conjugating enzyme E2 C | -6.748738 | 6.259590 | 0.001929312 | 0.032657793 |
| 80320 | NM_001258248SP6 | Sp6 transcription factor | -6.707282 | 2.156546 | 0.002234109 | 0.036273586 |
| 79365 | NM_030762 BHLHE41 | basic helix-loop-helix family member e41 | -6.700168 | 2.341602 | 0.002165196 | 0.036002479 |
| 2620 | NM_001143830GAS2 | growth arrest specific 2 | -6.692213 | -3.010820 | 0.017459041 | 0.154065215 |
| 23768 | NM_001346143FLRT2 | fibronectin leucine rich transmembrane protein 2 | -6.686356 | 1.905912 | 0.002192287 | 0.036130384 |
| 145501 | NM_182509,N ISM2 | isthmin 2 | -6.665900 | -0.705626 | 0.004001999 | 0.055077408 |
| 50506 | NM_014080 DUOX2 | dual oxidase 2 | -6.658186 | -1.583458 | 0.008248451 | 0.093901599 |
| 375704 | NM_198573 ENHO | energy homeostasis associated | -6.658186 | -1.396015 | 0.008248451 | 0.093901599 |
| 85366 | NM_033118 MYLK2 | myosin light chain kinase 2 | -6.646613 | -0.888413 | 0.005543636 | 0.069793129 |
| 2047 | NM_004441 EPHB1 | EPH receptor B1 | -6.635810 | -3.169013 | 0.027120138 | 0.209633802 |
| 23138 | NM_015111 N4BP3 | NEDD4 binding protein 3 | -6.631249 | 1.484157 | 0.002669165 | 0.041669194 |
| 101927957 | NR_126330 LINC01572 | long intergenic non-protein coding RNA 1572 | -6.628677 | -1.120495 | 0.008983126 | 0.09792029 |
| 9156 | NM_001319224EXO1 | exonuclease 1 | -6.624592 | 5.277475 | 0.002133208 | 0.035527711 |
| 100287314 | NR_040245 LINC00941 | long intergenic non-protein coding RNA 941 | -6.611566 | 2.970499 | 0.002271615 | 0.036795898 |
| 113157 | NR_002775 RPLP0P2 | ribosomal protein lateral stalk subunit P0 pseudogene 2 | -6.573684 | -1.784076 | 0.007678842 | 0.088488499 |
| 9572 | NM_021724 NR1D1 | nuclear receptor subfamily 1 group D member 1 | -6.566981 | 5.260629 | 0.002206311 | 0.036130384 |
| 55165 | NM_001127182CEP55 | centrosomal protein 55 | -6.559775 | 6.835092 | 0.002211281 | 0.036130384 |
| 3038 | NM_001199280HAS3 | hyaluronan synthase 3 | -6.544873 | 5.075270 | 0.002249326 | 0.036492014 |
| 148203 | NR_027130 ZNF738 | zinc finger protein 738 | -6.538467 | -1.327337 | 0.006454321 | 0.076949641 |
| 284403 | NM_001083961WDR62 | WD repeat domain 62 | -6.491519 | 6.164931 | 0.002330118 | 0.037603259 |
| 54658 | NM_000463 UGT1A1 | UDP glucuronosyltransferase family 1 member A1 | -6.481062 | 2.811942 | 0.002459475 | 0.039174859 |
| 79000 | NM_001287490AUNIP | aurora kinase A and ninein interacting protein | -6.480382 | 2.693088 | 0.002605979 | 0.040867837 |
| 857 | NM_001172895CAV1 | caveolin 1 | -6.474681 | 8.099945 | 0.00234709 | 0.037811188 |
| 29128 | NM_001048201UHRF1 | ubiquitin like with PHD and ring finger domains 1 | -6.474623 | 6.334973 | 0.002356896 | 0.037939608 |
| 4502 | NM_005953 MT2A | metallothionein 2A | -6.471254 | 6.591527 | 0.002360402 | 0.037966501 |
| 10622 | NM_006467 POLR3G | RNA polymerase III subunit G | -6.467055 | 4.841308 | 0.002398734 | 0.038433635 |
| 6274 | NM_002960 S100A3 | S100 calcium binding protein A3 | -6.459125 | 4.162597 | 0.002460147 | 0.039174859 |
| 55355 | NM_001282962HJURP | Holliday junction recognition protein | -6.433949 | 7.187973 | 0.002423152 | 0.038734853 |
| 22979 | NM_001319099EFR3B | EFR3 homolog B | -6.430496 | 5.372991 | 0.002444917 | 0.039018654 |
| 5888 | NM_001164269RAD51 | RAD51 recombinase | -6.403462 | 3.670019 | 0.002600164 | 0.040865545 |
| 56896 | NM_001253723DPYSL5 | dihydropyrimidinase like 5 | -6.402655 | 3.138973 | 0.002652658 | 0.041444069 |
| 8482 | NM_001146029SEMA7A | semaphorin 7A (John Milton Hagen blood group) | -6.374507 | 0.850981 | 0.003416764 | 0.049527507 |
| 8013 | NM_006981,N NR4A3 | nuclear receptor subfamily 4 group A member 3 | -6.344757 | 2.512823 | 0.002765403 | 0.0427381 |
| 2898 | NM_001166247GRIK2 | glutamate ionotropic receptor kainate type subunit 2 | -6.343604 | -2.800025 | 0.016035504 | 0.146302603 |
| 339535 | NR_015407 LINC01139 | long intergenic non-protein coding RNA 1139 | -6.326534 | -0.161217 | 0.003842336 | 0.053629365 |
| 4998 | NM_001190818ORC1 | origin recognition complex subunit 1 | -6.324680 | 4.802248 | 0.002684541 | 0.04181456 |
| 3938 | NM_002299 LCT | lactase | -6.321706 | -2.638026 | 0.03224147 | 0.233618934 |
| 122786 | NM_001042481FRMD6 | FERM domain containing 6 | -6.312142 | 5.092472 | 0.002676835 | 0.041725954 |
| 100507003 | NM_001195256GFY | golgi associated olfactory signaling regulator | -6.295844 | -2.050413 | 0.009676606 | 0.103388741 |
| 195828 | NM_153695 ZNF367 | zinc finger protein 367 | -6.292213 | 4.210563 | 0.002769635 | 0.0427381 |
| 116496 | NM_052966 FAM129A | family with sequence similarity 129 member A | -6.262394 | 4.668065 | 0.002823029 | 0.0427381 |
| 84541 | NM_032505 KBTBD8 | kelch repeat and BTB domain containing 8 | -6.254246 | 0.888575 | 0.003679422 | 0.051642736 |

| 595135 | NR_002836 | PGM5P2 | phosphoglucomutase 5 pseudogene 2 | -6.251056 | -1.833220 | 0.009555837 | 0.102415798 |
| --- | --- | --- | --- | --- | --- | --- | --- |
| 79075 | NM_024094 | DSCC1 | DNA replication and sister chromatid cohesion 1 | -6.200228 | 4.196791 | 0.002965912 | 0.04413661 |
| 23603 | NM_001105237CORO1C | | coronin 1C | -6.136118 | 8.184893 | 0.003036826 | 0.045074119 |
| 27145 | NM_001289987FILIP1 | | filamin A interacting protein 1 | -6.129530 | 1.659354 | 0.00337428 | 0.048980339 |
| 79413 | NM_024508 ZBED2 | | zinc finger BED-type containing 2 | -6.121398 | 1.327610 | 0.004317098 | 0.058518458 |
| 8534 | NM_003654 CHST1 | | carbohydrate sulfotransferase 1 | -6.100928 | -0.166006 | 0.005619322 | 0.07054368 |
| 23406 | NM_021149 COTL1 | | coactosin like F-actin binding protein 1 | -6.097710 | 7.859997 | 0.003129626 | 0.046133225 |
| 27063 | NM_014391 ANKRD1 | | ankyrin repeat domain 1 | -6.097213 | 2.768802 | 0.003310381 | 0.04835822 |
| 6335 | NM_002977 SCN9A | | sodium voltage-gated channel alpha subunit 9 | -6.063204 | 4.406026 | 0.003262324 | 0.047757373 |
| 105369340 | NR_135086,NR LOC105369340 | | uncharacterized LOC105369340 | -6.041211 | 1.862604 | 0.00367313 | 0.051642736 |
| 333926 | NM_005167 PPM1J | | protein phosphatase, Mg2+/Mn2+ dependent 1J | -6.037257 | 0.051450 | 0.00558002 | 0.070123149 |
| 55022 | NM_001100818PID1 | | phosphotyrosine interaction domain containing 1 | -6.030451 | 1.419381 | 0.004080842 | 0.055902135 |
| 107161151 | NR_135011,NR AOX3P-AOX2P | | AOX3P-AOX2P readthrough | -6.007603 | -3.256061 | 0.03850259 | 0.26226593 |
| 7480 | NM_003394 WNT10B | | Wnt family member 10B | -5.981035 | 1.651010 | 0.003973214 | 0.054829606 |
| 10297 | NM_001351273APC2 | | APC2, WNT signaling pathway regulator | -5.949696 | 0.839294 | 0.004722384 | 0.062257821 |
| 768239 | NM_001085382PSAPL1 | | prosaposin-like 1 (gene/pseudogene) | -5.948621 | -2.587021 | 0.016636829 | 0.149104333 |
| 388662 | NM_001010898SLC6A17 | | solute carrier family 6 member 17 | -5.935398 | 3.175036 | 0.003724521 | 0.052196284 |
| 153020 | NM_001300735RASGEF1B | | RasGEF domain family member 1B | -5.933501 | -2.284003 | 0.012387782 | 0.124147898 |
| 4117 | NM_001242385MAK | | male germ cell associated kinase | -5.928427 | -0.740318 | 0.011980945 | 0.120714004 |
| 23397 | NM_001281710NCAPH | | non-SMC condensin I complex subunit H | -5.926370 | 5.945733 | 0.003599778 | 0.051642736 |
| 23590 | NM_001321978PDSS1 | | decaprenyl diphosphate synthase subunit 1 | -5.847112 | 3.809457 | 0.003931419 | 0.054468459 |
| 9833 | NM_001256685MELK | | maternal embryonic leucine zipper kinase | -5.833642 | 6.535118 | 0.003870574 | 0.053841847 |
| 2113 | NM_001143820ETS1 | | ETS proto-oncogene 1, transcription factor | -5.833096 | 5.862985 | 0.003877346 | 0.053899795 |
| 81610 | NM_030919 FAM83D | | family with sequence similarity 83 member D | -5.828868 | 6.178814 | 0.003891428 | 0.054022944 |
| 51659 | NM_016095 GINS2 | | GINS complex subunit 2 | -5.814764 | 4.966097 | 0.00397339 | 0.054829606 |
| 9212 | NM_001256834AURKB | | aurora kinase B | -5.812891 | 5.801036 | 0.003945401 | 0.054552613 |
| 332 | NM_001012270BIRC5 | | baculoviral IAP repeat containing 5 | -5.811040 | 7.084311 | 0.003936199 | 0.05449818 |
| 57556 | NM_001300780SEMA6A | | semaphorin 6A | -5.788394 | -1.913487 | 0.018523963 | 0.160995033 |
| 169044 | NM_152888 COL22A1 | | collagen type XXII alpha 1 chain | -5.774892 | -1.248212 | 0.009118805 | 0.09880696 |
| 84230 | NM_032270 LRRC8C | | leucine rich repeat containing 8 family member C | -5.755483 | 4.825211 | 0.004164176 | 0.056817935 |
| 83461 | NM_001297602CDCA3 | | cell division cycle associated 3 | -5.747790 | 5.260217 | 0.00420496 | 0.057223415 |
| 728361 | NM_001302757OVOL3 | | ovo like zinc finger 3 | -5.693499 | -3.006229 | 0.046195495 | 0.296479617 |
| 100526835 | NM_001112808FPGT-TNNI3K | | FPGT-TNNI3K readthrough | -5.691940 | -1.567106 | 0.01468201 | 0.140796188 |
| 767 | NM_001321837CA8 | | carbonic anhydrase 8 | -5.690226 | 5.105075 | 0.004419476 | 0.059400172 |
| 890 | NM_001237 CCNA2 | | cyclin A2 | -5.687299 | 6.106355 | 0.004366848 | 0.058960997 |
| 81624 | NM_001042517DIAPH3 | | diaphanous related formin 3 | -5.676747 | 5.032202 | 0.004468386 | 0.059785619 |
| 220134 | NM_001039535SKA1 | | spindle and kinetochore associated complex subunit 1 | -5.671293 | 4.653935 | 0.004512524 | 0.060142757 |
| 64131 | NM_022166 XYLT1 | | xylosyltransferase 1 | -5.651859 | 1.183612 | 0.005237611 | 0.066794073 |
| 4288 | NM_001145966MKI67 | | marker of proliferation Ki-67 | -5.643276 | 9.092521 | 0.004502014 | 0.060041371 |
| 221150 | NM_001166017SKA3 | | spindle and kinetochore associated complex subunit 3 | -5.643020 | 4.548186 | 0.004624859 | 0.061202582 |
| 57405 | NM_020675 SPC25 | | SPC25, NDC80 kinetochore complex component | -5.639949 | 4.138898 | 0.004666106 | 0.061633724 |
| 83903 | NM_031965 GSG2 | | germ cell associated 2, haspin | -5.639871 | 4.037355 | 0.004679473 | 0.061770831 |
| 7083 | NM_001346663TK1 | | thymidine kinase 1 | -5.630857 | 6.843880 | 0.004563538 | 0.060705064 |

| 84623 | NM_001161707KIRREL3 | kin of IRRE like 3 (Drosophila) | -5.626017 | 2.141409 | 0.004968885 | 0.063998367 |
| --- | --- | --- | --- | --- | --- | --- |
| 256158 | NM_001291815HMCN2 | hemicentin 2 | -5.605799 | -1.992591 | 0.013991958 | 0.1361353 |
| 55789 | NM_001145208DEPDC1B | DEP domain containing 1B | -5.588689 | 4.940562 | 0.004796996 | 0.062415821 |
| 100874296 | NR_046784 KCNH1-IT1 | KCNH1 intronic transcript 1 | -5.577611 | -2.253659 | 0.0262636 | 0.205382014 |
| 729522 | NR_024035,NR AACSP1 | acetoacetyl-CoA synthetase pseudogene 1 | -5.571159 | -2.009616 | 0.01601018 | 0.146302603 |
| 101927079 | NR_110480,NR LOC101927079 | uncharacterized LOC101927079 | -5.571159 | -1.511366 | 0.01601018 | 0.146302603 |
| 90139 | NM_130783 TSPAN18 | tetraspanin 18 | -5.567836 | 1.549159 | 0.006365978 | 0.076320644 |
| 344838 | NM_198504 PAQR9 | progestin and adipoQ receptor family member 9 | -5.555553 | 2.035789 | 0.005386786 | 0.068254845 |
| 51514 | NM_001286229DTL | denticleless E3 ubiquitin protein ligase homolog | -5.528343 | 5.800288 | 0.00498749 | 0.064198029 |
| 388121 | NM_001311175TNFAIP8L3 | TNF alpha induced protein 8 like 3 | -5.505051 | -0.097815 | 0.008433131 | 0.094530216 |
| 3694 | NM_000888,N ITGB6 | integrin subunit beta 6 | -5.494985 | 1.779417 | 0.005658165 | 0.070889853 |
| 2637 | NM_001301687GBX2 | gastrulation brain homeobox 2 | -5.477192 | 0.885459 | 0.00685928 | 0.080707743 |
| 2171 | NM_001444 FABP5 | fatty acid binding protein 5 | -5.472258 | 6.185685 | 0.005212965 | 0.066520778 |
| 6425 | NM_003015 SFRP5 | secreted frizzled related protein 5 | -5.450378 | -0.318620 | 0.017478245 | 0.154065215 |
| 151246 | NM_001160033SGO2 | shugoshin 2 | -5.447966 | 5.399014 | 0.005350492 | 0.067925788 |
| 55521 | NM_001017397TRIM36 | tripartite motif containing 36 | -5.433530 | 2.216097 | 0.005974516 | 0.074046048 |
| 55057 | NM_001039775CRYBG2 | crystallin beta-gamma domain containing 2 | -5.418004 | 1.389616 | 0.006115721 | 0.075434525 |
| 113130 | NM_080668 CDCA5 | cell division cycle associated 5 | -5.416092 | 6.169114 | 0.005469716 | 0.068988463 |
| 3604 | NM_001561 TNFRSF9 | TNF receptor superfamily member 9 | -5.413728 | 3.105427 | 0.005930396 | 0.073631597 |
| 55038 | NM_017955,N CDCA4 | cell division cycle associated 4 | -5.398594 | 5.740145 | 0.005556769 | 0.069877354 |
| 5347 | NM_005030 PLK1 | polo like kinase 1 | -5.395280 | 6.821054 | 0.005557082 | 0.069877354 |
| 57608 | NM_001350001KIAA1462 | KIAA1462 | -5.372831 | 4.801933 | 0.005707264 | 0.071203116 |
| 29893 | NM_001256014PSMC3IP | PSMC3 interacting protein | -5.371468 | 4.285268 | 0.005766434 | 0.071811371 |
| 2237 | NM_004111 FEN1 | flap structure-specific endonuclease 1 | -5.370337 | 6.652359 | 0.005678736 | 0.071061501 |
| 5979 | NM_020630,N RET | ret proto-oncogene | -5.366570 | -0.618369 | 0.010568105 | 0.110965098 |
| 80000 | NM_001142966GREB1L | growth regulation by estrogen in breast cancer 1 like | -5.352584 | 3.112589 | 0.006025678 | 0.074546137 |
| 2300 | NM_005250 FOXL1 | forkhead box L1 | -5.346865 | 1.027755 | 0.007140774 | 0.083450228 |
| 157570 | NM_001017420ESCO2 | establishment of sister chromatid cohesion N-acetyltransfer | -5.329538 | 5.247813 | 0.005925193 | 0.073611184 |
| 6289 | NM_001127380SAA2 | serum amyloid A2 | -5.307713 | 1.774948 | 0.025843036 | 0.203178717 |
| 389633 | NR_073395 FAM90A25P | family with sequence similarity 90 member A25, pseudogen | -5.307713 | -2.373512 | 0.025843036 | 0.203178717 |
| 105378732 | NR_131923 LINC01771 | long intergenic non-protein coding RNA 1771 | -5.307713 | -2.325022 | 0.025843036 | 0.203178717 |
| 161582 | NM_001033559DNAAF4 | dynein axonemal assembly factor 4 | -5.305587 | 2.430337 | 0.006460595 | 0.076980071 |
| 3481 | NM_000612,N IGF2 | insulin like growth factor 2 | -5.296863 | 2.411508 | 0.006445345 | 0.076926562 |
| 2878 | NM_001329790GPX3 | glutathione peroxidase 3 | -5.272121 | 3.904730 | 0.00634851 | 0.076320644 |
| 100128770 | NR_047572 LOC100128770 | uncharacterized LOC100128770 | -5.264601 | -3.257649 | 0.042028125 | 0.279264941 |
| 10468 | NM_006350,N FST | follistatin | -5.263831 | 2.901647 | 0.006408767 | 0.076583105 |
| 80714 | NM_025245,NRPBX4 | PBX homeobox 4 | -5.263089 | 0.050817 | 0.00946158 | 0.101774719 |
| 58538 | NM_033066 MPP4 | membrane palmitoylated protein 4 | -5.251323 | -2.587556 | 0.023000594 | 0.185556665 |
| 6586 | NM_001271946SLIT3 | slit guidance ligand 3 | -5.247358 | 5.512490 | 0.006327334 | 0.076320644 |
| 100499227 | NR_034160 USP2-AS1 | USP2 antisense RNA 1 (head to head) | -5.245915 | -1.218800 | 0.016730606 | 0.149620658 |
| 145508 | NM_152446 CEP128 | centrosomal protein 128 | -5.217556 | 3.789677 | 0.00670351 | 0.079190242 |
| 10112 | NM_005733 KIF20A | kinesin family member 20A | -5.210822 | 6.081246 | 0.006534596 | 0.077671618 |
| 55143 | NM_001256875CDCA8 | cell division cycle associated 8 | -5.203460 | 5.324069 | 0.006597161 | 0.078246718 |

| 2305 | NM_001243088FOXM1 forkhead box M1 | -5.189210 | 6.857811 | 0.00663849 | 0.078646714 |
| --- | --- | --- | --- | --- | --- |
| 23109 | NM_015086 DDN dendrin | -5.167918 | -0.127001 | 0.00991703 | 0.105684574 |
| 22974 | NM_012112 TPX2 TPX2, microtubule nucleation factor | -5.152013 | 7.829013 | 0.00684659 | 0.080604276 |
| 81831 | NM_001201477NETO2 neuropilin and tolloid like 2 | -5.142927 | 6.398766 | 0.006911494 | 0.08122969 |
| 9319 | NM_001166260TRIP13 thyroid hormone receptor interactor 13 | -5.096230 | 6.132811 | 0.007215559 | 0.08422903 |
| 140453 | NM_001040105MUC17 mucin 17, cell surface associated | -5.088036 | -2.453064 | 0.022901344 | 0.18519359 |
| 144455 | NM_203394 E2F7 E2F transcription factor 7 | -5.078641 | 4.619094 | 0.007416133 | 0.086205295 |
| 91624 | NM_001172309NEXN nexilin F-actin binding protein | -5.071794 | 1.837113 | 0.00799181 | 0.091523703 |
| 6941 | NM_001077511TCF19 transcription factor 19 | -5.062681 | 4.436334 | 0.007577823 | 0.087524778 |
| 157773 | NM_001007090C8orf48 chromosome 8 open reading frame 48 | -5.052075 | -1.733236 | 0.019453666 | 0.167317707 |
| 4953 | NM_001287188ODC1 ornithine decarboxylase 1 | -5.037059 | 7.965550 | 0.007575689 | 0.087524778 |
| 4440 | NM_002442 MSI1 musashi RNA binding protein 1 | -5.030894 | 0.772516 | 0.010065588 | 0.106937176 |
| 1869 | NM_005225 E2F1 E2F transcription factor 1 | -5.014448 | 5.283683 | 0.007788532 | 0.089552969 |
| 101928994 | NR_120648 LOC101928994 uncharacterized LOC101928994 | -5.003117 | -2.640710 | 0.038983062 | 0.263949145 |
| 388630 | NM_001194986TRABD2B TraB domain containing 2B | -5.000171 | -0.538812 | 0.011817586 | 0.119533867 |
| 6273 | NM_005978 S100A2 S100 calcium binding protein A2 | -4.988467 | 1.573413 | 0.008807763 | 0.096651761 |
| 11247 | NM_007224 NXPH4 neurexophilin 4 | -4.971889 | 1.251846 | 0.00988708 | 0.105419719 |
| 200315 | NM_001270406APOBEC3A apolipoprotein B mRNA editing enzyme catalytic subunit 3A | -4.960542 | -1.829651 | 0.022798603 | 0.184936901 |
| 401647 | NM_001010917GOLGA7B golgin A7 family member B | -4.951898 | 2.484972 | 0.008898241 | 0.09729953 |
| 84069 | NM_001160184PLEKHN1 pleckstrin homology domain containing N1 | -4.948552 | 2.121434 | 0.00889972 | 0.09729953 |
| 899 | NM_001323538CCNF cyclin F | -4.938687 | 5.438913 | 0.008339177 | 0.094530216 |
| 4173 | NM_005914,N MCM4 minichromosome maintenance complex component 4 | -4.934554 | 7.839776 | 0.008309565 | 0.094415666 |
| 146909 | NM_001264573KIF18B kinesin family member 18B | -4.918029 | 6.098596 | 0.008464111 | 0.094530216 |
| 3833 | NM_002263 KIFC1 kinesin family member C1 | -4.907099 | 5.580932 | 0.008573857 | 0.094586788 |
| 4603 | NM_001080416MYBL1 MYB proto-oncogene like 1 | -4.900732 | 4.816690 | 0.008688676 | 0.095649427 |
| 2048 | NM_001309192EPHB2 EPH receptor B2 | -4.891093 | 6.534525 | 0.008660723 | 0.095443295 |
| 84790 | NM_001303114TUBA1C tubulin alpha 1c | -4.888893 | 9.866282 | 0.008651522 | 0.095392717 |
| 144453 | NM_001282613BEST3 bestrophin 3 | -4.888809 | -0.120768 | 0.012606805 | 0.125371035 |
| 266727 | NM_153487 MDGA1 MAM domain containing glycosylphosphatidylinositol anch | -4.885886 | 2.513800 | 0.009174862 | 0.099258377 |
| 3437 | NM_001031683IFIT3 interferon induced protein with tetratricopeptide repeats 3 | -4.883407 | 4.015296 | 0.008865786 | 0.097082464 |
| 57821 | NM_001300968CCDC181 coiled-coil domain containing 181 | -4.881973 | -0.993368 | 0.014889348 | 0.142191661 |
| 7102 | NM_004615 TSPAN7 tetraspanin 7 | -4.879892 | 2.728420 | 0.009078836 | 0.098608124 |
| 11004 | NM_001297655KIF2C kinesin family member 2C | -4.859241 | 5.717647 | 0.008952959 | 0.097829872 |
| 100534595 | NR_037946 HNRNPUL2-BSCL HNRNPUL2-BSCL2 readthrough (NMD candidate) | -4.857246 | 4.052188 | 0.009088045 | 0.098608124 |
| 220963 | NM_001323977SLC16A9 solute carrier family 16 member 9 | -4.853340 | 2.684590 | 0.009483202 | 0.101901314 |
| 3400 | NM_001546 ID4 inhibitor of DNA binding 4, HLH protein | -4.848078 | 3.191118 | 0.009279377 | 0.100075038 |
| 23594 | NM_014321,NRORC6 origin recognition complex subunit 6 | -4.844108 | 4.736016 | 0.009157798 | 0.099125616 |
| 2646 | NM_001486 GCKR glucokinase regulator | -4.842658 | -0.276180 | 0.014340644 | 0.138227501 |
| 89795 | NM_001024383NAV3 neuron navigator 3 | -4.841161 | 5.269046 | 0.009112559 | 0.098791028 |
| 84617 | NM_001303524TUBB6 tubulin beta 6 class V | -4.837953 | 6.613641 | 0.009088823 | 0.098608124 |
| 1008 | NM_001317222CDH10 cadherin 10 | -4.827032 | -2.866554 | 0.036672434 | 0.252856432 |
| 4327 | NM_001272101MMP19 matrix metallopeptidase 19 | -4.827032 | -2.501177 | 0.036672434 | 0.252856432 |
| 23493 | NM_012259 HEY2 hes related family bHLH transcription factor with YRPW mo | -4.811619 | -2.899277 | 0.044697393 | 0.289577333 |

| 50615 | NM_021798,N | IL21R | interleukin 21 receptor | -4.811619 | -2.899277 | 0.044697393 | 0.289577333 |
| --- | --- | --- | --- | --- | --- | --- | --- |
| 79682 | NM_024629,NR | CENPU | centromere protein U | -4.810136 | 4.776816 | 0.009475376 | 0.101870141 |
| 64084 | NM_022131 | CLSTN2 | calsyntenin 2 | -4.809150 | -0.852156 | 0.018828856 | 0.162892044 |
| 114569 | NM_052886 | MAL2 | mal, T-cell differentiation protein 2 (gene/pseudogene) | -4.796476 | 0.429723 | 0.011575521 | 0.118301114 |
| 24137 | NM_012310 | KIF4A | kinesin family member 4A | -4.776694 | 6.237956 | 0.009634451 | 0.103044788 |
| 3014 | NM_002105 | H2AFX | H2A histone family member X | -4.774221 | 6.288162 | 0.009652785 | 0.103187525 |
| 147841 | NM_001317031SPC24 | | SPC24, NDC80 kinetochore complex component | -4.770149 | 3.836835 | 0.010013817 | 0.106551341 |
| 5004 | NM_000607 ORM1 | | orosomucoid 1 | -4.745456 | -2.377928 | 0.027046234 | 0.209375505 |
| 580 | NM_000465,N BARD1 | | BRCA1 associated RING domain 1 | -4.740675 | 4.078338 | 0.010147262 | 0.107473692 |
| 3399 | NM_002167 ID3 | | inhibitor of DNA binding 3, HLH protein | -4.737183 | 7.269457 | 0.009964079 | 0.106131297 |
| 25758 | NM_012194 KIAA1549L | | KIAA1549 like | -4.733922 | 5.261301 | 0.010060846 | 0.106937176 |
| 105369807 | NR_146529 LINC02454 | | long intergenic non-protein coding RNA 2454 | -4.725693 | -1.417367 | 0.030342931 | 0.227077973 |
| 10383 | NM_006088 TUBB4B | | tubulin beta 4B class IVb | -4.708549 | 9.214333 | 0.010219669 | 0.108019342 |
| 9645 | NM_001282663MICAL2 | | microtubule associated monooxygenase, calponin and LIM | -4.705933 | 5.404722 | 0.010316788 | 0.10887896 |
| 56776 | NM_001305424FMN2 | | formin 2 | -4.704381 | 2.742631 | 0.010775452 | 0.112741644 |
| 7153 | NM_001067 TOP2A | | topoisomerase (DNA) II alpha | -4.702076 | 9.571325 | 0.010281383 | 0.108616148 |
| 23649 | NM_002689 POLA2 | | DNA polymerase alpha 2, accessory subunit | -4.701471 | 5.274186 | 0.010379462 | 0.10942873 |
| 150142 | NR_027273,NR ZNF295-AS1 | | ZNF295 antisense RNA 1 | -4.700382 | -2.585992 | 0.034864293 | 0.243637802 |
| 144481 | NR_038263 SOCS2-AS1 | | SOCS2 antisense RNA 1 | -4.696420 | 0.218070 | 0.012683341 | 0.125890073 |
| 10376 | NM_006082 TUBA1B | | tubulin alpha 1b | -4.684055 | 10.753307 | 0.01045293 | 0.109980851 |
| 152007 | NM_001287010GLIPR2 | | GLI pathogenesis related 2 | -4.660935 | 2.377248 | 0.011664792 | 0.118301114 |
| 10403 | NM_006101 NDC80 | | NDC80, kinetochore complex component | -4.653523 | 4.118375 | 0.011050824 | 0.115098837 |
| 6383 | NM_002998 SDC2 | | syndecan 2 | -4.650860 | -0.883698 | 0.017765924 | 0.155847389 |
| 9787 | NM_001146015DLGAP5 | | DLG associated protein 5 | -4.643379 | 6.175022 | 0.010921817 | 0.114157551 |
| 8739 | NM_003806,NRHRK | | harakiri, BCL2 interacting protein | -4.637913 | -1.970946 | 0.029505815 | 0.222179751 |
| 113115 | NM_001099286MTFR2 | | mitochondrial fission regulator 2 | -4.636003 | 3.514283 | 0.011326313 | 0.117602386 |
| 5021 | NM_000916 OXTR | | oxytocin receptor | -4.635401 | 3.189014 | 0.011365228 | 0.117780435 |
| 2674 | NM_001145453GFRA1 | | GDNF family receptor alpha 1 | -4.618658 | 1.252873 | 0.012570255 | 0.125356208 |
| 89958 | NM_178448 SAPCD2 | | suppressor APC domain containing 2 | -4.613269 | 5.384137 | 0.011255113 | 0.116990964 |
| 57650 | NM_020890 KIAA1524 | | KIAA1524 | -4.595119 | 5.849554 | 0.011434917 | 0.118301114 |
| 5427 | NM_001197330POLE2 | | DNA polymerase epsilon 2, accessory subunit | -4.590560 | 3.835544 | 0.011774404 | 0.119205343 |
| 84176 | NR_002147 MYH16 | | myosin heavy chain 16 pseudogene | -4.589424 | -0.692927 | 0.019246127 | 0.165877557 |
| 3756 | NM_002238,N KCNH1 | | potassium voltage-gated channel subfamily H member 1 | -4.587632 | 1.696653 | 0.01241577 | 0.124368139 |
| 5569 | NM_006823,N PKIA | | cAMP-dependent protein kinase inhibitor alpha | -4.587015 | -0.516394 | 0.017024419 | 0.151462412 |
| 5865 | NM_002867 RAB3B | | RAB3B, member RAS oncogene family | -4.582643 | 5.843189 | 0.011547452 | 0.118301114 |
| 5905 | NM_001278651RANGAP1 | | Ran GTPase activating protein 1 | -4.579655 | 6.859842 | 0.011561463 | 0.118301114 |
| 55872 | NM_001278945PBK | | PDZ binding kinase | -4.575246 | 6.582438 | 0.011626277 | 0.118301114 |
| 5596 | NM_001292039MAPK4 | | mitogen-activated protein kinase 4 | -4.574319 | -0.666617 | 0.017518358 | 0.154264466 |
| 25903 | NM_001297713OLFML2B | | olfactomedin like 2B | -4.573692 | -0.513146 | 0.018600603 | 0.161457603 |
| 114794 | NM_052906,NRELFN2 | | extracellular leucine rich repeat and fibronectin type III dom | -4.570835 | 4.177008 | 0.011833385 | 0.119567935 |
| 9824 | NM_001286479ARHGAP11A | | Rho GTPase activating protein 11A | -4.567119 | 7.532917 | 0.011694388 | 0.118519553 |
| 7133 | NM_001066 TNFRSF1B | | TNF receptor superfamily member 1B | -4.562647 | 0.236619 | 0.038840397 | 0.263451779 |
| 4094 | NM_001031804MAF | | MAF bZIP transcription factor | -4.549381 | 0.060479 | 0.015837498 | 0.146302603 |

| 349667 | NM_178570 | RTN4RL2 | reticulon 4 receptor like 2 | -4.545615 | 5.982207 | 0.011954173 | 0.120561714 |
| --- | --- | --- | --- | --- | --- | --- | --- |
| 84915 | NM_032829 | FAM222A | family with sequence similarity 222 member A | -4.543325 | 2.485392 | 0.012787895 | 0.126684674 |
| 6790 | NM_001323303AURKA | | aurora kinase A | -4.540947 | 7.073905 | 0.012000196 | 0.120849097 |
| 874 | NM_001236 CBR3 | | carbonyl reductase 3 | -4.534697 | 3.067371 | 0.012547429 | 0.125356208 |
| 1870 | NM_004091 E2F2 | | E2F transcription factor 2 | -4.526928 | 3.689541 | 0.012557699 | 0.125356208 |
| 54962 | NM_001289986TIPIN | | TIMELESS interacting protein | -4.512095 | 4.415497 | 0.012532908 | 0.125298794 |
| 126961 | NM_021059,N HIST2H3C | | histone cluster 2 H3 family member c | -4.506577 | -2.447549 | 0.046781564 | 0.298715722 |
| 54478 | NM_001195228PIMREG | | PICALM interacting mitotic regulator | -4.506548 | 5.032373 | 0.01252397 | 0.125269978 |
| 10561 | NM_006417,NRIFI44 | | interferon induced protein 44 | -4.486522 | 1.644043 | 0.013782616 | 0.134478028 |
| 26032 | NM_015551 SUSD5 | | sushi domain containing 5 | -4.485416 | 1.994646 | 0.013528778 | 0.132438609 |
| 25786 | NR_024157 DGCR11 | | DiGeorge syndrome critical region gene 11 (non-protein co | -4.485185 | 0.225874 | 0.016976139 | 0.15116291 |
| 2786 | NM_001098721GNG4 | | G protein subunit gamma 4 | -4.479498 | 3.214362 | 0.013178661 | 0.129871658 |
| 4842 | NM_000620,N NOS1 | | nitric oxide synthase 1 | -4.470056 | 0.520173 | 0.015940858 | 0.146302603 |
| 983 | NM_001170406CDK1 | | cyclin dependent kinase 1 | -4.459088 | 7.087010 | 0.012983591 | 0.128377427 |
| 284040 | NM_001204477CDRT4 | | CMT1A duplicated region transcript 4 | -4.459082 | 2.479316 | 0.013660993 | 0.133435913 |
| 90381 | NM_001308025TICRR | | TOPBP1 interacting checkpoint and replication regulator | -4.453219 | 5.465968 | 0.01312551 | 0.129533006 |
| 3576 | NM_000584 CXCL8 | | C-X-C motif chemokine ligand 8 | -4.451204 | 7.252362 | 0.013110952 | 0.129512912 |
| 3161 | NM_001142556HMMR | | hyaluronan mediated motility receptor | -4.448691 | 6.421384 | 0.013142032 | 0.129634205 |
| 54984 | NM_001284356PINX1 | | PIN2/TERF1 interacting, telomerase inhibitor 1 | -4.445325 | 4.366105 | 0.013347351 | 0.13109684 |
| 54908 | NM_001329639SPDL1 | | spindle apparatus coiled-coil protein 1 | -4.442752 | 6.675370 | 0.013193016 | 0.129889357 |
| 478 | NM_001256213ATP1A3 | | ATPase Na+/K+ transporting subunit alpha 3 | -4.419278 | 2.316600 | 0.014482978 | 0.139339724 |
| 3394 | NM_002163 IRF8 | | interferon regulatory factor 8 | -4.418333 | -0.984934 | 0.020983402 | 0.177522156 |
| 2187 | NM_001018113FANCB | | Fanconi anemia complementation group B | -4.413567 | 2.910910 | 0.014242072 | 0.137724153 |
| 56675 | NM_020645 NRIP3 | | nuclear receptor interacting protein 3 | -4.404920 | 2.167620 | 0.01584977 | 0.146302603 |
| 151648 | NM_001012409SGO1 | | shugoshin 1 | -4.397679 | 4.546777 | 0.014030957 | 0.136450564 |
| 55055 | NM_001287821ZWILCH | | zwilch kinetochore protein | -4.389821 | 6.461568 | 0.013902949 | 0.135396657 |
| 162681 | NM_001288980C18orf54 | | chromosome 18 open reading frame 54 | -4.389214 | 3.400785 | 0.014501961 | 0.139457493 |
| 157739 | NR_001578 TDH | | L-threonine dehydrogenase (pseudogene) | -4.385464 | -1.736802 | 0.028512962 | 0.216914533 |
| 2316 | NM_001110556FLNA | | filamin A | -4.367487 | 11.081710 | 0.014159215 | 0.137053518 |
| 84057 | NM_001253861MND1 | | meiotic nuclear divisions 1 | -4.363926 | 3.329885 | 0.014837389 | 0.142017273 |
| 157983 | NM_152569 C9orf66 | | chromosome 9 open reading frame 66 | -4.363351 | -2.425062 | 0.040683222 | 0.272853118 |
| 348938 | NM_001099287NIPAL4 | | NIPA like domain containing 4 | -4.359022 | 2.248116 | 0.015554831 | 0.146302603 |
| 9837 | NM_021067,NRGINS1 | | GINS complex subunit 1 | -4.350966 | 6.191258 | 0.014452695 | 0.139113073 |
| 2675 | NM_001165038GFRA2 | | GDNF family receptor alpha 2 | -4.345800 | -1.703894 | 0.031048896 | 0.231107025 |
| 55215 | NM_001113378FANCI | | Fanconi anemia complementation group I | -4.338352 | 7.300916 | 0.014601518 | 0.140279145 |
| 57194 | NM_024490 ATP10A | | ATPase phospholipid transporting 10A (putative) | -4.334242 | 3.761804 | 0.014864051 | 0.142146502 |
| 9353 | NM_001289135SLIT2 | | slit guidance ligand 2 | -4.333286 | -1.865056 | 0.032799238 | 0.233707282 |
| 54898 | NM_017770 ELOVL2 | | ELOVL fatty acid elongase 2 | -4.326640 | 4.379051 | 0.014999327 | 0.143043371 |
| 57082 | NM_144508,N KNL1 | | kinetochore scaffold 1 | -4.322496 | 5.213143 | 0.014950685 | 0.142711083 |
| 9401 | NM_004260 RECQL4 | | RecQ like helicase 4 | -4.313431 | 5.661528 | 0.015021114 | 0.143119181 |
| 55771 | NM_018304 PRR11 | | proline rich 11 | -4.308286 | 6.862698 | 0.015050898 | 0.143270978 |
| 29028 | NM_014109 ATAD2 | | ATPase family, AAA domain containing 2 | -4.306664 | 7.017430 | 0.015067896 | 0.14330089 |
| 9700 | NM_012291 ESPL1 | | extra spindle pole bodies like 1, separase | -4.305275 | 6.102929 | 0.015124026 | 0.143737334 |

| 8787 | NM_001081955RGS9 | regulator of G protein signaling 9 | -4.304759 | -1.401773 | 0.027228959 | 0.210160825 |
| --- | --- | --- | --- | --- | --- | --- |
| 3956 | NM_002305 LGALS1 | galectin 1 | -4.299047 | 7.638893 | 0.015168077 | 0.143737334 |
| 9355 | NM_004789 LHX2 | LIM homeobox 2 | -4.297984 | 1.941741 | 0.016398509 | 0.147811798 |
| 84296 | NM_032336 GINS4 | GINS complex subunit 4 | -4.295636 | 5.491904 | 0.01530724 | 0.144646079 |
| 116936 | NR_000026 SNORD139 | small nucleolar RNA, C/D box 139 | -4.291477 | -0.491787 | 0.020913128 | 0.177072473 |
| 81704 | NM_001190458DOCK8 | dedicator of cytokinesis 8 | -4.289926 | 1.532843 | 0.016444689 | 0.14789495 |
| 79968 | NM_001167941WDR76 | WD repeat domain 76 | -4.288380 | 4.654038 | 0.015550788 | 0.146302603 |
| 6347 | NM_002982 CCL2 | C-C motif chemokine ligand 2 | -4.278367 | 5.499132 | 0.015599982 | 0.146302603 |
| 10036 | NM_005483 CHAF1A | chromatin assembly factor 1 subunit A | -4.273403 | 5.412058 | 0.015650681 | 0.146302603 |
| 8438 | NM_001142548RAD54L | RAD54 like (S. cerevisiae) | -4.249256 | 3.720287 | 0.016443748 | 0.14789495 |
| 677842 | NR_002995 SNORA50C | small nucleolar RNA, H/ACA box 50C | -4.247458 | -0.950456 | 0.028704807 | 0.217893183 |
| 2260 | NM_001174063FGFR1 | fibroblast growth factor receptor 1 | -4.242287 | 7.383575 | 0.01604832 | 0.146302603 |
| 51512 | NM_016426 GTSE1 | G2 and S-phase expressed 1 | -4.241203 | 5.351834 | 0.016174534 | 0.146382587 |
| 701 | NM_001211 BUB1B | BUB1 mitotic checkpoint serine/threonine kinase B | -4.236627 | 6.084959 | 0.016194963 | 0.146382587 |
| 27338 | NM_014501 UBE2S | ubiquitin conjugating enzyme E2 S | -4.235546 | 7.853373 | 0.016154303 | 0.146302603 |
| 7062 | NM_007113 TCHH | trichohyalin | -4.224803 | 0.698050 | 0.021182633 | 0.178768975 |
| 26153 | NM_015656 KIF26A | kinesin family member 26A | -4.224766 | 2.928898 | 0.017044281 | 0.151573928 |
| 118738 | NM_001346932ZNF488 | zinc finger protein 488 | -4.223389 | 3.265459 | 0.016961039 | 0.151093491 |
| 2919 | NM_001511,NRCXCL1 | C-X-C motif chemokine ligand 1 | -4.221981 | 7.330667 | 0.016460364 | 0.147971587 |
| 254268 | NM_152763,NRAKNAD1 | AKNA domain containing 1 | -4.206615 | -1.189677 | 0.030871457 | 0.230550528 |
| 9945 | NM_005110 GFPT2 | glutamine-fructose-6-phosphate transaminase 2 | -4.198201 | 6.175354 | 0.01684963 | 0.150478687 |
| 54892 | NM_001281932NCAPG2 | non-SMC condensin II complex subunit G2 | -4.187441 | 6.731954 | 0.016986946 | 0.151194053 |
| 7280 | NM_001069,N TUBB2A | tubulin beta 2A class IIa | -4.183972 | 5.052810 | 0.017119507 | 0.152112116 |
| 9493 | NM_001281301KIF23 | kinesin family member 23 | -4.177124 | 7.341849 | 0.017144422 | 0.152268084 |
| 114787 | NM_052899 GPRIN1 | G protein regulated inducer of neurite outgrowth 1 | -4.171885 | 4.271731 | 0.017561953 | 0.154582548 |
| 9586 | NM_001011666CREB5 | cAMP responsive element binding protein 5 | -4.163707 | 2.785990 | 0.018027358 | 0.157672681 |
| 3398 | NM_002166 ID2 | inhibitor of DNA binding 2, HLH protein | -4.162677 | 6.790262 | 0.017404473 | 0.153851081 |
| 642938 | NM_001039762FAM196A | family with sequence similarity 196 member A | -4.161753 | 1.389677 | 0.018744778 | 0.162436423 |
| 4174 | NM_006739 MCM5 | minichromosome maintenance complex component 5 | -4.160125 | 6.344219 | 0.017464515 | 0.154065215 |
| 3838 | NM_001320611KPNA2 | karyopherin subunit alpha 2 | -4.150494 | 9.062241 | 0.017586409 | 0.154731972 |
| 10228 | NM_001286210STX6 | syntaxin 6 | -4.146134 | 5.546178 | 0.017782815 | 0.15592943 |
| 11339 | NM_001317860OIP5 | Opa interacting protein 5 | -4.143875 | 3.195367 | 0.018374138 | 0.160096484 |
| 1821 | NM_001171184DRP2 | dystrophin related protein 2 | -4.130447 | 1.403454 | 0.020696002 | 0.175593441 |
| 84870 | NM_032784 RSPO3 | R-spondin 3 | -4.111711 | 7.167840 | 0.018308322 | 0.159792256 |
| 10721 | NM_199420 POLQ | DNA polymerase theta | -4.105588 | 5.472609 | 0.018523746 | 0.160995033 |
| 134285 | NM_001161342TMEM171 | transmembrane protein 171 | -4.102323 | -0.009561 | 0.025106413 | 0.198823185 |
| 4131 | NM_001324255MAP1B | microtubule associated protein 1B | -4.096055 | 8.931744 | 0.018585876 | 0.161397499 |
| 4085 | NM_002358 MAD2L1 | mitotic arrest deficient 2 like 1 | -4.094463 | 5.707467 | 0.018722575 | 0.162312013 |
| 4175 | NM_005915 MCM6 | minichromosome maintenance complex component 6 | -4.087281 | 6.879430 | 0.018787592 | 0.162739251 |
| 10105 | NM_005729 PPIF | peptidylprolyl isomerase F | -4.073581 | 6.723620 | 0.01905304 | 0.164350345 |
| 84953 | NM_032867 MICALCL | MICAL C-terminal like | -4.061180 | -0.605759 | 0.027927152 | 0.213715556 |
| 3008 | NM_005321 HIST1H1E | histone cluster 1 H1 family member e | -4.056798 | -1.446261 | 0.039579885 | 0.267202974 |
| 27074 | NM_014398 LAMP3 | lysosomal associated membrane protein 3 | -4.047132 | 0.621815 | 0.027147537 | 0.209767204 |

| 91687 | NM_001127181CENPL | centromere protein L | -4.046863 | 3.651300 | 0.020068347 | 0.171680039 |
| --- | --- | --- | --- | --- | --- | --- |
| 29089 | NM_001310326UBE2T | ubiquitin conjugating enzyme E2 T | -4.045402 | 5.658657 | 0.019681419 | 0.168995499 |
| 1503 | NM_001301237CTPS1 | CTP synthase 1 | -4.043894 | 6.997807 | 0.019632796 | 0.168682863 |
| 29127 | NM_001126103RACGAP1 | Rac GTPase activating protein 1 | -4.032484 | 6.952241 | 0.019877977 | 0.170400315 |
| 5271 | NM_001031848SERPINB8 | serpin family B member 8 | -4.028950 | 4.186271 | 0.020265159 | 0.172931031 |
| 8732 | NM_001286426RNGTT | RNA guanylyltransferase and 5'-phosphatase | -4.028779 | 5.101526 | 0.020077061 | 0.171680039 |
| 25886 | NM_001161580POC1A | POC1 centriolar protein A | -4.024975 | 4.101667 | 0.020422333 | 0.173612331 |
| 90417 | NM_001142761KNSTRN | kinetochore localized astrin/SPAG5 binding protein | -4.016811 | 5.274897 | 0.020310219 | 0.173101312 |
| 94122 | NM_001163334SYTL5 | synaptotagmin like 5 | -4.010239 | 0.848155 | 0.022864372 | 0.18519359 |
| 203068 | NM_001293212TUBB | tubulin beta class I | -4.009279 | 8.073974 | 0.020331816 | 0.173178001 |
| 145773 | NM_152450 FAM81A | family with sequence similarity 81 member A | -4.007822 | 3.800511 | 0.020899454 | 0.177072473 |
| 11130 | NM_001005413ZWINT | ZW10 interacting kinetochore protein | -4.006582 | 6.512650 | 0.020432318 | 0.173612331 |
| 84823 | NM_032737 LMNB2 | lamin B2 | -3.996337 | 7.523102 | 0.020614796 | 0.174976221 |
| 27113 | NM_001127240BBC3 | BCL2 binding component 3 | -3.989307 | 3.636208 | 0.021146006 | 0.178605611 |
| 10085 | NM_001278642EDIL3 | EGF like repeats and discoidin domains 3 | -3.980457 | -1.916828 | 0.044849939 | 0.290275655 |
| 60386 | NM_001126121SLC25A19 | solute carrier family 25 member 19 | -3.979688 | 3.886276 | 0.021366078 | 0.180243604 |
| 8986 | NM_001006944RPS6KA4 | ribosomal protein S6 kinase A4 | -3.973313 | 6.034013 | 0.021157922 | 0.178633309 |
| 135 | NM_000675,N ADORA2A | adenosine A2a receptor | -3.965737 | -1.336591 | 0.040195758 | 0.270654055 |
| 10272 | NM_005860 FSTL3 | follistatin like 3 | -3.961423 | 6.929220 | 0.021382083 | 0.180305089 |
| 7594 | NM_001256648ZNF43 | zinc finger protein 43 | -3.950563 | 0.911965 | 0.026321416 | 0.205533594 |
| 100131897 | NM_001129891FAM196B | family with sequence similarity 196 member B | -3.930181 | 1.594438 | 0.024182153 | 0.193130439 |
| 112885 | NM_001135862PHF21B | PHD finger protein 21B | -3.915352 | 1.633213 | 0.023967048 | 0.191823324 |
| 10460 | NM_006342 TACC3 | transforming acidic coiled-coil containing protein 3 | -3.913701 | 6.683953 | 0.022501751 | 0.184819135 |
| 8728 | NM_033274 ADAM19 | ADAM metallopeptidase domain 19 | -3.912974 | 4.179825 | 0.02287811 | 0.18519359 |
| 5570 | NM_001270393PKIB | cAMP-dependent protein kinase inhibitor beta | -3.904206 | 2.299380 | 0.023972119 | 0.191823324 |
| 101927267 | NR_120449 LOC101927267 | uncharacterized LOC101927267 | -3.900859 | -1.423251 | 0.042761799 | 0.282146036 |
| 3142 | NM_021958 HLX | H2.0 like homeobox | -3.900367 | 0.952686 | 0.02582313 | 0.203178717 |
| 55118 | NM_001206528CRTAC1 | cartilage acidic protein 1 | -3.899164 | -0.109435 | 0.044644611 | 0.289577333 |
| 2904 | NM_000834 GRIN2B | glutamate ionotropic receptor NMDA type subunit 2B | -3.898004 | -0.088113 | 0.02742938 | 0.210999154 |
| 84815 | NR_026880 MGC12916 | uncharacterized protein MGC12916 | -3.894934 | -2.151566 | 0.045915158 | 0.295505617 |
| 3975 | NM_005568 LHX1 | LIM homeobox 1 | -3.870520 | 2.066977 | 0.025738867 | 0.202976575 |
| 65989 | NM_001286655DLK2 | delta like non-canonical Notch ligand 2 | -3.865365 | 1.095764 | 0.028102779 | 0.214821134 |
| 9585 | NM_001284259KIF20B | kinesin family member 20B | -3.856343 | 6.190782 | 0.023936312 | 0.19175934 |
| 25791 | NM_001114090NGEF | neuronal guanine nucleotide exchange factor | -3.853511 | 3.875813 | 0.024472414 | 0.19477179 |
| 64105 | NM_001267038CENPK | centromere protein K | -3.852820 | 5.019708 | 0.024141014 | 0.192876353 |
| 9651 | NM_001303012PLCH2 | phospholipase C eta 2 | -3.850117 | -0.675466 | 0.032283042 | 0.233618934 |
| 78995 | NM_001171251C17orf53 | chromosome 17 open reading frame 53 | -3.849607 | 3.349598 | 0.024657864 | 0.196021491 |
| 55635 | NM_001114120DEPDC1 | DEP domain containing 1 | -3.846081 | 5.859977 | 0.024223245 | 0.193358211 |
| 100874048 | NR_104029,NR DGUOK-AS1 | DGUOK antisense RNA 1 | -3.837536 | 0.294563 | 0.029347003 | 0.221386859 |
| 182 | NM_000214 JAG1 | jagged 1 | -3.835530 | 7.373221 | 0.024396734 | 0.19439385 |
| 494143 | NM_001008708CHAC2 | ChaC cation transport regulator homolog 2 | -3.834844 | 3.714355 | 0.024904578 | 0.197588253 |
| 1948 | NM_004093 EFNB2 | ephrin B2 | -3.833234 | 3.024359 | 0.025748636 | 0.202976575 |
| 10256 | NM_001297647CNKSR1 | connector enhancer of kinase suppressor of Ras 1 | -3.829693 | -1.541791 | 0.047710877 | 0.298715722 |

| 124872 | NM_001159387B4GALNT2 | beta-1,4-N-acetyl-galactosaminyltransferase 2 | -3.829693 | -1.061461 | 0.047710877 | 0.298715722 |
| --- | --- | --- | --- | --- | --- | --- |
| 219736 | NM_001130159STOX1 | storkhead box 1 | -3.828843 | 0.590742 | 0.029485654 | 0.222179751 |
| 9055 | NM_001267580PRC1 | protein regulator of cytokinesis 1 | -3.828535 | 7.669700 | 0.024584639 | 0.195514515 |
| 80328 | NM_025217 ULBP2 | UL16 binding protein 2 | -3.828039 | 2.370979 | 0.026298264 | 0.205430373 |
| 124222 | NM_001284511PAQR4 | progestin and adipoQ receptor family member 4 | -3.822609 | 5.073859 | 0.024882882 | 0.197506679 |
| 51083 | NM_015973 GAL | galanin and GMAP prepropeptide | -3.818063 | 1.418780 | 0.028390857 | 0.216224183 |
| 692201 | NR_004399 SNORD86 | small nucleolar RNA, C/D box 86 | -3.815430 | 3.025872 | 0.025822661 | 0.203178717 |
| 891 | NM_031966 CCNB1 | cyclin B1 | -3.810219 | 7.510678 | 0.025077368 | 0.198669226 |
| 2150 | NM_005242 F2RL1 | F2R like trypsin receptor 1 | -3.808191 | 7.685042 | 0.025121709 | 0.198868177 |
| 100132891 | NR_033651,NR MSC-AS1 | MSC antisense RNA 1 | -3.800420 | 2.257479 | 0.026762341 | 0.207721962 |
| 2139 | NM_005244,N EYA2 | EYA transcriptional coactivator and phosphatase 2 | -3.798207 | 2.152816 | 0.027196672 | 0.209989979 |
| 2281 | NM_001322963FKBP1B | FK506 binding protein 1B | -3.789413 | 1.541653 | 0.027642734 | 0.212009624 |
| 4359 | NM_000530,N MPZ | myelin protein zero | -3.787772 | -1.174042 | 0.037849174 | 0.258984508 |
| 57580 | NM_020820 PREX1 | phosphatidylinositol-3,4,5-trisphosphate dependent Rac exc | -3.787709 | 4.304793 | 0.026017875 | 0.204390323 |
| 9021 | NM_003955 SOCS3 | suppressor of cytokine signaling 3 | -3.787291 | 5.738671 | 0.025767682 | 0.203049335 |
| 595 | NM_053056 CCND1 | cyclin D1 | -3.785280 | 8.963647 | 0.025720321 | 0.20293439 |
| 10675 | NM_001206942CSPG5 | chondroitin sulfate proteoglycan 5 | -3.783884 | 1.599025 | 0.029433127 | 0.221874723 |
| 29887 | NM_001199835SNX10 | sorting nexin 10 | -3.780963 | 3.832631 | 0.026439327 | 0.206220765 |
| 3619 | NM_001040694INCENP | inner centromere protein | -3.776945 | 6.364597 | 0.026026788 | 0.204390323 |
| 2894 | NM_017551 GRID1 | glutamate ionotropic receptor delta type subunit 1 | -3.770939 | 0.337791 | 0.031210166 | 0.231973514 |
| 5558 | NM_000947,N PRIM2 | primase (DNA) subunit 2 | -3.770896 | 4.006180 | 0.026672248 | 0.207532496 |
| 26579 | NM_001293291MYEOV | myeloma overexpressed | -3.769336 | 6.283486 | 0.026230949 | 0.205382014 |
| 2247 | NM_002006 FGF2 | fibroblast growth factor 2 | -3.768286 | 6.784262 | 0.026227148 | 0.205382014 |
| 347733 | NM_178012 TUBB2B | tubulin beta 2B class IIb | -3.767737 | 4.546314 | 0.026484252 | 0.206493309 |
| 11169 | NM_001008396WDHD1 | WD repeat and HMG-box DNA binding protein 1 | -3.765149 | 5.620245 | 0.026414374 | 0.206103857 |
| 1062 | NM_001286734CENPE | centromere protein E | -3.757325 | 5.969687 | 0.02661858 | 0.207462442 |
| 10615 | NM_006461 SPAG5 | sperm associated antigen 5 | -3.753827 | 7.127733 | 0.026646099 | 0.207532496 |
| 898 | NM_001238,N CCNE1 | cyclin E1 | -3.746247 | 5.286862 | 0.026975708 | 0.209050991 |
| 101927934 | NR_105045 LINC01843 | long intergenic non-protein coding RNA 1843 | -3.734382 | 0.377158 | 0.033828855 | 0.238496884 |
| 23541 | NM_001204204SEC14L2 | SEC14 like lipid binding 2 | -3.731318 | 3.911820 | 0.027673176 | 0.212164435 |
| 7128 | NM_001270507TNFAIP3 | TNF alpha induced protein 3 | -3.729953 | 5.865828 | 0.027440695 | 0.211007723 |
| 3429 | NM_001130080IFI27 | interferon alpha inducible protein 27 | -3.726247 | 0.651806 | 0.033364974 | 0.236516276 |
| 56992 | NM_020242 KIF15 | kinesin family member 15 | -3.723302 | 4.531501 | 0.027902088 | 0.213602773 |
| 9928 | NM_001305792KIF14 | kinesin family member 14 | -3.722575 | 5.998163 | 0.027633578 | 0.212009624 |
| 79605 | NM_001258311PGBD5 | piggyBac transposable element derived 5 | -3.719782 | -0.920395 | 0.049165447 | 0.302855055 |
| 10046 | NM_001177465MAMLD1 | mastermind like domain containing 1 | -3.710646 | 3.968558 | 0.028328663 | 0.21584601 |
| 7368 | NM_001128174UGT8 | UDP glycosyltransferase 8 | -3.706516 | 0.084211 | 0.036656632 | 0.252856432 |
| 3159 | NM_001319077HMGA1 | high mobility group AT-hook 1 | -3.702581 | 9.168957 | 0.028117876 | 0.214857137 |
| 4171 | NM_004526,NRMCM2 | minichromosome maintenance complex component 2 | -3.698987 | 6.609146 | 0.028291597 | 0.215706481 |
| 5982 | NM_001278791RFC2 | replication factor C subunit 2 | -3.697969 | 5.468537 | 0.028404344 | 0.216247276 |
| 558 | NM_001278599AXL | AXL receptor tyrosine kinase | -3.688850 | 7.915151 | 0.028555275 | 0.217156569 |
| 9133 | NM_004701 CCNB2 | cyclin B2 | -3.685085 | 6.170648 | 0.028776298 | 0.218115693 |
| 641 | NM_000057,N BLM | Bloom syndrome RecQ like helicase | -3.683200 | 5.327348 | 0.028909511 | 0.218964935 |

| 7781 | NM_001318949SLC30A3 | solute carrier family 30 member 3 | -3.673437 | 1.518055 | 0.032488562 | 0.233618934 |
| --- | --- | --- | --- | --- | --- | --- |
| 440288 | NR_038448,NR UBL7-AS1 | UBL7 antisense RNA 1 (head to head) | -3.672880 | 1.326699 | 0.033056287 | 0.235133869 |
| 79172 | NM_001199803CENPO | centromere protein O | -3.672043 | 5.894146 | 0.029179228 | 0.22052332 |
| 8632 | NM_173628 DNAH17 | dynein axonemal heavy chain 17 | -3.671822 | 0.770430 | 0.034151697 | 0.24019988 |
| 100130967 | NM_001195032C6orf99 | chromosome 6 open reading frame 99 | -3.667707 | -0.548354 | 0.040333044 | 0.271313504 |
| 2977 | NM_000855,N GUCY1A2 | guanylate cyclase 1 soluble subunit alpha 2 | -3.663018 | -1.604415 | 0.046505816 | 0.29800892 |
| 10849 | NM_001297590CD3EAP | CD3e molecule associated protein | -3.660000 | 5.627846 | 0.029566944 | 0.222397177 |
| 3320 | NM_001017963HSP90AA1 | heat shock protein 90 alpha family class A member 1 | -3.658107 | 11.339072 | 0.029502108 | 0.222179751 |
| 127343 | NM_147192,N DMBX1 | diencephalon/mesencephalon homeobox 1 | -3.653784 | 0.893064 | 0.034857966 | 0.243637802 |
| 374378 | NM_198516 GALNT18 | polypeptide N-acetylgalactosaminyltransferase 18 | -3.648309 | 4.205554 | 0.030322157 | 0.227004639 |
| 146429 | NM_001242757SLC22A31 | solute carrier family 22 member 31 | -3.644017 | 0.898391 | 0.033239873 | 0.235931188 |
| 57057 | NM_001077653TBX20 | T-box 20 | -3.639094 | 0.956547 | 0.03428318 | 0.240960783 |
| 2921 | NM_002090 CXCL3 | C-X-C motif chemokine ligand 3 | -3.625701 | 5.330108 | 0.030949917 | 0.230550528 |
| 3139 | NR_027822 HLA-L | major histocompatibility complex, class I, L (pseudogene) | -3.617649 | 0.075492 | 0.040229074 | 0.27074664 |
| 284119 | NM_012232 CAVIN1 | caveolae associated protein 1 | -3.614691 | 8.541008 | 0.03095519 | 0.230550528 |
| 6525 | NM_001207017SMTN | smoothelin | -3.609321 | 5.434240 | 0.031280081 | 0.232326205 |
| 100289092 | NR_046287,NR ATP2A1-AS1 | ATP2A1 antisense RNA 1 | -3.608186 | 0.660862 | 0.037530079 | 0.257056186 |
| 374 | NM_001657,N AREG | amphiregulin | -3.604217 | 5.699522 | 0.031449905 | 0.233336187 |
| 3131 | NM_001330375HLF | HLF, PAR bZIP transcription factor | -3.602044 | 1.457733 | 0.033205708 | 0.235855103 |
| 147 | NM_000679 ADRA1B | adrenoceptor alpha 1B | -3.592172 | 2.344345 | 0.033077636 | 0.235204846 |
| 6385 | NM_002999 SDC4 | syndecan 4 | -3.588237 | 6.710664 | 0.031944026 | 0.233618934 |
| 5137 | NM_001191056PDE1C | phosphodiesterase 1C | -3.588135 | 3.929051 | 0.03219268 | 0.233618934 |
| 9712 | NM_001080491USP6NL | USP6 N-terminal like | -3.569465 | 5.014042 | 0.032816363 | 0.233748784 |
| 56938 | NM_001248002ARNTL2 | aryl hydrocarbon receptor nuclear translocator like 2 | -3.566507 | 6.238878 | 0.032740065 | 0.233618934 |
| 387103 | NM_001012507CENPW | centromere protein W | -3.563783 | 2.751264 | 0.034576716 | 0.242365086 |
| 10733 | NM_001190799PLK4 | polo like kinase 4 | -3.556678 | 4.938986 | 0.033272599 | 0.235991372 |
| 23057 | NM_015039,N NMNAT2 | nicotinamide nucleotide adenylyltransferase 2 | -3.552434 | 2.242482 | 0.034500653 | 0.242078019 |
| 1959 | NM_000399,N EGR2 | early growth response 2 | -3.551831 | 1.535265 | 0.035243273 | 0.246036821 |
| 7516 | NM_005431 XRCC2 | X-ray repair cross complementing 2 | -3.543109 | 4.428231 | 0.033939569 | 0.239033023 |
| 23462 | NM_001040708HEY1 | hes related family bHLH transcription factor with YRPW mo | -3.536978 | 0.315354 | 0.041209107 | 0.275771719 |
| 23246 | NM_015201 BOP1 | block of proliferation 1 | -3.536884 | 6.665623 | 0.033783208 | 0.238337535 |
| 8208 | NM_005441 CHAF1B | chromatin assembly factor 1 subunit B | -3.525444 | 4.892922 | 0.034434502 | 0.241695853 |
| 154 | NM_000024 ADRB2 | adrenoceptor beta 2 | -3.519279 | 0.507900 | 0.043021329 | 0.28313592 |
| 9510 | NM_006988 ADAMTS1 | ADAM metallopeptidase with thrombospondin type 1 motif | -3.519279 | 0.301963 | 0.043021329 | 0.28313592 |
| 55839 | NM_001100624CENPN | centromere protein N | -3.518417 | 6.006827 | 0.034557817 | 0.242365086 |
| 90627 | NM_001243466STARD13 | StAR related lipid transfer domain containing 13 | -3.506921 | 3.280646 | 0.035554532 | 0.24770815 |
| 8727 | NM_001286974CTNNAL1 | catenin alpha like 1 | -3.502166 | 6.220313 | 0.03516468 | 0.245653973 |
| 78990 | NM_023112 OTUB2 | OTU deubiquitinase, ubiquitin aldehyde binding 2 | -3.498622 | 4.057385 | 0.035716922 | 0.248504718 |
| 7015 | NM_001193376TERT | telomerase reverse transcriptase | -3.495875 | 1.808463 | 0.03794923 | 0.259583275 |
| 672 | NM_007294,N BRCA1 | BRCA1, DNA repair associated | -3.486480 | 6.452143 | 0.035775708 | 0.248746391 |
| 8520 | NM_003642,NRHAT1 | histone acetyltransferase 1 | -3.486303 | 6.117271 | 0.03579649 | 0.248807257 |
| 23645 | NM_014330 PPP1R15A | protein phosphatase 1 regulatory subunit 15A | -3.485548 | 7.066783 | 0.035753813 | 0.248677745 |
| 547 | NM_001244008KIF1A | kinesin family member 1A | -3.482309 | 0.985150 | 0.042705869 | 0.281956877 |

| 64946 | NM_022909 | CENPH | centromere protein H | -3.478542 | 4.719879 | 0.036467218 | 0.252282412 |
| --- | --- | --- | --- | --- | --- | --- | --- |
| 2571 | NM_000817,N | GAD1 | glutamate decarboxylase 1 | -3.469538 | -0.187220 | 0.047102154 | 0.298715722 |
| 347240 | NM_194313 | KIF24 | kinesin family member 24 | -3.465533 | 3.756694 | 0.037121969 | 0.255020898 |
| 10855 | NM_001098540HPSE | | heparanase | -3.464206 | 1.742339 | 0.039285377 | 0.265822056 |
| 56937 | NM_001255976PMEPA1 | | prostate transmembrane protein, androgen induced 1 | -3.462529 | 8.327827 | 0.036667755 | 0.252856432 |
| 1058 | NM_001042426CENPA | | centromere protein A | -3.454146 | 4.727254 | 0.037387796 | 0.256251343 |
| 51203 | NM_001243142NUSAP1 | | nucleolar and spindle associated protein 1 | -3.451654 | 6.606817 | 0.037212325 | 0.255387177 |
| 57120 | NM_001017408GOPC | | golgi associated PDZ and coiled-coil motif containing | -3.450059 | 5.891670 | 0.037302117 | 0.25575771 |
| 5933 | NM_001323281RBL1 | | RB transcriptional corepressor like 1 | -3.447325 | 5.454591 | 0.037474614 | 0.256761311 |
| 7070 | NM_001311160THY1 | | Thy-1 cell surface antigen | -3.443898 | 1.361396 | 0.043806106 | 0.286387263 |
| 1839 | NM_001945 HBEGF | | heparin binding EGF like growth factor | -3.438397 | 3.013052 | 0.03874704 | 0.263299121 |
| 79019 | NM_001002876CENPM | | centromere protein M | -3.425984 | 3.169932 | 0.039315702 | 0.265940255 |
| 24147 | NM_014344 FJX1 | | four jointed box 1 | -3.425355 | 4.402047 | 0.038568617 | 0.262492711 |
| 144501 | NM_001081492KRT80 | | keratin 80 | -3.416089 | 6.365932 | 0.038716812 | 0.263240845 |
| 7298 | NM_001071 TYMS | | thymidylate synthetase | -3.411428 | 6.855261 | 0.038902852 | 0.263664974 |
| 283551 | NR_126496,NR LINC01588 | | long intergenic non-protein coding RNA 1588 | -3.408644 | 2.575369 | 0.040483653 | 0.272061197 |
| 9099 | NM_001243759USP2 | | ubiquitin specific peptidase 2 | -3.405135 | -0.008071 | 0.046776362 | 0.298715722 |
| 84665 | NM_001256267MYPN | | myopalladin | -3.396951 | 1.518366 | 0.042282839 | 0.280291436 |
| 100996928 | NM_001244584C7orf55-LUC7L2 | | C7orf55-LUC7L2 readthrough | -3.396835 | 2.195382 | 0.041321531 | 0.276345251 |
| 55084 | NM_018013 SOBP | | sine oculis binding protein homolog | -3.396506 | 1.363784 | 0.042601539 | 0.281537647 |
| 1017 | NM_001290230CDK2 | | cyclin dependent kinase 2 | -3.389868 | 6.095150 | 0.039933435 | 0.269238298 |
| 151176 | NM_001291832ERFE | | erythroferrone | -3.388179 | 1.645229 | 0.042473991 | 0.281015561 |
| 3832 | NM_004523 KIF11 | | kinesin family member 11 | -3.376771 | 7.081803 | 0.040465233 | 0.272061197 |
| 100302736 | NM_001164468TMED7-TICAM2 | | TMED7-TICAM2 readthrough | -3.368017 | 3.465823 | 0.04157216 | 0.277304134 |
| 2037 | NM_001135554EPB41L2 | | erythrocyte membrane protein band 4.1 like 2 | -3.363432 | 6.513905 | 0.041111359 | 0.27520662 |
| 51422 | NM_001040633PRKAG2 | | protein kinase AMP-activated non-catalytic subunit gamma | -3.362273 | 5.280926 | 0.041256797 | 0.276001565 |
| 55698 | NM_018059 RADIL | | Ras association and DIL domains | -3.362128 | 2.170982 | 0.043673746 | 0.285883368 |
| 80178 | NM_025108 C16orf59 | | chromosome 16 open reading frame 59 | -3.356602 | 3.839038 | 0.042068424 | 0.279442953 |
| 4751 | NM_001204182NEK2 | | NIMA related kinase 2 | -3.355051 | 5.792046 | 0.041635848 | 0.277639432 |
| 200916 | NM_001099645RPL22L1 | | ribosomal protein L22 like 1 | -3.348024 | 5.835273 | 0.041872443 | 0.278678086 |
| 5985 | NM_001130112RFC5 | | replication factor C subunit 5 | -3.346332 | 5.946256 | 0.041994551 | 0.279264941 |
| 2669 | NM_005261,N GEM | | GTP binding protein overexpressed in skeletal muscle | -3.337445 | 3.615261 | 0.042875822 | 0.282624206 |
| 100128191 | NR_027157 TMPO-AS1 | | TMPO antisense RNA 1 | -3.335135 | 3.061618 | 0.043900348 | 0.286912701 |
| 5270 | NM_001136528SERPINE2 | | serpin family E member 2 | -3.333844 | 6.459391 | 0.042540327 | 0.281312875 |
| 654342 | NR_027238 LOC654342 | | lymphocyte-specific protein 1 pseudogene | -3.332749 | 2.671731 | 0.043772646 | 0.286349519 |
| 7739 | NM_001178106ZNF185 | | zinc finger protein 185 with LIM domain | -3.329225 | 5.618882 | 0.04282416 | 0.282377349 |
| 4940 | NM_006187 OAS3 | | 2'-5'-oligoadenylate synthetase 3 | -3.325764 | 7.092928 | 0.042888924 | 0.282624206 |
| 6615 | NM_005985 SNAI1 | | snail family transcriptional repressor 1 | -3.317200 | 2.499473 | 0.045158197 | 0.291634403 |
| 1434 | NM_001256135CSE1L | | chromosome segregation 1 like | -3.309909 | 8.048578 | 0.043647547 | 0.285802312 |
| 128239 | NM_178229 IQGAP3 | | IQ motif containing GTPase activating protein 3 | -3.309015 | 6.161330 | 0.043795327 | 0.286387263 |
| 404550 | NM_206967 C16orf74 | | chromosome 16 open reading frame 74 | -3.308586 | 2.646893 | 0.045299447 | 0.292361645 |
| 259232 | NM_001350748NALCN | | sodium leak channel, non-selective | -3.304930 | 3.556796 | 0.044672464 | 0.289577333 |
| 55210 | NM_001170535ATAD3A | | ATPase family, AAA domain containing 3A | -3.301362 | 5.143058 | 0.044275876 | 0.28868718 |

| 3778 | NM_001014797KCNMA1 | potassium calcium-activated channel subfamily M alpha 1 | -3.297690 | 4.549379 | 0.044700045 | 0.289577333 |
| --- | --- | --- | --- | --- | --- | --- |
| 1468 | NM_001270888SLC25A10 | solute carrier family 25 member 10 | -3.295768 | 5.131333 | 0.044628578 | 0.289577333 |
| 8835 | NM_001270467SOCS2 | suppressor of cytokine signaling 2 | -3.288358 | 4.237288 | 0.045158569 | 0.291634403 |
| 9768 | NM_001029989PCLAF | PCNA clamp associated factor | -3.285142 | 5.948867 | 0.045059593 | 0.291177032 |
| 1893 | NM_001202858ECM1 | extracellular matrix protein 1 | -3.280858 | 2.092297 | 0.047524972 | 0.298715722 |
| 9688 | NM_001242795NUP93 | nucleoporin 93 | -3.274762 | 6.425077 | 0.045535151 | 0.293608042 |
| 3099 | NM_000189 HK2 | hexokinase 2 | -3.273622 | 2.706402 | 0.047169006 | 0.298715722 |
| 7378 | NM_001287426UPP1 | uridine phosphorylase 1 | -3.268756 | 4.529059 | 0.046161481 | 0.296479617 |
| 7272 | NM_001166691TTK | TTK protein kinase | -3.268143 | 5.353052 | 0.046045906 | 0.296162803 |
| 100874032 | NR_046734 PRRT3-AS1 | PRRT3 antisense RNA 1 | -3.266202 | 1.358816 | 0.048875207 | 0.30169611 |
| 91010 | NM_175736,N FMNL3 | formin like 3 | -3.259478 | 2.703957 | 0.048037341 | 0.298715722 |
| 51666 | NM_016116,N ASB4 | ankyrin repeat and SOCS box containing 4 | -3.258411 | 3.102428 | 0.047367972 | 0.298715722 |
| 22809 | NM_001193646ATF5 | activating transcription factor 5 | -3.257285 | 5.429494 | 0.04654377 | 0.298159764 |
| 26586 | NM_001098525CKAP2 | cytoskeleton associated protein 2 | -3.253948 | 6.318630 | 0.046669193 | 0.298715722 |
| 3589 | NM_000641,N IL11 | interleukin 11 | -3.250179 | 5.536610 | 0.04687076 | 0.298715722 |
| 1033 | NM_001130851CDKN3 | cyclin dependent kinase inhibitor 3 | -3.246820 | 5.355857 | 0.047256536 | 0.298715722 |
| 10024 | NM_001100620TROAP | trophinin associated protein | -3.240671 | 5.298506 | 0.047549447 | 0.298715722 |
| 1649 | NM_001195053DDIT3 | DNA damage inducible transcript 3 | -3.227478 | 4.462101 | 0.048300136 | 0.298998884 |
| 6781 | NM_003155 STC1 | stanniocalcin 1 | -3.223010 | 5.695668 | 0.048454208 | 0.29963387 |
| 55722 | NM_018140 CEP72 | centrosomal protein 72 | -3.222667 | 3.728775 | 0.049116273 | 0.302855055 |
| 2175 | NM_000135,N FANCA | Fanconi anemia complementation group A | -3.221321 | 5.541486 | 0.048556902 | 0.300089488 |
| 388962 | NM_001035505BOLA3 | bolA family member 3 | -3.220268 | 5.101844 | 0.048697305 | 0.300777475 |
| 4277 | NM_001289160MICB | MHC class I polypeptide-related sequence B | -3.216009 | 4.327474 | 0.049295114 | 0.303473045 |
| 1809 | NM_001197294DPYSL3 | dihydropyrimidinase like 3 | -3.215229 | 5.186702 | 0.048976947 | 0.302143793 |
| 6835 | NM_001278928SURF2 | surfeit 2 | -3.214597 | 3.919889 | 0.049417109 | 0.303914694 |
| 10635 | NM_001130862RAD51AP1 | RAD51 associated protein 1 | -3.214507 | 4.762776 | 0.04916331 | 0.302855055 |
| 7262 | NM_003311 PHLDA2 | pleckstrin homology like domain family A member 2 | -3.214030 | 4.277743 | 0.049159573 | 0.302855055 |
| 113146 | NM_001350929AHNAK2 | AHNAK nucleoprotein 2 | -3.212373 | 9.353095 | 0.04885921 | 0.301687391 |
| 140707 | NM_080626 BRI3BP | BRI3 binding protein | -3.209482 | 4.458198 | 0.049469514 | 0.304094172 |
| 64651 | NM_001320559CSRNP1 | cysteine and serine rich nuclear protein 1 | -3.206987 | 5.257212 | 0.049359107 | 0.30377659 |
| 2177 | NM_001018115FANCD2 | Fanconi anemia complementation group D2 | -3.197457 | 5.646062 | 0.049943385 | 0.306369785 |
| 10236 | NM_001102397HNRNPR | heterogeneous nuclear ribonucleoprotein R | -3.195362 | 7.873847 | 0.049877399 | 0.306055771 |
| 4326 | NM_016155 MMP17 | matrix metallopeptidase 17 | 3.204858 | 4.116297 | 0.049752865 | 0.305654297 |
| 55204 | NM_018178 GOLPH3L | golgi phosphoprotein 3 like | 3.209685 | 5.861967 | 0.04914406 | 0.302855055 |
| 9311 | NM_004769,N ASIC3 | acid sensing ion channel subunit 3 | 3.212111 | 3.573067 | 0.049524473 | 0.304341567 |
| 79094 | NM_001142776CHAC1 | ChaC glutathione specific gamma-glutamylcyclotransferase | 3.213767 | 3.475564 | 0.049425624 | 0.303914694 |
| 8828 | NM_003872,N NRP2 | neuropilin 2 | 3.219092 | 7.592386 | 0.048516246 | 0.299927838 |
| 84706 | NM_001142466GPT2 | glutamic--pyruvic transaminase 2 | 3.228114 | 5.873270 | 0.048087646 | 0.298715722 |
| 8759 | NR_036636 ADAM1A | ADAM metallopeptidase domain 1A (pseudogene) | 3.229303 | 2.265725 | 0.049390517 | 0.303879487 |
| 79815 | NM_001321635NIPAL2 | NIPA like domain containing 2 | 3.229491 | 3.950711 | 0.048397438 | 0.299461862 |
| 9266 | NM_004228,N CYTH2 | cytohesin 2 | 3.232988 | 7.274001 | 0.047730219 | 0.298715722 |
| 80017 | NM_001102366C14orf159 | chromosome 14 open reading frame 159 | 3.234347 | 4.816848 | 0.047938552 | 0.298715722 |
| 6662 | NM_000346 SOX9 | SRY-box 9 | 3.241663 | 5.636937 | 0.047353236 | 0.298715722 |

| 283970 | NR_003610 PDXDC2P-NPIPB1 | nuclear pore complex-interacting protein | 3.243001 | 4.784294 | 0.047395125 | 0.298715722 |
| --- | --- | --- | --- | --- | --- | --- |
| 6303 | NM_002970,NRSAT1 | spermidine/spermine N1-acetyltransferase 1 | 3.243266 | 9.090851 | 0.047129071 | 0.298715722 |
| 56204 | NM_001286495FAM214A | family with sequence similarity 214 member A | 3.247129 | 5.319115 | 0.047087456 | 0.298715722 |
| 89953 | NM_001289034KLC4 | kinesin light chain 4 | 3.254424 | 3.816581 | 0.047008654 | 0.298715722 |
| 84874 | NM_001318005ZNF514 | zinc finger protein 514 | 3.255395 | 5.119713 | 0.046680482 | 0.298715722 |
| 64220 | NM_001142617STRA6 | stimulated by retinoic acid 6 | 3.261568 | 6.202080 | 0.046272325 | 0.296880598 |
| 8742 | NM_003809,NRTNFSF12 | TNF superfamily member 12 | 3.262214 | 3.496323 | 0.046758184 | 0.298715722 |
| 100532746 | NR_037861 PPT2-EGFL8 | PPT2-EGFL8 readthrough (NMD candidate) | 3.265819 | 2.077637 | 0.048022253 | 0.298715722 |
| 254896 | NR_046173 LOC254896 | uncharacterized LOC254896 | 3.268099 | 1.798081 | 0.048255242 | 0.298850203 |
| 4082 | NM_002356 MARCKS | myristoylated alanine rich protein kinase C substrate | 3.273389 | 7.365207 | 0.0455494 | 0.293608392 |
| 54407 | NM_001307936SLC38A2 | solute carrier family 38 member 2 | 3.273527 | 9.658817 | 0.045493529 | 0.293431136 |
| 80127 | NM_025057 BBOF1 | basal body orientation factor 1 | 3.276970 | 2.430608 | 0.046822404 | 0.298715722 |
| 27239 | NM_014449,N GPR162 | G protein-coupled receptor 162 | 3.279241 | 1.417919 | 0.049832815 | 0.305872931 |
| 197257 | NM_153486,N LDHD | lactate dehydrogenase D | 3.284356 | 1.238141 | 0.048914543 | 0.301848846 |
| 440270 | NM_001023567GOLGA8B | golgin A8 family member B | 3.289979 | 7.530711 | 0.044662304 | 0.289577333 |
| 100506686 | NR_040051,NR IQCH-AS1 | IQCH antisense RNA 1 | 3.290548 | 3.265907 | 0.04540634 | 0.292960119 |
| 130872 | NM_001321300AHSA2 | activator of HSP90 ATPase homolog 2 | 3.294221 | 7.326415 | 0.044450917 | 0.289577333 |
| 1958 | NM_001964 EGR1 | early growth response 1 | 3.297335 | 7.608216 | 0.044283511 | 0.28868718 |
| 8436 | NM_004657 CAVIN2 | caveolae associated protein 2 | 3.298638 | 4.695891 | 0.044527895 | 0.289577333 |
| 658 | NM_001203,N BMPR1B | bone morphogenetic protein receptor type 1B | 3.300477 | 5.523151 | 0.044232425 | 0.288627038 |
| 132671 | NM_001297608SPATA18 | spermatogenesis associated 18 | 3.304056 | 3.657512 | 0.044513233 | 0.289577333 |
| 1742 | NM_001128827DLG4 | discs large MAGUK scaffold protein 4 | 3.304286 | 4.774607 | 0.044173925 | 0.288336272 |
| 646851 | NM_001013647FAM227A | family with sequence similarity 227 member A | 3.305558 | 3.365563 | 0.044677747 | 0.289577333 |
| 2644 | NM_005258 GCHFR | GTP cyclohydrolase I feedback regulator | 3.310001 | 2.600447 | 0.045830295 | 0.295051245 |
| 147138 | NM_152468 TMC8 | transmembrane channel like 8 | 3.310243 | 1.536401 | 0.047443076 | 0.298715722 |
| 100996738 | NR_145479 HERC2P11 | hect domain and RLD 2 pseudogene 11 | 3.314400 | 0.836902 | 0.04798532 | 0.298715722 |
| 4041 | NM_001291902LRP5 | LDL receptor related protein 5 | 3.315142 | 8.057205 | 0.043371468 | 0.284807883 |
| 677 | NM_001244698ZFP36L1 | ZFP36 ring finger protein like 1 | 3.315411 | 8.025786 | 0.043371759 | 0.284807883 |
| 339318 | NM_001029997ZNF181 | zinc finger protein 181 | 3.319911 | 4.463652 | 0.043450264 | 0.285232852 |
| 23092 | NM_001135608ARHGAP26 | Rho GTPase activating protein 26 | 3.321075 | 7.639388 | 0.043085392 | 0.283377213 |
| 10693 | NM_001193529CCT6B | chaperonin containing TCP1 subunit 6B | 3.321223 | 1.296684 | 0.046885327 | 0.298715722 |
| 100130933 | NM_001162997SMIM6 | small integral membrane protein 6 | 3.322694 | 2.572764 | 0.044372617 | 0.289176931 |
| 57491 | NM_001242412AHRR | aryl-hydrocarbon receptor repressor | 3.323456 | 4.048537 | 0.043360278 | 0.284807883 |
| 102724571 | NR_132738 LINC01759 | long intergenic non-protein coding RNA 1759 | 3.326076 | 0.419266 | 0.048693612 | 0.300777475 |
| 115399 | NM_198075 LRRC56 | leucine rich repeat containing 56 | 3.326807 | 0.989287 | 0.046680835 | 0.298715722 |
| 283537 | NM_001135919SLC46A3 | solute carrier family 46 member 3 | 3.330273 | 3.568483 | 0.043515347 | 0.285478893 |
| 284751 | NR_034124 LINC01270 | long intergenic non-protein coding RNA 1270 | 3.332481 | 2.943597 | 0.043537327 | 0.285532535 |
| 85442 | NM_001347864KNDC1 | kinase non-catalytic C-lobe domain containing 1 | 3.332875 | 2.826702 | 0.043988497 | 0.287307247 |
| 285464 | NM_175918 CRIPAK | cysteine rich PAK1 inhibitor | 3.333413 | 3.572230 | 0.042974686 | 0.283009035 |
| 6458 | NR_033412 SH3GL1P1 | SH3 domain containing GRB2 like 1, endophilin A2 pseudo | 3.333705 | 0.948086 | 0.04633913 | 0.29712489 |
| 256643 | NM_198279 CXorf23 | chromosome X open reading frame 23 | 3.336629 | 4.555257 | 0.042615875 | 0.281542438 |
| 23015 | NM_181077,NRGOLGA8A | golgin A8 family member A | 3.336703 | 6.960526 | 0.042337088 | 0.280291436 |
| 8673 | NM_003761 VAMP8 | vesicle associated membrane protein 8 | 3.340706 | 5.307334 | 0.042329585 | 0.280291436 |

| 3383 | NM_000201 | ICAM1 | intercellular adhesion molecule 1 | 3.341662 | 5.294667 | 0.043484995 | 0.285370282 |
| --- | --- | --- | --- | --- | --- | --- | --- |
| 56898 | NM_020139 | BDH2 | 3-hydroxybutyrate dehydrogenase 2 | 3.344720 | 3.936244 | 0.042559725 | 0.281351199 |
| 29116 | NM_013262 | MYLIP | myosin regulatory light chain interacting protein | 3.346578 | 3.751817 | 0.042481782 | 0.281015561 |
| 11001 | NM_001159629SLC27A2 | | solute carrier family 27 member 2 | 3.348233 | 6.393419 | 0.041844837 | 0.278583986 |
| 148418 | NM_001010971SAMD13 | | sterile alpha motif domain containing 13 | 3.348591 | 0.569190 | 0.047567285 | 0.298715722 |
| 100506965 | NR_146168 PWAR6 | | Prader Willi/Angelman region RNA 6 | 3.348653 | 2.752160 | 0.042753257 | 0.282146036 |
| 492311 | NM_001007189IGIP | | IgA inducing protein | 3.350558 | 2.384921 | 0.042945905 | 0.282909571 |
| 100996634 | NM_001277339LOC100996634 | | transmembrane protein FLJ37396 | 3.351995 | 1.918114 | 0.043602021 | 0.285685085 |
| 375190 | NM_001145710FAM228B | | family with sequence similarity 228 member B | 3.361087 | 1.732726 | 0.043255437 | 0.284405184 |
| 9028 | NM_001278720RHBDL1 | | rhomboid like 1 | 3.361751 | 1.147281 | 0.044827306 | 0.290275655 |
| 9976 | NM_005127 CLEC2B | | C-type lectin domain family 2 member B | 3.365493 | 1.491569 | 0.044618061 | 0.289577333 |
| 23530 | NM_001331026NNT | | nicotinamide nucleotide transhydrogenase | 3.371577 | 5.817255 | 0.040783347 | 0.273276167 |
| 284004 | NM_001330542HEXDC | | hexosaminidase D | 3.376382 | 4.230476 | 0.040807038 | 0.273346368 |
| 1519 | NM_001334 CTSO | | cathepsin O | 3.376561 | 2.153114 | 0.042334857 | 0.280291436 |
| 1775 | NM_001301680DNASE1L2 | | deoxyribonuclease 1 like 2 | 3.378078 | 0.260731 | 0.046043846 | 0.296162803 |
| 79628 | NM_024577 SH3TC2 | | SH3 domain and tetratricopeptide repeats 2 | 3.380605 | 1.162318 | 0.04459804 | 0.289577333 |
| 120071 | NM_001300721LARGE2 | | LARGE xylosyl- and glucuronyltransferase 2 | 3.380614 | 1.966939 | 0.042294173 | 0.280291436 |
| 399972 | NR_033839 GSEC | | G-quadruplex forming sequence containing lncRNA | 3.381143 | 2.133147 | 0.042299274 | 0.280291436 |
| 55080 | NM_001351355TAPBPL | | TAP binding protein like | 3.384169 | 3.005717 | 0.041366872 | 0.2763541 |
| 100287616 | NR_040066,NR LOXL1-AS1 | | LOXL1 antisense RNA 1 | 3.387630 | 0.850312 | 0.045639417 | 0.294096994 |
| 253039 | NR_024408 PSMD5-AS1 | | PSMD5 antisense RNA 1 (head to head) | 3.388201 | 4.058844 | 0.040235686 | 0.27074664 |
| 8437 | NM_001193520RASAL1 | | RAS protein activator like 1 | 3.391312 | 1.325062 | 0.043964515 | 0.28724131 |
| 1636 | NM_000789,N ACE | | angiotensin I converting enzyme | 3.397662 | 0.470043 | 0.044086297 | 0.287855133 |
| 349152 | NR_003561,NR DPY19L2P2 | | DPY19L2 pseudogene 2 | 3.400761 | 2.290548 | 0.041439752 | 0.276599312 |
| 140738 | NM_183240 TMEM37 | | transmembrane protein 37 | 3.403776 | 2.705372 | 0.04047961 | 0.272061197 |
| 4882 | NM_003995 NPR2 | | natriuretic peptide receptor 2 | 3.406440 | 3.364088 | 0.039642914 | 0.267541165 |
| 400619 | NR_033876 LINC00511 | | long intergenic non-protein coding RNA 511 | 3.408664 | 0.913966 | 0.043347538 | 0.284807883 |
| 140688 | NM_001256798NOL4L | | nucleolar protein 4 like | 3.411151 | 6.076707 | 0.03894543 | 0.263867087 |
| 9985 | NM_001048205REC8 | | REC8 meiotic recombination protein | 3.416247 | 1.884825 | 0.040718368 | 0.272929178 |
| 170425 | NR_038220 LINC00858 | | long intergenic non-protein coding RNA 858 | 3.419416 | 0.627483 | 0.043707125 | 0.286011354 |
| 57035 | NM_001321772RSRP1 | | arginine and serine rich protein 1 | 3.421870 | 5.576586 | 0.038506243 | 0.26226593 |
| 5732 | NM_000956 PTGER2 | | prostaglandin E receptor 2 | 3.423530 | 1.543442 | 0.040521452 | 0.272226776 |
| 54845 | NM_001034915ESRP1 | | epithelial splicing regulatory protein 1 | 3.423702 | 0.501967 | 0.042651415 | 0.281687266 |
| 2272 | NM_001166243FHIT | | fragile histidine triad | 3.425197 | 0.414813 | 0.043552143 | 0.285539166 |
| 81621 | NM_001319303KAZALD1 | | Kazal type serine peptidase inhibitor domain 1 | 3.425906 | 3.576365 | 0.03885866 | 0.263451779 |
| 80199 | NM_001171937FUZ | | fuzzy planar cell polarity protein | 3.429377 | 3.540819 | 0.038820386 | 0.263451341 |
| 489 | NM_005173,N ATP2A3 | | ATPase sarcoplasmic/endoplasmic reticulum Ca2+ transport | 3.430477 | 4.328694 | 0.03834972 | 0.261803284 |
| 374650 | NR_003246 GOLGA6L5P | | golgin A6 family-like 5, pseudogene | 3.431765 | 3.835313 | 0.038346097 | 0.261803284 |
| 3641 | NM_002195 INSL4 | | insulin like 4 | 3.432425 | 4.163660 | 0.038249244 | 0.261362961 |
| 54413 | NM_001166660NLGN3 | | neuroligin 3 | 3.441344 | 2.507196 | 0.038856005 | 0.263451779 |
| 643707 | NM_001267536GOLGA6L4 | | golgin A6 family-like 4 | 3.444748 | 2.245539 | 0.038577609 | 0.262492711 |
| 100652772 | NR_073113 NNT-AS1 | | NNT antisense RNA 1 | 3.446573 | 3.000670 | 0.038659852 | 0.262965816 |
| 9537 | NM_001258320TP53I11 | | tumor protein p53 inducible protein 11 | 3.448991 | 6.231206 | 0.037301718 | 0.25575771 |

| 100129550 | NR_024618 | LINC02035 | long intergenic non-protein coding RNA 2035 | 3.449580 | 3.709037 | 0.03766019 | 0.257861973 |
| --- | --- | --- | --- | --- | --- | --- | --- |
| 57507 | NM_020747 | ZNF608 | zinc finger protein 608 | 3.450448 | 2.128039 | 0.038980763 | 0.263949145 |
| 64115 | NM_022153 | VSIR | V-set immunoregulatory receptor | 3.455546 | 4.385348 | 0.037303409 | 0.25575771 |
| 79090 | NM_001270891TRAPPC6A | | trafficking protein particle complex 6A | 3.457176 | 4.228717 | 0.037210012 | 0.255387177 |
| 100132403 | NR_146178_chr FAM157B | | family with sequence similarity 157 member B (non-protein | 3.460131 | 1.824923 | 0.038725738 | 0.263240845 |
| 9644 | NM_014631 SH3PXD2A | | SH3 and PX domains 2A | 3.464478 | 5.341625 | 0.03671675 | 0.252930186 |
| 100616408 | NR_039969 MIR5047 | | microRNA 5047 | 3.465284 | 3.692192 | 0.037209957 | 0.255387177 |
| 93233 | NM_144577 CCDC114 | | coiled-coil domain containing 114 | 3.466539 | 0.588188 | 0.041796468 | 0.278351561 |
| 84992 | NM_001042616PIGY | | phosphatidylinositol glycan anchor biosynthesis class Y | 3.469088 | 0.474123 | 0.041453613 | 0.276602573 |
| 728262 | NR_146164 FAM157A | | family with sequence similarity 157 member A (non-protein | 3.471558 | 1.445847 | 0.039348467 | 0.266074872 |
| 3767 | NM_000525,N KCNJ11 | | potassium voltage-gated channel subfamily J member 11 | 3.471571 | 1.090517 | 0.039828639 | 0.268619301 |
| 9906 | NM_001199787SLC35E2 | | solute carrier family 35 member E2 | 3.473674 | 4.921960 | 0.03641181 | 0.252067703 |
| 2165 | NM_001994 F13B | | coagulation factor XIII B chain | 3.474633 | -0.347084 | 0.04216587 | 0.280000329 |
| 3306 | NM_021979 HSPA2 | | heat shock protein family A (Hsp70) member 2 | 3.475743 | 6.841193 | 0.036174244 | 0.250758795 |
| 9866 | NM_014818 TRIM66 | | tripartite motif containing 66 | 3.476070 | 4.829151 | 0.036309703 | 0.251529207 |
| 10780 | NM_001144824ZNF234 | | zinc finger protein 234 | 3.476346 | 3.541489 | 0.036719814 | 0.252930186 |
| 9476 | NM_004851 NAPSA | | napsin A aspartic peptidase | 3.480666 | 0.892363 | 0.039975865 | 0.269436548 |
| 1953 | NM_001409 MEGF6 | | multiple EGF like domains 6 | 3.482589 | 5.197151 | 0.036030045 | 0.249842935 |
| 10752 | NM_001253387CHL1 | | cell adhesion molecule L1 like | 3.483258 | 3.432497 | 0.036429523 | 0.252105946 |
| 90843 | NM_001006684TCEAL8 | | transcription elongation factor A like 8 | 3.483262 | 5.345956 | 0.036007872 | 0.249772915 |
| 376497 | NM_198580 SLC27A1 | | solute carrier family 27 member 1 | 3.484034 | 3.900452 | 0.036338465 | 0.251644173 |
| 4192 | NM_001012333MDK | | midkine (neurite growth-promoting factor 2) | 3.484825 | 3.312856 | 0.036630221 | 0.252856432 |
| 2217 | NM_001136019FCGRT | | Fc fragment of IgG receptor and transporter | 3.485078 | 5.182272 | 0.035905901 | 0.249232737 |
| 84448 | NM_001130083ABLIM2 | | actin binding LIM protein family member 2 | 3.487853 | 0.068985 | 0.045011161 | 0.290954955 |
| 29800 | NM_001323627ZDHHC1 | | zinc finger DHHC-type containing 1 | 3.496427 | 3.635418 | 0.035893028 | 0.249227017 |
| 100506548 | NR_037665 LOC100506548 | | uncharacterized LOC100506548 | 3.497274 | 5.310542 | 0.035399875 | 0.246963379 |
| 100132832 | NR_028058 PMS2P9 | | PMS1 homolog 2, mismatch repair system component pseu | 3.500044 | 1.748067 | 0.037007049 | 0.254400402 |
| 154141 | NM_001080480MBOAT1 | | membrane bound O-acyltransferase domain containing 1 | 3.502233 | 3.489092 | 0.035846198 | 0.248985428 |
| 83699 | NM_031469 SH3BGRL2 | | SH3 domain binding glutamate rich protein like 2 | 3.503212 | 3.865350 | 0.03544314 | 0.247181843 |
| 283232 | NM_001042463TMEM80 | | transmembrane protein 80 | 3.504242 | 3.251232 | 0.035646889 | 0.248100908 |
| 717 | NM_000063,N C2 | | complement C2 | 3.509251 | 0.351750 | 0.039685012 | 0.267737922 |
| 445 | NM_000050,N ASS1 | | argininosuccinate synthase 1 | 3.513778 | 6.182007 | 0.034695261 | 0.243113646 |
| 55056 | NR_026891 GABPB1-IT1 | | GABPB1 intronic transcript | 3.514176 | 3.149570 | 0.03550694 | 0.247459924 |
| 1773 | NM_001351825DNASE1 | | deoxyribonuclease 1 | 3.518256 | 2.391543 | 0.035637829 | 0.248100908 |
| 116442 | NM_171998 RAB39B | | RAB39B, member RAS oncogene family | 3.521593 | 2.304065 | 0.035497269 | 0.247459924 |
| 6490 | NM_001200053PMEL | | premelanosome protein | 3.522211 | 1.020634 | 0.038209795 | 0.261192862 |
| 93166 | NM_001136239PRDM6 | | PR/SET domain 6 | 3.522961 | 0.220773 | 0.039467241 | 0.266742222 |
| 4602 | NM_001130172MYB | | MYB proto-oncogene, transcription factor | 3.527796 | 0.756266 | 0.038153897 | 0.260896978 |
| 55815 | NM_001288990TSNAXIP1 | | translin associated factor X interacting protein 1 | 3.529345 | 0.208083 | 0.043075918 | 0.283377213 |
| 147948 | NM_001320371ZNF582 | | zinc finger protein 582 | 3.540249 | 1.211586 | 0.036982735 | 0.254317779 |
| 347051 | NM_001010893SLC10A5 | | solute carrier family 10 member 5 | 3.540279 | -0.710938 | 0.047910007 | 0.298715722 |
| 100631383 | NM_001242939FAM47E-STBD1 | | FAM47E-STBD1 readthrough | 3.541896 | -0.352828 | 0.047102154 | 0.298715722 |
| 9414 | NM_001170414TJP2 | | tight junction protein 2 | 3.542420 | 4.777237 | 0.033771634 | 0.238337168 |

| 100294145 | NR_037177,NR LOC100294145 | uncharacterized LOC100294145 | 3.543934 | 4.982078 | 0.033631267 | 0.237988891 |
| --- | --- | --- | --- | --- | --- | --- |
| 90333 | NM_001008801ZNF468 | zinc finger protein 468 | 3.548737 | 1.859944 | 0.03709175 | 0.254897956 |
| 2886 | NM_001030002GRB7 | growth factor receptor bound protein 7 | 3.549204 | 4.257423 | 0.03357886 | 0.237869427 |
| 11094 | NM_001135775CACFD1 | calcium channel flower domain containing 1 | 3.551505 | 3.643733 | 0.033822944 | 0.238496884 |
| 1375 | NM_001145134CPT1B | carnitine palmitoyltransferase 1B | 3.553230 | 5.362369 | 0.033248219 | 0.235931188 |
| 497258 | NR_002832,NR BDNF-AS | BDNF antisense RNA | 3.555540 | -0.849275 | 0.048439637 | 0.299633338 |
| 100129196 | NR_034182 MATN1-AS1 | MATN1 antisense RNA 1 | 3.557738 | 1.389785 | 0.035817012 | 0.248866272 |
| 1513 | NM_000396 CTSK | cathepsin K | 3.558539 | 2.657066 | 0.034108108 | 0.239974906 |
| 93663 | NM_033515 ARHGAP18 | Rho GTPase activating protein 18 | 3.559799 | 5.674796 | 0.032994713 | 0.234857414 |
| 80221 | NM_001288968ACSF2 | acyl-CoA synthetase family member 2 | 3.562784 | 4.668609 | 0.033040742 | 0.235104142 |
| 22807 | NM_001079526IKZF2 | IKAROS family zinc finger 2 | 3.563381 | 0.741578 | 0.036687027 | 0.25287276 |
| 84952 | NM_001252335CGNL1 | cingulin like 1 | 3.564378 | 2.604968 | 0.033902423 | 0.238852733 |
| 57658 | NM_001143682CALCOCO1 | calcium binding and coiled-coil domain 1 | 3.569321 | 5.591458 | 0.03264921 | 0.233618934 |
| 221981 | NM_015204 THSD7A | thrombospondin type 1 domain containing 7A | 3.570541 | 5.910146 | 0.032566427 | 0.233618934 |
| 55083 | NM_018012 KIF26B | kinesin family member 26B | 3.571171 | -0.491641 | 0.042778838 | 0.282168453 |
| 100532732 | NR_037846 MSH5-SAPCD1 | MSH5-SAPCD1 readthrough (NMD candidate) | 3.577504 | 3.468813 | 0.0327766 | 0.233626455 |
| 56911 | NM_001286617MAP3K7CL | MAP3K7 C-terminal like | 3.579070 | 2.097523 | 0.034001925 | 0.239390677 |
| 4151 | NM_005368,N MB | myoglobin | 3.581095 | 1.236228 | 0.035295386 | 0.246317498 |
| 25992 | NM_001080437SNED1 | sushi, nidogen and EGF like domains 1 | 3.583042 | 1.433533 | 0.034862003 | 0.243637802 |
| 100271927 | NM_001277335RASA4B | RAS p21 protein activator 4B | 3.587219 | 1.835211 | 0.033706426 | 0.238099055 |
| 10811 | NM_001256067NOXA1 | NADPH oxidase activator 1 | 3.587546 | 2.922890 | 0.032666271 | 0.233618934 |
| 29964 | NM_013397 PRICKLE4 | prickle planar cell polarity protein 4 | 3.588483 | 3.242413 | 0.032500883 | 0.233618934 |
| 388165 | NR_003661 UBE2Q2P1 | ubiquitin conjugating enzyme E2 Q2 pseudogene 1 | 3.590311 | 2.146239 | 0.033384955 | 0.236576841 |
| 100130894 | NR_034083 VAC14-AS1 | VAC14 antisense RNA 1 | 3.593668 | -1.154106 | 0.047581058 | 0.298715722 |
| 644943 | NM_001080521RASSF10 | Ras association domain family member 10 | 3.593872 | 4.566675 | 0.031950334 | 0.233618934 |
| 157638 | NM_174911 FAM84B | family with sequence similarity 84 member B | 3.594843 | 4.670397 | 0.031854659 | 0.233618934 |
| 652 | NM_001202,N BMP4 | bone morphogenetic protein 4 | 3.597534 | 4.708236 | 0.031772842 | 0.233618934 |
| 642799 | NM_001277324NPIPA2 | nuclear pore complex interacting protein family member A2 | 3.597693 | 1.415640 | 0.034316326 | 0.24111182 |
| 8705 | NM_003782 B3GALT4 | beta-1,3-galactosyltransferase 4 | 3.601206 | 0.368873 | 0.036298895 | 0.251529207 |
| 100131827 | NM_001128223ZNF717 | zinc finger protein 717 | 3.602390 | 2.663723 | 0.032516803 | 0.233618934 |
| 6820 | NM_004605,N SULT2B1 | sulfotransferase family 2B member 1 | 3.606004 | 3.761585 | 0.031840157 | 0.233618934 |
| 145482 | NM_001146154PTGR2 | prostaglandin reductase 2 | 3.608744 | 5.046990 | 0.031335139 | 0.232651598 |
| 5457 | NM_006237 POU4F1 | POU class 4 homeobox 1 | 3.608975 | 2.871903 | 0.032297987 | 0.233618934 |
| 9962 | NM_005116,N SLC23A2 | solute carrier family 23 member 2 | 3.611298 | 8.042497 | 0.03107453 | 0.23121462 |
| 79935 | NM_024877 CNTD2 | cyclin N-terminal domain containing 2 | 3.621134 | -0.559118 | 0.041351253 | 0.2763541 |
| 285489 | NM_001164673DOK7 | docking protein 7 | 3.625872 | 0.758025 | 0.033732353 | 0.238141201 |
| 613038 | NR_002557,NR LOC613038 | SAGA complex associated factor 29 pseudogene | 3.626146 | 1.447328 | 0.033279518 | 0.235991372 |
| 57333 | NM_020650 RCN3 | reticulocalbin 3 | 3.627959 | 1.928668 | 0.032258779 | 0.233618934 |
| 8353 | NM_003532 HIST1H3E | histone cluster 1 H3 family member e | 3.631432 | 0.460276 | 0.034361509 | 0.241347307 |
| 11201 | NM_001351610POLI | DNA polymerase iota | 3.633332 | 4.815010 | 0.030509266 | 0.227992837 |
| 100505696 | NR_046084 SH3BP5-AS1 | SH3BP5 antisense RNA 1 | 3.633944 | 3.076640 | 0.030890967 | 0.230550528 |
| 6094 | NM_000327 ROM1 | retinal outer segment membrane protein 1 | 3.637102 | 0.299738 | 0.034839611 | 0.243637802 |
| 57088 | NM_001128304PLSCR4 | phospholipid scramblase 4 | 3.642355 | 4.267925 | 0.030300011 | 0.226920976 |

| 378938 | NR_002819,NR MALAT1 | metastasis associated lung adenocarcinoma transcript 1 (no | 3.642744 | 9.248461 | 0.030007788 | 0.224976837 |
| --- | --- | --- | --- | --- | --- | --- |
| 100131755 | NM_001256155ARMCX4 | armadillo repeat containing, X-linked 4 | 3.643460 | 1.525398 | 0.034573526 | 0.242365086 |
| 221442 | NR_026938 ADCY10P1 | adenylate cyclase 10, soluble pseudogene 1 | 3.655569 | -0.693149 | 0.045232199 | 0.292018739 |
| 2946 | NM_000848,N GSTM2 | glutathione S-transferase mu 2 | 3.657963 | 0.891283 | 0.033194692 | 0.235855103 |
| 100507547 | NR_037169,NR LOC100507547 | uncharacterized LOC100507547 | 3.660271 | 0.569908 | 0.034210573 | 0.240532191 |
| 286527 | NM_001350211TMSB15B | thymosin beta 15B | 3.665017 | 1.319779 | 0.032963413 | 0.234715386 |
| 26872 | NM_012449 STEAP1 | STEAP family member 1 | 3.666831 | 5.836038 | 0.029343139 | 0.221386859 |
| 100130557 | NR_024567 NFYC-AS1 | NFYC antisense RNA 1 | 3.667974 | 1.428678 | 0.031845915 | 0.233618934 |
| 100507424 | NR_038933 LOC100507424 | uncharacterized LOC100507424 | 3.670045 | 0.323274 | 0.033714872 | 0.238099055 |
| 53373 | NM_001143819TPCN1 | two pore segment channel 1 | 3.673555 | 7.319440 | 0.029046749 | 0.219753043 |
| 338376 | NM_176891 IFNE | interferon epsilon | 3.675776 | 2.144362 | 0.030386927 | 0.227242802 |
| 55647 | NM_017817 RAB20 | RAB20, member RAS oncogene family | 3.676059 | 2.434835 | 0.030482097 | 0.227872127 |
| 8839 | NM_001323369WISP2 | WNT1 inducible signaling pathway protein 2 | 3.676289 | 3.994621 | 0.029370139 | 0.221480617 |
| 80350 | NR_028092,NR LPAL2 | lipoprotein(a) like 2, pseudogene | 3.678154 | -0.012032 | 0.03403126 | 0.239515689 |
| 4051 | NM_000896,N CYP4F3 | cytochrome P450 family 4 subfamily F member 3 | 3.678372 | 4.517962 | 0.029066682 | 0.219753043 |
| 56128 | NM_019120 PCDHB8 | protocadherin beta 8 | 3.678405 | -0.339540 | 0.042345213 | 0.280291436 |
| 285908 | NR_026873 LINC00174 | long intergenic non-protein coding RNA 174 | 3.679077 | 2.775501 | 0.029541227 | 0.222284568 |
| 9001 | NM_001079870HAP1 | huntingtin associated protein 1 | 3.679587 | 2.470049 | 0.03035837 | 0.227111353 |
| 171546 | NM_138288 SPTSSA | serine palmitoyltransferase small subunit A | 3.680606 | 7.097369 | 0.02883624 | 0.218489975 |
| 401247 | NR_130726 LINC00243 | long intergenic non-protein coding RNA 243 | 3.683336 | -0.971216 | 0.048308159 | 0.298998884 |
| 3993 | NM_001015002LLGL2 | LLGL2, scribble cell polarity complex component | 3.686824 | 6.530427 | 0.028647489 | 0.217775582 |
| 5446 | NM_000940 PON3 | paraoxonase 3 | 3.689570 | 4.971246 | 0.028687376 | 0.217840814 |
| 8741 | NM_001198622TNFSF13 | TNF superfamily member 13 | 3.693445 | 2.363852 | 0.029980311 | 0.224852335 |
| 644794 | NR_136637 LOC644794 | uncharacterized LOC644794 | 3.696809 | 2.596367 | 0.029295794 | 0.221242604 |
| 5308 | NM_000325,N PITX2 | paired like homeodomain 2 | 3.697861 | 1.290370 | 0.031810016 | 0.233618934 |
| 90668 | NM_138360 CARMIL3 | capping protein regulator and myosin 1 linker 3 | 3.698862 | 1.354655 | 0.031109096 | 0.231388584 |
| 493913 | NR_103711 PAPPA-AS1 | PAPPA antisense RNA 1 | 3.702878 | -1.183442 | 0.046505816 | 0.29800892 |
| 100130950 | NR_034082 LOC100130950 | uncharacterized LOC100130950 | 3.704526 | -0.053322 | 0.037757786 | 0.258444675 |
| 91074 | NM_052997 ANKRD30A | ankyrin repeat domain 30A | 3.706593 | 4.466041 | 0.028214117 | 0.215310308 |
| 56413 | NM_001164692LTB4R2 | leukotriene B4 receptor 2 | 3.708663 | 2.997800 | 0.028729825 | 0.21800309 |
| 50832 | NM_016944 TAS2R4 | taste 2 receptor member 4 | 3.710695 | -1.071687 | 0.042302593 | 0.280291436 |
| 101060264 | NR_126415,NR FOXP4-AS1 | FOXP4 antisense RNA 1 | 3.713396 | -0.649153 | 0.04088034 | 0.273748734 |
| 6911 | NM_004608 TBX6 | T-box 6 | 3.717178 | 0.498910 | 0.031992839 | 0.233618934 |
| 55504 | NM_001204458TNFRSF19 | TNF receptor superfamily member 19 | 3.717375 | 2.440457 | 0.029278107 | 0.221189792 |
| 102724094 | NR_120519,NR LOC102724094 | uncharacterized LOC102724094 | 3.717414 | -0.276409 | 0.038794572 | 0.263451341 |
| 3728 | NM_002230,N JUP | junction plakoglobin | 3.720424 | 8.445300 | 0.027584015 | 0.211791744 |
| 4854 | NM_000435 NOTCH3 | notch 3 | 3.721478 | 6.414657 | 0.027593848 | 0.211791744 |
| 30819 | NM_014591,N KCNIP2 | potassium voltage-gated channel interacting protein 2 | 3.725288 | 0.813847 | 0.030891579 | 0.230550528 |
| 1052 | NM_005195 CEBPD | CCAAT/enhancer binding protein delta | 3.731661 | 4.692152 | 0.02749815 | 0.211370953 |
| 102723354 | NR_110546 LINC02298 | long intergenic non-protein coding RNA 2298 | 3.734242 | 1.425420 | 0.029948522 | 0.224695385 |
| 342184 | NM_001103184FMN1 | formin 1 | 3.740875 | 4.794640 | 0.02719043 | 0.209989979 |
| 10156 | NM_001079877RASA4 | RAS p21 protein activator 4 | 3.746218 | 2.661581 | 0.027839096 | 0.213236956 |
| 128497 | NM_080608 SPATA25 | spermatogenesis associated 25 | 3.748387 | -0.758263 | 0.039472949 | 0.266742222 |

| 157506 | NM_172037 | RDH10 | retinol dehydrogenase 10 | 3.748690 | 5.850330 | 0.026825986 | 0.207981827 |
| --- | --- | --- | --- | --- | --- | --- | --- |
| 105379194 | NR_134244,NR LOC105379194 | | uncharacterized LOC105379194 | 3.749704 | -0.946733 | 0.04069383 | 0.272853118 |
| 6297 | NM_001291446SALL2 | | spalt like transcription factor 2 | 3.750064 | 2.307283 | 0.028152365 | 0.214961857 |
| 22973 | NR_004405 LAMB2P1 | | laminin subunit beta 2 pseudogene 1 | 3.751363 | -1.321710 | 0.042242227 | 0.280291436 |
| 222962 | NM_001040661SLC29A4 | | solute carrier family 29 member 4 | 3.752683 | 4.964950 | 0.026821301 | 0.207981827 |
| 8864 | NM_022817 PER2 | | period circadian clock 2 | 3.753366 | 4.978987 | 0.026751779 | 0.207717927 |
| 102465463 | NR_106830 MIR6772 | | microRNA 6772 | 3.753516 | -1.344076 | 0.044268966 | 0.28868718 |
| 5787 | NM_001109754PTPRB | | protein tyrosine phosphatase, receptor type B | 3.755096 | 1.004229 | 0.029875405 | 0.224295781 |
| 8557 | NM_003673 TCAP | | titin-cap | 3.755302 | 0.010894 | 0.031264707 | 0.232295423 |
| 11000 | NM_001317929SLC27A3 | | solute carrier family 27 member 3 | 3.759702 | 2.347917 | 0.027428817 | 0.210999154 |
| 9518 | NM_004864 GDF15 | | growth differentiation factor 15 | 3.762348 | 6.978698 | 0.026388376 | 0.205978701 |
| 440918 | NR_147695 FLJ46875 | | uncharacterized LOC440918 | 3.763791 | 1.665946 | 0.027843994 | 0.213236956 |
| 100302692 | NR_028379 FTX | | FTX transcript, XIST regulator (non-protein coding) | 3.766040 | 0.874294 | 0.029535763 | 0.222284568 |
| 100996301 | NR_121634,NR FOXD3-AS1 | | FOXD3 antisense RNA 1 (head to head) | 3.768202 | 1.563019 | 0.02865773 | 0.217775582 |
| 472 | NM_000051,N ATM | | ATM serine/threonine kinase | 3.771716 | 6.840861 | 0.02612934 | 0.204962231 |
| 401265 | NM_001003760KLHL31 | | kelch like family member 31 | 3.772411 | -0.277499 | 0.036000762 | 0.249772915 |
| 400931 | NR_027033,NR MIRLET7BHG | | MIRLET7B host gene | 3.774970 | 0.627620 | 0.029314488 | 0.221302987 |
| 26018 | NM_015541 LRIG1 | | leucine rich repeats and immunoglobulin like domains 1 | 3.780949 | 4.270545 | 0.026224848 | 0.205382014 |
| 10307 | NM_006051,N APBB3 | | amyloid beta precursor protein binding family B member 3 | 3.781477 | 3.915333 | 0.026244908 | 0.205382014 |
| 100506084 | NM_001039083ARL17B | | ADP ribosylation factor like GTPase 17B | 3.786223 | 0.876634 | 0.028464058 | 0.216622165 |
| 7294 | NM_003328 TXK | | TXK tyrosine kinase | 3.788023 | -0.542428 | 0.036656632 | 0.252856432 |
| 8928 | NM_003923 FOXH1 | | forkhead box H1 | 3.788023 | -0.650912 | 0.036656632 | 0.252856432 |
| 79895 | NM_024837,NRATP8B4 | | ATPase phospholipid transporting 8B4 (putative) | 3.788298 | 1.574076 | 0.028056173 | 0.214544152 |
| 101929634 | NR_135293 LINC02280 | | long intergenic non-protein coding RNA 2280 | 3.788713 | -1.071844 | 0.039154437 | 0.265022751 |
| 440934 | NR_136638,NR LOC440934 | | uncharacterized LOC440934 | 3.789908 | -1.319520 | 0.04357416 | 0.285592997 |
| 100532742 | NR_037859 PIR-FIGF | | PIR-FIGF readthrough | 3.792588 | 0.685340 | 0.029046101 | 0.219753043 |
| 5742 | NM_000962,N PTGS1 | | prostaglandin-endoperoxide synthase 1 | 3.794346 | 1.287410 | 0.028143577 | 0.214961857 |
| 284185 | NR_038080 LINC00482 | | long intergenic non-protein coding RNA 482 | 3.795480 | 1.299174 | 0.027827817 | 0.213236956 |
| 59285 | NM_031897,N CACNG6 | | calcium voltage-gated channel auxiliary subunit gamma 6 | 3.797161 | 4.146876 | 0.025723663 | 0.20293439 |
| 6623 | NM_001330120SNCG | | synuclein gamma | 3.798591 | 3.902088 | 0.025646244 | 0.202632756 |
| 57165 | NM_020435 GJC2 | | gap junction protein gamma 2 | 3.799766 | -1.760208 | 0.046308998 | 0.29702376 |
| 246329 | NM_001286256STAC3 | | SH3 and cysteine rich domain 3 | 3.801247 | 0.641833 | 0.028676675 | 0.217839527 |
| 100130827 | NM_001199824SBK3 | | SH3 domain binding kinase family member 3 | 3.802780 | 0.700327 | 0.028761955 | 0.218115693 |
| 55567 | NM_001347886DNAH3 | | dynein axonemal heavy chain 3 | 3.805854 | 0.189603 | 0.02972362 | 0.223494393 |
| 3777 | NM_002246 KCNK3 | | potassium two pore domain channel subfamily K member 3 | 3.806246 | 2.944548 | 0.026059813 | 0.204572005 |
| 92960 | NM_001270539PEX11G | | peroxisomal biogenesis factor 11 gamma | 3.811306 | 0.934871 | 0.028330767 | 0.21584601 |
| 79924 | NM_001253845ADM2 | | adrenomedullin 2 | 3.812361 | 2.402675 | 0.025890577 | 0.203475146 |
| 100507331 | NR_038357 ZSWIM8-AS1 | | ZSWIM8 antisense RNA 1 | 3.814984 | 1.325858 | 0.027534916 | 0.211574941 |
| 283316 | NM_001297650CD163L1 | | CD163 molecule like 1 | 3.815195 | 1.634152 | 0.02728648 | 0.210330017 |
| 107985544 | NR_146603 LOC107985544 | | uncharacterized LOC107985544 | 3.815580 | 1.469877 | 0.027253446 | 0.210271362 |
| 150135 | NR_027272 LINC00479 | | long intergenic non-protein coding RNA 479 | 3.818369 | -0.758397 | 0.036820723 | 0.25345646 |
| 4329 | NM_001278593ALDH6A1 | | aldehyde dehydrogenase 6 family member A1 | 3.822739 | 4.462399 | 0.024943868 | 0.197763094 |
| 4316 | NM_002423 MMP7 | | matrix metallopeptidase 7 | 3.827951 | 5.708275 | 0.024714804 | 0.196398663 |

| 92285 | NM_152279 | ZNF585B | zinc finger protein 585B | 3.830535 | 4.299839 | 0.024912263 | 0.197588253 |
| --- | --- | --- | --- | --- | --- | --- | --- |
| 728 | NM_001736 | C5AR1 | complement C5a receptor 1 | 3.833519 | 1.467410 | 0.026734972 | 0.207665372 |
| 284233 | NR_026756 | CYP4F35P | cytochrome P450 family 4 subfamily F member 35, pseudo | 3.835462 | 2.078724 | 0.02569642 | 0.202882294 |
| 9220 | NM_004740 | TIAF1 | TGFB1-induced anti-apoptotic factor 1 | 3.836333 | 1.012406 | 0.026984102 | 0.209050991 |
| 1286 | NM_000092 | COL4A4 | collagen type IV alpha 4 chain | 3.838770 | 4.694848 | 0.024463953 | 0.19477179 |
| 81669 | NM_001039577CCNL2 | | cyclin L2 | 3.839299 | 7.588257 | 0.02429384 | 0.193723239 |
| 285313 | NM_001178145IGSF10 | | immunoglobulin superfamily member 10 | 3.847600 | 2.617172 | 0.024962161 | 0.197832295 |
| 101929511 | NR_135639 LINC01970 | | long intergenic non-protein coding RNA 1970 | 3.852604 | 0.098359 | 0.03321307 | 0.235855103 |
| 10673 | NM_001145645TNFSF13B | | TNF superfamily member 13b | 3.854793 | -0.981352 | 0.04015312 | 0.270454992 |
| 51171 | NM_016246 HSD17B14 | | hydroxysteroid 17-beta dehydrogenase 14 | 3.857924 | 3.793882 | 0.024266274 | 0.193578048 |
| 2710 | NM_000167,N GK | | glycerol kinase | 3.861211 | 3.741743 | 0.024229368 | 0.193358211 |
| 399687 | NM_001346765MYO18A | | myosin XVIIIA | 3.861469 | 7.709108 | 0.023729247 | 0.190321627 |
| 100128893 | NR_102763 GATA6-AS1 | | GATA6 antisense RNA 1 (head to head) | 3.862965 | -1.578265 | 0.043639988 | 0.285802312 |
| 55251 | NM_001104925PCMTD2 | | protein-L-isoaspartate (D-aspartate) O-methyltransferase do | 3.873796 | 5.675399 | 0.023494002 | 0.188727542 |
| 254100 | NR_038923 SSSCA1-AS1 | | SSSCA1 antisense RNA 1 (head to head) | 3.874470 | -0.633698 | 0.035582706 | 0.247820971 |
| 84913 | NM_032827 ATOH8 | | atonal bHLH transcription factor 8 | 3.876793 | 3.059024 | 0.023966029 | 0.191823324 |
| 54494 | NM_001271562C11orf71 | | chromosome 11 open reading frame 71 | 3.880605 | 2.743913 | 0.024063288 | 0.192403983 |
| 10317 | NM_001278650B3GALT5 | | beta-1,3-galactosyltransferase 5 | 3.882023 | 2.148263 | 0.024449685 | 0.194740749 |
| 644890 | NM_001080836MEIG1 | | meiosis/spermiogenesis associated 1 | 3.886541 | -0.713932 | 0.040604915 | 0.272698917 |
| 3425 | NM_000203,NRIDUA | | iduronidase, alpha-L- | 3.890009 | 4.215264 | 0.023243022 | 0.187001913 |
| 9315 | NM_001142474NREP | | neuronal regeneration related protein | 3.890372 | 3.883919 | 0.023529028 | 0.188862218 |
| 100287569 | NR_027345,NR LINC00173 | | long intergenic non-protein coding RNA 173 | 3.896306 | 2.789928 | 0.02402558 | 0.19217677 |
| 2661 | NM_001288824GDF9 | | growth differentiation factor 9 | 3.901472 | -0.133922 | 0.031833672 | 0.233618934 |
| 163175 | NM_139284 LGI4 | | leucine rich repeat LGI family member 4 | 3.905432 | -1.239102 | 0.038259949 | 0.261362961 |
| 79853 | NM_024795 TM4SF20 | | transmembrane 4 L six family member 20 | 3.912811 | 5.577693 | 0.022535189 | 0.184819135 |
| 646643 | NM_001101401SBK2 | | SH3 domain binding kinase family member 2 | 3.913561 | 0.313819 | 0.025697443 | 0.202882294 |
| 56256 | NM_019605 SERTAD4 | | SERTA domain containing 4 | 3.921889 | 1.023632 | 0.025209483 | 0.199361347 |
| 3428 | NM_001206567IFI16 | | interferon gamma inducible protein 16 | 3.921908 | 3.613708 | 0.022872414 | 0.18519359 |
| 100130311 | NM_001145536C17orf107 | | chromosome 17 open reading frame 107 | 3.922243 | 0.455857 | 0.025397355 | 0.200742946 |
| 645158 | NR_033754 CBX3P2 | | chromobox 3 pseudogene 2 | 3.923343 | -0.672223 | 0.033214692 | 0.235855103 |
| 177 | NM_001136,N AGER | | advanced glycosylation end-product specific receptor | 3.924104 | 2.258440 | 0.02334837 | 0.187776451 |
| 387644 | NR_026795 FAM238C | | family with sequence similarity 238 member C (non-protein | 3.926646 | 0.752847 | 0.025141768 | 0.19895083 |
| 114823 | NM_052925 LENG8 | | leukocyte receptor cluster member 8 | 3.929987 | 8.171712 | 0.022071687 | 0.184819135 |
| 25861 | NM_001083885WHRN | | whirlin | 3.938462 | 3.654128 | 0.02240007 | 0.184819135 |
| 80164 | NM_001190467PRR36 | | proline rich 36 | 3.939193 | 1.636323 | 0.02379583 | 0.190707769 |
| 100873954 | NR_046757 SNRK-AS1 | | SNRK antisense RNA 1 | 3.939967 | -0.393225 | 0.030097892 | 0.225570614 |
| 54806 | NM_001134830AHI1 | | Abelson helper integration site 1 | 3.940950 | 5.889458 | 0.021882439 | 0.18384982 |
| 1241 | NM_001143919LTB4R | | leukotriene B4 receptor | 3.948812 | 5.701881 | 0.021710589 | 0.182777177 |
| 374819 | NM_001303255LRRC37A3 | | leucine rich repeat containing 37 member A3 | 3.954426 | 3.065830 | 0.022070252 | 0.184819135 |
| 7031 | NM_003225 TFF1 | | trefoil factor 1 | 3.957758 | 3.622804 | 0.021769842 | 0.183085373 |
| 4671 | NM_001346870NAIP | | NLR family apoptosis inhibitory protein | 3.962329 | 2.240511 | 0.022498995 | 0.184819135 |
| 283417 | NM_173812 DPY19L2 | | dpy-19 like 2 | 3.964201 | 2.668022 | 0.022444479 | 0.184819135 |
| 393 | NM_001164741ARHGAP4 | | Rho GTPase activating protein 4 | 3.970528 | 1.376566 | 0.023237748 | 0.187001913 |

| 28986 | NM_014061 | MAGEH1 | MAGE family member H1 | 3.973278 | 2.763281 | 0.022192439 | 0.184819135 |
| --- | --- | --- | --- | --- | --- | --- | --- |
| 10023 | NM_005479 | FRAT1 | FRAT1, WNT signaling pathway regulator | 3.974918 | 1.901874 | 0.02237105 | 0.184819135 |
| 837 | NM_001225,N | CASP4 | caspase 4 | 3.976960 | 3.741813 | 0.021476783 | 0.18102985 |
| 388780 | NM_001287682LOC388780 | | uncharacterized LOC388780 | 3.989363 | -1.192608 | 0.03881103 | 0.263451341 |
| 102724450 | NR_125997 LOC102724450 | | uncharacterized LOC102724450 | 3.989363 | -1.391422 | 0.03881103 | 0.263451341 |
| 7786 | NM_001193511MAP3K12 | | mitogen-activated protein kinase kinase kinase 12 | 3.993482 | 5.655613 | 0.020732202 | 0.175783302 |
| 146547 | NM_001258290PRSS36 | | protease, serine 36 | 3.998993 | -0.925018 | 0.036545084 | 0.252736566 |
| 84163 | NM_001281447GTF2IRD2 | | GTF2I repeat domain containing 2 | 4.001051 | 3.049592 | 0.021059926 | 0.178023933 |
| 389791 | NR_024425 PTGES2-AS1 | | PTGES2 antisense RNA 1 (head to head) | 4.022768 | -0.540105 | 0.031200715 | 0.231973514 |
| 222950 | NM_173564 NYAP1 | | neuronal tyrosine phosphorylated phosphoinositide-3-kinas | 4.022925 | 1.119947 | 0.022825508 | 0.185082572 |
| 3547 | NM_001170961IGSF1 | | immunoglobulin superfamily member 1 | 4.026530 | 1.744631 | 0.021777565 | 0.183085373 |
| 1950 | NM_001178130EGF | | epidermal growth factor | 4.030085 | 0.425495 | 0.023786482 | 0.190706737 |
| 100272228 | NR_027456 LINC00894 | | long intergenic non-protein coding RNA 894 | 4.034062 | 2.843922 | 0.020335962 | 0.173178001 |
| 100113393 | NR_003695 SNORD12B | | small nucleolar RNA, C/D box 12B | 4.034151 | -0.481497 | 0.028772771 | 0.218115693 |
| 8816 | NM_001284206DCAF5 | | DDB1 and CUL4 associated factor 5 | 4.042834 | 5.920571 | 0.019712257 | 0.169190056 |
| 534 | NM_001204078ATP6V1G2 | | ATPase H+ transporting V1 subunit G2 | 4.043228 | -0.262081 | 0.026272217 | 0.205382014 |
| 6296 | NM_005622,N ACSM3 | | acyl-CoA synthetase medium-chain family member 3 | 4.051015 | 3.952880 | 0.019758655 | 0.169517954 |
| 131870 | NM_001171905NUDT16 | | nudix hydrolase 16 | 4.051866 | 4.350544 | 0.019636854 | 0.168682863 |
| 8997 | NM_001024660KALRN | | kalirin RhoGEF kinase | 4.054764 | 2.143557 | 0.020489009 | 0.173979944 |
| 55867 | NM_001307985SLC22A11 | | solute carrier family 22 member 11 | 4.056339 | -0.889683 | 0.033884188 | 0.238805596 |
| 4303 | NM_001170931FOXO4 | | forkhead box O4 | 4.062332 | 2.865550 | 0.019960398 | 0.170965144 |
| 5893 | NM_001297419RAD52 | | RAD52 homolog, DNA repair protein | 4.063339 | 4.813558 | 0.019358073 | 0.166564782 |
| 7098 | NM_003265 TLR3 | | toll like receptor 3 | 4.065737 | 1.911208 | 0.021132683 | 0.178565989 |
| 7227 | NM_001282902TRPS1 | | transcriptional repressor GATA binding 1 | 4.070702 | 2.469209 | 0.020139435 | 0.172142234 |
| 9722 | NM_001126060NOS1AP | | nitric oxide synthase 1 adaptor protein | 4.072281 | 1.069575 | 0.021652467 | 0.182436363 |
| 3902 | NM_002286 LAG3 | | lymphocyte activating 3 | 4.086036 | 0.551787 | 0.02169314 | 0.182704642 |
| 339541 | NM_001145636C1orf228 | | chromosome 1 open reading frame 228 | 4.090618 | -0.095311 | 0.024759024 | 0.196599005 |
| 3216 | NM_018952 HOXB6 | | homeobox B6 | 4.091007 | 3.975645 | 0.018983267 | 0.163885176 |
| 2244 | NM_001184741FGB | | fibrinogen beta chain | 4.099438 | 5.672210 | 0.018574971 | 0.161370547 |
| 389840 | NM_001001671MAP3K15 | | mitogen-activated protein kinase kinase kinase 15 | 4.100360 | 3.641357 | 0.018923848 | 0.163440415 |
| 256987 | NM_001174071SERINC5 | | serine incorporator 5 | 4.104658 | 6.490453 | 0.018468431 | 0.160714976 |
| 1287 | NM_000495,N COL4A5 | | collagen type IV alpha 5 chain | 4.107461 | 7.577916 | 0.018383657 | 0.160111973 |
| 84217 | NM_001146192ZMYND12 | | zinc finger MYND-type containing 12 | 4.107986 | -0.676314 | 0.031640769 | 0.233618934 |
| 653641 | NM_001164404GOLGA6C | | golgin A6 family member C | 4.107986 | -1.262182 | 0.031640769 | 0.233618934 |
| 1281 | NM_000090 COL3A1 | | collagen type III alpha 1 chain | 4.113477 | -1.306995 | 0.033712064 | 0.238099055 |
| 50840 | NM_023922 TAS2R14 | | taste 2 receptor member 14 | 4.114032 | -0.217701 | 0.024307434 | 0.193756948 |
| 148808 | NM_181644 MFSD4A | | major facilitator superfamily domain containing 4A | 4.116829 | 1.380708 | 0.02029588 | 0.1730504 |
| 123036 | NM_001128595TC2N | | tandem C2 domains, nuclear | 4.118793 | 4.153511 | 0.018425644 | 0.160410118 |
| 89866 | NM_033127 SEC16B | | SEC16 homolog B, endoplasmic reticulum export factor | 4.119258 | 3.244932 | 0.018693331 | 0.162194442 |
| 54361 | NM_030761 WNT4 | | Wnt family member 4 | 4.129994 | 0.336691 | 0.020735376 | 0.175783302 |
| 56260 | NM_019607 C8orf44 | | chromosome 8 open reading frame 44 | 4.130122 | 1.433769 | 0.019827743 | 0.170040163 |
| 124961 | NM_153018 ZFP3 | | ZFP3 zinc finger protein | 4.132900 | 2.026328 | 0.019570277 | 0.168250697 |
| 106660613 | NR_003660,NR LINC00680-GUSB | | LINC00680-GUSBP4 readthrough | 4.133344 | -1.823205 | 0.044849939 | 0.290275655 |

| 84875 | NM_001317895PARP10 | poly(ADP-ribose) polymerase family member 10 | 4.135514 | 2.293936 | 0.018888553 | 0.163203724 |
| --- | --- | --- | --- | --- | --- | --- |
| 101927288 | NR_047502,NR MANEA-AS1 | MANEA antisense RNA 1 (head to head) | 4.138944 | -1.909468 | 0.047571201 | 0.298715722 |
| 102723672 | NR_134325 LOC102723672 | uncharacterized LOC102723672 | 4.138944 | -1.964288 | 0.047571201 | 0.298715722 |
| 285600 | NM_001145678KIAA0825 | KIAA0825 | 4.144738 | 0.780518 | 0.020437323 | 0.173612331 |
| 497661 | NM_001035005C18orf32 | chromosome 18 open reading frame 32 | 4.145064 | 4.746679 | 0.017855385 | 0.156433136 |
| 5256 | NM_000292 PHKA2 | phosphorylase kinase regulatory subunit alpha 2 | 4.146265 | 6.448673 | 0.017695984 | 0.155524292 |
| 145788 | NM_001198784C15orf65 | chromosome 15 open reading frame 65 | 4.146544 | 2.251381 | 0.018705729 | 0.162233962 |
| 7164 | NM_001003395TPD52L1 | tumor protein D52 like 1 | 4.147973 | 5.472899 | 0.017714055 | 0.155524292 |
| 5790 | NM_005608 PTPRCAP | protein tyrosine phosphatase, receptor type C associated pr | 4.151717 | -0.007387 | 0.022978168 | 0.185520456 |
| 645811 | NM_001143980CCDC154 | coiled-coil domain containing 154 | 4.153277 | -0.143191 | 0.023028082 | 0.185633622 |
| 644150 | NM_001080529WIPF3 | WAS/WASL interacting protein family member 3 | 4.171556 | 2.895814 | 0.018198086 | 0.158897174 |
| 55336 | NM_018378 FBXL8 | F-box and leucine rich repeat protein 8 | 4.174162 | 3.032940 | 0.017664451 | 0.155352542 |
| 388403 | NM_001005404YPEL2 | yippee like 2 | 4.182401 | 4.335677 | 0.017219036 | 0.15273403 |
| 79846 | NM_001039706CFAP69 | cilia and flagella associated protein 69 | 4.192494 | 2.917155 | 0.017488283 | 0.154065215 |
| 407043 | NR_029605 MIR7-1 | microRNA 7-1 | 4.193447 | -1.635594 | 0.034433792 | 0.241695853 |
| 57189 | NM_001080392KIAA1147 | KIAA1147 | 4.194010 | 6.616532 | 0.01685565 | 0.150478687 |
| 9185 | NM_001080975REPS2 | RALBP1 associated Eps domain containing 2 | 4.196113 | 2.742331 | 0.017707722 | 0.155524292 |
| 4306 | NM_000901,N NR3C2 | nuclear receptor subfamily 3 group C member 2 | 4.196954 | -0.374676 | 0.023010444 | 0.18556376 |
| 130733 | NM_001167959TMEM178A | transmembrane protein 178A | 4.200790 | -0.116465 | 0.022173873 | 0.184819135 |
| 440456 | NR_024386 PLEKHM1P1 | pleckstrin homology and RUN domain containing M1 pseu | 4.201719 | 4.442196 | 0.016855567 | 0.150478687 |
| 84698 | NM_001286547CAPS2 | calcyphosine 2 | 4.202501 | 2.649474 | 0.017353697 | 0.153533453 |
| 91947 | NM_183376 ARRDC4 | arrestin domain containing 4 | 4.203516 | 3.829720 | 0.016958202 | 0.151093491 |
| 254263 | NM_182553,NRCNIH2 | cornichon family AMPA receptor auxiliary protein 2 | 4.203542 | -0.006289 | 0.022208323 | 0.184819135 |
| 92359 | NM_139161,N CRB3 | crumbs 3, cell polarity complex component | 4.203910 | 2.107671 | 0.017704929 | 0.155524292 |
| 51233 | NM_016449 DRICH1 | aspartate rich 1 | 4.212713 | -2.082310 | 0.039579885 | 0.267202974 |
| 102465879 | NR_107052 MIR8085 | microRNA 8085 | 4.212713 | -1.604877 | 0.039579885 | 0.267202974 |
| 100190938 | NR_024461,NR RAMP2-AS1 | RAMP2 antisense RNA 1 | 4.224944 | -2.041681 | 0.041421069 | 0.276563853 |
| 6657 | NM_003106 SOX2 | SRY-box 2 | 4.225337 | 2.517836 | 0.01720438 | 0.15273403 |
| 340277 | NM_001127364FAM221A | family with sequence similarity 221 member A | 4.226126 | 0.220227 | 0.021735074 | 0.182908872 |
| 23784 | NM_001136213POTEH | POTE ankyrin domain family member H | 4.232495 | -1.067902 | 0.030272862 | 0.226799765 |
| 1741 | NM_001166278DLG3 | discs large MAGUK scaffold protein 3 | 4.232662 | 4.900301 | 0.016316514 | 0.147190186 |
| 5016 | NM_002557 OVGP1 | oviductal glycoprotein 1 | 4.233965 | 2.277504 | 0.017090808 | 0.151922371 |
| 79899 | NM_001160167PRR5L | proline rich 5 like | 4.237136 | 3.907001 | 0.016406859 | 0.147811798 |
| 7767 | NM_001321645ZNF224 | zinc finger protein 224 | 4.239114 | 5.599009 | 0.016152356 | 0.146302603 |
| 440104 | NR_036476,NR TMEM198B | transmembrane protein 198B (pseudogene) | 4.241229 | 4.623928 | 0.016185581 | 0.146382587 |
| 2628 | NM_001321015GATM | glycine amidinotransferase | 4.246611 | 4.278876 | 0.016198682 | 0.146382587 |
| 342909 | NM_001037813ZNF284 | zinc finger protein 284 | 4.249329 | 2.624077 | 0.016536033 | 0.148458269 |
| 11155 | NM_001080114LDB3 | LIM domain binding 3 | 4.249513 | -0.502089 | 0.023434718 | 0.188324453 |
| 728448 | NR_003929,NR PPIEL | peptidylprolyl isomerase E like pseudogene | 4.253765 | 3.341986 | 0.016195901 | 0.146382587 |
| 80737 | NM_025258 VWA7 | von Willebrand factor A domain containing 7 | 4.261370 | 2.264556 | 0.016660499 | 0.14924508 |
| 80863 | NM_030651 PRRT1 | proline rich transmembrane protein 1 | 4.261462 | 1.025617 | 0.018008807 | 0.157577065 |
| 51132 | NM_016120,N RLIM | ring finger protein, LIM domain interacting | 4.265552 | 6.242368 | 0.015729492 | 0.146302603 |
| 101929402 | NR_126027 EXTL3-AS1 | EXTL3 antisense RNA 1 | 4.265594 | -1.507958 | 0.032316502 | 0.233618934 |

| 90673 | NM_001276318PPP1R3E | protein phosphatase 1 regulatory subunit 3E | 4.271195 | 3.679097 | 0.01585809 | 0.146302603 |
| --- | --- | --- | --- | --- | --- | --- |
| 100131564 | NR_034089 CCDC18-AS1 | CCDC18 antisense RNA 1 | 4.274820 | 4.096558 | 0.015740976 | 0.146302603 |
| 29799 | NM_013313,NRYPEL1 | yippee like 1 | 4.277634 | 0.960159 | 0.017882147 | 0.156601272 |
| 7096 | NM_003263 TLR1 | toll like receptor 1 | 4.278035 | 2.067807 | 0.016443434 | 0.14789495 |
| 56127 | NM_019119 PCDHB9 | protocadherin beta 9 | 4.279695 | -2.059026 | 0.049814168 | 0.30584923 |
| 283981 | NR_027231_chr LINC00685 | long intergenic non-protein coding RNA 685 | 4.279695 | -2.250057 | 0.049814168 | 0.30584923 |
| 1490 | NM_001901 CTGF | connective tissue growth factor | 4.283069 | 6.441669 | 0.01542514 | 0.145693618 |
| 83886 | NM_001318395PRSS27 | protease, serine 27 | 4.289099 | 0.624029 | 0.017747782 | 0.15575429 |
| 84101 | NM_001042403USP44 | ubiquitin specific peptidase 44 | 4.289170 | 0.512955 | 0.020387006 | 0.173469852 |
| 100101467 | NM_001112734ZSCAN30 | zinc finger and SCAN domain containing 30 | 4.289628 | 3.060212 | 0.015915049 | 0.146302603 |
| 60680 | NM_001172673CELF5 | CUGBP Elav-like family member 5 | 4.294578 | 1.356449 | 0.016911285 | 0.150780142 |
| 100288152 | NR_125375 SLC9A3-AS1 | SLC9A3 antisense RNA 1 | 4.296333 | 2.866531 | 0.015782751 | 0.146302603 |
| 348174 | NM_001136214CLEC18A | C-type lectin domain family 18 member A | 4.296760 | -1.664297 | 0.03679012 | 0.253330107 |
| 109136579 | NR_145459 TALAM1 | MALAT1 antisense transcript | 4.297979 | 0.953988 | 0.017441679 | 0.154065215 |
| 7644 | NM_001300951ZNF91 | zinc finger protein 91 | 4.300558 | -0.142844 | 0.022971639 | 0.185520456 |
| 9498 | NM_001039960SLC4A8 | solute carrier family 4 member 8 | 4.303371 | 2.099480 | 0.016574436 | 0.148631913 |
| 105369343 | NR_133638,NR MIR194-2HG | MIR194-2 host gene | 4.303955 | -0.303750 | 0.020374914 | 0.173438313 |
| 6480 | NM_003032,N ST6GAL1 | ST6 beta-galactoside alpha-2,6-sialyltransferase 1 | 4.305216 | 4.355803 | 0.015213571 | 0.144024122 |
| 120892 | NM_198578 LRRK2 | leucine rich repeat kinase 2 | 4.309268 | 5.722124 | 0.015058844 | 0.143280677 |
| 8771 | NM_003823 TNFRSF6B | TNF receptor superfamily member 6b | 4.310539 | -0.684241 | 0.026298266 | 0.205430373 |
| 347746 | NR_022011 PWARSN | Prader Willi/Angelman region RNA, SNRPN neighbor | 4.321659 | 1.069983 | 0.017218375 | 0.15273403 |
| 442213 | NM_001013732PTCHD4 | patched domain containing 4 | 4.325322 | -0.833142 | 0.023214157 | 0.18691508 |
| 727800 | NM_031297 RNF208 | ring finger protein 208 | 4.328188 | 0.400015 | 0.019279403 | 0.166095146 |
| 101927258 | NR_121120,NR LINC01474 | long intergenic non-protein coding RNA 1474 | 4.329936 | 1.162914 | 0.01650369 | 0.148257713 |
| 9723 | NM_001178129SEMA3E | semaphorin 3E | 4.332447 | 2.819582 | 0.015226784 | 0.144078619 |
| 2696 | NM_000164,N GIPR | gastric inhibitory polypeptide receptor | 4.336584 | 2.995886 | 0.015170308 | 0.143737334 |
| 342096 | NM_001038640GOLGA6A | golgin A6 family member A | 4.340235 | -2.207214 | 0.040082012 | 0.270063977 |
| 148898 | NR_033690,NR ZNF436-AS1 | ZNF436 antisense RNA 1 | 4.351095 | 1.525006 | 0.015995058 | 0.146302603 |
| 5465 | NM_001001928PPARA | peroxisome proliferator activated receptor alpha | 4.351434 | 4.028489 | 0.014607749 | 0.140279145 |
| 100996255 | NR_102756 LINC02256 | long intergenic non-protein coding RNA 2256 | 4.353955 | 1.046957 | 0.016506527 | 0.148257713 |
| 22797 | NM_001018058TFEC | transcription factor EC | 4.356245 | -0.840281 | 0.023509308 | 0.188777191 |
| 100533107 | NR_037882 RTEL1-TNFRSF6B | RTEL1-TNFRSF6B readthrough (NMD candidate) | 4.364879 | 3.311272 | 0.014515719 | 0.139524927 |
| 10581 | NM_006435 IFITM2 | interferon induced transmembrane protein 2 | 4.366796 | 5.087494 | 0.014263809 | 0.137724153 |
| 102465454 | NR_106816 MIR6758 | microRNA 6758 | 4.369119 | -2.228535 | 0.04226773 | 0.280291436 |
| 8082 | NM_001135823SSPN | sarcospan | 4.373355 | 1.358960 | 0.016226393 | 0.146504993 |
| 825 | NM_000070,N CAPN3 | calpain 3 | 4.373877 | 0.647674 | 0.01630769 | 0.147174766 |
| 200958 | NM_001282506MUC20 | mucin 20, cell surface associated | 4.378896 | 0.944296 | 0.015852595 | 0.146302603 |
| 22901 | NM_001267727ARSG | arylsulfatase G | 4.379579 | 2.605025 | 0.014772928 | 0.14153683 |
| 137872 | NM_144650 ADHFE1 | alcohol dehydrogenase, iron containing 1 | 4.381163 | 0.975281 | 0.01577871 | 0.146302603 |
| 286256 | NM_178536 LCN12 | lipocalin 12 | 4.381789 | -0.682059 | 0.020905305 | 0.177072473 |
| 102465448 | NR_106806 MIR6748 | microRNA 6748 | 4.387857 | -1.508795 | 0.028034104 | 0.214454678 |
| 3696 | NM_002214 ITGB8 | integrin subunit beta 8 | 4.388028 | 2.043141 | 0.014764455 | 0.141521197 |
| 10481 | NM_006361 HOXB13 | homeobox B13 | 4.396349 | 3.377995 | 0.014143784 | 0.137027842 |

| 57643 | NM_020883 | ZSWIM5 | zinc finger SWIM-type containing 5 | 4.399615 | 3.093093 | 0.01425668 | 0.137724153 |
| --- | --- | --- | --- | --- | --- | --- | --- |
| 9390 | NM_004256 | SLC22A13 | solute carrier family 22 member 13 | 4.405112 | -2.368298 | 0.044985511 | 0.290880056 |
| 100422936 | NR_036108 | MIR3153 | microRNA 3153 | 4.405112 | -2.039392 | 0.044985511 | 0.290880056 |
| 100874253 | NR_047498 | LINC00853 | long intergenic non-protein coding RNA 853 | 4.405112 | -1.890357 | 0.044985511 | 0.290880056 |
| 9936 | NM_001198763CD302 | | CD302 molecule | 4.408208 | 4.475839 | 0.013753037 | 0.13425275 |
| 7582 | NM_001305033ZNF33B | | zinc finger protein 33B | 4.411336 | 4.957876 | 0.013662909 | 0.133435913 |
| 2970 | NR_002206,NR GTF2IP1 | | general transcription factor IIi pseudogene 1 | 4.415292 | 6.903065 | 0.013542452 | 0.13250975 |
| 54800 | NM_001349413KLHL24 | | kelch like family member 24 | 4.420979 | 4.691411 | 0.013559451 | 0.132613352 |
| 11184 | NM_001042600MAP4K1 | | mitogen-activated protein kinase kinase kinase kinase 1 | 4.426766 | 0.967121 | 0.015176328 | 0.143737334 |
| 642658 | NM_001080514SCX | | scleraxis bHLH transcription factor | 4.431386 | -0.368975 | 0.018481967 | 0.160765135 |
| 6019 | NM_001329191RLN2 | | relaxin 2 | 4.431757 | -0.786082 | 0.026126751 | 0.204962231 |
| 28968 | NM_014037 SLC6A16 | | solute carrier family 6 member 16 | 4.432550 | 2.379901 | 0.014103755 | 0.136965338 |
| 5623 | NM_004158 PSPN | | persephin | 4.435276 | -1.665192 | 0.029056273 | 0.219753043 |
| 23127 | NM_001303420COLGALT2 | | collagen beta(1-O)galactosyltransferase 2 | 4.435877 | -0.258582 | 0.020284917 | 0.173028251 |
| 3299 | NM_001040667HSF4 | | heat shock transcription factor 4 | 4.436148 | 4.424219 | 0.013378891 | 0.131344264 |
| 2153 | NM_000130 F5 | | coagulation factor V | 4.436332 | 4.913742 | 0.013321695 | 0.130907014 |
| 283869 | NM_001099456NPW | | neuropeptide W | 4.440731 | -0.831883 | 0.021782601 | 0.183085373 |
| 84866 | NM_001144034TMEM25 | | transmembrane protein 25 | 4.440881 | -1.756760 | 0.036847945 | 0.253475136 |
| 1628 | NM_001352 DBP | | D-box binding PAR bZIP transcription factor | 4.447754 | 2.513734 | 0.013855895 | 0.135001974 |
| 338557 | NM_001195755FFAR4 | | free fatty acid receptor 4 | 4.449753 | 0.512258 | 0.017401685 | 0.153851081 |
| 30832 | NM_014594 ZNF354C | | zinc finger protein 354C | 4.450524 | 2.560968 | 0.013836349 | 0.134938649 |
| 7732 | NM_007148 RNF112 | | ring finger protein 112 | 4.453004 | 0.355046 | 0.015799353 | 0.146302603 |
| 79800 | NM_001104586CARF | | calcium responsive transcription factor | 4.457847 | 2.825055 | 0.013501045 | 0.132292335 |
| 8029 | NM_001081 CUBN | | cubilin | 4.461421 | -0.240821 | 0.020261462 | 0.172931031 |
| 100129583 | NM_001136570FAM47E | | family with sequence similarity 47 member E | 4.462455 | -1.217642 | 0.022964829 | 0.185520456 |
| 100505585 | NR_135626 LOC100505585 | | uncharacterized LOC100505585 | 4.465931 | -0.127450 | 0.017286112 | 0.153000956 |
| 158131 | NM_012364 OR1Q1 | | olfactory receptor family 1 subfamily Q member 1 | 4.480793 | -2.100423 | 0.038568237 | 0.262492711 |
| 63950 | NM_032110 DMRTA2 | | DMRT like family A2 | 4.482035 | -0.752543 | 0.026804893 | 0.207974196 |
| 8519 | NM_003641 IFITM1 | | interferon induced transmembrane protein 1 | 4.484007 | 1.198794 | 0.01413537 | 0.137027842 |
| 55450 | NM_018584 CAMK2N1 | | calcium/calmodulin dependent protein kinase II inhibitor 1 | 4.487102 | 5.925037 | 0.012657106 | 0.125810786 |
| 7148 | NM_019105,N TNXB | | tenascin XB | 4.489210 | 2.905397 | 0.013107362 | 0.129512912 |
| 100270804 | NR_026885 LOC100270804 | | uncharacterized LOC100270804 | 4.490730 | 1.232566 | 0.014281765 | 0.137724153 |
| 8578 | NM_003693,N SCARF1 | | scavenger receptor class F member 1 | 4.490877 | -0.816019 | 0.020233479 | 0.172803268 |
| 257044 | NM_001130957CATSPERE | | catsper channel auxiliary subunit epsilon | 4.494956 | -1.379667 | 0.024734938 | 0.196483175 |
| 65987 | NM_001282406KCTD14 | | potassium channel tetramerization domain containing 14 | 4.498013 | 1.249568 | 0.015037671 | 0.143210966 |
| 55603 | NM_017633 FAM46A | | family with sequence similarity 46 member A | 4.503984 | 4.672203 | 0.012516524 | 0.125256075 |
| 65012 | NM_133489 SLC26A10 | | solute carrier family 26 member 10 | 4.505399 | -0.272756 | 0.017486347 | 0.154065215 |
| 100130015 | NR_027335,NR URAHP | | urate (hydroxyiso-) hydrolase, pseudogene | 4.513300 | 0.527254 | 0.014879098 | 0.142191661 |
| 84941 | NM_001291274HSH2D | | hematopoietic SH2 domain containing | 4.513333 | 0.727272 | 0.014275318 | 0.137724153 |
| 56978 | NM_001099403PRDM8 | | PR/SET domain 8 | 4.518193 | -0.111429 | 0.019031536 | 0.164233342 |
| 494513 | NM_001042702DFNB59 | | deafness, autosomal recessive 59 | 4.524744 | 1.348440 | 0.013584421 | 0.132794776 |
| 441381 | NM_001024678LRRC24 | | leucine rich repeat containing 24 | 4.526849 | 1.869722 | 0.012944114 | 0.128048299 |
| 100874366 | NR_047507 HOXC13-AS | | HOXC13 antisense RNA | 4.529812 | -0.072605 | 0.01780098 | 0.156022572 |

| 677810 | NR_003016 | SNORA26 | small nucleolar RNA, H/ACA box 26 | 4.530528 | -2.344028 | 0.040683222 | 0.272853118 |
| --- | --- | --- | --- | --- | --- | --- | --- |
| 105371730 | NR_136413 | LOC105371730 | uncharacterized LOC105371730 | 4.530528 | -1.855485 | 0.040683222 | 0.272853118 |
| 105375800 | NR_136279 | LOC105375800 | chromosome 12 open reading frame 49 pseudogene | 4.530528 | -2.524157 | 0.040683222 | 0.272853118 |
| 653720 | NM_001282468GOLGA8M | | golgin A8 family member M | 4.531068 | 0.869281 | 0.013974373 | 0.136028192 |
| 155060 | NR_036573 LOC155060 | | AI894139 pseudogene | 4.531177 | 3.977151 | 0.012276281 | 0.123149793 |
| 3490 | NM_001253835IGFBP7 | | insulin like growth factor binding protein 7 | 4.535375 | 5.531516 | 0.012100347 | 0.121696164 |
| 3590 | NM_001142784IL11RA | | interleukin 11 receptor subunit alpha | 4.539002 | 3.080828 | 0.012347278 | 0.123801963 |
| 360132 | NR_003949,NR FKBP9P1 | | FK506 binding protein 9 pseudogene 1 | 4.539999 | 1.671249 | 0.013214777 | 0.1300417 |
| 23321 | NM_001130067TRIM2 | | tripartite motif containing 2 | 4.543064 | 3.785646 | 0.012168558 | 0.122187677 |
| 643401 | NR_038848 LINC01021 | | long intergenic non-protein coding RNA 1021 | 4.551550 | 3.842453 | 0.012101958 | 0.121696164 |
| 100507218 | NR_040017 RNF157-AS1 | | RNF157 antisense RNA 1 | 4.554182 | -1.727984 | 0.025212922 | 0.199361347 |
| 317760 | NR_037933 ADAM20P1 | | ADAM metallopeptidase domain 20 pseudogene 1 | 4.558155 | -1.469500 | 0.023427372 | 0.188324453 |
| 100132356 | NR_034127 LOC100132356 | | uncharacterized LOC100132356 | 4.561251 | -1.119938 | 0.022072778 | 0.184819135 |
| 645553 | NR_135234 IGFL2-AS1 | | IGFL2 antisense RNA 1 | 4.564738 | 4.710008 | 0.012070439 | 0.12149734 |
| 729603 | NR_003288 LOC729603 | | calcineurin like EF-hand protein 1 pseudogene | 4.565764 | -0.724490 | 0.020158232 | 0.172231736 |
| 100216546 | NR_039981 LINC01004 | | long intergenic non-protein coding RNA 1004 | 4.566410 | 2.656156 | 0.012159058 | 0.122151582 |
| 100133005 | NR_146066 RASA4DP | | RAS p21 protein activator 4CD, pseudogene | 4.583120 | 1.729450 | 0.012702506 | 0.126015418 |
| 54627 | NM_019090 MAP10 | | microtubule associated protein 10 | 4.586589 | 2.284829 | 0.01257366 | 0.125356208 |
| 619373 | NM_001100916MBOAT4 | | membrane bound O-acyltransferase domain containing 4 | 4.589988 | -1.578691 | 0.023570697 | 0.189123298 |
| 101927895 | NR_135114,NR LOC101927895 | | uncharacterized LOC101927895 | 4.592468 | -2.080230 | 0.035233806 | 0.246036821 |
| 4242 | NM_001166343MFNG | | MFNG O-fucosylpeptide 3-beta-N-acetylglucosaminyltransf | 4.595251 | 1.412185 | 0.012708154 | 0.126015418 |
| 100534593 | NR_037945 STX16-NPEPL1 | | STX16-NPEPL1 readthrough (NMD candidate) | 4.595337 | 0.464299 | 0.013426642 | 0.131750514 |
| 8786 | NM_001286485RGS11 | | regulator of G protein signaling 11 | 4.596079 | 2.467150 | 0.011838295 | 0.119567935 |
| 100192386 | NR_024389 FLJ16779 | | uncharacterized LOC100192386 | 4.600170 | 0.587188 | 0.013158967 | 0.129739388 |
| 30813 | NM_001256271VSX1 | | visual system homeobox 1 | 4.608203 | 1.013135 | 0.013121479 | 0.129533006 |
| 8620 | NM_001320296NPFF | | neuropeptide FF-amide peptide precursor | 4.608717 | 1.009512 | 0.01277774 | 0.126644727 |
| 1999 | NM_001114309ELF3 | | E74 like ETS transcription factor 3 | 4.615029 | 8.150063 | 0.011164135 | 0.116103634 |
| 171177 | NM_133639 RHOV | | ras homolog family member V | 4.615975 | 4.144980 | 0.011334532 | 0.117602386 |
| 221262 | NR_028595 CCDC162P | | coiled-coil domain containing 162, pseudogene | 4.627201 | 0.180308 | 0.014623397 | 0.14036425 |
| 30061 | NM_014585 SLC40A1 | | solute carrier family 40 member 1 | 4.632090 | 5.605668 | 0.011026761 | 0.114963988 |
| 57134 | NM_001289010MAN1C1 | | mannosidase alpha class 1C member 1 | 4.632629 | 0.663169 | 0.013306009 | 0.130815016 |
| 5364 | NM_001130082PLXNB1 | | plexin B1 | 4.633023 | 6.240659 | 0.010998581 | 0.114843537 |
| 57216 | NM_020335 VANGL2 | | VANGL planar cell polarity protein 2 | 4.636490 | 3.683918 | 0.011284941 | 0.117242097 |
| 51557 | NM_001143940LGSN | | lengsin, lens protein with glutamine synthetase domain | 4.639937 | 5.849960 | 0.010938004 | 0.114268998 |
| 100128076 | NR_015444 LOC100128076 | | protein tyrosine phosphatase pseudogene | 4.642485 | -0.872845 | 0.0200758 | 0.171680039 |
| 5251 | NM_000444,N PHEX | | phosphate regulating endopeptidase homolog, X-linked | 4.650274 | 2.053952 | 0.01195133 | 0.120561714 |
| 1536 | NM_000397 CYBB | | cytochrome b-245 beta chain | 4.651945 | -0.485567 | 0.01806615 | 0.157878462 |
| 283463 | NM_173600 MUC19 | | mucin 19, oligomeric | 4.651945 | -0.984716 | 0.01806615 | 0.157878462 |
| 677767 | NR_003001 SCARNA7 | | small Cajal body-specific RNA 7 | 4.655945 | -2.320160 | 0.036844159 | 0.253475136 |
| 284009 | NR_028335 LOC284009 | | uncharacterized LOC284009 | 4.657365 | -1.342425 | 0.022268105 | 0.184819135 |
| 10551 | NM_006408 AGR2 | | anterior gradient 2, protein disulphide isomerase family me | 4.659411 | 8.398769 | 0.010702081 | 0.112144147 |
| 728743 | NR_027237 LOC728743 | | zinc finger protein pseudogene | 4.662167 | 2.592043 | 0.011101073 | 0.115505886 |
| 100507419 | NR_123723 MMP25-AS1 | | MMP25 antisense RNA 1 | 4.665306 | 2.071776 | 0.011336677 | 0.117602386 |

| 165082 | NM_001145168ADGRF3 | adhesion G protein-coupled receptor F3 | 4.676822 | -1.042088 | 0.017973623 | 0.157335755 |
| --- | --- | --- | --- | --- | --- | --- |
| 56144 | NM_018907,N PCDHA4 | protocadherin alpha 4 | 4.678385 | 4.193023 | 0.010731501 | 0.112338612 |
| 221481 | NM_001286574ARMC12 | armadillo repeat containing 12 | 4.678833 | -0.727533 | 0.016691726 | 0.149402142 |
| 6556 | NM_000578 SLC11A1 | solute carrier family 11 member 1 | 4.684553 | -1.469689 | 0.021042707 | 0.177951105 |
| 100506801 | NR_038404 LOC100506801 | uncharacterized LOC100506801 | 4.694846 | 0.273979 | 0.012675927 | 0.125890073 |
| 144321 | NM_001270396GLIPR1L2 | GLI pathogenesis related 1 like 2 | 4.701033 | -0.541581 | 0.016576931 | 0.148631913 |
| 10896 | NM_022375 OCLM | oculomedin | 4.703897 | -0.118936 | 0.014843671 | 0.142017273 |
| 3205 | NM_152739 HOXA9 | homeobox A9 | 4.714213 | 0.725619 | 0.011960814 | 0.120569905 |
| 729975 | NR_034159 LINC01530 | long intergenic non-protein coding RNA 1530 | 4.716990 | -1.402378 | 0.024095046 | 0.192583474 |
| 6857 | NM_001135805SYT1 | synaptotagmin 1 | 4.722217 | 5.344611 | 0.010153984 | 0.107489849 |
| 8357 | NM_003536 HIST1H3H | histone cluster 1 H3 family member h | 4.736431 | -0.394764 | 0.016787396 | 0.150063654 |
| 114757 | NM_134268 CYGB | cytoglobin | 4.741499 | 0.905675 | 0.011544974 | 0.118301114 |
| 100526820 | NR_037593 CAHM | colon adenocarcinoma hypermethylated (non-protein codin | 4.745849 | -1.341230 | 0.02242318 | 0.184819135 |
| 283848 | NM_001190201CES4A | carboxylesterase 4A | 4.746953 | 2.746666 | 0.010440852 | 0.109980851 |
| 102723166 | NR_110370 STAM-AS1 | STAM antisense RNA 1 (head to head) | 4.747752 | -0.794701 | 0.019955481 | 0.170965144 |
| 55268 | NM_001198961ECHDC2 | enoyl-CoA hydratase domain containing 2 | 4.747789 | 3.688509 | 0.010108004 | 0.107271384 |
| 11156 | NM_007079,N PTP4A3 | protein tyrosine phosphatase type IVA, member 3 | 4.760598 | 3.330679 | 0.010031889 | 0.106688754 |
| 5343 | NM_001032392PLGLB1 | plasminogen-like B1 | 4.765620 | 0.113374 | 0.011587601 | 0.118301114 |
| 643008 | NM_001162995SMIM5 | small integral membrane protein 5 | 4.766149 | 0.157851 | 0.011837847 | 0.119567935 |
| 100505648 | NR_040058 RAD51-AS1 | RAD51 antisense RNA 1 (head to head) | 4.771054 | 1.604445 | 0.010717562 | 0.112249505 |
| 100846999 | NR_049831_chr MIR4444-2 | microRNA 4444-2 | 4.774173 | -0.599886 | 0.015006868 | 0.143049334 |
| 10252 | NM_001258038SPRY1 | sprouty RTK signaling antagonist 1 | 4.776224 | 2.758018 | 0.009986518 | 0.106315556 |
| 101928062 | NR_120459,NR LINC01481 | long intergenic non-protein coding RNA 1481 | 4.790378 | 1.467994 | 0.01081587 | 0.113107319 |
| 63027 | NM_001286455SLC22A23 | solute carrier family 22 member 23 | 4.790811 | 5.921586 | 0.00950014 | 0.102030323 |
| 2877 | NM_002083,NRGPX2 | glutathione peroxidase 2 | 4.793064 | 8.532281 | 0.009449305 | 0.101748504 |
| 399821 | NR_033847 FLJ37035 | uncharacterized LOC399821 | 4.794835 | 0.370367 | 0.012683341 | 0.125890073 |
| 770 | NM_001217,NRCA11 | carbonic anhydrase 11 | 4.804321 | 4.000683 | 0.009544616 | 0.102348565 |
| 1438 | NM_001161529CSF2RA | colony stimulating factor 2 receptor alpha subunit | 4.809358 | 2.525697 | 0.010325724 | 0.1089177 |
| 57586 | NM_001247987SYT13 | synaptotagmin 13 | 4.811731 | 5.800740 | 0.009328548 | 0.100552902 |
| 100506380 | NR_103858 LINC01006 | long intergenic non-protein coding RNA 1006 | 4.816924 | -1.142443 | 0.01690757 | 0.150780142 |
| 1015 | NM_001144663CDH17 | cadherin 17 | 4.819374 | 5.725756 | 0.009255999 | 0.099927105 |
| 3268 | NM_006076 AGFG2 | ArfGAP with FG repeats 2 | 4.822375 | 5.142483 | 0.009273836 | 0.100067445 |
| 100529145 | NR_037709 TEN1-CDK3 | TEN1-CDK3 readthrough (NMD candidate) | 4.838500 | 3.696428 | 0.009238639 | 0.099791775 |
| 100508120 | NR_046229 GMDS-AS1 | GMDS antisense RNA 1 (head to head) | 4.841503 | 0.317177 | 0.012123269 | 0.12185122 |
| 9788 | NM_001282971MTSS1 | MTSS1, I-BAR domain containing | 4.844707 | 3.118332 | 0.009380996 | 0.101065578 |
| 55779 | NM_001164496CFAP44 | cilia and flagella associated protein 44 | 4.844955 | 4.684907 | 0.009090921 | 0.098608124 |
| 1128 | NM_000738 CHRM1 | cholinergic receptor muscarinic 1 | 4.845070 | -2.894914 | 0.041376294 | 0.2763541 |
| 165186 | NM_001321538TOGARAM2 | TOG array regulator of axonemal microtubules 2 | 4.845070 | -2.727949 | 0.041376294 | 0.2763541 |
| 8645 | NM_003740 KCNK5 | potassium two pore domain channel subfamily K member 5 | 4.850385 | 4.235794 | 0.00906714 | 0.098608124 |
| 112464 | NM_145040 CAVIN3 | caveolae associated protein 3 | 4.850487 | 2.131256 | 0.010095883 | 0.107203976 |
| 400793 | NM_001085375C1orf226 | chromosome 1 open reading frame 226 | 4.852091 | 2.339828 | 0.0095659 | 0.102470558 |
| 285505 | NR_147407,NR LOC285505 | uncharacterized LOC285505 | 4.859497 | -1.215161 | 0.018828856 | 0.162892044 |
| 728537 | NM_001351368LINC01125 | long intergenic non-protein coding RNA 1125 | 4.859497 | -1.509468 | 0.018828856 | 0.162892044 |

| 6565 | NM_001145998SLC15A2 | solute carrier family 15 member 2 | 4.868390 | -0.079225 | 0.012587133 | 0.125356208 |
| --- | --- | --- | --- | --- | --- | --- |
| 57835 | NM_021196,N SLC4A5 | solute carrier family 4 member 5 | 4.874234 | 2.607391 | 0.009146812 | 0.099058537 |
| 55357 | NM_001267571TBC1D2 | TBC1 domain family member 2 | 4.876305 | 5.182973 | 0.008817206 | 0.096652838 |
| 54997 | NM_001168325TESC | tescalcin | 4.878231 | 7.418653 | 0.008748393 | 0.096153298 |
| 100128126 | NR_038406 STAU2-AS1 | STAU2 antisense RNA 1 | 4.880254 | -2.496354 | 0.034864293 | 0.243637802 |
| 101928617 | NR_135036,NR LOC101928617 | uncharacterized LOC101928617 | 4.880254 | -2.078722 | 0.034864293 | 0.243637802 |
| 102238594 | NR_108100 SPACA6P-AS | SPACA6P antisense RNA | 4.882735 | -0.288585 | 0.013515887 | 0.132375062 |
| 256329 | NM_173573 LMNTD2 | lamin tail domain containing 2 | 4.886961 | 2.581670 | 0.009185483 | 0.099321332 |
| 286 | NM_000037,N ANK1 | ankyrin 1 | 4.895223 | 3.104705 | 0.008970552 | 0.09786702 |
| 79925 | NM_024867,N SPEF2 | sperm flagellar 2 | 4.897535 | 3.389596 | 0.008863754 | 0.097082464 |
| 8714 | NM_001144070ABCC3 | ATP binding cassette subfamily C member 3 | 4.908931 | 9.898636 | 0.008494407 | 0.094530216 |
| 51147 | NM_001127582ING4 | inhibitor of growth family member 4 | 4.918377 | 4.256746 | 0.00854428 | 0.094530216 |
| 25854 | NM_001006655FAM149A | family with sequence similarity 149 member A | 4.921807 | 2.058144 | 0.009014466 | 0.098190749 |
| 57731 | NM_020971,N SPTBN4 | spectrin beta, non-erythrocytic 4 | 4.927380 | 2.538721 | 0.008877504 | 0.097159349 |
| 100289178 | NR_040077 GNG12-AS1 | GNG12 antisense RNA 1 | 4.927490 | -1.964322 | 0.027046234 | 0.209375505 |
| 5662 | NM_001270965PSD | pleckstrin and Sec7 domain containing | 4.930523 | -0.054945 | 0.011779344 | 0.119205343 |
| 7869 | NM_001005914SEMA3B | semaphorin 3B | 4.943454 | 5.486157 | 0.008278947 | 0.094196925 |
| 149428 | NM_001159642BNIPL | BCL2 interacting protein like | 4.944111 | -1.806550 | 0.024549514 | 0.19531027 |
| 126823 | NM_001007255KLHDC9 | kelch domain containing 9 | 4.949019 | 0.639139 | 0.010112603 | 0.107271384 |
| 2556 | NM_000808 GABRA3 | gamma-aminobutyric acid type A receptor alpha3 subunit | 4.951334 | 1.260480 | 0.009618435 | 0.102926713 |
| 340544 | NR_038978 MORF4L2-AS1 | MORF4L2 antisense RNA 1 | 4.952814 | -0.283252 | 0.012599102 | 0.125371035 |
| 146 | NM_000678 ADRA1D | adrenoceptor alpha 1D | 4.954029 | 2.535905 | 0.008675147 | 0.095551336 |
| 7035 | NM_001032281TFPI | tissue factor pathway inhibitor | 4.954716 | 7.127370 | 0.008162001 | 0.093276785 |
| 4059 | NM_001013257BCAM | basal cell adhesion molecule (Lutheran blood group) | 4.955572 | 6.834013 | 0.008160138 | 0.093276785 |
| 2155 | NM_000131,N F7 | coagulation factor VII | 4.976485 | 0.343550 | 0.009518853 | 0.102178242 |
| 158055 | NM_152571 C9orf163 | chromosome 9 open reading frame 163 | 4.980315 | -0.789770 | 0.014140051 | 0.137027842 |
| 78989 | NM_001255982COLEC11 | collectin subfamily member 11 | 4.980963 | -0.312321 | 0.012426789 | 0.124418264 |
| 728577 | NM_001201380CNTNAP3B | contactin associated protein-like 3B | 4.987248 | 5.513574 | 0.007953012 | 0.09119072 |
| 10396 | NM_001105529ATP8A1 | ATPase phospholipid transporting 8A1 | 4.991422 | 1.612476 | 0.008813359 | 0.096652838 |
| 25849 | NM_015393 PARM1 | prostate androgen-regulated mucin-like protein 1 | 4.993766 | 4.247502 | 0.007995327 | 0.091523703 |
| 644634 | NM_001348147FAM231D | family with sequence similarity 231 member D | 4.994251 | -1.047803 | 0.016874492 | 0.150581913 |
| 1733 | NM_000792,N DIO1 | iodothyronine deiodinase 1 | 4.994974 | -3.082475 | 0.044697393 | 0.289577333 |
| 4588 | NM_005961 MUC6 | mucin 6, oligomeric mucus/gel-forming | 4.994974 | -3.082475 | 0.044697393 | 0.289577333 |
| 84249 | NM_032289 PSD2 | pleckstrin and Sec7 domain containing 2 | 4.994974 | -3.082475 | 0.044697393 | 0.289577333 |
| 283140 | NR_126004 LOC283140 | uncharacterized LOC283140 | 4.994974 | -2.824765 | 0.044697393 | 0.289577333 |
| 284365 | NR_038359 LINC01869 | long intergenic non-protein coding RNA 1869 | 4.994974 | -2.858896 | 0.044697393 | 0.289577333 |
| 100847035 | NR_049839 MIR548AR | microRNA 548ar | 4.994974 | -2.824765 | 0.044697393 | 0.289577333 |
| 170302 | NM_139058 ARX | aristaless related homeobox | 4.995129 | 0.929197 | 0.009064129 | 0.098608124 |
| 3213 | NM_001330322HOXB3 | homeobox B3 | 4.999847 | 4.965221 | 0.007886628 | 0.090479697 |
| 221935 | NM_001079653SDK1 | sidekick cell adhesion molecule 1 | 5.000350 | 0.895795 | 0.010646185 | 0.111614971 |
| 643669 | NM_001243212CCER2 | coiled-coil glutamate rich protein 2 | 5.005918 | 0.903984 | 0.008984903 | 0.09792029 |
| 693216 | NR_030360 MIR631 | microRNA 631 | 5.011432 | -2.728100 | 0.036672434 | 0.252856432 |
| 100188954 | NR_024130 DNMBP-AS1 | DNMBP antisense RNA 1 | 5.011432 | -2.728100 | 0.036672434 | 0.252856432 |

| 58510 | NM_021232 | PRODH2 | proline dehydrogenase 2 | 5.011723 | 1.067508 | 0.008774646 | 0.09639063 |
| --- | --- | --- | --- | --- | --- | --- | --- |
| 730092 | NR_003370 | RRN3P1 | RRN3 homolog, RNA polymerase I transcription factor pseu | 5.013352 | 3.094775 | 0.007962616 | 0.091250256 |
| 100131434 | NR_027455 | LINC00893 | long intergenic non-protein coding RNA 893 | 5.014740 | 2.282768 | 0.008228124 | 0.093773409 |
| 26157 | NM_015660 | GIMAP2 | GTPase, IMAP family member 2 | 5.019478 | -0.574100 | 0.014279166 | 0.137724153 |
| 1576 | NM_001202855CYP3A4 | | cytochrome P450 family 3 subfamily A member 4 | 5.023270 | -2.418000 | 0.031346675 | 0.23265374 |
| 4026 | NM_001167671LPP | | LIM domain containing preferred translocation partner in li | 5.029516 | 6.665930 | 0.007635186 | 0.088055725 |
| 100130717 | NR_024482,NR HDHD5-AS1 | | HDHD5 antisense RNA 1 | 5.032195 | -2.419056 | 0.027591054 | 0.211791744 |
| 441307 | NR_033963 HRAT92 | | heart tissue-associated transcript 92 | 5.040537 | 0.862747 | 0.008729792 | 0.095999862 |
| 4983 | NM_002547 OPHN1 | | oligophrenin 1 | 5.055414 | 4.427269 | 0.007578288 | 0.087524778 |
| 101928177 | NR_135113 LOC101928177 | | uncharacterized LOC101928177 | 5.059209 | -1.682041 | 0.016666963 | 0.14924508 |
| 203274 | NR_146625,NR LINC00537 | | long intergenic non-protein coding RNA 537 | 5.061628 | -1.283591 | 0.015985273 | 0.146302603 |
| 100500865 | NR_037500 MIR3936 | | microRNA 3936 | 5.065618 | -1.130470 | 0.014889395 | 0.142191661 |
| 399715 | NR_040079 LOC399715 | | uncharacterized LOC399715 | 5.068774 | -1.011202 | 0.014048388 | 0.136491737 |
| 100616668 | NR_038258,NR TPTE2P5 | | transmembrane phosphoinositide 3-phosphatase and tensi | 5.068774 | -0.873413 | 0.014048388 | 0.136491737 |
| 3955 | NM_001040167LFNG | | LFNG O-fucosylpeptide 3-beta-N-acetylglucosaminyltransfe | 5.071374 | 4.119175 | 0.007497597 | 0.086883916 |
| 7494 | NM_001079539XBP1 | | X-box binding protein 1 | 5.086808 | 7.148429 | 0.007251501 | 0.084553152 |
| 392636 | NM_001004320AGMO | | alkylglycerol monooxygenase | 5.087801 | 2.302455 | 0.007730101 | 0.088980039 |
| 55512 | NM_018667 SMPD3 | | sphingomyelin phosphodiesterase 3 | 5.088630 | 0.587233 | 0.008552443 | 0.094530216 |
| 84073 | NM_032133 MYCBPAP | | MYCBP associated protein | 5.104792 | 2.065088 | 0.00763703 | 0.088055725 |
| 57715 | NM_001203244SEMA4G | | semaphorin 4G | 5.107811 | 5.749399 | 0.007138755 | 0.083450228 |
| 10083 | NM_001297764USH1C | | USH1 protein network component harmonin | 5.110372 | 4.719680 | 0.007152682 | 0.083542192 |
| 3718 | NM_000215 JAK3 | | Janus kinase 3 | 5.124181 | 2.614664 | 0.007456865 | 0.086460338 |
| 10410 | NM_021034,NRIFITM3 | | interferon induced transmembrane protein 3 | 5.125755 | 4.218474 | 0.007117091 | 0.08326755 |
| 3174 | NM_001330561HNF4G | | hepatocyte nuclear factor 4 gamma | 5.126962 | 3.289517 | 0.007300996 | 0.084986551 |
| 9796 | NM_001099335PHYHIP | | phytanoyl-CoA 2-hydroxylase interacting protein | 5.131076 | 0.680368 | 0.009759049 | 0.104161991 |
| 283375 | NM_001135195SLC39A5 | | solute carrier family 39 member 5 | 5.134065 | -1.792668 | 0.016434585 | 0.14789495 |
| 346653 | NM_001012454FAM71F2 | | family with sequence similarity 71 member F2 | 5.136691 | 0.514389 | 0.008311865 | 0.094415666 |
| 54757 | NM_001243746FAM20A | | FAM20A, golgi associated secretory pathway pseudokinase | 5.137221 | -1.353149 | 0.017256092 | 0.152800626 |
| 79015 | NR_034104 LINC01260 | | long intergenic non-protein coding RNA 1260 | 5.137221 | -1.605980 | 0.017256092 | 0.152800626 |
| 340267 | NM_001037763COL28A1 | | collagen type XXVIII alpha 1 chain | 5.137221 | -1.086235 | 0.017256092 | 0.152800626 |
| 375719 | NR_002817 AQP7P1 | | aquaporin 7 pseudogene 1 | 5.137221 | -1.680549 | 0.017256092 | 0.152800626 |
| 1364 | NM_001305 CLDN4 | | claudin 4 | 5.138237 | 3.938752 | 0.007092963 | 0.083126315 |
| 101927415 | NR_110049 LOC101927415 | | uncharacterized LOC101927415 | 5.154032 | -0.377390 | 0.011442864 | 0.118301114 |
| 574036 | NR_024337 SERTAD4-AS1 | | SERTAD4 antisense RNA 1 | 5.156106 | 0.202982 | 0.009609082 | 0.102879847 |
| 81607 | NM_030916 NECTIN4 | | nectin cell adhesion molecule 4 | 5.158149 | 1.761441 | 0.007720088 | 0.088914266 |
| 1815 | NM_000797 DRD4 | | dopamine receptor D4 | 5.166286 | -2.296060 | 0.028229227 | 0.215310308 |
| 100506606 | NR_103860 LOC100506606 | | uncharacterized LOC100506606 | 5.166286 | -2.443835 | 0.028229227 | 0.215310308 |
| 201229 | NM_001076680LYRM9 | | LYR motif containing 9 | 5.166670 | 1.752498 | 0.007568958 | 0.087524778 |
| 3382 | NM_001136020ICA1 | | islet cell autoantigen 1 | 5.168227 | 3.604831 | 0.006968006 | 0.081800908 |
| 346007 | NM_001142800EYS | | eyes shut homolog (Drosophila) | 5.179735 | -1.089969 | 0.013189951 | 0.129889357 |
| 3707 | NM_002221 ITPKB | | inositol-trisphosphate 3-kinase B | 5.195690 | 3.719751 | 0.006793379 | 0.080188171 |
| 80034 | NM_001172173CSRNP3 | | cysteine and serine rich nuclear protein 3 | 5.201190 | 1.117973 | 0.007608629 | 0.087826165 |
| 130367 | NM_001320833SGPP2 | | sphingosine-1-phosphate phosphatase 2 | 5.202556 | 5.180824 | 0.006650564 | 0.078716138 |

| 5950 | NM_001323517RBP4 | retinol binding protein 4 | 5.206938 | 2.920204 | 0.006799556 | 0.080188171 |
| --- | --- | --- | --- | --- | --- | --- |
| 130132 | NM_144629 RFTN2 | raftlin family member 2 | 5.211708 | -1.681450 | 0.015470278 | 0.145853555 |
| 283888 | NR_037158 IL21R-AS1 | IL21R antisense RNA 1 | 5.211708 | -1.636234 | 0.015470278 | 0.145853555 |
| 285386 | NM_198485 TPRG1 | tumor protein p63 regulated 1 | 5.211708 | -1.117730 | 0.015470278 | 0.145853555 |
| 100133172 | NR_026789 FAM66A | family with sequence similarity 66 member A | 5.211708 | -1.403635 | 0.015470278 | 0.145853555 |
| 9351 | NM_001130012SLC9A3R2 | SLC9A3 regulator 2 | 5.215994 | 6.530239 | 0.006479126 | 0.077112035 |
| 115111 | NM_001282356SLC26A7 | solute carrier family 26 member 7 | 5.217537 | -2.576280 | 0.049219036 | 0.303094897 |
| 8522 | NM_001130831GAS7 | growth arrest specific 7 | 5.222545 | 0.179555 | 0.009458691 | 0.101774719 |
| 80035 | NR_026808 ANP32A-IT1 | ANP32A intronic transcript 1 | 5.239538 | 0.516478 | 0.007737837 | 0.089019553 |
| 728621 | NM_001080850CCDC30 | coiled-coil domain containing 30 | 5.242906 | -0.226209 | 0.009718016 | 0.103777575 |
| 122945 | NM_001113475NOXRED1 | NADP dependent oxidoreductase domain containing 1 | 5.244145 | -1.120284 | 0.012907207 | 0.127744299 |
| 641649 | NM_001042595TMEM91 | transmembrane protein 91 | 5.257550 | 2.305038 | 0.00667733 | 0.078971163 |
| 54760 | NM_017573 PCSK4 | proprotein convertase subtilisin/kexin type 4 | 5.259810 | 1.659895 | 0.00699229 | 0.082039435 |
| 146713 | NM_001082575RBFOX3 | RNA binding protein, fox-1 homolog 3 | 5.260551 | 4.094293 | 0.006360783 | 0.076320644 |
| 728963 | NR_026768 RPS15AP10 | ribosomal protein S15a pseudogene 10 | 5.262513 | -2.101280 | 0.020964854 | 0.177437809 |
| 55592 | NR_024261,NR GOLGA2P5 | golgin A2 pseudogene 5 | 5.263059 | 3.220147 | 0.006471947 | 0.07707094 |
| 101752400 | NR_103792 CAPN10-AS1 | CAPN10 antisense RNA 1 (head to head) | 5.267928 | 0.754594 | 0.007437009 | 0.086278476 |
| 12 | NM_001085 SERPINA3 | serpin family A member 3 | 5.280058 | 2.639901 | 0.006565296 | 0.07791345 |
| 129804 | NM_001128165FBLN7 | fibulin 7 | 5.283028 | -1.724054 | 0.022901344 | 0.18519359 |
| 655 | NM_001719 BMP7 | bone morphogenetic protein 7 | 5.283998 | 2.099081 | 0.006544547 | 0.0777118 |
| 2159 | NM_000504,N F10 | coagulation factor X | 5.285588 | -0.867757 | 0.0106124 | 0.111317191 |
| 100132234 | NR_135254 LINC00543 | long intergenic non-protein coding RNA 543 | 5.285588 | -0.493158 | 0.0106124 | 0.111317191 |
| 56171 | NM_018897 DNAH7 | dynein axonemal heavy chain 7 | 5.286702 | 2.259644 | 0.00653126 | 0.077671618 |
| 101927391 | NR_110084 LOC101927391 | uncharacterized LOC101927391 | 5.296825 | 0.123892 | 0.008288537 | 0.094254194 |
| 26112 | NM_015621 CCDC69 | coiled-coil domain containing 69 | 5.299779 | 3.955292 | 0.006209858 | 0.076320644 |
| 101927043 | NR_110207,NR LOC101927043 | uncharacterized LOC101927043 | 5.305313 | -1.365593 | 0.015166636 | 0.143737334 |
| 25956 | NM_015490 SEC31B | SEC31 homolog B, COPII coat complex component | 5.307831 | 4.133306 | 0.00607245 | 0.074949347 |
| 55065 | NM_001104577SLC52A1 | solute carrier family 52 member 1 | 5.308274 | 0.944684 | 0.007276549 | 0.084797412 |
| 641455 | NM_001145442POTEM | POTE ankyrin domain family member M | 5.312540 | -0.546694 | 0.011009656 | 0.114843537 |
| 100506385 | NR_038885,NR LINC01426 | long intergenic non-protein coding RNA 1426 | 5.316543 | -0.925573 | 0.012602189 | 0.125371035 |
| 63914 | NR_026784 LINC01590 | long intergenic non-protein coding RNA 1590 | 5.318596 | -0.620335 | 0.010162502 | 0.107524988 |
| 29944 | NM_001282535PNMA3 | paraneoplastic Ma antigen 3 | 5.324144 | -1.453752 | 0.015875293 | 0.146302603 |
| 80072 | NR_027262 HEXA-AS1 | HEXA antisense RNA 1 | 5.335204 | -0.685035 | 0.011343315 | 0.117612265 |
| 50636 | NM_001001666ANO7 | anoctamin 7 | 5.337770 | 1.973043 | 0.006251728 | 0.076320644 |
| 105682749 | NR_131782 CCEPR | cervical carcinoma expressed PCNA regulatory lncRNA | 5.344154 | -2.295179 | 0.028984011 | 0.219448851 |
| 404037 | NM_023002 HAPLN4 | hyaluronan and proteoglycan link protein 4 | 5.347796 | -1.294949 | 0.01330018 | 0.130815016 |
| 168544 | NM_001329856ZNF467 | zinc finger protein 467 | 5.356265 | 2.855754 | 0.005987022 | 0.074156621 |
| 145957 | NM_138573 NRG4 | neuregulin 4 | 5.369571 | 2.055478 | 0.006317416 | 0.076320644 |
| 136227 | NM_001278563COL26A1 | collagen type XXVI alpha 1 chain | 5.371827 | 0.242443 | 0.007865849 | 0.090301368 |
| 574406 | NR_104133 ADAMTSL4-AS1 | ADAMTSL4 antisense RNA 1 | 5.374187 | -1.822940 | 0.019298276 | 0.166119364 |
| 653677 | NR_004401 SEC1P | secretory blood group 1, pseudogene | 5.374187 | -2.122012 | 0.019298276 | 0.166119364 |
| 146880 | NR_027487 ARHGAP27P1 | Rho GTPase activating protein 27 pseudogene 1 | 5.378284 | 3.089733 | 0.005876787 | 0.073135502 |
| 8153 | NM_005440 RND2 | Rho family GTPase 2 | 5.389359 | -0.801532 | 0.014231827 | 0.137691926 |

| 7007 | NM_005422 | TECTA | tectorin alpha | 5.404638 | -0.838661 | 0.011040586 | 0.115050132 |
| --- | --- | --- | --- | --- | --- | --- | --- |
| 102723566 | NR_136302 | LOC102723566 | uncharacterized LOC102723566 | 5.409279 | 0.874549 | 0.006439795 | 0.076909441 |
| 64122 | NM_022158 | FN3K | fructosamine 3 kinase | 5.410496 | 3.510423 | 0.005649054 | 0.070818598 |
| 27324 | NM_001080430TOX3 | | TOX high mobility group box family member 3 | 5.411748 | 3.316305 | 0.005704451 | 0.071203116 |
| 149134 | NR_015422 LINC01341 | | long intergenic non-protein coding RNA 1341 | 5.415744 | -1.710990 | 0.014824518 | 0.141965347 |
| 2523 | NM_000148,N FUT1 | | fucosyltransferase 1 (H blood group) | 5.427419 | 0.864863 | 0.006448666 | 0.076926562 |
| 56109 | NM_018919,N PCDHGA6 | | protocadherin gamma subfamily A, 6 | 5.427963 | 0.067255 | 0.008792968 | 0.096540624 |
| 221756 | NR_033851 SERPINB9P1 | | serpin family B member 9 pseudogene 1 | 5.442941 | -1.009900 | 0.011425695 | 0.118301114 |
| 503569 | NR_033932 RGMB-AS1 | | RGMB antisense RNA 1 | 5.447340 | -1.648992 | 0.016730606 | 0.149620658 |
| 8330 | NM_003510 HIST1H2AK | | histone cluster 1 H2A family member k | 5.452318 | -2.250224 | 0.023000594 | 0.185556665 |
| 4600 | NM_002463 MX2 | | MX dynamin like GTPase 2 | 5.464535 | -2.180665 | 0.042028125 | 0.279264941 |
| 84740 | NR_026892 AFAP1-AS1 | | AFAP1 antisense RNA 1 | 5.464535 | -2.269947 | 0.042028125 | 0.279264941 |
| 100616359 | NR_039883 MIR4730 | | microRNA 4730 | 5.464535 | -3.003128 | 0.042028125 | 0.279264941 |
| 6452 | NM_001122681SH3BP2 | | SH3 domain binding protein 2 | 5.467478 | 5.177351 | 0.005260068 | 0.066997853 |
| 280 | NM_020978 AMY2B | | amylase, alpha 2B (pancreatic) | 5.468228 | 1.393644 | 0.005971018 | 0.074046048 |
| 107984921 | NR_147210,NR LINC01772 | | long intergenic non-protein coding RNA 1772 | 5.492090 | -1.430228 | 0.011914274 | 0.120276597 |
| 101928978 | NR_125909 LOC101928978 | | uncharacterized LOC101928978 | 5.496803 | -0.548378 | 0.00896833 | 0.09786702 |
| 101929736 | NR_108104 LINC01372 | | long intergenic non-protein coding RNA 1372 | 5.496803 | -0.742806 | 0.00896833 | 0.09786702 |
| 100421577 | NR_038275 FAM21EP | | family with sequence similarity 21 member E, pseudogene | 5.507344 | -1.695548 | 0.013852865 | 0.135001974 |
| 726 | NM_004055 CAPN5 | | calpain 5 | 5.509281 | 5.406938 | 0.005062632 | 0.064882622 |
| 153657 | NM_001317949TTC23L | | tetratricopeptide repeat domain 23 like | 5.510515 | -2.417355 | 0.025843036 | 0.203178717 |
| 642273 | NM_001077710FAM110C | | family with sequence similarity 110 member C | 5.512029 | 2.759281 | 0.005362134 | 0.068004748 |
| 201456 | NM_001142958FBXO15 | | F-box protein 15 | 5.513451 | 0.370287 | 0.007046927 | 0.082633614 |
| 1612 | NM_001288729DAPK1 | | death associated protein kinase 1 | 5.516162 | 6.897860 | 0.005013262 | 0.064431951 |
| 339977 | NM_001024611LRRC66 | | leucine rich repeat containing 66 | 5.516296 | -0.245177 | 0.007511748 | 0.086999166 |
| 55036 | NM_001243342CCDC40 | | coiled-coil domain containing 40 | 5.521673 | 3.908676 | 0.00511491 | 0.065390547 |
| 100506874 | NR_038273,NR LINC00933 | | long intergenic non-protein coding RNA 933 | 5.522281 | -1.416702 | 0.012211177 | 0.122556139 |
| 53841 | NM_001171968CDHR5 | | cadherin related family member 5 | 5.525863 | -0.937314 | 0.009527172 | 0.102214498 |
| 100287497 | NR_136590,NR LOC100287497 | | septin 7 pseudogene | 5.528434 | 2.159247 | 0.005312104 | 0.067577415 |
| 56142 | NM_018909,N PCDHA6 | | protocadherin alpha 6 | 5.533268 | -0.537082 | 0.011072497 | 0.115266534 |
| 146456 | NM_144676 TMED6 | | transmembrane p24 trafficking protein 6 | 5.533862 | -2.297467 | 0.019102998 | 0.164712594 |
| 64081 | NM_001033083PBLD | | phenazine biosynthesis like protein domain containing | 5.540570 | 3.227530 | 0.005060236 | 0.064882622 |
| 6326 | NM_001040142SCN2A | | sodium voltage-gated channel alpha subunit 2 | 5.547986 | -1.634191 | 0.014436237 | 0.139019348 |
| 55911 | NM_018690 APOBR | | apolipoprotein B receptor | 5.547986 | -1.807205 | 0.014436237 | 0.139019348 |
| 400579 | NR_104343 LINC02076 | | long intergenic non-protein coding RNA 2076 | 5.548587 | 0.225848 | 0.006815591 | 0.080285027 |
| 10154 | NM_005761,NRPLXNC1 | | plexin C1 | 5.554850 | 2.246072 | 0.005167975 | 0.066028145 |
| 2974 | NR_003923 GUCY1B2 | | guanylate cyclase 1 soluble subunit beta 2 (pseudogene) | 5.556185 | 1.776939 | 0.005410257 | 0.068358604 |
| 3664 | NM_001206696IRF6 | | interferon regulatory factor 6 | 5.558212 | -0.750384 | 0.008706248 | 0.09579188 |
| 10878 | NM_001166624CFHR3 | | complement factor H related 3 | 5.560311 | 0.627446 | 0.005883208 | 0.073135502 |
| 1767 | NM_001369 DNAH5 | | dynein axonemal heavy chain 5 | 5.565396 | 3.781179 | 0.004929302 | 0.063567711 |
| 23704 | NM_080671 KCNE4 | | potassium voltage-gated channel subfamily E regulatory su | 5.570642 | 2.312563 | 0.005091958 | 0.065137391 |
| 51087 | NM_015982 YBX2 | | Y-box binding protein 2 | 5.584714 | -0.306071 | 0.007866718 | 0.090301368 |
| 341019 | NM_001350255DCDC1 | | doublecortin domain containing 1 | 5.586451 | 2.465267 | 0.005021123 | 0.064470477 |

| 1735 | NM_001362 | DIO3 | iodothyronine deiodinase 3 | 5.591417 | -2.696187 | 0.029884417 | 0.224295781 |
| --- | --- | --- | --- | --- | --- | --- | --- |
| 9389 | NM_001320033SLC22A14 | | solute carrier family 22 member 14 | 5.591417 | -2.825231 | 0.029884417 | 0.224295781 |
| 26045 | NM_015564 LRRTM2 | | leucine rich repeat transmembrane neuronal 2 | 5.591417 | -2.791879 | 0.029884417 | 0.224295781 |
| 286464 | NM_001304548CFAP47 | | cilia and flagella associated protein 47 | 5.591417 | -2.966872 | 0.029884417 | 0.224295781 |
| 646576 | NR_037595 HHIP-AS1 | | HHIP antisense RNA 1 | 5.592206 | 0.230493 | 0.006651961 | 0.078716138 |
| 430 | NM_005170 ASCL2 | | achaete-scute family bHLH transcription factor 2 | 5.597535 | -2.229946 | 0.016400808 | 0.147811798 |
| 105372483 | NR_136527 LOC105372483 | | uncharacterized LOC105372483 | 5.601634 | -0.874125 | 0.009220227 | 0.099644925 |
| 5582 | NM_001316329PRKCG | | protein kinase C gamma | 5.607200 | 2.793534 | 0.004933693 | 0.063584702 |
| 10161 | NM_001162497LPAR6 | | lysophosphatidic acid receptor 6 | 5.611708 | 2.702799 | 0.004927964 | 0.063567711 |
| 375449 | NM_001164664MAST4 | | microtubule associated serine/threonine kinase family mem | 5.628779 | 4.592655 | 0.004627548 | 0.061202582 |
| 100506827 | NR_131958,NR LINC02475 | | long intergenic non-protein coding RNA 2475 | 5.659278 | -1.945621 | 0.017478245 | 0.154065215 |
| 51710 | NM_001164276ZNF44 | | zinc finger protein 44 | 5.663512 | 1.096746 | 0.005395489 | 0.068302136 |
| 7059 | NM_001252607THBS3 | | thrombospondin 3 | 5.665363 | 4.837703 | 0.004461253 | 0.059767499 |
| 3959 | NM_005567 LGALS3BP | | galectin 3 binding protein | 5.670857 | 8.174722 | 0.004402801 | 0.059285385 |
| 66004 | NM_023946,N LYNX1 | | Ly6/neurotoxin 1 | 5.675231 | 3.302441 | 0.004577175 | 0.060730504 |
| 147798 | NM_001145303TMC4 | | transmembrane channel like 4 | 5.682553 | 1.645593 | 0.004887916 | 0.063112704 |
| 678655 | NR_015382 CD27-AS1 | | CD27 antisense RNA 1 | 5.697339 | 1.845383 | 0.004873482 | 0.062972567 |
| 284186 | NM_178520 TMEM105 | | transmembrane protein 105 | 5.699385 | 1.129368 | 0.00507764 | 0.064997195 |
| 11093 | NM_139025,N ADAMTS13 | | ADAM metallopeptidase with thrombospondin type 1 motif | 5.707382 | 2.288372 | 0.004580278 | 0.060732723 |
| 100861412 | NM_001256141FSBP | | fibrinogen silencer binding protein | 5.708105 | 2.956181 | 0.004455292 | 0.059726323 |
| 149837 | NR_015406 LINC00654 | | long intergenic non-protein coding RNA 654 | 5.708530 | -1.379903 | 0.010135147 | 0.107400363 |
| 100505678 | NR_040093 STARD4-AS1 | | STARD4 antisense RNA 1 | 5.709209 | -1.589711 | 0.015141462 | 0.143737334 |
| 563 | NM_001185 AZGP1 | | alpha-2-glycoprotein 1, zinc-binding | 5.711538 | 0.223635 | 0.005277167 | 0.067174281 |
| 619207 | NR_002934 SCART1 | | scavenger receptor family member expressed on T-cells 1 | 5.713354 | 0.754807 | 0.005048498 | 0.064781746 |
| 26751 | NM_001159597SH3YL1 | | SH3 and SYLF domain containing 1 | 5.719888 | 3.533011 | 0.004365777 | 0.058960997 |
| 100289137 | NR_047651 FAM95C | | family with sequence similarity 95 member C | 5.728774 | 4.657399 | 0.004240879 | 0.057598545 |
| 100128071 | NM_001167676FAM229A | | family with sequence similarity 229 member A | 5.729469 | 0.703948 | 0.005077857 | 0.064997195 |
| 128434 | NM_080607 VSTM2L | | V-set and transmembrane domain containing 2 like | 5.730702 | 2.592611 | 0.004474234 | 0.05982482 |
| 84911 | NM_001256838ZNF382 | | zinc finger protein 382 | 5.738351 | -1.838448 | 0.018852638 | 0.162961477 |
| 400891 | NM_001291006LRRC74B | | leucine rich repeat containing 74B | 5.738351 | -2.524339 | 0.018852638 | 0.162961477 |
| 101928058 | NR_136274,NR LOC101928058 | | uncharacterized LOC101928058 | 5.741538 | 0.316163 | 0.00569582 | 0.071163158 |
| 203429 | NR_026595 FAM226A | | family with sequence similarity 226 member A (non-protein | 5.742884 | -0.155115 | 0.006681283 | 0.078972764 |
| 160335 | NM_001320321TMTC2 | | transmembrane and tetratricopeptide repeat containing 2 | 5.747518 | 5.169723 | 0.004168391 | 0.056837955 |
| 80319 | NM_025212,NRCXXC4 | | CXXC finger protein 4 | 5.755571 | 2.307495 | 0.004403178 | 0.059285385 |
| 54682 | NM_018050 MANSC1 | | MANSC domain containing 1 | 5.759409 | 5.199781 | 0.004126615 | 0.056342598 |
| 100505865 | NR_046242 LINC00920 | | long intergenic non-protein coding RNA 920 | 5.761987 | 1.307052 | 0.00485189 | 0.062843668 |
| 2353 | NM_005252 FOS | | Fos proto-oncogene, AP-1 transcription factor subunit | 5.762225 | 7.594211 | 0.00408822 | 0.055966129 |
| 196403 | NM_001286245DTX3 | | deltex E3 ubiquitin ligase 3 | 5.762656 | 4.866016 | 0.004123612 | 0.056338776 |
| 400673 | NM_001017921VMAC | | vimentin-type intermediate filament associated coiled-coil p | 5.771945 | 2.188657 | 0.00434465 | 0.058776375 |
| 65266 | NM_001321299WNK4 | | WNK lysine deficient protein kinase 4 | 5.782011 | 4.002999 | 0.004111203 | 0.056206371 |
| 693122 | NR_030398 MIR421 | | microRNA 421 | 5.784695 | -2.206907 | 0.01601018 | 0.146302603 |
| 100506125 | NR_110019,NR LOC100506125 | | uncharacterized LOC100506125 | 5.784695 | -1.839411 | 0.01601018 | 0.146302603 |
| 137835 | NM_001145153TMEM71 | | transmembrane protein 71 | 5.790231 | -2.342008 | 0.0262636 | 0.205382014 |

| 283673 | NR_026949 | EWSAT1 | Ewing sarcoma associated transcript 1 | 5.790231 | -2.577826 | 0.0262636 | 0.205382014 |
| --- | --- | --- | --- | --- | --- | --- | --- |
| 100996266 | NR_120506 | LINC01510 | long intergenic non-protein coding RNA 1510 | 5.790231 | -2.759428 | 0.0262636 | 0.205382014 |
| 9924 | NM_001127460PAN2 | | PAN2 poly(A) specific ribonuclease subunit | 5.795338 | 6.336382 | 0.003986724 | 0.054976921 |
| 22996 | NM_001080494TTC39A | | tetratricopeptide repeat domain 39A | 5.810352 | 3.516860 | 0.004102466 | 0.056124016 |
| 358 | NM_001329872AQP1 | | aquaporin 1 (Colton blood group) | 5.823314 | 0.669782 | 0.004772058 | 0.062415821 |
| 339400 | NR_103778,NR FLG-AS1 | | FLG antisense RNA 1 | 5.849238 | 1.184218 | 0.004694862 | 0.061934456 |
| 57156 | NM_020431 TMEM63C | | transmembrane protein 63C | 5.859188 | 1.680346 | 0.004307515 | 0.058426851 |
| 283229 | NM_001286606CRACR2B | | calcium release activated channel regulator 2B | 5.861251 | 2.075758 | 0.004208901 | 0.057239394 |
| 23179 | NM_001297669RGL1 | | ral guanine nucleotide dissociation stimulator like 1 | 5.866026 | 3.062345 | 0.003928533 | 0.054464951 |
| 29121 | NM_001004419CLEC2D | | C-type lectin domain family 2 member D | 5.869533 | 2.041027 | 0.004174002 | 0.056876967 |
| 140733 | NM_001033087MACROD2 | | MACRO domain containing 2 | 5.882388 | 0.946988 | 0.004453791 | 0.059726323 |
| 56521 | NM_021800,N DNAJC12 | | DnaJ heat shock protein family (Hsp40) member C12 | 5.883027 | 5.220889 | 0.003734132 | 0.052295548 |
| 56261 | NM_019593 GPCPD1 | | glycerophosphocholine phosphodiesterase 1 | 5.884764 | 7.288066 | 0.003703647 | 0.051938944 |
| 100505592 | NR_110428,NR GAPLINC | | gastric adenocarcinoma associated, positive CD44 regulator | 5.895908 | 0.786316 | 0.004546843 | 0.060522162 |
| 389524 | NM_001003795GTF2IRD2B | | GTF2I repeat domain containing 2B | 5.900207 | 1.968307 | 0.004075693 | 0.055868589 |
| 22917 | NM_207341 ZP1 | | zona pellucida glycoprotein 1 | 5.908494 | -2.789504 | 0.046195495 | 0.296479617 |
| 57863 | NM_001127173CADM3 | | cell adhesion molecule 3 | 5.908494 | -3.251117 | 0.046195495 | 0.296479617 |
| 125931 | NM_001102597CEACAM20 | | carcinoembryonic antigen related cell adhesion molecule 20 | 5.908494 | -3.497315 | 0.046195495 | 0.296479617 |
| 285051 | NM_001163561STPG4 | | sperm-tail PG-rich repeat containing 4 | 5.908494 | -2.756923 | 0.046195495 | 0.296479617 |
| 102724708 | NR_136223,NR LOC102724708 | | uncharacterized LOC102724708 | 5.908494 | -3.002032 | 0.046195495 | 0.296479617 |
| 101929372 | NM_001288707LOC101929372 | | uncharacterized LOC101929372 | 5.910112 | -2.121555 | 0.01468201 | 0.140796188 |
| 56164 | NM_001260504STK31 | | serine/threonine kinase 31 | 5.910993 | 1.218328 | 0.004417194 | 0.059400172 |
| 56131 | NM_018938 PCDHB4 | | protocadherin beta 4 | 5.921351 | -0.814441 | 0.007249959 | 0.084553152 |
| 23025 | NM_001080421UNC13A | | unc-13 homolog A | 5.924107 | 5.271230 | 0.003606899 | 0.051642736 |
| 60598 | NM_022358 KCNK15 | | potassium two pore domain channel subfamily K member 1 | 5.924382 | -0.177269 | 0.005750748 | 0.071659166 |
| 92270 | NM_001017971ATP6AP1L | | ATPase H+ transporting accessory protein 1 like | 5.930152 | 0.655732 | 0.004438005 | 0.059571789 |
| 604 | NM_001130845BCL6 | | B-cell CLL/lymphoma 6 | 5.931648 | 5.130874 | 0.003590157 | 0.051571109 |
| 219621 | NM_173554 C10orf107 | | chromosome 10 open reading frame 107 | 5.934214 | -0.282207 | 0.006195184 | 0.076320644 |
| 100506076 | NR_103732,NR LOC100506076 | | uncharacterized LOC100506076 | 5.937531 | -1.172751 | 0.008207657 | 0.09359172 |
| 2243 | NM_000508,N FGA | | fibrinogen alpha chain | 5.949019 | 6.567922 | 0.003522162 | 0.050788504 |
| 10739 | NM_001098527RFPL2 | | ret finger protein like 2 | 5.952831 | -0.266186 | 0.005696592 | 0.071163158 |
| 1262 | NM_001037329CNGA4 | | cyclic nucleotide gated channel alpha 4 | 5.958531 | -2.493806 | 0.030962986 | 0.230550528 |
| 8842 | NM_001145847PROM1 | | prominin 1 | 5.958531 | -2.965787 | 0.030962986 | 0.230550528 |
| 102465449 | NR_106809 MIR6751 | | microRNA 6751 | 5.958531 | -2.965787 | 0.030962986 | 0.230550528 |
| 102724297 | NR_125406 LOC102724297 | | uncharacterized LOC102724297 | 5.958531 | -2.929071 | 0.030962986 | 0.230550528 |
| 107546764 | NR_136406 LINC01976 | | long intergenic non-protein coding RNA 1976 | 5.958531 | -2.824188 | 0.030962986 | 0.230550528 |
| 1846 | NM_001394,N DUSP4 | | dual specificity phosphatase 4 | 5.963064 | 8.779221 | 0.003476346 | 0.050215238 |
| 442117 | NM_001034845GALNTL6 | | polypeptide N-acetylgalactosaminyltransferase-like 6 | 5.973223 | -0.578374 | 0.006016724 | 0.074479908 |
| 127845 | NM_198447 GOLT1A | | golgi transport 1A | 5.974418 | 2.065711 | 0.003855167 | 0.053699744 |
| 257407 | NM_001144994C2orf72 | | chromosome 2 open reading frame 72 | 5.977605 | 4.970929 | 0.003463715 | 0.05010276 |
| 2049 | NM_004443 EPHB3 | | EPH receptor B3 | 5.988703 | 1.582561 | 0.003850935 | 0.053699744 |
| 100505625 | NR_103771 LINC02102 | | long intergenic non-protein coding RNA 2102 | 5.989046 | -2.894642 | 0.023136836 | 0.18636505 |
| 100507398 | NR_103812 INTS6-AS1 | | INTS6 antisense RNA 1 | 5.995991 | 0.119730 | 0.005388454 | 0.068254845 |

| 105370941 | NR_135665 | LOC105370941 | uncharacterized LOC105370941 | 5.998907 | -0.395940 | 0.00622935 | 0.076320644 |
| --- | --- | --- | --- | --- | --- | --- | --- |
| 26249 | NM_001257194KLHL3 | | kelch like family member 3 | 6.013794 | 1.688912 | 0.00381766 | 0.053320927 |
| 2591 | NM_004482 GALNT3 | | polypeptide N-acetylgalactosaminyltransferase 3 | 6.022543 | 0.724593 | 0.004059928 | 0.055757652 |
| 162962 | NM_001102657ZNF836 | | zinc finger protein 836 | 6.023121 | -0.484455 | 0.006928561 | 0.08138404 |
| 123096 | NM_001039355SLC25A29 | | solute carrier family 25 member 29 | 6.024171 | 5.249048 | 0.00333808 | 0.048625487 |
| 100129424 | NR_026660 RPL19P12 | | ribosomal protein L19 pseudogene 12 | 6.024383 | -2.393702 | 0.015540812 | 0.146302603 |
| 101060171 | NR_138082 ARMC4P1 | | armadillo repeat containing 4 pseudogene 1 | 6.024383 | -2.418959 | 0.015540812 | 0.146302603 |
| 54567 | NM_019074 DLL4 | | delta like canonical Notch ligand 4 | 6.025871 | -0.182840 | 0.005329484 | 0.067756842 |
| 27124 | NM_001002837INPP5J | | inositol polyphosphate-5-phosphatase J | 6.026192 | 1.518156 | 0.003794614 | 0.053070717 |
| 104413891 | NR_126423 SAPCD1-AS1 | | SAPCD1 antisense RNA 1 | 6.042976 | -0.635156 | 0.006029803 | 0.074552582 |
| 23767 | NM_013281,N FLRT3 | | fibronectin leucine rich transmembrane protein 3 | 6.043509 | 4.658238 | 0.003315793 | 0.048403084 |
| 9034 | NM_001130910CCRL2 | | C-C motif chemokine receptor like 2 | 6.044564 | 0.371595 | 0.004874019 | 0.062972567 |
| 6844 | NM_001330125VAMP2 | | vesicle associated membrane protein 2 | 6.051900 | 5.202626 | 0.003268702 | 0.047816908 |
| 1520 | NM_001199739CTSS | | cathepsin S | 6.067973 | 5.895651 | 0.003222172 | 0.047269947 |
| 8398 | NM_001004426PLA2G6 | | phospholipase A2 group VI | 6.069225 | 3.161442 | 0.003349837 | 0.048762406 |
| 93653 | NR_002330 ST7-AS1 | | ST7 antisense RNA 1 | 6.069264 | -0.990208 | 0.007806323 | 0.089707667 |
| 386758 | NR_037159,NR ZNF582-AS1 | | ZNF582 antisense RNA 1 (head to head) | 6.077268 | -0.412162 | 0.006881669 | 0.080925138 |
| 375513 | NR_132999,NR GUSBP4 | | glucuronidase, beta pseudogene 4 | 6.081495 | 1.927974 | 0.003546029 | 0.051043566 |
| 79148 | NM_001032278MMP28 | | matrix metallopeptidase 28 | 6.100982 | 1.570952 | 0.003531503 | 0.050869867 |
| 162968 | NM_001207009ZNF497 | | zinc finger protein 497 | 6.106468 | 2.412459 | 0.003334229 | 0.048603612 |
| 100128890 | NR_027423 FAM66B | | family with sequence similarity 66 member B | 6.133524 | -0.413672 | 0.0060441 | 0.074684708 |
| 144448 | NM_001100917TSPAN19 | | tetraspanin 19 | 6.141411 | -1.249725 | 0.007418198 | 0.086205295 |
| 201305 | NM_001320449SPNS3 | | sphingolipid transporter 3 (putative) | 6.155906 | -1.561623 | 0.010295292 | 0.108707559 |
| 115265 | NM_145244 DDIT4L | | DNA damage inducible transcript 4 like | 6.166521 | 2.756216 | 0.003181293 | 0.046703372 |
| 10266 | NM_005854 RAMP2 | | receptor activity modifying protein 2 | 6.167399 | -1.855964 | 0.014138806 | 0.137027842 |
| 2199 | NM_001004019FBLN2 | | fibulin 2 | 6.173605 | 0.712131 | 0.004332741 | 0.058653633 |
| 57096 | NM_020366 RPGRIP1 | | retinitis pigmentosa GTPase regulator interacting protein 1 | 6.180887 | -0.327551 | 0.005697181 | 0.071163158 |
| 162514 | NM_001258205TRPV3 | | transient receptor potential cation channel subfamily V me | 6.187769 | 0.380341 | 0.004577081 | 0.060730504 |
| 64577 | NM_001193480ALDH8A1 | | aldehyde dehydrogenase 8 family member A1 | 6.187781 | -1.003189 | 0.005972451 | 0.074046048 |
| 648740 | NR_024438 ACTG1P4 | | actin gamma 1 pseudogene 4 | 6.187860 | -2.578114 | 0.020429716 | 0.173612331 |
| 100289019 | NR_033374 SLC25A25-AS1 | | SLC25A25 antisense RNA 1 | 6.188054 | 4.711720 | 0.002946639 | 0.04394465 |
| 83719 | NM_001145524YPEL3 | | yippee like 3 | 6.189712 | 2.923831 | 0.003114001 | 0.045943736 |
| 5739 | NM_000960 PTGIR | | prostaglandin I2 (prostacyclin) receptor (IP) | 6.205529 | -2.317135 | 0.026707832 | 0.207532496 |
| 10637 | NM_020997 LEFTY1 | | left-right determination factor 1 | 6.205529 | -3.122873 | 0.026707832 | 0.207532496 |
| 445582 | NM_001083538POTEE | | POTE ankyrin domain family member E | 6.205529 | -2.441192 | 0.026707832 | 0.207532496 |
| 100126317 | NR_030620 MIR374B | | microRNA 374b | 6.205529 | -3.042292 | 0.026707832 | 0.207532496 |
| 100874372 | NR_046816 ARHGAP26-IT1 | | ARHGAP26 intronic transcript 1 | 6.205529 | -3.082023 | 0.026707832 | 0.207532496 |
| 727 | NM_001317163C5 | | complement C5 | 6.232956 | 6.762048 | 0.002821926 | 0.0427381 |
| 2529 | NM_004479 FUT7 | | fucosyltransferase 7 | 6.234501 | -3.444802 | 0.03850259 | 0.26226593 |
| 26134 | NR_104269 RALGAPA1P1 | | Ral GTPase activating protein catalytic alpha subunit 1 pseu | 6.234501 | -3.163422 | 0.03850259 | 0.26226593 |
| 56106 | NM_018913,N PCDHGA10 | | protocadherin gamma subfamily A, 10 | 6.234501 | -2.927837 | 0.03850259 | 0.26226593 |
| 102466270 | NR_106799 MIR6741 | | microRNA 6741 | 6.234501 | -3.163422 | 0.03850259 | 0.26226593 |
| 109623452 | NR_145780 SNORD159 | | small nucleolar RNA, C/D box 159 | 6.234501 | -3.393952 | 0.03850259 | 0.26226593 |

| 80144 | NM_001166133FRAS1 Fraser extracellular matrix complex subunit 1 | 6.241813 | 6.370475 | 0.002806896 | 0.0427381 |
| --- | --- | --- | --- | --- | --- |
| 126075 | NM_001080503CCDC159 coiled-coil domain containing 159 | 6.250564 | 2.287323 | 0.002984532 | 0.044381772 |
| 284677 | NR_147122,NR SNHG28 small nucleolar RNA host gene 28 | 6.258987 | 0.703353 | 0.003468557 | 0.050137738 |
| 9048 | NM_001136215ARTN artemin | 6.267751 | -0.255179 | 0.004516623 | 0.060158621 |
| 3732 | NM_001024844CD82 CD82 molecule | 6.268813 | 3.431073 | 0.002869379 | 0.043071916 |
| 84171 | NM_032211 LOXL4 lysyl oxidase like 4 | 6.284913 | 2.176169 | 0.003047389 | 0.045154178 |
| 400242 | NR_015415 DICER1-AS1 DICER1 antisense RNA 1 | 6.290674 | 1.259778 | 0.00315006 | 0.046310581 |
| 8646 | NM_001304472CHRD chordin | 6.294353 | -0.901601 | 0.005535597 | 0.069734366 |
| 57161 | NM_021255 PELI2 pellino E3 ubiquitin protein ligase family member 2 | 6.311557 | 4.099070 | 0.002728905 | 0.042409763 |
| 125206 | NM_001042450SLC5A10 solute carrier family 5 member 10 | 6.313867 | -1.468763 | 0.007338043 | 0.085369756 |
| 10516 | NM_006329 FBLN5 fibulin 5 | 6.326485 | 2.426633 | 0.002926952 | 0.043682547 |
| 51351 | NM_015852 ZNF117 zinc finger protein 117 | 6.335850 | 1.432715 | 0.003067985 | 0.04539432 |
| 94240 | NM_001002264EPSTI1 epithelial stromal interaction 1 | 6.342321 | -1.925929 | 0.014967264 | 0.142803435 |
| 952 | NM_001775,NRCD38 CD38 molecule | 6.361140 | 4.631430 | 0.002605014 | 0.040867837 |
| 109729182 | NR_147193 DM1-AS DM1 locus antisense RNA | 6.376356 | -1.405012 | 0.006633377 | 0.078631171 |
| 10590 | NM_006998 SCGN secretagogin, EF-hand calcium binding protein | 6.386674 | -2.666234 | 0.018080005 | 0.157932812 |
| 100133669 | NR_026913 LOC100133669 uncharacterized LOC100133669 | 6.409171 | -1.039570 | 0.005883368 | 0.073135502 |
| 116828 | NR_026928 N4BP2L2-IT2 N4BPL2 intronic transcript 2 | 6.415231 | 0.957424 | 0.002958552 | 0.044077797 |
| 552891 | NM_004125 DNAJC25-GNG10 DNAJC25-GNG10 readthrough | 6.415737 | 0.750915 | 0.003805184 | 0.053182585 |
| 102724312 | NR_125994,NR LINC01770 long intergenic non-protein coding RNA 1770 | 6.423345 | -1.390829 | 0.007286762 | 0.084868617 |
| 2705 | NM_000166,N GJB1 gap junction protein beta 1 | 6.426146 | -0.744269 | 0.004482885 | 0.05982482 |
| 282763 | NM_001005567OR51B5 olfactory receptor family 51 subfamily B member 5 | 6.429998 | 0.198310 | 0.006072763 | 0.074949347 |
| 388743 | NM_001143962CAPN8 calpain 8 | 6.430877 | 1.821187 | 0.002712218 | 0.042182134 |
| 122622 | NM_001320424ADSSL1 adenylosuccinate synthase like 1 | 6.433701 | 4.144344 | 0.002488919 | 0.039541697 |
| 2634 | NM_004120 GBP2 guanylate binding protein 2 | 6.451878 | 3.426373 | 0.002548721 | 0.040250432 |
| 343521 | NM_001013632TCTEX1D4 Tctex1 domain containing 4 | 6.452527 | -2.791153 | 0.02310992 | 0.18622076 |
| 866 | NM_001756 SERPINA6 serpin family A member 6 | 6.468629 | 2.698677 | 0.002530136 | 0.040012133 |
| 1193 | NM_001289 CLIC2 chloride intracellular channel 2 | 6.470336 | -0.473142 | 0.004638009 | 0.061301741 |
| 1114 | NM_001819 CHGB chromogranin B | 6.471892 | 4.992112 | 0.002370615 | 0.038071568 |
| 83959 | NM_001174089SLC4A11 solute carrier family 4 member 11 | 6.476230 | 4.646118 | 0.002380393 | 0.038198938 |
| 149478 | NM_001136537BTBD19 BTB domain containing 19 | 6.478046 | 2.556007 | 0.002514869 | 0.039830432 |
| 8608 | NM_001320108RDH16 retinol dehydrogenase 16 (all-trans) | 6.480342 | -0.489131 | 0.004049102 | 0.055651614 |
| 284013 | NM_001144939VMO1 vitelline membrane outer layer 1 homolog | 6.501015 | 0.508208 | 0.003181127 | 0.046703372 |
| 10599 | NM_006446 SLCO1B1 solute carrier organic anion transporter family member 1B1 | 6.502489 | 2.115524 | 0.002568887 | 0.040531987 |
| 154822 | NR_024394 LINC00689 long intergenic non-protein coding RNA 689 | 6.508682 | -2.368108 | 0.01348744 | 0.132221653 |
| 100129845 | NR_038910 PCOLCE-AS1 PCOLCE antisense RNA 1 | 6.508682 | -2.099307 | 0.01348744 | 0.132221653 |
| 6819 | NM_001056,N SULT1C2 sulfotransferase family 1C member 2 | 6.511918 | 1.127862 | 0.002773315 | 0.0427381 |
| 338799 | NR_002809 LINC01089 long intergenic non-protein coding RNA 1089 | 6.524885 | 2.856775 | 0.002420025 | 0.038714778 |
| 8416 | NM_003568 ANXA9 annexin A9 | 6.528657 | 1.373242 | 0.002648461 | 0.041439804 |
| 440173 | NR_027471 LOC440173 uncharacterized LOC440173 | 6.540883 | 0.769354 | 0.003091499 | 0.045676893 |
| 79668 | NM_001178055PARP8 poly(ADP-ribose) polymerase family member 8 | 6.545132 | 2.431754 | 0.002492322 | 0.03956537 |
| 100505881 | NR_038343,NR MAGI2-AS3 MAGI2 antisense RNA 3 | 6.548565 | 1.538073 | 0.002619117 | 0.041042755 |
| 4135 | NM_033063,N MAP6 microtubule associated protein 6 | 6.551092 | 2.625105 | 0.002389 | 0.038307339 |

| 220004 | NM_001170753PPP1R32 protein phosphatase 1 regulatory subunit 32 | 6.556785 | 0.818835 | 0.002693465 | 0.041921991 |
| --- | --- | --- | --- | --- | --- |
| 139728 | NM_001039582PNCK pregnancy up-regulated nonubiquitous CaM kinase | 6.560508 | -3.163592 | 0.03224147 | 0.233618934 |
| 100616262 | NR_039850 MIR4701 microRNA 4701 | 6.560508 | -3.206863 | 0.03224147 | 0.233618934 |
| 101927703 | NR_125804 FOXCUT FOXC1 upstream transcript (non-protein coding) | 6.560508 | -2.466255 | 0.03224147 | 0.233618934 |
| 51411 | NM_001290007BIN2 bridging integrator 2 | 6.560784 | -0.195050 | 0.004595519 | 0.06089578 |
| 440452 | NR_027486 TBC1D3P2 TBC1 domain family member 3 pseudogene 2 | 6.574292 | -1.320094 | 0.005506886 | 0.069414954 |
| 319089 | NM_001310135TTC6 tetratricopeptide repeat domain 6 | 6.580301 | 0.856574 | 0.002652741 | 0.041444069 |
| 345079 | NM_001029870SOWAHB sosondowah ankyrin repeat domain family member B | 6.589270 | -0.671822 | 0.004381448 | 0.059119544 |
| 79698 | NM_001135731ZMAT4 zinc finger matrin-type 4 | 6.603191 | 1.216521 | 0.002633634 | 0.041238993 |
| 5346 | NM_001145311PLIN1 perilipin 1 | 6.609286 | -1.215800 | 0.005669237 | 0.070985578 |
| 114038 | NR_026863 LINC00313 long intergenic non-protein coding RNA 313 | 6.619894 | 0.327820 | 0.003044047 | 0.045136999 |
| 646214 | NR_027053 LOC646214 p21 protein (Cdc42/Rac)-activated kinase 2 pseudogene | 6.635154 | 1.329755 | 0.002474793 | 0.039377759 |
| 3929 | NM_004139 LBP lipopolysaccharide binding protein | 6.638519 | -0.792223 | 0.004030544 | 0.055433373 |
| 220594 | NR_003554 USP32P2 ubiquitin specific peptidase 32 pseudogene 2 | 6.650808 | 2.975184 | 0.002207266 | 0.036130384 |
| 11074 | NM_007028,NRTRIM31 tripartite motif containing 31 | 6.652804 | 3.199431 | 0.0023107 | 0.037341279 |
| 4060 | NM_002345 LUM lumican | 6.693962 | 3.534351 | 0.002066287 | 0.03463626 |
| 100302263 | NR_031729 MIR1908 microRNA 1908 | 6.699524 | -2.929551 | 0.020057392 | 0.171680039 |
| 388815 | NR_027790,NR MIR99AHG mir-99a-let-7c cluster host gene | 6.712035 | 0.694363 | 0.002750678 | 0.042688996 |
| 222171 | NM_001329996PRR15 proline rich 15 | 6.724529 | 3.938026 | 0.002005488 | 0.03378136 |
| 84070 | NM_032130,NRFAM186B family with sequence similarity 186 member B | 6.739463 | -1.438686 | 0.009812054 | 0.104673721 |
| 9938 | NM_001007231ARHGAP25 Rho GTPase activating protein 25 | 6.766307 | 0.583841 | 0.002895691 | 0.04343537 |
| 11209 | NR_027504 MST1P2 macrophage stimulating 1 pseudogene 2 | 6.782886 | 2.642686 | 0.002012453 | 0.033843562 |
| 10255 | NR_028032 HCG9 HLA complex group 9 (non-protein coding) | 6.784303 | -2.249007 | 0.014252316 | 0.137724153 |
| 414235 | NR_027151,NR PRR26 proline rich 26 | 6.788563 | 0.932367 | 0.002270105 | 0.036795898 |
| 6524 | NM_003041,NRSLC5A2 solute carrier family 5 member 2 | 6.789745 | -1.681616 | 0.005729603 | 0.071438726 |
| 118663 | NM_001318189BTBD16 BTB domain containing 16 | 6.792158 | 0.026255 | 0.0035492 | 0.051053686 |
| 57121 | NM_001142961LPAR5 lysophosphatidic acid receptor 5 | 6.817552 | 2.244131 | 0.002091424 | 0.034972602 |
| 3784 | NM_000218,N KCNQ1 potassium voltage-gated channel subfamily Q member 1 | 6.827918 | 0.679589 | 0.002549097 | 0.040250432 |
| 497190 | NM_001011880CLEC18B C-type lectin domain family 18 member B | 6.833319 | -1.011576 | 0.004237983 | 0.057597029 |
| 3078 | NM_002113 CFHR1 complement factor H related 1 | 6.838379 | 1.830220 | 0.00203956 | 0.034215984 |
| 134121 | NM_001089584C5orf49 chromosome 5 open reading frame 49 | 6.841404 | -2.227750 | 0.011004551 | 0.114843537 |
| 1365 | NM_001306 CLDN3 claudin 3 | 6.845292 | 2.266797 | 0.002035837 | 0.034209003 |
| 339942 | NR_026991 H1FX-AS1 H1FX antisense RNA 1 | 6.850950 | 0.040022 | 0.002804324 | 0.0427381 |
| 90634 | NM_001079691N4BP2L1 NEDD4 binding protein 2 like 1 | 6.852947 | -0.194026 | 0.003037627 | 0.045074119 |
| 79957 | NM_001272104PAQR6 progestin and adipoQ receptor family member 6 | 6.852966 | 4.000896 | 0.001831541 | 0.031310274 |
| 109286553 | NR_026899 ARHGAP27P1-BP ARHGAP27P1-BPTFP1-KPNA2P3 readthrough, transcribed p | 6.856243 | 5.107715 | 0.00179123 | 0.030722712 |
| 9914 | NM_001286527ATP2C2 ATPase secretory pathway Ca2+ transporting 2 | 6.856268 | -0.767279 | 0.003560033 | 0.051173934 |
| 285696 | NR_027253 LOC285696 uncharacterized LOC285696 | 6.866508 | -0.426237 | 0.003854741 | 0.053699744 |
| 100505633 | NR_038849 LINC01133 long intergenic non-protein coding RNA 1133 | 6.867481 | 0.158293 | 0.002595185 | 0.040853431 |
| 51700 | NM_001302826CYB5R2 cytochrome b5 reductase 2 | 6.870156 | 1.662436 | 0.002231332 | 0.036256953 |
| 2266 | NM_000509,N FGG fibrinogen gamma chain | 6.878795 | 5.626026 | 0.001756335 | 0.030310349 |
| 6369 | NM_002991 CCL24 C-C motif chemokine ligand 24 | 6.886516 | -2.964863 | 0.027120138 | 0.209633802 |
| 79722 | NM_024669 ANKRD55 ankyrin repeat domain 55 | 6.886516 | -2.440265 | 0.027120138 | 0.209633802 |

| 283971 | NM_173619 | CLEC18C | C-type lectin domain family 18 member C | 6.886516 | -2.857417 | 0.027120138 | 0.209633802 |
| --- | --- | --- | --- | --- | --- | --- | --- |
| 340156 | NM_001012418MYLK4 | | myosin light chain kinase family member 4 | 6.893656 | 0.255154 | 0.002674902 | 0.041725954 |
| 3821 | NM_001304448KLRC1 | | killer cell lectin like receptor C1 | 6.896518 | -0.490972 | 0.004064904 | 0.055757652 |
| 5657 | NM_002777 PRTN3 | | proteinase 3 | 6.896518 | -0.574053 | 0.004064904 | 0.055757652 |
| 80125 | NM_001287181CCDC33 | | coiled-coil domain containing 33 | 6.919859 | -0.630451 | 0.004624411 | 0.061202582 |
| 9823 | NM_001282231ARMCX2 | | armadillo repeat containing, X-linked 2 | 6.946522 | -2.181422 | 0.017459041 | 0.154065215 |
| 8722 | NM_003793 CTSF | | cathepsin F | 6.958628 | 5.647377 | 0.001662463 | 0.029205285 |
| 84960 | NM_001039374CCDC183 | | coiled-coil domain containing 183 | 6.959443 | 1.816313 | 0.001879006 | 0.031963193 |
| 255488 | NM_182757 RNF144B | | ring finger protein 144B | 6.975127 | 2.583321 | 0.001764648 | 0.030367505 |
| 79674 | NM_001167911VEPH1 | | ventricular zone expressed PH domain containing 1 | 6.975482 | 1.589995 | 0.001987544 | 0.033533731 |
| 57571 | NM_001166222CARNS1 | | carnosine synthase 1 | 6.994378 | -0.466588 | 0.003334052 | 0.048603612 |
| 1285 | NM_000091 COL4A3 | | collagen type IV alpha 3 chain | 7.000527 | 4.139689 | 0.001641107 | 0.029038743 |
| 64097 | NM_001347887EPB41L4A | | erythrocyte membrane protein band 4.1 like 4A | 7.002551 | 4.263198 | 0.00164958 | 0.029089147 |
| 8938 | NM_001199096BAIAP3 | | BAI1 associated protein 3 | 7.002853 | 2.354450 | 0.001809026 | 0.030959154 |
| 51276 | NM_001290314ZNF571 | | zinc finger protein 571 | 7.003461 | 0.830189 | 0.002482637 | 0.039472206 |
| 4888 | NR_002713 NPY6R | | neuropeptide Y receptor Y6 (pseudogene) | 7.007009 | -0.434984 | 0.003142139 | 0.046226988 |
| 200197 | NR_027136 TMEM51-AS1 | | TMEM51 antisense RNA 1 | 7.023089 | 1.981219 | 0.001800493 | 0.03085601 |
| 2660 | NM_005259 MSTN | | myostatin | 7.038862 | -1.984154 | 0.006537408 | 0.077671618 |
| 23150 | NM_015123 FRMD4B | | FERM domain containing 4B | 7.040571 | 3.463891 | 0.001635508 | 0.028964449 |
| 3866 | NM_002275 KRT15 | | keratin 15 | 7.042236 | 3.944115 | 0.001614353 | 0.028663426 |
| 7464 | NM_003389,N CORO2A | | coronin 2A | 7.046453 | 4.228903 | 0.001595101 | 0.028370305 |
| 4909 | NM_006179 NTF4 | | neurotrophin 4 | 7.049234 | -0.541870 | 0.003131305 | 0.046133225 |
| 5909 | NM_001145657RAP1GAP | | RAP1 GTPase activating protein | 7.055561 | 6.539017 | 0.001546384 | 0.027718328 |
| 100126351 | NR_030635 MIR939 | | microRNA 939 | 7.057675 | -1.591874 | 0.005243495 | 0.066827913 |
| 105377105 | NR_135534 FLNB-AS1 | | FLNB antisense RNA 1 | 7.070282 | -1.496309 | 0.004466789 | 0.059785619 |
| 2022 | NM_000118,N ENG | | endoglin | 7.075145 | -1.250007 | 0.004186383 | 0.05700812 |
| 143689 | NM_152431 PIWIL4 | | piwi like RNA-mediated gene silencing 4 | 7.076504 | -0.074875 | 0.002513168 | 0.039830432 |
| 834 | NM_001223,N CASP1 | | caspase 1 | 7.079320 | -0.510335 | 0.00395413 | 0.054636721 |
| 440078 | NR_026788 FAM66C | | family with sequence similarity 66 member C | 7.110245 | 1.472769 | 0.001781345 | 0.030578517 |
| 255394 | NM_001286262TCP11L2 | | t-complex 11 like 2 | 7.115643 | 3.905322 | 0.001534996 | 0.02756198 |
| 284723 | NM_207348 SLC25A34 | | solute carrier family 25 member 34 | 7.116166 | 0.383217 | 0.002164759 | 0.036002479 |
| 10912 | NM_006705 GADD45G | | growth arrest and DNA damage inducible gamma | 7.151467 | -0.237695 | 0.003776467 | 0.052852652 |
| 23037 | NM_178140 PDZD2 | | PDZ domain containing 2 | 7.160973 | -1.841702 | 0.005416473 | 0.068358604 |
| 100507410 | NR_040018,NR C1QTNF1-AS1 | | C1QTNF1 antisense RNA 1 | 7.168511 | -0.513228 | 0.007555632 | 0.087458444 |
| 7576 | NM_006969,NRZNF28 | | zinc finger protein 28 | 7.175853 | 2.242393 | 0.001696576 | 0.029715214 |
| 5176 | NM_001329903SERPINF1 | | serpin family F member 1 | 7.189964 | 1.939655 | 0.001591309 | 0.028327217 |
| 138948 | NR_102735 DBH-AS1 | | DBH antisense RNA 1 | 7.193520 | -2.966595 | 0.015240223 | 0.144078619 |
| 345193 | NM_198506 LRIT3 | | leucine rich repeat, Ig-like and transmembrane domains 3 | 7.212523 | -3.163932 | 0.022910824 | 0.18519359 |
| 109616972 | NR_145718 SNORA68B | | small nucleolar RNA, H/ACA box 68B | 7.212523 | -3.002685 | 0.022910824 | 0.18519359 |
| 79661 | NM_001256552NEIL1 | | nei like DNA glycosylase 1 | 7.220167 | 2.054747 | 0.00155883 | 0.027820876 |
| 100499484 | NR_036526 LOC100499484 | | SUGT1-1300002K09Rik pseudogene | 7.247570 | -0.322208 | 0.002826714 | 0.0427381 |
| 728175 | NR_040108 LINC02363 | | long intergenic non-protein coding RNA 2363 | 7.249400 | -3.343630 | 0.041756881 | 0.278177479 |
| 101929431 | NR_120660 LOC101929431 | | uncharacterized LOC101929431 | 7.249400 | -2.926762 | 0.041756881 | 0.278177479 |

| 730091 | NR_038387 LINC00886 | long intergenic non-protein coding RNA 886 | 7.250380 | 2.703044 | 0.001514961 | 0.027344656 |
| --- | --- | --- | --- | --- | --- | --- |
| 1028 | NM_000076,N CDKN1C | cyclin dependent kinase inhibitor 1C | 7.251535 | 0.192447 | 0.002330548 | 0.037603259 |
| 728047 | NM_001277308GOLGA8O | golgin A8 family member O | 7.253045 | 3.417934 | 0.001411502 | 0.02565634 |
| 3215 | NM_002147 HOXB5 | homeobox B5 | 7.280894 | 3.348263 | 0.001387282 | 0.025331018 |
| 954 | NM_001246,N ENTPD2 | ectonucleoside triphosphate diphosphohydrolase 2 | 7.281779 | 2.815300 | 0.001413775 | 0.025675088 |
| 100288181 | NR_038925 LOC100288181 | uncharacterized LOC100288181 | 7.301066 | 2.727262 | 0.001398974 | 0.025450996 |
| 4128 | NM_000240,N MAOA | monoamine oxidase A | 7.303763 | 4.356414 | 0.001339697 | 0.024742521 |
| 126432 | NM_001195833RINL | Ras and Rab interactor like | 7.309940 | 3.852257 | 0.001342445 | 0.024771166 |
| 57214 | NM_001293298CEMIP | cell migration inducing hyaluronan binding protein | 7.328218 | 4.906170 | 0.001291669 | 0.024048755 |
| 692247 | NR_147607,NR LOC692247 | uncharacterized LOC692247 | 7.330566 | 0.787267 | 0.001613051 | 0.028663426 |
| 6898 | NM_000353 TAT | tyrosine aminotransferase | 7.338912 | -1.021859 | 0.003058065 | 0.04527994 |
| 54490 | NM_001207004UGT2B28 | UDP glucuronosyltransferase family 2 member B28 | 7.340487 | -2.444565 | 0.008202272 | 0.093581908 |
| 101409256 | NR_102424 LOC101409256 | cell division cycle 42 pseudogene | 7.340487 | -2.184126 | 0.008202272 | 0.093581908 |
| 100506472 | NR_040535 LOC100506472 | uncharacterized LOC100506472 | 7.368813 | 0.383889 | 0.001869884 | 0.031834193 |
| 91828 | NM_001077594EXOC3L4 | exocyst complex component 3 like 4 | 7.374540 | 1.099836 | 0.001555836 | 0.027791419 |
| 100996425 | NR_131947,NR LINC02170 | long intergenic non-protein coding RNA 2170 | 7.380746 | -2.226890 | 0.01012814 | 0.107381125 |
| 55586 | NM_017584 MIOX | myo-inositol oxygenase | 7.384322 | -1.791496 | 0.004734598 | 0.062379084 |
| 79937 | NM_033655 CNTNAP3 | contactin associated protein-like 3 | 7.387052 | 6.404743 | 0.001232854 | 0.023162195 |
| 441389 | NR_038977 LINC01239 | long intergenic non-protein coding RNA 1239 | 7.387887 | 0.530047 | 0.001833743 | 0.031322033 |
| 29933 | NM_001278694GPR132 | G protein-coupled receptor 132 | 7.443719 | -0.284734 | 0.002090632 | 0.034972602 |
| 27293 | NM_001009568SMPDL3B | sphingomyelin phosphodiesterase acid like 3B | 7.445288 | 2.894307 | 0.001274047 | 0.023820337 |
| 169436 | NM_153710,NRSTKLD1 | serine/threonine kinase like domain containing 1 | 7.465310 | -0.857044 | 0.002825579 | 0.0427381 |
| 259 | NM_001633 AMBP | alpha-1-microglobulin/bikunin precursor | 7.468975 | 2.362229 | 0.001259009 | 0.023610701 |
| 101926889 | NR_109994 LOC101926889 | uncharacterized LOC101926889 | 7.487205 | -0.992449 | 0.002913748 | 0.043516884 |
| 5071 | NM_004562,N PRKN | parkin RBR E3 ubiquitin protein ligase | 7.490513 | -1.380321 | 0.003443231 | 0.049841309 |
| 100532724 | NR_037804 NPHP3-ACAD11 | NPHP3-ACAD11 readthrough (NMD candidate) | 7.505182 | -0.109805 | 0.001979778 | 0.033429968 |
| 100996511 | NR_110616 LINC01355 | long intergenic non-protein coding RNA 1355 | 7.523355 | 1.677090 | 0.001305781 | 0.024267816 |
| 8876 | NM_004666 VNN1 | vanin 1 | 7.531649 | 1.933748 | 0.00229647 | 0.037140335 |
| 100528021 | NM_001199760ST20-MTHFS | ST20-MTHFS readthrough | 7.541218 | -0.583054 | 0.002498216 | 0.03962852 |
| 7984 | NM_005435 ARHGEF5 | Rho guanine nucleotide exchange factor 5 | 7.543390 | 2.335893 | 0.001269698 | 0.023768055 |
| 100131366 | NR_033938 LINC00605 | long intergenic non-protein coding RNA 605 | 7.560905 | -0.643954 | 0.003862936 | 0.05377176 |
| 7276 | NM_000371 TTR | transthyretin | 7.562424 | -0.212028 | 0.001881846 | 0.031985198 |
| 105372795 | NR_131189 LOC105372795 | uncharacterized LOC105372795 | 7.574559 | -1.238372 | 0.003272616 | 0.047840326 |
| 646405 | NR_026730 TPTE2P1 | transmembrane phosphoinositide 3-phosphatase and tensi | 7.589264 | 0.341039 | 0.001741117 | 0.030261321 |
| 8911 | NM_001003406CACNA1I | calcium voltage-gated channel subunit alpha1 I | 7.597444 | -0.401451 | 0.002412622 | 0.038626225 |
| 2065 | NM_001005915ERBB3 | erb-b2 receptor tyrosine kinase 3 | 7.621012 | 5.363932 | 0.001059849 | 0.020799779 |
| 144571 | NR_026971,NR A2M-AS1 | A2M antisense RNA 1 (head to head) | 7.637939 | 1.051516 | 0.001308408 | 0.02427302 |
| 9127 | NM_001159554P2RX6 | purinergic receptor P2X 6 | 7.649322 | 0.662477 | 0.001714023 | 0.029995398 |
| 5444 | NM_000446 PON1 | paraoxonase 1 | 7.652419 | 1.350585 | 0.001229487 | 0.023119951 |
| 57232 | NM_001037735ZNF630 | zinc finger protein 630 | 7.659703 | 0.619130 | 0.001519802 | 0.027384238 |
| 130271 | NM_172069 PLEKHH2 | pleckstrin homology, MyTH4 and FERM domain containing | 7.678975 | 5.881273 | 0.001017568 | 0.020103527 |
| 64518 | NM_031898 TEKT3 | tektin 3 | 7.687516 | -2.521911 | 0.011706775 | 0.118586996 |
| 284443 | NM_001076678ZNF493 | zinc finger protein 493 | 7.689337 | -0.830821 | 0.002574789 | 0.040594146 |

| 221416 | NM_001171992C6orf223 | chromosome 6 open reading frame 223 | 7.696271 | 3.205325 | 0.00105224 | 0.02067007 |
| --- | --- | --- | --- | --- | --- | --- |
| 27165 | NM_001280796GLS2 | glutaminase 2 | 7.705746 | -1.430218 | 0.003256853 | 0.047711056 |
| 105374285 | NR_146718 LINC02038 | long intergenic non-protein coding RNA 2038 | 7.719344 | -1.859464 | 0.003889019 | 0.054022944 |
| 4056 | NM_145867 LTC4S | leukotriene C4 synthase | 7.728737 | -3.443763 | 0.033664757 | 0.237988891 |
| 153163 | NR_051996 MGC32805 | uncharacterized LOC153163 | 7.728737 | -3.079618 | 0.033664757 | 0.237988891 |
| 102465474 | NR_106849 MIR6791 | microRNA 6791 | 7.728737 | -3.392925 | 0.033664757 | 0.237988891 |
| 102723859 | NM_001291466TBC1D3E | TBC1 domain family member 3E | 7.735179 | 4.228168 | 0.001006474 | 0.019934819 |
| 288 | NM_001149,N ANK3 | ankyrin 3 | 7.756422 | 4.272648 | 0.000998637 | 0.019862318 |
| 100129842 | NM_001159293ZNF737 | zinc finger protein 737 | 7.762196 | -1.160008 | 0.003431927 | 0.049712466 |
| 338821 | NM_001009562SLCO1B7 | solute carrier organic anion transporter family member 1B7 | 7.785694 | 3.270868 | 0.000991995 | 0.019806384 |
| 9249 | NM_001319225DHRS3 | dehydrogenase/reductase 3 | 7.790223 | 6.241173 | 0.000946258 | 0.018966412 |
| 9595 | NM_004288 CYTIP | cytohesin 1 interacting protein | 7.797346 | -1.392198 | 0.003086039 | 0.045628817 |
| 101928710 | NR_110848,NR LINC02081 | long intergenic non-protein coding RNA 2081 | 7.826698 | -0.795194 | 0.002816464 | 0.0427381 |
| 100526794 | NM_001199103NT5C1B-RDH14 | NT5C1B-RDH14 readthrough | 7.854655 | 2.280554 | 0.001024665 | 0.020224421 |
| 9388 | NM_001308006LIPG | lipase G, endothelial type | 7.860273 | 1.507451 | 0.00108093 | 0.021193393 |
| 285961 | NR_027269 SEPT7P9 | septin 7 pseudogene 9 | 7.864537 | -3.003012 | 0.016551802 | 0.148535369 |
| 339192 | NR_147507 LOC339192 | uncharacterized LOC339192 | 7.899721 | 1.953770 | 0.001005394 | 0.019934819 |
| 3695 | NM_000889,NRITGB7 | integrin subunit beta 7 | 7.908294 | 0.529370 | 0.001319006 | 0.024447706 |
| 84985 | NM_001288587FAM83A | family with sequence similarity 83 member A | 7.993189 | 3.491239 | 0.000869108 | 0.017856468 |
| 90865 | NM_001199640IL33 | interleukin 33 | 8.017228 | -1.142427 | 0.002228217 | 0.0362348 |
| 9481 | NM_001204051SLC25A27 | solute carrier family 25 member 27 | 8.021555 | 2.456544 | 0.000916949 | 0.018450479 |
| 64090 | NM_022134 GAL3ST2 | galactose-3-O-sulfotransferase 2 | 8.026103 | -0.449841 | 0.001571627 | 0.028025086 |
| 728378 | NM_001099771POTEF | POTE ankyrin domain family member F | 8.026608 | -1.589670 | 0.004264421 | 0.057880282 |
| 221400 | NM_001010870TDRD6 | tudor domain containing 6 | 8.034101 | -0.670940 | 0.002002572 | 0.033759747 |
| 286499 | NM_001171109FAM133A | family with sequence similarity 133 member A | 8.039203 | 3.470691 | 0.000923237 | 0.018540934 |
| 80129 | NM_025059 CCDC170 | coiled-coil domain containing 170 | 8.047250 | -0.168733 | 0.001837507 | 0.031360424 |
| 11069 | NM_001100397RAPGEF4 | Rap guanine nucleotide exchange factor 4 | 8.059863 | 1.452380 | 0.000955837 | 0.019139864 |
| 352954 | NM_178831,NRGATS | GATS, stromal antigen 3 opposite strand | 8.075690 | 5.831518 | 0.000789606 | 0.016365741 |
| 4868 | NM_004646 NPHS1 | NPHS1, nephrin | 8.113323 | 2.737661 | 0.000899412 | 0.018158517 |
| 10398 | NM_006097,N MYL9 | myosin light chain 9 | 8.122283 | 0.619970 | 0.001333289 | 0.024646193 |
| 147968 | NM_144691 CAPN12 | calpain 12 | 8.132567 | 4.022408 | 0.000785066 | 0.016304316 |
| 5196 | NM_002619 PF4 | platelet factor 4 | 8.169624 | -1.315427 | 0.003945405 | 0.054552613 |
| 275 | NM_000481,N AMT | aminomethyltransferase | 8.176664 | 1.680419 | 0.0008693 | 0.017856468 |
| 820 | NM_004345 CAMP | cathelicidin antimicrobial peptide | 8.190544 | -3.164443 | 0.014149938 | 0.137027842 |
| 104326051 | NR_126358 MAFA-AS1 | MAFA antisense RNA 1 | 8.190544 | -2.892990 | 0.014149938 | 0.137027842 |
| 64129 | NM_001204414TINAGL1 | tubulointerstitial nephritis antigen like 1 | 8.206014 | 3.666501 | 0.000786253 | 0.016312574 |
| 56097 | NM_018929,N PCDHGC5 | protocadherin gamma subfamily C, 5 | 8.208074 | -3.393105 | 0.027322057 | 0.210330017 |
| 677771 | NR_003005 SCARNA4 | small Cajal body-specific RNA 4 | 8.208074 | -3.496632 | 0.027322057 | 0.210330017 |
| 102723344 | NR_120375 LOC102723344 | uncharacterized LOC102723344 | 8.208074 | -2.891292 | 0.027322057 | 0.210330017 |
| 6095 | NM_002943,N RORA | RAR related orphan receptor A | 8.226675 | 1.212541 | 0.000907035 | 0.018268757 |
| 284021 | NM_001085423MILR1 | mast cell immunoglobulin like receptor 1 | 8.313080 | 1.747232 | 0.000859002 | 0.017697657 |
| 8622 | NM_001029851PDE8B | phosphodiesterase 8B | 8.316963 | 4.229476 | 0.000695363 | 0.014824777 |
| 140628 | NM_080473 GATA5 | GATA binding protein 5 | 8.348571 | 0.839628 | 0.001044326 | 0.020573225 |

| 28984 | NM_014059 | RGCC | regulator of cell cycle | 8.354894 | 0.832868 | 0.000936905 | 0.018797176 |
| --- | --- | --- | --- | --- | --- | --- | --- |
| 55876 | NM_001042471GSDMB | | gasdermin B | 8.361037 | 3.601599 | 0.000691885 | 0.014800051 |
| 101059918 | NM_001282484GOLGA8R | | golgin A8 family member R | 8.417351 | 2.964547 | 0.00068647 | 0.014714641 |
| 114769 | NM_001017534CARD16 | | caspase recruitment domain family member 16 | 8.455656 | -1.245891 | 0.003386477 | 0.049122915 |
| 79961 | NM_001271833DENND2D | | DENN domain containing 2D | 8.489873 | 3.091894 | 0.000658979 | 0.014258347 |
| 105221694 | NR_130765,NR BISPR | | BST2 interferon stimulated positive regulator (non-protein c | 8.505015 | -2.142282 | 0.004331767 | 0.058653633 |
| 5345 | NM_000934,N SERPINF2 | | serpin family F member 2 | 8.526104 | 2.514804 | 0.000671303 | 0.014464482 |
| 7448 | NM_000638 VTN | | vitronectin | 8.559146 | 5.657501 | 0.000586897 | 0.013081864 |
| 283587 | NR_046094 LINC01146 | | long intergenic non-protein coding RNA 1146 | 8.598672 | -2.061835 | 0.003141633 | 0.046226988 |
| 375775 | NM_001098537PNPLA7 | | patatin like phospholipase domain containing 7 | 8.634683 | 2.815835 | 0.000603187 | 0.013273321 |
| 64798 | NM_001283012DEPTOR | | DEP domain containing MTOR interacting protein | 8.646764 | 3.171729 | 0.000595467 | 0.013229577 |
| 89876 | NM_001320316MAATS1 | | MYCBP associated and testis expressed 1 | 8.669279 | -0.817916 | 0.002601882 | 0.040865545 |
| 285735 | NR_026969 LINC00326 | | long intergenic non-protein coding RNA 326 | 8.669279 | -1.389999 | 0.002601882 | 0.040865545 |
| 56961 | NM_020209 SHD | | Src homology 2 domain containing transforming protein D | 8.675507 | -2.759929 | 0.007116069 | 0.08326755 |
| 123904 | NM_001348682NRN1L | | neuritin 1 like | 8.675507 | -2.666277 | 0.007116069 | 0.08326755 |
| 122773 | NM_172193 KLHDC1 | | kelch domain containing 1 | 8.676224 | 1.737507 | 0.00065718 | 0.014234321 |
| 100130370 | NM_001272086LOC100130370 | | uncharacterized LOC100130370 | 8.687411 | -3.444127 | 0.022314819 | 0.184819135 |
| 57468 | NM_001134771SLC12A5 | | solute carrier family 12 member 5 | 8.724112 | 0.586154 | 0.000865183 | 0.017807266 |
| 65983 | NM_001146319GRAMD2B | | GRAM domain containing 2B | 8.734008 | 3.248844 | 0.000594704 | 0.013229577 |
| 84189 | NM_032229 SLITRK6 | | SLIT and NTRK like family member 6 | 8.760114 | 1.375032 | 0.0006267 | 0.013703274 |
| 401145 | NM_001145065CCSER1 | | coiled-coil serine rich protein 1 | 8.768655 | -1.108340 | 0.001958322 | 0.033094676 |
| 55805 | NM_018409 LRP2BP | | LRP2 binding protein | 8.772446 | -1.787938 | 0.004860852 | 0.062881001 |
| 101926943 | NR_110044 LOC101926943 | | uncharacterized LOC101926943 | 8.794695 | -1.727010 | 0.002436393 | 0.038916433 |
| 7955 | NR_026876 RNF217-AS1 | | RNF217 antisense RNA 1 (head to head) | 8.810843 | -0.900917 | 0.001423127 | 0.025799629 |
| 57639 | NM_020879 CCDC146 | | coiled-coil domain containing 146 | 8.815000 | 3.549687 | 0.000527906 | 0.01189514 |
| 7837 | NM_012293 PXDN | | peroxidasin | 8.830237 | 4.711387 | 0.000515917 | 0.011675864 |
| 219527 | NM_001005210LRRC55 | | leucine rich repeat containing 55 | 8.842559 | -0.891944 | 0.010453099 | 0.109980851 |
| 644990 | NR_046244 THRB-AS1 | | THRB antisense RNA 1 | 8.842559 | -2.790890 | 0.010453099 | 0.109980851 |
| 153478 | NM_052909 PLEKHG4B | | pleckstrin homology and RhoGEF domain containing G4B | 8.848808 | 0.879317 | 0.000731181 | 0.01532333 |
| 84734 | NM_032648 FAM167B | | family with sequence similarity 167 member B | 8.857097 | 0.085822 | 0.000870935 | 0.017858119 |
| 9615 | NM_001242505GDA | | guanine deaminase | 8.859506 | 4.788646 | 0.000500755 | 0.01142019 |
| 8991 | NM_001258288SELENBP1 | | selenium binding protein 1 | 8.869781 | 5.231929 | 0.000490517 | 0.01131223 |
| 4582 | NM_001018016MUC1 | | mucin 1, cell surface associated | 8.901110 | 3.017757 | 0.000515359 | 0.011675864 |
| 83875 | NM_001037290BCO2 | | beta-carotene oxygenase 2 | 8.943542 | 0.743984 | 0.000723103 | 0.01532333 |
| 57453 | NM_020693 DSCAML1 | | DS cell adhesion molecule like 1 | 8.952565 | 1.614232 | 0.000552399 | 0.012393033 |
| 63895 | NM_022068 PIEZO2 | | piezo type mechanosensitive ion channel component 2 | 8.967669 | 3.274369 | 0.000496357 | 0.011332395 |
| 5239 | NM_021965 PGM5 | | phosphoglucomutase 5 | 8.971260 | -2.368551 | 0.004405204 | 0.059285385 |
| 388407 | NM_203425 C17orf82 | | chromosome 17 open reading frame 82 | 8.971260 | -2.296531 | 0.004405204 | 0.059285385 |
| 101929125 | NR_109859 LINC01730 | | long intergenic non-protein coding RNA 1730 | 9.002795 | 0.678878 | 0.00067966 | 0.014589822 |
| 101927843 | NR_109959 LINC01687 | | long intergenic non-protein coding RNA 1687 | 9.004098 | -1.856888 | 0.003355072 | 0.048769965 |
| 64093 | NM_001034852SMOC1 | | SPARC related modular calcium binding 1 | 9.016358 | 6.216482 | 0.000447881 | 0.010551729 |
| 79574 | NM_001319952EPS8L3 | | EPS8 like 3 | 9.036935 | 3.811632 | 0.000463583 | 0.01085981 |
| 93986 | NM_001172766FOXP2 | | forkhead box P2 | 9.061696 | 0.844185 | 0.000640983 | 0.013929631 |

| 100287846 | NR_037168 | LOC100287846 | patched 1 pseudogene | 9.070543 | 0.425397 | 0.000748212 | 0.015601572 |
| --- | --- | --- | --- | --- | --- | --- | --- |
| 643699 | NM_001282494GOLGA8N | | golgin A8 family member N | 9.093725 | 3.389977 | 0.000458751 | 0.010758794 |
| 4640 | NM_001256041MYO1A | | myosin IA | 9.131023 | 1.377436 | 0.000510916 | 0.011588052 |
| 283422 | NR_036555 LINC01559 | | long intergenic non-protein coding RNA 1559 | 9.158521 | 1.772080 | 0.000503147 | 0.011449509 |
| 113828 | NM_138435 FAM83F | | family with sequence similarity 83 member F | 9.166747 | -3.496999 | 0.018334706 | 0.159820227 |
| 729020 | NM_001143909RPEL1 | | ribulose-5-phosphate-3-epimerase like 1 | 9.166747 | -3.162960 | 0.018334706 | 0.159820227 |
| 7802 | NM_003462 DNALI1 | | dynein axonemal light intermediate chain 1 | 9.169503 | -2.578589 | 0.005623714 | 0.07054368 |
| 51285 | NM_001307930RASL12 | | RAS like family 12 | 9.169503 | -1.232036 | 0.005623714 | 0.07054368 |
| 54508 | NR_027706 EPB41L4A-AS2 | | EPB41L4A antisense RNA 2 (head to head) | 9.170075 | -2.320276 | 0.003998158 | 0.055061183 |
| 100506033 | NR_040037 PTOV1-AS1 | | PTOV1 antisense RNA 1 | 9.170737 | -1.649186 | 0.002346676 | 0.037811188 |
| 128344 | NM_001300831PIFO | | primary cilia formation | 9.170945 | -0.835173 | 0.002008336 | 0.033801816 |
| 105372476 | NR_136528,NR LOC105372476 | | uncharacterized LOC105372476 | 9.171512 | -0.891535 | 0.001245973 | 0.023387435 |
| 2028 | NM_001977 ENPEP | | glutamyl aminopeptidase | 9.172089 | 0.736780 | 0.000666794 | 0.014382302 |
| 6558 | NM_001046,N SLC12A2 | | solute carrier family 12 member 2 | 9.182931 | 8.223935 | 0.000405716 | 0.009751222 |
| 55304 | NM_001349945SPTLC3 | | serine palmitoyltransferase long chain base subunit 3 | 9.206753 | 2.584331 | 0.000453033 | 0.010647027 |
| 57593 | NM_001110514EBF4 | | early B-cell factor 4 | 9.233516 | 3.440063 | 0.000415162 | 0.009870838 |
| 6328 | NM_001081676SCN3A | | sodium voltage-gated channel alpha subunit 3 | 9.234424 | 0.872129 | 0.000572948 | 0.012798522 |
| 342892 | NM_001193552ZNF850 | | zinc finger protein 850 | 9.270273 | 1.417659 | 0.000662159 | 0.014297247 |
| 619351 | NR_026765 LINC00589 | | long intergenic non-protein coding RNA 589 | 9.284217 | -0.521767 | 0.000890838 | 0.018158517 |
| 57167 | NM_001318031SALL4 | | spalt like transcription factor 4 | 9.331797 | 3.422083 | 0.000400957 | 0.009700336 |
| 10388 | NM_014258 SYCP2 | | synaptonemal complex protein 2 | 9.364561 | 3.655662 | 0.000385912 | 0.009391279 |
| 60484 | NM_021817 HAPLN2 | | hyaluronan and proteoglycan link protein 2 | 9.368889 | -2.184163 | 0.003633928 | 0.051642736 |
| 368 | NM_001079528ABCC6 | | ATP binding cassette subfamily C member 6 | 9.390567 | 2.694338 | 0.000407416 | 0.009751222 |
| 407009 | NR_029638 MIR224 | | microRNA 224 | 9.416501 | -2.578733 | 0.005015008 | 0.064431951 |
| 100846978 | NR_047469,NR LINC00506 | | long intergenic non-protein coding RNA 506 | 9.421779 | -0.988310 | 0.001771198 | 0.03045489 |
| 9120 | NM_001174166SLC16A6 | | solute carrier family 16 member 6 | 9.447314 | 5.430142 | 0.00035665 | 0.008761654 |
| 285758 | NR_038863 LINC01268 | | long intergenic non-protein coding RNA 1268 | 9.451275 | 1.277323 | 0.000504028 | 0.011456941 |
| 6304 | NM_001131010SATB1 | | SATB homeobox 1 | 9.451947 | 0.615685 | 0.000617646 | 0.013533914 |
| 5625 | NM_001195226PRODH | | proline dehydrogenase 1 | 9.452060 | 2.768631 | 0.000378514 | 0.009254805 |
| 440503 | NM_001013706PLIN5 | | perilipin 5 | 9.456769 | -1.824324 | 0.002037955 | 0.034215984 |
| 64699 | NM_001256317TMPRSS3 | | transmembrane protease, serine 3 | 9.460164 | -0.183857 | 0.001008065 | 0.019934819 |
| 56110 | NM_018918,N PCDHGA5 | | protocadherin gamma subfamily A, 5 | 9.461310 | 0.180723 | 0.000738415 | 0.015428391 |
| 643827 | NR_111893 CNTNAP3P2 | | contactin associated protein-like 3 pseudogene 2 | 9.461683 | 6.310750 | 0.000348394 | 0.008589436 |
| 285084 | NR_038897 LINC01305 | | long intergenic non-protein coding RNA 1305 | 9.485524 | 2.078267 | 0.000401608 | 0.009700885 |
| 643641 | NM_001099220ZNF862 | | zinc finger protein 862 | 9.522258 | 4.844379 | 0.000343059 | 0.008488253 |
| 953 | NM_001098175ENTPD1 | | ectonucleoside triphosphate diphosphohydrolase 1 | 9.558497 | 1.140661 | 0.000478297 | 0.011141412 |
| 257629 | NM_145865 ANKS4B | | ankyrin repeat and sterile alpha motif domain containing 4 | 9.574086 | 2.757638 | 0.000354931 | 0.008729771 |
| 100129726 | NR_027251 LINC01126 | | long intergenic non-protein coding RNA 1126 | 9.633557 | 0.409215 | 0.000532249 | 0.011966934 |
| 3758 | NM_000220,N KCNJ1 | | potassium voltage-gated channel subfamily J member 1 | 9.646084 | -2.927562 | 0.015150304 | 0.143737334 |
| 23460 | NM_080284 ABCA6 | | ATP binding cassette subfamily A member 6 | 9.646084 | -3.344518 | 0.015150304 | 0.143737334 |
| 23500 | NM_001201427DAAM2 | | dishevelled associated activator of morphogenesis 2 | 9.646084 | -3.297002 | 0.015150304 | 0.143737334 |
| 1014 | NM_001204744CDH16 | | cadherin 16 | 9.656006 | 0.575731 | 0.000477063 | 0.011141412 |
| 51085 | NM_032951,N MLXIPL | | MLX interacting protein like | 9.656542 | 3.995091 | 0.000323813 | 0.008160663 |

| 400668 | NM_001308209PRSS57 | protease, serine 57 | 9.663499 | -2.550693 | 0.004481104 | 0.05982482 |
| --- | --- | --- | --- | --- | --- | --- |
| 3635 | NM_001017915INPP5D | inositol polyphosphate-5-phosphatase D | 9.671383 | 1.213417 | 0.000398252 | 0.009646181 |
| 624 | NM_000623 BDKRB2 | bradykinin receptor B2 | 9.679570 | 4.991123 | 0.000316417 | 0.008030784 |
| 401261 | NR_134654 LOC401261 | uncharacterized LOC401261 | 9.715532 | 0.425582 | 0.000546155 | 0.012266257 |
| 161753 | NM_175881 ODF3L1 | outer dense fiber of sperm tails 3 like 1 | 9.742801 | -1.679875 | 0.001775228 | 0.030498836 |
| 25759 | NM_012435 SHC2 | SHC adaptor protein 2 | 9.744082 | 2.393558 | 0.000341053 | 0.008456898 |
| 100533181 | NM_001204268FXYD6-FXYD2 | FXYD6-FXYD2 readthrough | 9.766518 | -1.963646 | 0.003014449 | 0.044794449 |
| 4013 | NM_001130142VWA5A | von Willebrand factor A domain containing 5A | 9.766889 | 0.982301 | 0.00045347 | 0.010647027 |
| 3426 | NM_000204,N CFI | complement factor I | 9.807265 | 2.580507 | 0.000313933 | 0.007977512 |
| 389434 | NM_001164694IYD | iodotyrosine deiodinase | 9.820580 | -2.635222 | 0.006803492 | 0.080188171 |
| 474383 | NM_001007523F8A2 | coagulation factor VIII-associated 2 | 9.820580 | -2.858830 | 0.006803492 | 0.080188171 |
| 56925 | NM_020169 LXN | latexin | 9.823624 | 5.686022 | 0.000287156 | 0.007415499 |
| 3034 | NM_001258333HAL | histidine ammonia-lyase | 9.870301 | -0.709009 | 0.000871107 | 0.017858119 |
| 406962 | NR_029707 MIR186 | microRNA 186 | 9.965332 | -2.040216 | 0.002751 | 0.042688996 |
| 204962 | NM_001130058SLC44A5 | solute carrier family 44 member 5 | 9.967598 | 2.534945 | 0.00030146 | 0.007688908 |
| 101929520 | NR_104061 LINC00993 | long intergenic non-protein coding RNA 993 | 9.977688 | 1.548806 | 0.000322547 | 0.008160663 |
| 6817 | NM_001055,N SULT1A1 | sulfotransferase family 1A member 1 | 9.992820 | 4.237896 | 0.000270472 | 0.007127011 |
| 100652929 | NR_109783 LINC02078 | long intergenic non-protein coding RNA 2078 | 10.002265 | -1.465816 | 0.001917416 | 0.032483003 |
| 776 | NM_000720,N CACNA1D | calcium voltage-gated channel subunit alpha1 D | 10.024542 | 4.337812 | 0.000264507 | 0.006996576 |
| 27147 | NM_001318052DENND2A | DENN domain containing 2A | 10.028833 | -1.365007 | 0.001550881 | 0.027726851 |
| 408 | NM_004041,N ARRB1 | arrestin beta 1 | 10.038117 | 5.478320 | 0.000256618 | 0.006840386 |
| 25837 | NM_001308053RAB26 | RAB26, member RAS oncogene family | 10.056956 | 3.710102 | 0.000265861 | 0.007023416 |
| 103752588 | NR_125801 PACERR | PTGS2 antisense NFKB1 complex-mediated expression regu | 10.064502 | -0.915579 | 0.001135603 | 0.021649727 |
| 1949 | NM_001406 EFNB3 | ephrin B3 | 10.084054 | 1.935057 | 0.000303268 | 0.007715976 |
| 6038 | NM_001282192RNASE4 | ribonuclease A family member 4 | 10.109608 | 5.686487 | 0.000246714 | 0.006670943 |
| 10692 | NM_006583 RRH | retinal pigment epithelium-derived rhodopsin homolog | 10.125421 | -2.927722 | 0.012586629 | 0.125356208 |
| 406886 | NR_029481 MIRLET7D | microRNA let-7d | 10.125421 | -3.080452 | 0.012586629 | 0.125356208 |
| 1051 | NM_001285878CEBPB | CCAAT/enhancer binding protein beta | 10.138861 | 5.863554 | 0.000248054 | 0.006685785 |
| 83988 | NM_001040624NCALD | neurocalcin delta | 10.143755 | 3.924974 | 0.000253476 | 0.006782862 |
| 10071 | NM_001164462MUC12 | mucin 12, cell surface associated | 10.144647 | 1.568984 | 0.000300074 | 0.007681957 |
| 101928063 | NR_110727 LOC101928063 | uncharacterized LOC101928063 | 10.171849 | -1.246915 | 0.001451105 | 0.026260805 |
| 971 | NM_001782 CD72 | CD72 molecule | 10.178947 | -0.763381 | 0.000873346 | 0.017886298 |
| 728377 | NR_033942 ARHGEF34P | Rho guanine nucleotide exchange factor 34, pseudogene | 10.187469 | 0.914548 | 0.000395752 | 0.009608134 |
| 7225 | NM_004621 TRPC6 | transient receptor potential cation channel subfamily C me | 10.220153 | 2.075399 | 0.000271888 | 0.007155213 |
| 105371795 | NR_135646 LOC105371795 | uncharacterized LOC105371795 | 10.228216 | 2.791337 | 0.000251429 | 0.006754286 |
| 55335 | NM_018376,NRNIPSNAP3B | nipsnap homolog 3B | 10.235160 | 2.568167 | 0.000262627 | 0.006965205 |
| 100507012 | NR_121610 BMPR1B-AS1 | BMPR1B antisense RNA 1 (head to head) | 10.249668 | -0.680012 | 0.000641629 | 0.013929631 |
| 2122 | NM_001105077MECOM | MDS1 and EVI1 complex locus | 10.255628 | 2.595091 | 0.000261854 | 0.006962017 |
| 10137 | NM_001198838RBM12 | RNA binding motif protein 12 | 10.261120 | 6.291605 | 0.000231118 | 0.006350145 |
| 828 | NM_004058,N CAPS | calcyphosine | 10.273669 | 2.834797 | 0.000245074 | 0.006635293 |
| 3108 | NM_006120 HLA-DMA | major histocompatibility complex, class II, DM alpha | 10.297858 | 2.716253 | 0.000251785 | 0.006755087 |
| 5334 | NM_006226 PLCL1 | phospholipase C like 1 | 10.334987 | -1.648705 | 0.001648214 | 0.029089147 |
| 165679 | NM_001040100SPTSSB | serine palmitoyltransferase small subunit B | 10.334987 | -1.823587 | 0.001648214 | 0.029089147 |

| 29774 | NR_003714 | POM121L9P | POM121 transmembrane nucleoporin like 9, pseudogene | 10.403215 | 1.348175 | 0.000269325 | 0.007105854 |
| --- | --- | --- | --- | --- | --- | --- | --- |
| 100130449 | NR_024014 | PP14571 | uncharacterized LOC100130449 | 10.404493 | -2.551122 | 0.003233264 | 0.047399055 |
| 50839 | NM_023921 | TAS2R10 | taste 2 receptor member 10 | 10.464959 | -0.288688 | 0.000490791 | 0.01131223 |
| 221883 | NR_002795 | HOXA11-AS | HOXA11 antisense RNA | 10.498863 | -0.281536 | 0.000524706 | 0.011848843 |
| 79608 | NM_001135109RIC3 | | RIC3 acetylcholine receptor chaperone | 10.500073 | 3.073730 | 0.000219086 | 0.006058537 |
| 114904 | NM_031910,N C1QTNF6 | | C1q and TNF related 6 | 10.501416 | 5.036247 | 0.000205509 | 0.005760092 |
| 3176 | NM_001024074HNMT | | histamine N-methyltransferase | 10.533418 | 2.003689 | 0.000242651 | 0.006578286 |
| 653073 | NM_001282472GOLGA8J | | golgin A8 family member J | 10.534094 | 1.625007 | 0.000253001 | 0.006778907 |
| 9752 | NM_014005,N PCDHA9 | | protocadherin alpha 9 | 10.561775 | -2.001839 | 0.002106798 | 0.035172821 |
| 364 | NM_001170,N AQP7 | | aquaporin 7 | 10.575091 | 1.065922 | 0.000281376 | 0.007386129 |
| 25999 | NM_001199570CLIP3 | | CAP-Gly domain containing linker protein 3 | 10.600338 | 3.130717 | 0.000206734 | 0.005786587 |
| 129807 | NM_001167599NEU4 | | neuraminidase 4 | 10.600897 | -1.695880 | 0.001193496 | 0.022586877 |
| 3273 | NM_000412 HRG | | histidine rich glycoprotein | 10.604758 | -2.964586 | 0.010510413 | 0.110415384 |
| 56667 | NM_033049 MUC13 | | mucin 13, cell surface associated | 10.617515 | 7.159768 | 0.000189243 | 0.005421732 |
| 84000 | NM_001077263TMPRSS13 | | transmembrane protease, serine 13 | 10.651490 | -2.523612 | 0.002910357 | 0.043497646 |
| 259294 | NM_176888 TAS2R19 | | taste 2 receptor member 19 | 10.651490 | -2.119726 | 0.002910357 | 0.043497646 |
| 100885782 | NR_047700 MYO16-AS1 | | MYO16 antisense RNA 1 | 10.651490 | -2.469837 | 0.002910357 | 0.043497646 |
| 339479 | NM_001317188BRINP3 | | BMP/retinoic acid inducible neural specific 3 | 10.667710 | -1.790399 | 0.001421769 | 0.025797619 |
| 5791 | NM_001316676PTPRE | | protein tyrosine phosphatase, receptor type E | 10.713084 | 2.762320 | 0.000210479 | 0.005851824 |
| 256021 | NR_046159,NR LINC01619 | | long intergenic non-protein coding RNA 1619 | 10.763831 | -0.156925 | 0.000385427 | 0.009391279 |
| 100505879 | NR_103750 KIF25-AS1 | | KIF25 antisense RNA 1 | 10.788685 | -0.147787 | 0.000480563 | 0.011181609 |
| 3689 | NM_000211,N ITGB2 | | integrin subunit beta 2 | 10.796777 | 2.083126 | 0.000204415 | 0.005737201 |
| 343450 | NM_001287819KCNT2 | | potassium sodium-activated channel subfamily T member 2 | 10.809239 | 2.061772 | 0.000203457 | 0.005721223 |
| 107987295 | NR_146322 LINC02246 | | long intergenic non-protein coding RNA 2246 | 10.840798 | 4.542527 | 0.000174366 | 0.005052482 |
| 401089 | NM_001040061FOXL2NB | | FOXL2 neighbor | 10.872362 | 2.128681 | 0.000200376 | 0.005677783 |
| 9965 | NM_005117 FGF19 | | fibroblast growth factor 19 | 10.882219 | -0.653550 | 0.000692814 | 0.014804603 |
| 347365 | NM_198510 ITIH6 | | inter-alpha-trypsin inhibitor heavy chain family member 6 | 10.886929 | -1.428473 | 0.001051105 | 0.020667411 |
| 25981 | NM_015512 DNAH1 | | dynein axonemal heavy chain 1 | 10.887313 | 4.209100 | 0.000172066 | 0.005044328 |
| 56139 | NM_018901,N PCDHA10 | | protocadherin alpha 10 | 10.957895 | -1.418197 | 0.000751399 | 0.015652249 |
| 340307 | NM_178561 CTAGE6 | | CTAGE family member 6 | 10.971629 | -3.859108 | 0.034780972 | 0.24354922 |
| 100500904 | NR_037507 MIR3942 | | microRNA 3942 | 10.971629 | -3.549869 | 0.034780972 | 0.24354922 |
| 10720 | NM_001073 UGT2B11 | | UDP glucuronosyltransferase family 2 member B11 | 11.005038 | -0.064141 | 0.000344102 | 0.008503873 |
| 400706 | NR_125344 LOC400706 | | uncharacterized LOC400706 | 11.011719 | 3.331228 | 0.000180682 | 0.00521257 |
| 84958 | NM_001193308SYTL1 | | synaptotagmin like 1 | 11.042370 | 1.470736 | 0.000194933 | 0.005561645 |
| 23639 | NM_001321961LRRC6 | | leucine rich repeat containing 6 | 11.091253 | 2.702618 | 0.0001744 | 0.005052482 |
| 3212 | NM_002145 HOXB2 | | homeobox B2 | 11.101125 | 1.887783 | 0.000179401 | 0.005182829 |
| 200172 | NM_001168247SLFNL1 | | schlafen like 1 | 11.124609 | -2.606498 | 0.00399788 | 0.055061183 |
| 148713 | NR_002930 PTPRVP | | protein tyrosine phosphatase, receptor type V, pseudogene | 11.166793 | -1.604956 | 0.001146185 | 0.021771195 |
| 84812 | NM_032726 PLCD4 | | phospholipase C delta 4 | 11.174766 | 1.682149 | 0.000180942 | 0.005212795 |
| 101929555 | NR_110873 LOC101929555 | | uncharacterized LOC101929555 | 11.197371 | -0.000061 | 0.000324571 | 0.008160663 |
| 6261 | NM_000540,N RYR1 | | ryanodine receptor 1 | 11.207304 | 2.402281 | 0.000173143 | 0.005044328 |
| 10346 | NM_001199573TRIM22 | | tripartite motif containing 22 | 11.217953 | 2.197143 | 0.000165749 | 0.004904888 |
| 101928607 | NR_109964 TRPM2-AS | | TRPM2 antisense RNA | 11.303029 | -1.077547 | 0.000741283 | 0.015472699 |

| 93664 | NM_001009571CADPS2 | calcium dependent secretion activator 2 | 11.322801 | 3.101297 | 0.000153058 | 0.004582618 |
| --- | --- | --- | --- | --- | --- | --- |
| 4586 | NM_001304359MUC5AC | mucin 5AC, oligomeric mucus/gel-forming | 11.329136 | 9.605116 | 0.000133316 | 0.004103627 |
| 101929374 | NR_110538 LINC01659 | long intergenic non-protein coding RNA 1659 | 11.356636 | -0.814795 | 0.000465937 | 0.010902607 |
| 100507472 | NR_045387 LOC100507472 | uncharacterized LOC100507472 | 11.404592 | -1.161836 | 0.00061713 | 0.013533914 |
| 284739 | NM_001348090C20orf204 | chromosome 20 open reading frame 204 | 11.446289 | 0.895264 | 0.000197964 | 0.005632585 |
| 653082 | NM_001351303ZDHHC11B | zinc finger DHHC-type containing 11B | 11.473567 | 4.051364 | 0.000129659 | 0.00400299 |
| 1087 | NM_001291485CEACAM7 | carcinoembryonic antigen related cell adhesion molecule 7 | 11.480275 | -0.767153 | 0.000383294 | 0.009360603 |
| 1050 | NM_001285829CEBPA | CCAAT/enhancer binding protein alpha | 11.485706 | 3.821581 | 0.000134289 | 0.004121311 |
| 1356 | NM_000096,NRCP | ceruloplasmin | 11.498742 | 7.061284 | 0.00012338 | 0.003902311 |
| 107303344 | NM_001320727SETDB2-PHF11 | SETDB2-PHF11 readthrough | 11.499515 | -1.126784 | 0.000996744 | 0.019854122 |
| 93010 | NM_145236 B3GNT7 | UDP-GlcNAc:betaGal beta-1,3-N-acetylglucosaminyltransfer | 11.560242 | 2.552388 | 0.000140471 | 0.004285611 |
| 4887 | NM_000910 NPY2R | neuropeptide Y receptor Y2 | 11.573246 | -0.403814 | 0.00032028 | 0.008118867 |
| 100128317 | NR_126025,NR LOC100128317 | uncharacterized LOC100128317 | 11.602010 | -1.621246 | 0.000773287 | 0.016091998 |
| 3872 | NM_000422 KRT17 | keratin 17 | 11.611195 | 2.622308 | 0.000134893 | 0.00413371 |
| 340061 | NM_001301738TMEM173 | transmembrane protein 173 | 11.620021 | 1.564163 | 0.000152 | 0.004563309 |
| 127733 | NM_152376 UBXN10 | UBX domain protein 10 | 11.664261 | 0.781210 | 0.000231166 | 0.006350145 |
| 8654 | NM_001083,N PDE5A | phosphodiesterase 5A | 11.679964 | 2.858299 | 0.000129229 | 0.004001664 |
| 1366 | NM_001185022CLDN7 | claudin 7 | 11.723119 | 1.888912 | 0.000140751 | 0.004287825 |
| 3249 | NM_002151,N HPN | hepsin | 11.746571 | 0.180239 | 0.000213666 | 0.00592451 |
| 339483 | NR_026850 MTMR9LP | myotubularin related protein 9-like, pseudogene | 11.754322 | 2.271305 | 0.000130782 | 0.004031641 |
| 105377736 | NR_146727 LINC01942 | long intergenic non-protein coding RNA 1942 | 11.764389 | 0.566806 | 0.000208 | 0.005806323 |
| 345557 | NM_001005473PLCXD3 | phosphatidylinositol specific phospholipase C X domain con | 11.766122 | 2.540527 | 0.000126849 | 0.003957588 |
| 100287102 | NR_046018 DDX11L1 | DEAD/H-box helicase 11 like 1 | 11.776624 | -2.759668 | 0.003112808 | 0.045943736 |
| 101929234 | NR_110304 LOC101929234 | uncharacterized LOC101929234 | 11.886480 | -2.000931 | 0.00176232 | 0.030352691 |
| 213 | NM_000477 ALB | albumin | 11.930112 | -1.355231 | 0.000568298 | 0.012708371 |
| 283710 | NM_001243538LOC283710 | uncharacterized LOC283710 | 11.935560 | 0.272338 | 0.000219827 | 0.006070933 |
| 84519 | NM_032489 ACRBP | acrosin binding protein | 12.007586 | -0.115380 | 0.000226203 | 0.006238684 |
| 3075 | NM_000186,N CFH | complement factor H | 12.013705 | 7.120187 | 9.73495E-05 | 0.003196309 |
| 140711 | NM_001304783TLDC2 | TBC/LysM-associated domain containing 2 | 12.014353 | 1.322072 | 0.000144847 | 0.004399654 |
| 6916 | NM_001061,N TBXAS1 | thromboxane A synthase 1 | 12.080413 | 4.759620 | 9.66314E-05 | 0.003182835 |
| 105369921 | NR_135014,NR LOC105369921 | uncharacterized LOC105369921 | 12.086156 | 0.721117 | 0.000169198 | 0.004992681 |
| 158800 | NM_139282 RHOXF1 | Rhox homeobox family member 1 | 12.102631 | -2.366744 | 0.002756531 | 0.042710739 |
| 150696 | NM_001165977PROM2 | prominin 2 | 12.152289 | -2.081345 | 0.001005703 | 0.019934819 |
| 200010 | NM_001011547SLC5A9 | solute carrier family 5 member 9 | 12.197097 | -0.832415 | 0.000324222 | 0.008160663 |
| 388551 | NM_001039213CEACAM16 | carcinoembryonic antigen related cell adhesion molecule 16 | 12.197097 | -0.774004 | 0.000324222 | 0.008160663 |
| 4485 | NM_020998,NRMST1 | macrophage stimulating 1 | 12.305850 | 2.293343 | 0.00010093 | 0.003308602 |
| 9420 | NM_001324112CYP7B1 | cytochrome P450 family 7 subfamily B member 1 | 12.329598 | 1.775950 | 0.000127758 | 0.003979938 |
| 100996307 | NR_073179,NR LIPE-AS1 | LIPE antisense RNA 1 | 12.331321 | -1.453987 | 0.000712016 | 0.015136743 |
| 6799 | NM_001054,N SULT1A2 | sulfotransferase family 1A member 2 | 12.346037 | -0.281516 | 0.000256079 | 0.006834839 |
| 51050 | NM_001324403PI15 | peptidase inhibitor 15 | 12.346037 | -0.142889 | 0.000256079 | 0.006834839 |
| 26040 | NM_001130110SETBP1 | SET binding protein 1 | 12.409758 | 3.546682 | 8.80533E-05 | 0.002956789 |
| 623 | NM_000710 BDKRB1 | bradykinin receptor B1 | 12.410852 | 1.779968 | 0.000107098 | 0.003483201 |
| 163255 | NM_001172225ZNF540 | zinc finger protein 540 | 12.428638 | -2.098190 | 0.002446565 | 0.039018654 |

| 3214 | NM_024015 | HOXB4 | homeobox B4 | 12.566753 | 0.814698 | 0.000129113 | 0.004001664 |
| --- | --- | --- | --- | --- | --- | --- | --- |
| 54511 | NM_001042406HMGCLL1 | | 3-hydroxymethyl-3-methylglutaryl-CoA lyase like 1 | 12.584430 | 0.389361 | 0.000207953 | 0.005806323 |
| 152195 | NR_002949,NR NUDT16P1 | | nudix hydrolase 16 pseudogene 1 | 12.678089 | 1.014465 | 0.000149476 | 0.004494054 |
| 26 | NM_001091,N AOC1 | | amine oxidase, copper containing 1 | 12.701374 | -0.718250 | 0.000263334 | 0.006974481 |
| 57653 | NR_036527,NR LOC100499484-C | | LOC100499484-C9orf174 readthrough | 12.770193 | 2.589208 | 8.27673E-05 | 0.002797453 |
| 9940 | NM_001321153DLEC1 | | deleted in lung and esophageal cancer 1 | 12.874471 | -2.120836 | 0.001211515 | 0.022844285 |
| 54905 | NM_017781 CYP2W1 | | cytochrome P450 family 2 subfamily W member 1 | 12.874471 | -2.206293 | 0.001211515 | 0.022844285 |
| 283 | NM_001097577ANG | | angiogenin | 12.879588 | 3.552045 | 7.20827E-05 | 0.002476795 |
| 4680 | NM_002483 CEACAM6 | | carcinoembryonic antigen related cell adhesion molecule 6 | 12.965682 | 10.253436 | 6.40288E-05 | 0.002299367 |
| 399671 | NM_001220484HEATR4 | | HEAT repeat containing 4 | 12.968030 | -0.944828 | 0.000323933 | 0.008160663 |
| 100506555 | NR_045114 PVRL3-AS1 | | PVRL3 antisense RNA 1 | 13.001442 | -2.857933 | 0.004569388 | 0.060705064 |
| 147923 | NM_001329515ZNF420 | | zinc finger protein 420 | 13.076442 | 3.305768 | 7.12444E-05 | 0.002456151 |
| 152273 | NM_001320276FGD5 | | FYVE, RhoGEF and PH domain containing 5 | 13.121469 | -2.184579 | 0.00110686 | 0.021383887 |
| 441046 | NR_003675 GUSBP5 | | glucuronidase, beta pseudogene 5 | 13.146361 | -1.388987 | 0.000690159 | 0.014778402 |
| 25803 | NM_001252294SPDEF | | SAM pointed domain containing ETS transcription factor | 13.162009 | 4.713314 | 6.09923E-05 | 0.002217511 |
| 219623 | NM_178505,NRTMEM26 | | transmembrane protein 26 | 13.191378 | -0.991756 | 0.000296957 | 0.007621112 |
| 753 | NM_001003674LDLRAD4 | | low density lipoprotein receptor class A domain containing | 13.224148 | 0.562067 | 0.000129522 | 0.00400299 |
| 1470 | NM_001322 CST2 | | cystatin SA | 13.264897 | 0.651585 | 0.000101898 | 0.00332978 |
| 101928068 | NR_111907 LOC101928068 | | uncharacterized LOC101928068 | 13.318531 | 2.390827 | 6.62401E-05 | 0.002358308 |
| 103344718 | NM_001293171HOTS | | H19 opposite tumor suppressor | 13.345175 | -1.965738 | 0.000641766 | 0.013929631 |
| 285359 | NR_002941 PDCL3P4 | | phosducin-like 3 pseudogene 4 | 13.428239 | -0.422800 | 0.000165671 | 0.004904888 |
| 634 | NM_001024912CEACAM1 | | carcinoembryonic antigen related cell adhesion molecule 1 | 13.458797 | 5.170249 | 5.34166E-05 | 0.001996869 |
| 339403 | NM_181885 RXFP4 | | relaxin/insulin like family peptide receptor 4 | 13.480778 | -3.003222 | 0.003915642 | 0.054322639 |
| 360 | NM_001318144AQP3 | | aquaporin 3 (Gill blood group) | 13.532907 | 6.678545 | 5.08196E-05 | 0.001943075 |
| 1593 | NM_000784 CYP27A1 | | cytochrome P450 family 27 subfamily A member 1 | 13.615465 | -2.040491 | 0.0008504 | 0.01753791 |
| 79887 | NM_024829 PLBD1 | | phospholipase B domain containing 1 | 13.652292 | 3.211925 | 5.58421E-05 | 0.002070061 |
| 83938 | NM_001305581LRMDA | | leucine rich melanocyte differentiation associated | 13.680385 | 1.994274 | 6.31221E-05 | 0.002274703 |
| 222223 | NM_001142749KIAA1324L | | KIAA1324 like | 13.707301 | 2.452985 | 6.06815E-05 | 0.002217511 |
| 10677 | NM_006576 AVIL | | advillin | 13.729478 | 2.172548 | 5.64093E-05 | 0.002087345 |
| 199964 | NM_182532 TMEM61 | | transmembrane protein 61 | 13.732667 | -2.204676 | 0.001550588 | 0.027726851 |
| 126147 | NM_145807 NTN5 | | netrin 5 | 13.785212 | -0.215933 | 0.000129035 | 0.004001664 |
| 644285 | NR_130929 LOC644285 | | uncharacterized LOC644285 | 13.788973 | -0.181365 | 0.00014421 | 0.004386741 |
| 5129 | NM_002596,N CDK18 | | cyclin dependent kinase 18 | 13.793804 | 3.456100 | 4.98515E-05 | 0.001909588 |
| 1369 | NM_001308 CPN1 | | carboxypeptidase N subunit 1 | 13.793974 | -0.742916 | 0.000166193 | 0.004911016 |
| 2006 | NM_000501,N ELN | | elastin | 13.820657 | 0.138117 | 0.000134244 | 0.004121311 |
| 3823 | NM_002261,N KLRC3 | | killer cell lectin like receptor C3 | 13.824603 | 1.772610 | 6.94712E-05 | 0.002430804 |
| 91461 | NM_138370 PKDCC | | protein kinase domain containing, cytoplasmic | 13.834182 | 5.889280 | 4.52101E-05 | 0.001781278 |
| 162966 | NM_001321866ZNF600 | | zinc finger protein 600 | 13.914261 | 0.787561 | 0.000107866 | 0.003497601 |
| 25884 | NM_001278473CHRDL2 | | chordin like 2 | 13.934549 | -0.648995 | 0.00017817 | 0.005154479 |
| 55064 | NM_001039395SPATA6L | | spermatogenesis associated 6 like | 13.936843 | 3.223449 | 4.72855E-05 | 0.001828226 |
| 55762 | NM_001172655ZNF701 | | zinc finger protein 701 | 13.941618 | -0.842961 | 0.000518533 | 0.011722235 |
| 392509 | NM_001162491ARL13A | | ADP ribosylation factor like GTPase 13A | 13.960115 | -2.695119 | 0.003367649 | 0.048918417 |
| 158584 | NM_174912 FAAH2 | | fatty acid amide hydrolase 2 | 14.022856 | 2.252599 | 5.24373E-05 | 0.001986565 |

| 9971 | NM_001206977NR1H4 | nuclear receptor subfamily 1 group H member 4 | 14.044254 | 0.271233 | 9.41263E-05 | 0.003115203 |
| --- | --- | --- | --- | --- | --- | --- |
| 100128398 | NR_036508 LOC100128398 | uncharacterized LOC100128398 | 14.062195 | -0.837896 | 0.000248232 | 0.006685785 |
| 81578 | NM_001318751COL21A1 | collagen type XXI alpha 1 chain | 14.064025 | 1.775936 | 6.20863E-05 | 0.002241281 |
| 285266 | NR_040100 ENTPD3-AS1 | ENTPD3 antisense RNA 1 | 14.140433 | -1.280901 | 0.000483714 | 0.011242276 |
| 3623 | NM_002191 INHA | inhibin alpha subunit | 14.185040 | 0.349003 | 9.2142E-05 | 0.003079089 |
| 9957 | NM_005114 HS3ST1 | heparan sulfate-glucosamine 3-sulfotransferase 1 | 14.209175 | 2.268536 | 4.75058E-05 | 0.001833316 |
| 397 | NM_001175,N ARHGDIB | Rho GDP dissociation inhibitor beta | 14.243287 | 2.060416 | 4.83431E-05 | 0.001858694 |
| 26471 | NM_001042483NUPR1 | nuclear protein 1, transcriptional regulator | 14.263218 | 4.084046 | 4.00494E-05 | 0.001618013 |
| 7433 | NM_001251882VIPR1 | vasoactive intestinal peptide receptor 1 | 14.326751 | 2.760704 | 4.29507E-05 | 0.001701983 |
| 646324 | NR_037195 LINC00607 | long intergenic non-protein coding RNA 607 | 14.356459 | -2.080652 | 0.000660263 | 0.014271194 |
| 7001 | NM_005809 PRDX2 | peroxiredoxin 2 | 14.417202 | 0.023852 | 0.000101768 | 0.00332978 |
| 100128553 | NM_198495,N CTAGE4 | CTAGE family member 4 | 14.439452 | -2.664732 | 0.002906428 | 0.043497646 |
| 102465666 | NR_106959 MIR7109 | microRNA 7109 | 14.591490 | -3.607418 | 0.012821408 | 0.126955873 |
| 84106 | NM_032152 PRAM1 | PML-RARA regulated adaptor molecule 1 | 14.660377 | -1.495142 | 0.00030226 | 0.007699813 |
| 1124 | NM_001039936CHN2 | chimerin 2 | 14.699233 | 0.480401 | 7.16091E-05 | 0.002464617 |
| 10610 | NM_006456 ST6GALNAC2 | ST6 N-acetylgalactosaminide alpha-2,6-sialyltransferase 2 | 14.742063 | 0.291043 | 7.4998E-05 | 0.002572694 |
| 100131096 | NR_040071 TNRC6C-AS1 | TNRC6C antisense RNA 1 | 14.762212 | 1.532518 | 4.83353E-05 | 0.001858694 |
| 83872 | NM_031935 HMCN1 | hemicentin 1 | 14.814695 | -0.123827 | 0.000189831 | 0.005431061 |
| 101927503 | NM_001323425LOC101927503 | uncharacterized LOC101927503 | 14.871175 | -0.123551 | 9.67853E-05 | 0.003182836 |
| 100874362 | NR_102279 HOXB-AS1 | HOXB cluster antisense RNA 1 | 14.911327 | 2.020242 | 3.76253E-05 | 0.001529038 |
| 3046 | NM_005330 HBE1 | hemoglobin subunit epsilon 1 | 14.942149 | 0.530367 | 0.000119605 | 0.003806187 |
| 123264 | NM_178859 SLC51B | solute carrier family 51 beta subunit | 15.122946 | 2.713454 | 3.2275E-05 | 0.00136029 |
| 388335 | NM_001004313TMEM220 | transmembrane protein 220 | 15.141339 | 2.393218 | 3.33073E-05 | 0.001380686 |
| 552 | NM_000706 AVPR1A | arginine vasopressin receptor 1A | 15.209994 | -0.206245 | 0.000125281 | 0.00394617 |
| 51703 | NM_016234,N ACSL5 | acyl-CoA synthetase long-chain family member 5 | 15.229662 | 3.711907 | 2.87746E-05 | 0.001224698 |
| 81794 | NM_001282352ADAMTS10 | ADAM metallopeptidase with thrombospondin type 1 motif | 15.240596 | 6.036642 | 2.63613E-05 | 0.001138379 |
| 6692 | NM_001032367SPINT1 | serine peptidase inhibitor, Kunitz type 1 | 15.275167 | 2.248605 | 3.3164E-05 | 0.001380052 |
| 2788 | NM_052847 GNG7 | G protein subunit gamma 7 | 15.306805 | -0.317157 | 9.62349E-05 | 0.00317483 |
| 401024 | NM_173651 FSIP2 | fibrous sheath interacting protein 2 | 15.310639 | -0.710115 | 0.000121003 | 0.003838874 |
| 53346 | NM_001144903TM6SF1 | transmembrane 6 superfamily member 1 | 15.313187 | -0.525553 | 0.000137318 | 0.004195589 |
| 710 | NM_000062,N SERPING1 | serpin family G member 1 | 15.344450 | -1.211825 | 0.000478283 | 0.011141412 |
| 54102 | NM_001317009CLIC6 | chloride intracellular channel 6 | 15.344450 | -1.094793 | 0.000478283 | 0.011141412 |
| 284434 | NM_001007525NWD1 | NACHT and WD repeat domain containing 1 | 15.398126 | -2.665028 | 0.00218621 | 0.036130384 |
| 26298 | NM_001206615EHF | ETS homologous factor | 15.452378 | 6.293724 | 2.44135E-05 | 0.001069901 |
| 100534612 | NR_037951 C1QTNF3-AMAC | C1QTNF3-AMACR readthrough (NMD candidate) | 15.496455 | -3.607606 | 0.010215989 | 0.108019342 |
| 9148 | NM_004210 NEURL1 | neuralized E3 ubiquitin protein ligase 1 | 15.539171 | 0.109214 | 6.49069E-05 | 0.00231549 |
| 54933 | NM_001304746RHBDL2 | rhomboid like 2 | 15.567579 | 2.164295 | 3.54092E-05 | 0.001450374 |
| 25787 | NR_024159 DGCR9 | DiGeorge syndrome critical region gene 9 (non-protein cod | 15.591448 | -1.892298 | 0.000442347 | 0.010445154 |
| 440603 | NM_001010922BCL2L15 | BCL2 like 15 | 15.596748 | 3.583043 | 2.65974E-05 | 0.00114618 |
| 929 | NM_000591,N CD14 | CD14 molecule | 15.655329 | 2.893171 | 2.68734E-05 | 0.001155668 |
| 100529262 | NR_037775 MIA-RAB4B | MIA-RAB4B readthrough (NMD candidate) | 15.688710 | -2.250117 | 0.000827393 | 0.017114631 |
| 401331 | NR_024116 RASA4CP | RAS p21 protein activator 4C, pseudogene | 15.713223 | -0.472702 | 0.0001055 | 0.003436637 |
| 7429 | NM_007127 VIL1 | villin 1 | 15.800663 | 4.146827 | 2.26475E-05 | 0.001000988 |

| 112609 | NM_001346541MRAP2 | melanocortin 2 receptor accessory protein 2 | 15.836334 | 2.006548 | 2.7912E-05 | 0.001195363 |
| --- | --- | --- | --- | --- | --- | --- |
| 9619 | NM_004915,N ABCG1 | ATP binding cassette subfamily G member 1 | 15.853231 | 4.091656 | 2.30661E-05 | 0.001017316 |
| 1909 | NM_001166055EDNRA | endothelin receptor type A | 15.877429 | 1.458759 | 3.40174E-05 | 0.001404489 |
| 285972 | NR_034033 LINC00996 | long intergenic non-protein coding RNA 996 | 15.877462 | -2.893828 | 0.001904897 | 0.032297378 |
| 103352539 | NR_121647 LINC01410 | long intergenic non-protein coding RNA 1410 | 15.877462 | -2.181370 | 0.001904897 | 0.032297378 |
| 113220 | NM_138424 KIF12 | kinesin family member 12 | 15.923380 | 3.748919 | 2.22908E-05 | 0.000995387 |
| 146754 | NM_001303270DNAH2 | dynein axonemal heavy chain 2 | 15.939131 | 0.414605 | 5.10387E-05 | 0.001944263 |
| 386593 | NR_027928 CHKB-CPT1B | CHKB-CPT1B readthrough (NMD candidate) | 15.970736 | 3.593762 | 2.19599E-05 | 0.000991789 |
| 728780 | NM_001276713ANKDD1B | ankyrin repeat and death domain containing 1B | 15.991266 | -1.456053 | 0.000194262 | 0.005550158 |
| 340547 | NM_001170553VSIG1 | V-set and immunoglobulin domain containing 1 | 16.022750 | 1.210315 | 3.26591E-05 | 0.001363127 |
| 653190 | NR_003569 ABCC6P1 | ATP binding cassette subfamily C member 6 pseudogene 1 | 16.025524 | -0.013825 | 6.49255E-05 | 0.00231549 |
| 7903 | NM_005668,N ST8SIA4 | ST8 alpha-N-acetyl-neuraminide alpha-2,8-sialyltransferase | 16.067991 | 4.962113 | 2.01248E-05 | 0.000914903 |
| 79849 | NM_001168468PDZD3 | PDZ domain containing 3 | 16.160356 | 1.008054 | 3.32253E-05 | 0.001380052 |
| 5136 | NM_001003683PDE1A | phosphodiesterase 1A | 16.226490 | 0.744295 | 3.91743E-05 | 0.001588864 |
| 57822 | NM_001195010GRHL3 | grainyhead like transcription factor 3 | 16.270176 | 2.311753 | 2.24204E-05 | 0.000995387 |
| 2128 | NM_001304519EVX1 | even-skipped homeobox 1 | 16.356799 | -2.606103 | 0.001664638 | 0.029205285 |
| 5314 | NM_138694,N PKHD1 | PKHD1, fibrocystin/polyductin | 16.356799 | -2.635412 | 0.001664638 | 0.029205285 |
| 124056 | NM_001267721NOXO1 | NADPH oxidase organizer 1 | 16.356799 | -2.695867 | 0.001664638 | 0.029205285 |
| 284100 | NR_024178 YWHAEP7 | tyrosine 3-monooxygenase/tryptophan 5-monooxygenase a | 16.356799 | -2.606103 | 0.001664638 | 0.029205285 |
| 100142659 | NM_001278507CTAGE8 | CTAGE family member 8 | 16.356799 | -2.606103 | 0.001664638 | 0.029205285 |
| 377841 | NM_001033113ENTPD8 | ectonucleoside triphosphate diphosphohydrolase 8 | 16.502770 | 0.118178 | 4.92704E-05 | 0.001890832 |
| 345275 | NM_001136230HSD17B13 | hydroxysteroid 17-beta dehydrogenase 13 | 16.653277 | -0.765574 | 8.82056E-05 | 0.002957104 |
| 64063 | NM_022119 PRSS22 | protease, serine 22 | 16.656710 | -1.318983 | 0.000157475 | 0.004692055 |
| 246176 | NM_139285 GAS2L2 | growth arrest specific 2 like 2 | 16.682550 | -0.528543 | 6.67247E-05 | 0.00236741 |
| 54866 | NM_001130143PPP1R14D | protein phosphatase 1 regulatory inhibitor subunit 14D | 16.725019 | -1.127776 | 0.000208764 | 0.005819792 |
| 57758 | NM_001170690SCUBE2 | signal peptide, CUB domain and EGF like domain containin | 16.750589 | -0.838057 | 0.000120773 | 0.003837461 |
| 6865 | NM_001057 TACR2 | tachykinin receptor 2 | 16.836136 | -2.057189 | 0.001458763 | 0.026376329 |
| 722 | NM_000715 C4BPA | complement component 4 binding protein alpha | 16.959542 | 2.477430 | 1.75016E-05 | 0.000809889 |
| 6414 | NM_001085486SELENOP | selenoprotein P | 17.140429 | 2.065228 | 1.72732E-05 | 0.000801112 |
| 240 | NM_000698,N ALOX5 | arachidonate 5-lipoxygenase | 17.164748 | 2.239619 | 2.25676E-05 | 0.000999596 |
| 350 | NM_000042 APOH | apolipoprotein H | 17.192764 | 5.048396 | 1.3765E-05 | 0.000659634 |
| 101927167 | NR_125398 GATA2-AS1 | GATA2 antisense RNA 1 | 17.197612 | -0.058372 | 8.69192E-05 | 0.002923455 |
| 201501 | NM_001039360ZBTB7C | zinc finger and BTB domain containing 7C | 17.239866 | -0.037194 | 5.09709E-05 | 0.001944263 |
| 730668 | NR_027240 LOC730668 | dynein heavy chain -like pseudogene | 17.315473 | -2.416282 | 0.001281795 | 0.023907955 |
| 3355 | NM_000866,N HTR1F | 5-hydroxytryptamine receptor 1F | 17.320433 | -1.327813 | 0.000262647 | 0.006965205 |
| 8515 | NM_001303040ITGA10 | integrin subunit alpha 10 | 17.530304 | 3.685419 | 1.3028E-05 | 0.000634079 |
| 3822 | NM_002260 KLRC2 | killer cell lectin like receptor C2 | 17.754028 | 3.397223 | 1.3383E-05 | 0.000645286 |
| 101928290 | NR_120465 PLBD1-AS1 | PLBD1 antisense RNA 1 | 17.794810 | -2.248345 | 0.00112921 | 0.021547706 |
| 146212 | NM_001100915KCTD19 | potassium channel tetramerization domain containing 19 | 17.889326 | 1.546750 | 1.83356E-05 | 0.000842827 |
| 66002 | NM_023944,NRCYP4F12 | cytochrome P450 family 4 subfamily F member 12 | 17.951852 | 3.525386 | 1.14562E-05 | 0.000572394 |
| 3934 | NM_005564 LCN2 | lipocalin 2 | 17.953708 | 6.951763 | 1.10184E-05 | 0.000554537 |
| 56477 | NM_001301873CCL28 | C-C motif chemokine ligand 28 | 18.061427 | -0.680687 | 0.000212712 | 0.005905979 |
| 3965 | NM_001330163LGALS9 | galectin 9 | 18.156890 | 2.489789 | 1.20469E-05 | 0.000595419 |

| 6337 | NM_001038,N | SCNN1A | sodium channel epithelial 1 alpha subunit | 18.197760 | 5.264373 | 9.97931E-06 | 0.000512213 |
| --- | --- | --- | --- | --- | --- | --- | --- |
| 102466983 | NR_106824 | MIR6766 | microRNA 6766 | 18.211351 | -3.296525 | 0.005404101 | 0.068327526 |
| 109623460 | NR_145802 | SNORD138 | small nucleolar RNA, C/D box 138 | 18.211351 | -3.205929 | 0.005404101 | 0.068327526 |
| 2277 | NM_004469 | VEGFD | vascular endothelial growth factor D | 18.274146 | -2.522135 | 0.000997265 | 0.019854122 |
| 497259 | NR_049896 | C18orf61 | uncharacterized LOC497259 | 18.308425 | -1.402048 | 0.000198579 | 0.005642325 |
| 1991 | NM_001972 | ELANE | elastase, neutrophil expressed | 18.320321 | -0.916445 | 9.58805E-05 | 0.003168192 |
| 84460 | NM_001011657ZMAT1 | | zinc finger matrin-type 1 | 18.627551 | 1.555059 | 1.40682E-05 | 0.000672056 |
| 4102 | NM_005362 MAGEA3 | | MAGE family member A3 | 18.764687 | 4.890295 | 8.82903E-06 | 0.000461183 |
| 3082 | NM_000601,N HGF | | hepatocyte growth factor | 18.807722 | 1.928852 | 1.05015E-05 | 0.000532409 |
| 3960 | NM_006149 LGALS4 | | galectin 4 | 18.866757 | 3.273568 | 9.03825E-06 | 0.000469739 |
| 312 | NM_001003954ANXA13 | | annexin A13 | 18.890588 | 4.878292 | 8.13233E-06 | 0.000435601 |
| 3483 | NM_001146006IGFALS | | insulin like growth factor binding protein acid labile subuni | 18.911976 | -1.379150 | 0.000110866 | 0.003555439 |
| 100132103 | NR_027424 FAM66E | | family with sequence similarity 66 member E | 18.948782 | -1.662515 | 0.000301013 | 0.007686975 |
| 120224 | NM_001331210TMEM45B | | transmembrane protein 45B | 19.008696 | 0.458042 | 2.36078E-05 | 0.001036789 |
| 105371919 | NR_146504 LINC01977 | | long intergenic non-protein coding RNA 1977 | 19.064152 | -0.237157 | 3.24813E-05 | 0.00136029 |
| 8626 | NM_001114978TP63 | | tumor protein p63 | 19.355968 | 3.575917 | 7.48883E-06 | 0.000409805 |
| 11254 | NM_007231 SLC6A14 | | solute carrier family 6 member 14 | 19.440936 | 3.951805 | 1.14346E-05 | 0.000572394 |
| 54 | NM_001111034ACP5 | | acid phosphatase 5, tartrate resistant | 19.484849 | -0.565796 | 6.92469E-05 | 0.002430804 |
| 349196 | NR_027000,NR LINC00965 | | long intergenic non-protein coding RNA 965 | 19.712157 | -2.495433 | 0.000696625 | 0.014824777 |
| 105371328 | NR_135173 LOC105371328 | | uncharacterized LOC105371328 | 19.712157 | -2.416967 | 0.000696625 | 0.014824777 |
| 100124700 | NR_003716,NR HOTAIR | | HOX transcript antisense RNA | 19.764298 | 0.527712 | 1.7107E-05 | 0.000795186 |
| 10628 | NM_001313972TXNIP | | thioredoxin interacting protein | 19.774711 | 5.306231 | 6.20807E-06 | 0.000350858 |
| 100526664 | NM_001198759LY75-CD302 | | LY75-CD302 readthrough | 19.888552 | -0.355136 | 2.57778E-05 | 0.001115511 |
| 3171 | NM_004497 FOXA3 | | forkhead box A3 | 19.921210 | 3.005358 | 7.01545E-06 | 0.000385943 |
| 286133 | NM_173833 SCARA5 | | scavenger receptor class A member 5 | 19.994600 | 6.368939 | 5.6657E-06 | 0.000322851 |
| 677799 | NR_002953 SNORA11 | | small nucleolar RNA, H/ACA box 11 | 20.021281 | -3.163008 | 0.00365261 | 0.051642736 |
| 54854 | NM_017708 FAM83E | | family with sequence similarity 83 member E | 20.119038 | 1.486872 | 8.9844E-06 | 0.000468116 |
| 79730 | NM_001330648NSUN7 | | NOP2/Sun RNA methyltransferase family member 7 | 20.299877 | 1.183948 | 1.47475E-05 | 0.000696468 |
| 84766 | NM_001144958CRACR2A | | calcium release activated channel regulator 2A | 20.403793 | 3.042503 | 6.24205E-06 | 0.000351817 |
| 158435 | NR_033838 LOC158435 | | uncharacterized LOC158435 | 20.531405 | -1.711523 | 0.000110389 | 0.003551167 |
| 399948 | NM_001302644COLCA1 | | colorectal cancer associated 1 | 20.577505 | 0.864914 | 1.04066E-05 | 0.000530748 |
| 101929882 | NR_135558 LOC101929882 | | uncharacterized LOC101929882 | 20.578818 | -1.927864 | 0.000199564 | 0.005662538 |
| 195814 | NM_001318049SDR16C5 | | short chain dehydrogenase/reductase family 16C member 5 | 20.695430 | 1.819256 | 7.59342E-06 | 0.000413342 |
| 729723 | NR_034113 DNAJC27-AS1 | | DNAJC27 antisense RNA 1 | 20.701305 | -1.171846 | 6.89926E-05 | 0.002430804 |
| 102467147 | NR_104664 LINC01948 | | long intergenic non-protein coding RNA 1948 | 20.904826 | -1.315092 | 0.000184391 | 0.005297386 |
| 111 | NM_001199642ADCY5 | | adenylate cyclase 5 | 21.110677 | 3.296583 | 4.80536E-06 | 0.000281099 |
| 113278 | NM_033409 SLC52A3 | | solute carrier family 52 member 3 | 21.393713 | 2.798586 | 4.98281E-06 | 0.00028871 |
| 100507308 | NR_109982 TARID | | TCF21 antisense RNA inducing promoter demethylation | 21.408017 | 2.829369 | 4.68345E-06 | 0.000276792 |
| 123099 | NM_206918 DEGS2 | | delta 4-desaturase, sphingolipid 2 | 21.457719 | 1.073022 | 7.67953E-06 | 0.000416361 |
| 101927318 | NR_110589,NR LOC101927318 | | uncharacterized LOC101927318 | 21.519397 | -1.650708 | 8.64573E-05 | 0.002912655 |
| 100422943 | NR_036156 MIR3189 | | microRNA 3189 | 21.526219 | 0.013645 | 1.5124E-05 | 0.000712618 |
| 5973 | NM_002910 RENBP | | renin binding protein | 21.556840 | -1.633387 | 0.000157876 | 0.004692055 |
| 388633 | NM_001010978LDLRAD1 | | low density lipoprotein receptor class A domain containing | 21.587196 | -0.711070 | 2.47175E-05 | 0.001080934 |

| 101927565 | NR_110135 LINC01267 | long intergenic non-protein coding RNA 1267 | 21.629504 | -2.469361 | 0.000444759 | 0.010490116 |
| --- | --- | --- | --- | --- | --- | --- |
| 948 | NM_000072,N CD36 | CD36 molecule | 21.838029 | -0.190631 | 2.32084E-05 | 0.001021419 |
| 84229 | NM_001289162DRC7 | dynein regulatory complex subunit 7 | 21.894191 | -1.129875 | 5.12421E-05 | 0.001948426 |
| 1577 | NM_000777,N CYP3A5 | cytochrome P450 family 3 subfamily A member 5 | 21.904175 | 5.421497 | 3.47813E-06 | 0.000215404 |
| 55282 | NM_001161575LRRC36 | leucine rich repeat containing 36 | 21.960456 | 0.646231 | 8.57773E-06 | 0.000450331 |
| 10791 | NM_006634 VAMP5 | vesicle associated membrane protein 5 | 22.146627 | -0.864886 | 3.48692E-05 | 0.001433933 |
| 57125 | NM_020405 PLXDC1 | plexin domain containing 1 | 22.146627 | -0.484089 | 3.48692E-05 | 0.001433933 |
| 81693 | NM_030943 AMN | amnion associated transmembrane protein | 22.204160 | 0.536332 | 8.80312E-06 | 0.000460994 |
| 8399 | NM_003561,NRPLA2G10 | phospholipase A2 group X | 22.208854 | -1.983975 | 0.000135681 | 0.004151707 |
| 346389 | NM_182762 MACC1 | MACC1, MET transcriptional regulator | 22.736177 | -2.757164 | 0.002116575 | 0.035307551 |
| 90246 | NR_026954 LOC90246 | uncharacterized LOC90246 | 22.754386 | -1.170635 | 6.452E-05 | 0.00230899 |
| 6338 | NM_000336 SCNN1B | sodium channel epithelial 1 beta subunit | 23.060374 | -0.232448 | 1.2773E-05 | 0.000626089 |
| 1299 | NM_001853 COL9A3 | collagen type IX alpha 3 chain | 23.277754 | -0.000449 | 1.08012E-05 | 0.000544934 |
| 677814 | NR_002967 SNORA31 | small nucleolar RNA, H/ACA box 31 | 23.546851 | -2.272760 | 0.000292548 | 0.007526564 |
| 102724354 | NR_136540 LINC01669 | long intergenic non-protein coding RNA 1669 | 23.546851 | -2.343594 | 0.000292548 | 0.007526564 |
| 168002 | NM_001286350DACT2 | dishevelled binding antagonist of beta catenin 2 | 23.620024 | 2.687529 | 2.70734E-06 | 0.000175553 |
| 100128979 | NR_147233 LOC100128979 | uncharacterized LOC100128979 | 23.742378 | -1.468706 | 5.15353E-05 | 0.001955979 |
| 374900 | NM_001204835ZNF568 | zinc finger protein 568 | 23.963558 | 0.680819 | 8.14974E-06 | 0.000435601 |
| 10178 | NM_001163278TENM1 | teneurin transmembrane protein 1 | 24.123978 | 2.324218 | 3.34899E-06 | 0.00021053 |
| 283460 | NR_024345 HNF1A-AS1 | HNF1A antisense RNA 1 | 24.281297 | 1.919457 | 2.92734E-06 | 0.000187468 |
| 2346 | NM_001014986FOLH1 | folate hydrolase 1 | 24.483372 | -1.271511 | 4.37661E-05 | 0.001727675 |
| 10203 | NM_001271751CALCRL | calcitonin receptor like receptor | 24.552262 | 1.778373 | 3.10569E-06 | 0.000196277 |
| 150244 | NR_003950 ZDHHC8P1 | zinc finger DHHC-type containing 8 pseudogene 1 | 24.859561 | 3.114050 | 1.93379E-06 | 0.00013072 |
| 84174 | NM_032214,N SLA2 | Src like adaptor 2 | 24.977367 | -1.456015 | 3.93398E-05 | 0.001592452 |
| 124220 | NM_145252 ZG16B | zymogen granule protein 16B | 25.091560 | 1.081377 | 3.70817E-06 | 0.000225599 |
| 2267 | NM_004467,N FGL1 | fibrinogen like 1 | 25.179847 | 6.644453 | 1.48667E-06 | 0.000108976 |
| 390226 | NR_024042 GUCY2EP | guanylate cyclase 2E, pseudogene | 25.188168 | 0.369519 | 5.24344E-06 | 0.000302119 |
| 100505746 | NR_038311,NR ITGB2-AS1 | ITGB2 antisense RNA 1 | 25.298958 | 2.266757 | 2.2006E-06 | 0.000145429 |
| 94025 | NM_024690 MUC16 | mucin 16, cell surface associated | 25.438097 | 4.430133 | 1.52327E-06 | 0.000109787 |
| 25928 | NM_015464 SOSTDC1 | sclerostin domain containing 1 | 25.464198 | -2.273281 | 0.000197524 | 0.005627799 |
| 158038 | NM_001258282LINGO2 | leucine rich repeat and Ig domain containing 2 | 25.476155 | 0.383389 | 6.3824E-06 | 0.000356811 |
| 79949 | NM_001193434PLEKHS1 | pleckstrin homology domain containing S1 | 25.718361 | -1.283531 | 3.36368E-05 | 0.001391553 |
| 27198 | NM_032554 HCAR1 | hydroxycarboxylic acid receptor 1 | 26.120941 | -1.605447 | 5.86005E-05 | 0.002164554 |
| 57523 | NM_025081 NYNRIN | NYN domain and retroviral integrase containing | 26.344235 | 3.167638 | 1.40439E-06 | 0.000104121 |
| 100507642 | NR_108064,NR LOC100507642 | uncharacterized LOC100507642 | 26.353029 | -0.276890 | 8.22341E-06 | 0.000438405 |
| 8972 | NM_004668 MGAM | maltase-glucoamylase | 26.406757 | 3.306604 | 1.35301E-06 | 0.000101036 |
| 643338 | NM_001130448C15orf62 | chromosome 15 open reading frame 62 | 27.381545 | -1.178098 | 0.000124843 | 0.003942562 |
| 9427 | NM_001290787ECEL1 | endothelin converting enzyme like 1 | 28.057435 | -0.716356 | 1.33394E-05 | 0.000645286 |
| 100128537 | NR_135298,NR C1orf132 | chromosome 1 open reading frame 132 | 28.092670 | 0.095867 | 4.1346E-06 | 0.000247896 |
| 3227 | NM_014212 HOXC11 | homeobox C11 | 28.132557 | 0.701038 | 3.64702E-06 | 0.000224519 |
| 101926935 | NR_110001 LOC101926935 | uncharacterized LOC101926935 | 28.132557 | 0.525715 | 3.64702E-06 | 0.000224519 |
| 115019 | NM_052934,N SLC26A9 | solute carrier family 26 member 9 | 28.278500 | 2.094043 | 1.25308E-06 | 9.50085E-05 |
| 92973 | NR_024006 LINC00950 | long intergenic non-protein coding RNA 950 | 28.286433 | 1.373182 | 1.59612E-06 | 0.000113847 |

| 23263 | NM_001112732MCF2L | MCF.2 cell line derived transforming sequence like | 28.287318 | 2.913707 | 9.67786E-07 | 7.64071E-05 |
| --- | --- | --- | --- | --- | --- | --- |
| 388536 | NM_001242800ZNF790 | zinc finger protein 790 | 28.340219 | -0.841624 | 0.000104849 | 0.003420819 |
| 6755 | NM_001053,N SSTR5 | somatostatin receptor 5 | 28.400987 | 0.835627 | 2.05457E-06 | 0.000137003 |
| 407012 | NR_029496 MIR24-1 | microRNA 24-1 | 28.468350 | -0.619637 | 8.49075E-06 | 0.000449028 |
| 4776 | NM_001136022NFATC4 | nuclear factor of activated T-cells 4 | 28.587907 | 2.913366 | 9.00223E-07 | 7.18962E-05 |
| 51365 | NM_001206960PLA1A | phospholipase A1 member A | 28.915041 | 1.503665 | 1.53142E-06 | 0.000109991 |
| 55553 | NM_001145811SOX6 | SRY-box 6 | 29.075697 | 1.030926 | 1.79568E-06 | 0.000123812 |
| 29993 | NM_001199583PACSIN1 | protein kinase C and casein kinase substrate in neurons 1 | 29.205773 | 0.751262 | 1.96014E-06 | 0.00013207 |
| 23676 | NM_014332,NRSMPX | small muscle protein, X-linked | 29.423329 | -1.140887 | 1.6221E-05 | 0.000757405 |
| 401474 | NM_001101676SAMD12 | sterile alpha motif domain containing 12 | 29.701459 | 3.140000 | 7.16165E-07 | 5.99752E-05 |
| 284353 | NM_198478 NKPD1 | NTPase KAP family P-loop domain containing 1 | 30.027361 | 0.868420 | 1.67136E-06 | 0.00011741 |
| 81793 | NM_001017388TLR10 | toll like receptor 10 | 30.443207 | -0.748949 | 8.48705E-06 | 0.000449028 |
| 11174 | NM_197941,NRADAMTS6 | ADAM metallopeptidase with thrombospondin type 1 motif | 31.026947 | 2.882448 | 5.6461E-07 | 4.86512E-05 |
| 100130872 | NR_024569 LOC100130872 | uncharacterized LOC100130872 | 31.216239 | -1.909737 | 6.38319E-05 | 0.002296283 |
| 64150 | NR_002770 DIO3OS | DIO3 opposite strand/antisense RNA (head to head) | 31.320646 | 0.746500 | 1.49094E-06 | 0.000108976 |
| 120376 | NM_001136105COLCA2 | colorectal cancer associated 2 | 31.439356 | 3.024727 | 5.14487E-07 | 4.52858E-05 |
| 10351 | NM_001288985ABCA8 | ATP binding cassette subfamily A member 8 | 31.465790 | 1.867771 | 7.84961E-07 | 6.36742E-05 |
| 727897 | NM_002458 MUC5B | mucin 5B, oligomeric mucus/gel-forming | 31.653093 | 10.839707 | 3.84516E-07 | 3.47324E-05 |
| 55365 | NM_018487 TMEM176A | transmembrane protein 176A | 31.695576 | -2.041160 | 5.89816E-05 | 0.002174751 |
| 127435 | NM_001199080PODN | podocan | 32.174913 | -2.021777 | 5.45535E-05 | 0.002027118 |
| 85477 | NM_001112706SCIN | scinderin | 32.219152 | 0.306541 | 1.89663E-06 | 0.00012953 |
| 340554 | NM_001010888ZC3H12B | zinc finger CCCH-type containing 12B | 32.387304 | 0.075686 | 9.56737E-06 | 0.000492291 |
| 1469 | NM_001898 CST1 | cystatin SN | 32.602155 | 6.169240 | 3.3331E-07 | 3.09171E-05 |
| 79667 | NR_026804 KLF3-AS1 | KLF3 antisense RNA 1 | 32.620311 | 0.479001 | 1.39252E-06 | 0.000103613 |
| 58473 | NM_001130033PLEKHB1 | pleckstrin homology domain containing B1 | 33.063625 | 2.210057 | 5.46935E-07 | 4.73362E-05 |
| 3760 | NM_001260508KCNJ3 | potassium voltage-gated channel subfamily J member 3 | 33.128297 | -1.080163 | 8.44048E-06 | 0.000448821 |
| 170392 | NM_152635,NROIT3 | oncoprotein induced transcript 3 | 33.133587 | -1.618671 | 4.6803E-05 | 0.001818542 |
| 444882 | NM_001002923IGFL4 | IGF like family member 4 | 33.293100 | -0.475151 | 1.60178E-05 | 0.00074961 |
| 3851 | NM_002272 KRT4 | keratin 4 | 33.433638 | 2.017566 | 4.9946E-07 | 4.4151E-05 |
| 51313 | NM_001031700FAM198B | family with sequence similarity 198 member B | 33.558956 | 4.789264 | 2.99136E-07 | 2.79983E-05 |
| 338440 | NM_001012302ANO9 | anoctamin 9 | 33.825340 | 3.034886 | 3.42122E-07 | 3.15928E-05 |
| 4935 | NM_000273 GPR143 | G protein-coupled receptor 143 | 34.271121 | -0.515836 | 1.36798E-05 | 0.000658063 |
| 102724562 | NR_135824 LOC102724562 | macrophage stimulating 1 pseudogene | 34.505113 | -0.001020 | 1.75282E-06 | 0.000121948 |
| 1084 | NM_001277163CEACAM3 | carcinoembryonic antigen related cell adhesion molecule 3 | 34.597128 | -1.404736 | 1.29903E-05 | 0.000634079 |
| 101669762 | NR_103783 BLACAT1 | bladder cancer associated transcript 1 (non-protein coding) | 34.923136 | -0.828084 | 1.23407E-05 | 0.000606335 |
| 23145 | NM_198455 SSPO | SCO-spondin | 35.266782 | 3.544121 | 2.48758E-07 | 2.46199E-05 |
| 100505989 | NR_038834 LINC01207 | long intergenic non-protein coding RNA 1207 | 35.275427 | 1.825393 | 4.08205E-07 | 3.65529E-05 |
| 106614088 | NR_132742 MIR34AHG | MIR34A host gene | 35.335173 | 4.028054 | 2.44601E-07 | 2.44424E-05 |
| 103344930 | NR_121649 LINC01391 | long intergenic non-protein coding RNA 1391 | 35.530271 | -1.694472 | 3.24104E-05 | 0.00136029 |
| 23732 | NM_014334 FRRS1L | ferric chelate reductase 1 like | 35.633863 | 1.352718 | 1.45815E-06 | 0.000107338 |
| 2615 | NM_001128922LRRC32 | leucine rich repeat containing 32 | 35.647846 | 0.796408 | 7.63242E-07 | 6.24018E-05 |
| 326624 | NM_001006638RAB37 | RAB37, member RAS oncogene family | 36.005022 | 5.083737 | 1.95484E-07 | 2.02179E-05 |
| 8857 | NM_003890 FCGBP | Fc fragment of IgG binding protein | 36.756967 | 7.513748 | 1.58834E-07 | 1.73101E-05 |

| 101929829 | NR_111920 | LOC101929829 | cytochrome P450 family 2 subfamily D member 6 pseudog | 36.805265 | -0.635125 | 2.83166E-06 | 0.00018304 |
| --- | --- | --- | --- | --- | --- | --- | --- |
| 4583 | NM_002457 | MUC2 | mucin 2, oligomeric mucus/gel-forming | 37.722090 | 2.262892 | 2.48243E-07 | 2.46199E-05 |
| 27134 | NM_001267560TJP3 | | tight junction protein 3 | 37.889522 | 4.173034 | 1.6001E-07 | 1.73288E-05 |
| 59352 | NM_001017403LGR6 | | leucine rich repeat containing G protein-coupled receptor 6 | 38.279220 | 2.787829 | 2.13225E-07 | 2.18344E-05 |
| 3109 | NM_002118 HLA-DMB | | major histocompatibility complex, class II, DM beta | 38.446412 | 4.518850 | 1.3426E-07 | 1.54443E-05 |
| 7018 | NM_001063 TF | | transferrin | 38.992223 | -0.556285 | 2.04199E-06 | 0.000137003 |
| 8631 | NM_001075099SKAP1 | | src kinase associated phosphoprotein 1 | 39.161229 | -0.476190 | 6.56424E-06 | 0.000365003 |
| 102724594 | NM_001320646U2AF1L5 | | U2 small nuclear RNA auxiliary factor 1 like 5 | 39.327032 | 4.051753 | 1.34396E-07 | 1.54443E-05 |
| 347 | NM_001647 APOD | | apolipoprotein D | 39.626102 | 1.077920 | 3.4797E-07 | 3.18528E-05 |
| 64284 | NM_022449,NRRAB17 | | RAB17, member RAS oncogene family | 39.836548 | 1.988195 | 2.19637E-07 | 2.22862E-05 |
| 10103 | NM_005727 TSPAN1 | | tetraspanin 1 | 41.445730 | 3.994947 | 9.38404E-08 | 1.15541E-05 |
| 285419 | NR_027105,NR LINC01091 | | long intergenic non-protein coding RNA 1091 | 42.421301 | -0.787780 | 4.20365E-06 | 0.000251308 |
| 338809 | NM_001037671C12orf74 | | chromosome 12 open reading frame 74 | 42.720322 | -1.454381 | 1.20609E-05 | 0.000595419 |
| 7365 | NM_001075,N UGT2B10 | | UDP glucuronosyltransferase family 2 member B10 | 42.892679 | 1.610586 | 1.61276E-07 | 1.7375E-05 |
| 1757 | NM_001134707SARDH | | sarcosine dehydrogenase | 44.637669 | -1.191960 | 9.49475E-06 | 0.000489773 |
| 2939 | NM_000846 GSTA2 | | glutathione S-transferase alpha 2 | 44.757838 | -0.366987 | 9.28552E-07 | 7.35904E-05 |
| 27071 | NM_001306151DAPP1 | | dual adaptor of phosphotyrosine and 3-phosphoinositides | 45.029359 | 0.294528 | 3.00695E-06 | 0.000191443 |
| 10826 | NM_032385 FAXDC2 | | fatty acid hydroxylase domain containing 2 | 46.664641 | 2.626751 | 6.86357E-08 | 8.87331E-06 |
| 10205 | NM_005797,N MPZL2 | | myelin protein zero like 2 | 46.768074 | 0.569594 | 4.94008E-07 | 4.38565E-05 |
| 54474 | NM_019010 KRT20 | | keratin 20 | 46.899273 | 1.639579 | 1.07672E-07 | 1.26545E-05 |
| 389903 | NM_001129826CSAG3 | | CSAG family member 3 | 47.093767 | 1.311894 | 1.80504E-07 | 1.89529E-05 |
| 284076 | NM_001130918TTLL6 | | tubulin tyrosine ligase like 6 | 47.853520 | 4.981547 | 3.59623E-08 | 5.20196E-06 |
| 116844 | NM_052972 LRG1 | | leucine rich alpha-2-glycoprotein 1 | 48.786191 | 0.317640 | 2.93829E-07 | 2.76266E-05 |
| 440905 | NR_026758 FAR2P1 | | fatty acyl-CoA reductase 2 pseudogene 1 | 49.053446 | 0.728057 | 2.25221E-07 | 2.27253E-05 |
| 6398 | NM_003004 SECTM1 | | secreted and transmembrane 1 | 49.358255 | 1.081714 | 2.74492E-07 | 2.63738E-05 |
| 100507387 | NR_038402 LOC100507387 | | uncharacterized LOC100507387 | 49.910374 | -1.181987 | 5.13665E-06 | 0.000296792 |
| 255631 | NM_001349955COL24A1 | | collagen type XXIV alpha 1 chain | 49.919467 | -0.892112 | 1.67755E-06 | 0.00011741 |
| 338817 | NR_033890 LINC01252 | | long intergenic non-protein coding RNA 1252 | 50.073335 | 0.459962 | 2.52361E-07 | 2.48576E-05 |
| 4320 | NM_005940,NRMMP11 | | matrix metallopeptidase 11 | 50.389711 | -0.906302 | 4.87164E-06 | 0.000283061 |
| 339778 | NM_001105519C2orf70 | | chromosome 2 open reading frame 70 | 51.348384 | -0.488662 | 4.38774E-06 | 0.000261557 |
| 440823 | NR_003491,NR MIAT | | myocardial infarction associated transcript (non-protein cod | 53.139370 | 1.210568 | 8.85946E-08 | 1.09735E-05 |
| 25840 | NM_014033 METTL7A | | methyltransferase like 7A | 53.871272 | 1.861246 | 4.70462E-08 | 6.4447E-06 |
| 163589 | NM_001199085TDRD5 | | tudor domain containing 5 | 54.399145 | 1.181275 | 1.01377E-07 | 1.21917E-05 |
| 27283 | NM_014464 TINAG | | tubulointerstitial nephritis antigen | 54.499740 | 0.092153 | 2.90566E-07 | 2.75572E-05 |
| 5288 | NM_001288772PIK3C2G | | phosphatidylinositol-4-phosphate 3-kinase catalytic subunit | 54.703742 | -1.260843 | 3.0828E-06 | 0.000195607 |
| 6376 | NM_001304392CX3CL1 | | C-X3-C motif chemokine ligand 1 | 55.921863 | 4.511088 | 1.65682E-08 | 2.80912E-06 |
| 4648 | NM_001080527MYO7B | | myosin VIIB | 56.801743 | 2.945889 | 1.97616E-08 | 3.2442E-06 |
| 375307 | NM_001320865CATIP | | ciliogenesis associated TTC17 interacting protein | 57.124854 | -1.772416 | 2.11108E-05 | 0.000957625 |
| 5104 | NM_000624 SERPINA5 | | serpin family A member 5 | 57.415185 | 0.673531 | 1.49401E-07 | 1.68266E-05 |
| 2785 | NM_012202 GNG3 | | G protein subunit gamma 3 | 57.536458 | -4.251067 | 0.048219086 | 0.298715722 |
| 5225 | NM_001166424PGC | | progastricsin | 57.536458 | -4.551402 | 0.048219086 | 0.298715722 |
| 25812 | NR_024591 POM121L1P | | POM121 transmembrane nucleoporin like 1, pseudogene | 57.536458 | -4.344428 | 0.048219086 | 0.298715722 |
| 51052 | NM_015893 PRLH | | prolactin releasing hormone | 57.536458 | -4.551402 | 0.048219086 | 0.298715722 |

| 54769 | NM_017594 | DIRAS2 | DIRAS family GTPase 2 | 57.536458 | -4.163360 | 0.048219086 | 0.298715722 |
| --- | --- | --- | --- | --- | --- | --- | --- |
| 84366 | NM_032391 | PRAC1 | PRAC1 small nuclear protein | 57.536458 | -4.551402 | 0.048219086 | 0.298715722 |
| 138255 | NM_001010940C9orf135 | | chromosome 9 open reading frame 135 | 57.536458 | -4.551402 | 0.048219086 | 0.298715722 |
| 149563 | NM_178840 C1orf64 | | chromosome 1 open reading frame 64 | 57.536458 | -4.551402 | 0.048219086 | 0.298715722 |
| 166979 | NM_001145734CDC20B | | cell division cycle 20B | 57.536458 | -4.163360 | 0.048219086 | 0.298715722 |
| 339398 | NM_001004432LINGO4 | | leucine rich repeat and Ig domain containing 4 | 57.536458 | -4.551402 | 0.048219086 | 0.298715722 |
| 387804 | NM_001144871VSTM5 | | V-set and transmembrane domain containing 5 | 57.536458 | -4.551402 | 0.048219086 | 0.298715722 |
| 392862 | NM_001145118GRID2IP | | Grid2 interacting protein | 57.536458 | -4.163360 | 0.048219086 | 0.298715722 |
| 440533 | NM_001130167PSG8 | | pregnancy specific beta-1-glycoprotein 8 | 57.536458 | -4.551402 | 0.048219086 | 0.298715722 |
| 552860 | NR_038210 SAMD12-AS1 | | SAMD12 antisense RNA 1 | 57.536458 | -4.551402 | 0.048219086 | 0.298715722 |
| 641776 | NR_146072,NR SPDYE14P | | speedy/RINGO cell cycle regulator family member E14, pse | 57.536458 | -4.551402 | 0.048219086 | 0.298715722 |
| 643224 | NR_027156 TUBBP5 | | tubulin beta pseudogene 5 | 57.536458 | -3.725915 | 0.048219086 | 0.298715722 |
| 100009667 | NR_131184 POU5F1P5 | | POU class 5 homeobox 1 pseudogene 5 | 57.536458 | -4.551402 | 0.048219086 | 0.298715722 |
| 100240735 | NR_026658 LOC100240735 | | uncharacterized LOC100240735 | 57.536458 | -3.857586 | 0.048219086 | 0.298715722 |
| 100506725 | NR_108082,NR LOC100506725 | | uncharacterized LOC100506725 | 57.536458 | -4.551402 | 0.048219086 | 0.298715722 |
| 100507254 | NR_038981,NR LINC01013 | | long intergenic non-protein coding RNA 1013 | 57.536458 | -4.551402 | 0.048219086 | 0.298715722 |
| 100820709 | NR_047465 LINC00501 | | long intergenic non-protein coding RNA 501 | 57.536458 | -4.551402 | 0.048219086 | 0.298715722 |
| 101926940 | NR_104632 LINC02104 | | long intergenic non-protein coding RNA 2104 | 57.536458 | -4.163360 | 0.048219086 | 0.298715722 |
| 101927132 | NR_110650 LINC02133 | | long intergenic non-protein coding RNA 2133 | 57.536458 | -3.790256 | 0.048219086 | 0.298715722 |
| 101927229 | NR_110743 LINC01929 | | long intergenic non-protein coding RNA 1929 | 57.536458 | -4.551402 | 0.048219086 | 0.298715722 |
| 101928509 | NR_125896 LINC02268 | | long intergenic non-protein coding RNA 2268 | 57.536458 | -4.163360 | 0.048219086 | 0.298715722 |
| 102723729 | NR_125824 LOC102723729 | | uncharacterized LOC102723729 | 57.536458 | -4.551402 | 0.048219086 | 0.298715722 |
| 102724101 | NM_001330061TP53TG3E | | TP53 target 3 family member E | 57.536458 | -2.630647 | 0.048219086 | 0.298715722 |
| 105370183 | NR_132368 LINC00390 | | long intergenic non-protein coding RNA 390 | 57.536458 | -4.551402 | 0.048219086 | 0.298715722 |
| 105371049 | NR_135175 LOC105371049 | | uncharacterized LOC105371049 | 57.536458 | -4.551402 | 0.048219086 | 0.298715722 |
| 105371907 | NR_136503 LOC105371907 | | uncharacterized LOC105371907 | 57.536458 | -4.551402 | 0.048219086 | 0.298715722 |
| 79782 | NM_001277127LRRC31 | | leucine rich repeat containing 31 | 58.746074 | 0.406737 | 1.30585E-07 | 1.5175E-05 |
| 10129 | NM_023037 FRY | | FRY microtubule binding protein | 59.608926 | 2.226913 | 2.16019E-08 | 3.43719E-06 |
| 23671 | NM_001305134TMEFF2 | | transmembrane protein with EGF like and two follistatin like | 59.699683 | -0.110676 | 5.82917E-07 | 4.94166E-05 |
| 54886 | NM_017753,N PLPPR1 | | phospholipid phosphatase related 1 | 60.298064 | -0.238025 | 2.697E-07 | 2.63738E-05 |
| 2938 | NM_001319059GSTA1 | | glutathione S-transferase alpha 1 | 61.060612 | 0.063535 | 1.49678E-07 | 1.68266E-05 |
| 79679 | NM_001253849VTCN1 | | V-set domain containing T-cell activation inhibitor 1 | 61.407852 | 0.543998 | 1.00605E-07 | 1.21877E-05 |
| 8743 | NM_001190942TNFSF10 | | TNF superfamily member 10 | 61.983201 | 2.566225 | 1.54657E-08 | 2.64388E-06 |
| 102724428 | NM_001320643LOC102724428 | | serine/threonine-protein kinase SIK1 | 62.554645 | -1.964713 | 1.3025E-05 | 0.000634079 |
| 84699 | NM_001271995CREB3L3 | | cAMP responsive element binding protein 3 like 3 | 63.756034 | 0.328628 | 1.94787E-07 | 2.02179E-05 |
| 654346 | NM_001040078LGALS9C | | galectin 9C | 64.290478 | -1.100088 | 1.23973E-06 | 9.4627E-05 |
| 89 | NM_001104,N ACTN3 | | actinin alpha 3 (gene/pseudogene) | 65.613094 | -4.444437 | 0.032764251 | 0.233618934 |
| 553 | NM_000707 AVPR1B | | arginine vasopressin receptor 1B | 65.613094 | -4.080864 | 0.032764251 | 0.233618934 |
| 6725 | NM_080823 SRMS | | src-related kinase lacking C-terminal regulatory tyrosine an | 65.613094 | -4.444437 | 0.032764251 | 0.233618934 |
| 50846 | NM_021044 DHH | | desert hedgehog | 65.613094 | -3.493979 | 0.032764251 | 0.233618934 |
| 55258 | NM_001244676THNSL2 | | threonine synthase like 2 | 65.613094 | -4.444437 | 0.032764251 | 0.233618934 |
| 57381 | NM_020663 RHOJ | | ras homolog family member J | 65.613094 | -4.444437 | 0.032764251 | 0.233618934 |
| 79334 | NM_001197287OR11H2 | | olfactory receptor family 11 subfamily H member 2 | 65.613094 | -4.444437 | 0.032764251 | 0.233618934 |

| 79946 | NM_024886 | C10orf95 | chromosome 10 open reading frame 95 | 65.613094 | -3.341421 | 0.032764251 | 0.233618934 |
| --- | --- | --- | --- | --- | --- | --- | --- |
| 81061 | NM_001005239OR11H1 | | olfactory receptor family 11 subfamily H member 1 | 65.613094 | -4.344641 | 0.032764251 | 0.233618934 |
| 85452 | NM_001304360CFAP74 | | cilia and flagella associated protein 74 | 65.613094 | -4.080864 | 0.032764251 | 0.233618934 |
| 93273 | NM_001001552LEMD1 | | LEM domain containing 1 | 65.613094 | -4.444437 | 0.032764251 | 0.233618934 |
| 116255 | NM_058165 MOGAT1 | | monoacylglycerol O-acyltransferase 1 | 65.613094 | -4.080864 | 0.032764251 | 0.233618934 |
| 127665 | NM_001009992ZNF648 | | zinc finger protein 648 | 65.613094 | -3.493979 | 0.032764251 | 0.233618934 |
| 131368 | NM_001329788ZPLD1 | | zona pellucida like domain containing 1 | 65.613094 | -3.664499 | 0.032764251 | 0.233618934 |
| 143941 | NM_001080441TTC36 | | tetratricopeptide repeat domain 36 | 65.613094 | -4.080864 | 0.032764251 | 0.233618934 |
| 157310 | NM_144962 PEBP4 | | phosphatidylethanolamine binding protein 4 | 65.613094 | -4.444437 | 0.032764251 | 0.233618934 |
| 168433 | NM_139175 RNF133 | | ring finger protein 133 | 65.613094 | -4.163567 | 0.032764251 | 0.233618934 |
| 200772 | NR_033841 LOC200772 | | uncharacterized LOC200772 | 65.613094 | -4.444437 | 0.032764251 | 0.233618934 |
| 255877 | NM_181844 BCL6B | | B-cell CLL/lymphoma 6B | 65.613094 | -3.790451 | 0.032764251 | 0.233618934 |
| 283089 | NR_033850 WDR11-AS1 | | WDR11 antisense RNA 1 | 65.613094 | -3.664499 | 0.032764251 | 0.233618934 |
| 285800 | NM_175922 PRR18 | | proline rich 18 | 65.613094 | -3.548621 | 0.032764251 | 0.233618934 |
| 339874 | NR_038976 LOC339874 | | uncharacterized LOC339874 | 65.613094 | -4.444437 | 0.032764251 | 0.233618934 |
| 339896 | NM_207359 GADL1 | | glutamate decarboxylase like 1 | 65.613094 | -4.444437 | 0.032764251 | 0.233618934 |
| 376844 | NR_001580 SDC4P | | syndecan 4 pseudogene | 65.613094 | -4.163567 | 0.032764251 | 0.233618934 |
| 390063 | NM_001005288OR51I1 | | olfactory receptor family 51 subfamily I member 1 | 65.613094 | -4.344641 | 0.032764251 | 0.233618934 |
| 390664 | NM_207419 C1QTNF8 | | C1q and TNF related 8 | 65.613094 | -4.080864 | 0.032764251 | 0.233618934 |
| 400950 | NM_001242815C2orf91 | | chromosome 2 open reading frame 91 | 65.613094 | -4.444437 | 0.032764251 | 0.233618934 |
| 440153 | NM_001013354OR11H12 | | olfactory receptor family 11 subfamily H member 12 | 65.613094 | -4.344641 | 0.032764251 | 0.233618934 |
| 100128568 | NR_046376 LOC100128568 | | uncharacterized LOC100128568 | 65.613094 | -4.444437 | 0.032764251 | 0.233618934 |
| 100133319 | NR_130736 PRO1804 | | uncharacterized LOC100133319 | 65.613094 | -4.444437 | 0.032764251 | 0.233618934 |
| 100422921 | NR_130460 MIR3149 | | microRNA 3149 | 65.613094 | -4.251277 | 0.032764251 | 0.233618934 |
| 100652865 | NR_103840,NR LINC00539 | | long intergenic non-protein coding RNA 539 | 65.613094 | -4.444437 | 0.032764251 | 0.233618934 |
| 100874092 | NR_046652 UBE2E1-AS1 | | UBE2E1 antisense RNA 1 | 65.613094 | -3.441322 | 0.032764251 | 0.233618934 |
| 100996447 | NR_104147 LOC100996447 | | uncharacterized LOC100996447 | 65.613094 | -4.444437 | 0.032764251 | 0.233618934 |
| 101927292 | NR_110048 LINC02395 | | long intergenic non-protein coding RNA 2395 | 65.613094 | -4.444437 | 0.032764251 | 0.233618934 |
| 101927688 | NR_110810 SEPT4-AS1 | | SEPT4 antisense RNA 1 | 65.613094 | -4.444437 | 0.032764251 | 0.233618934 |
| 101929176 | NR_109915 CTD-2297D10.2 | | uncharacterized LOC101929176 | 65.613094 | -4.444437 | 0.032764251 | 0.233618934 |
| 101929437 | NR_132102 PRRX2-AS1 | | PRRX2 antisense RNA 1 | 65.613094 | -4.444437 | 0.032764251 | 0.233618934 |
| 101929607 | NR_110039 LINC01990 | | long intergenic non-protein coding RNA 1990 | 65.613094 | -4.163567 | 0.032764251 | 0.233618934 |
| 102723322 | NR_134311 LOC102723322 | | uncharacterized LOC102723322 | 65.613094 | -4.163567 | 0.032764251 | 0.233618934 |
| 102724470 | NR_146509 LINC01887 | | long intergenic non-protein coding RNA 1887 | 65.613094 | -4.444437 | 0.032764251 | 0.233618934 |
| 105377682 | NR_134261 LINC01932 | | long intergenic non-protein coding RNA 1932 | 65.613094 | -3.548621 | 0.032764251 | 0.233618934 |
| 11213 | NM_001142523IRAK3 | | interleukin 1 receptor associated kinase 3 | 65.728488 | 1.182857 | 1.09309E-06 | 8.43681E-05 |
| 6097 | NM_001001523RORC | | RAR related orphan receptor C | 66.036177 | 1.223462 | 2.74037E-08 | 4.19886E-06 |
| 8302 | NM_013431 KLRC4 | | killer cell lectin like receptor C4 | 66.091136 | 0.858861 | 4.85029E-08 | 6.60054E-06 |
| 654463 | NM_001039112FER1L6 | | fer-1 like family member 6 | 67.693788 | 2.974076 | 7.39201E-09 | 1.39003E-06 |
| 100533496 | NM_001204478TVP23C-CDRT4 | | TVP23C-CDRT4 readthrough | 68.823491 | 4.222394 | 4.96165E-09 | 1.01615E-06 |
| 284194 | NM_001042685LGALS9B | | galectin 9B | 69.083845 | -1.001836 | 8.22648E-07 | 6.6212E-05 |
| 9452 | NM_001171581ITM2A | | integral membrane protein 2A | 71.165970 | -0.015567 | 1.00131E-07 | 1.21877E-05 |
| 5918 | NM_002888,N RARRES1 | | retinoic acid receptor responder 1 | 71.473515 | 5.081798 | 3.37098E-09 | 7.33986E-07 |

| 10008 | NM_005472 | KCNE3 | potassium voltage-gated channel subfamily E regulatory su | 73.388951 | 0.104725 | 8.35835E-08 | 1.05422E-05 |
| --- | --- | --- | --- | --- | --- | --- | --- |
| 335 | NM_000039,N | APOA1 | apolipoprotein A1 | 73.689731 | -3.664689 | 0.02277515 | 0.184819135 |
| 6010 | NM_000539 | RHO | rhodopsin | 73.689731 | -4.344853 | 0.02277515 | 0.184819135 |
| 8339 | NM_003518 | HIST1H2BG | histone cluster 1 H2B family member g | 73.689731 | -4.344853 | 0.02277515 | 0.184819135 |
| 8468 | NM_001135211FKBP6 | | FK506 binding protein 6 | 73.689731 | -4.344853 | 0.02277515 | 0.184819135 |
| 9227 | NM_001301645LRAT | | lecithin retinol acyltransferase (phosphatidylcholine--retinol | 73.689731 | -3.248135 | 0.02277515 | 0.184819135 |
| 10886 | NM_001144756NPFFR2 | | neuropeptide FF receptor 2 | 73.689731 | -4.344853 | 0.02277515 | 0.184819135 |
| 23498 | NM_012205 HAAO | | 3-hydroxyanthranilate 3,4-dioxygenase | 73.689731 | -3.605590 | 0.02277515 | 0.184819135 |
| 54766 | NM_017589 BTG4 | | BTG anti-proliferation factor 4 | 73.689731 | -3.341598 | 0.02277515 | 0.184819135 |
| 56159 | NM_001003811TEX11 | | testis expressed 11 | 73.689731 | -4.344853 | 0.02277515 | 0.184819135 |
| 56300 | NM_001278568IL36G | | interleukin 36, gamma | 73.689731 | -4.344853 | 0.02277515 | 0.184819135 |
| 57471 | NM_001009959ERMN | | ermin | 73.689731 | -4.344853 | 0.02277515 | 0.184819135 |
| 57501 | NM_001348520KIAA1257 | | KIAA1257 | 73.689731 | -3.857980 | 0.02277515 | 0.184819135 |
| 79981 | NM_001122841FRMD1 | | FERM domain containing 1 | 73.689731 | -4.344853 | 0.02277515 | 0.184819135 |
| 84466 | NM_001256545MEGF10 | | multiple EGF like domains 10 | 73.689731 | -3.341598 | 0.02277515 | 0.184819135 |
| 84530 | NM_194286 SRRM4 | | serine/arginine repetitive matrix 4 | 73.689731 | -4.002828 | 0.02277515 | 0.184819135 |
| 84873 | NM_001308362ADGRG7 | | adhesion G protein-coupled receptor G7 | 73.689731 | -3.790646 | 0.02277515 | 0.184819135 |
| 115560 | NM_001258280ZNF501 | | zinc finger protein 501 | 73.689731 | -4.344853 | 0.02277515 | 0.184819135 |
| 120935 | NM_182496 CCDC38 | | coiled-coil domain containing 38 | 73.689731 | -3.664689 | 0.02277515 | 0.184819135 |
| 124221 | NR_026864 PRSS30P | | protease, serine, 30 pseudogene | 73.689731 | -4.344853 | 0.02277515 | 0.184819135 |
| 131375 | NM_001304386LYZL4 | | lysozyme like 4 | 73.689731 | -4.344853 | 0.02277515 | 0.184819135 |
| 148741 | NM_001280799ANKRD35 | | ankyrin repeat domain 35 | 73.689731 | -4.344853 | 0.02277515 | 0.184819135 |
| 255167 | NR_024423,NR LINC01018 | | long intergenic non-protein coding RNA 1018 | 73.689731 | -3.664689 | 0.02277515 | 0.184819135 |
| 284837 | NR_026961 AATBC | | apoptosis associated transcript in bladder cancer | 73.689731 | -4.344853 | 0.02277515 | 0.184819135 |
| 285555 | NM_174952 STPG2 | | sperm tail PG-rich repeat containing 2 | 73.689731 | -4.344853 | 0.02277515 | 0.184819135 |
| 344807 | NM_001008784CD200R1L | | CD200 receptor 1 like | 73.689731 | -3.928596 | 0.02277515 | 0.184819135 |
| 353149 | NM_178571 TBC1D26 | | TBC1 domain family member 26 | 73.689731 | -3.790646 | 0.02277515 | 0.184819135 |
| 389384 | NM_001010903C6orf222 | | chromosome 6 open reading frame 222 | 73.689731 | -4.344853 | 0.02277515 | 0.184819135 |
| 392517 | NM_001348372NCBP2L | | nuclear cap binding protein subunit 2-like | 73.689731 | -4.344853 | 0.02277515 | 0.184819135 |
| 406888 | NR_029483 MIRLET7F1 | | microRNA let-7f-1 | 73.689731 | -4.163774 | 0.02277515 | 0.184819135 |
| 653361 | NM_000265 NCF1 | | neutrophil cytosolic factor 1 | 73.689731 | -4.344853 | 0.02277515 | 0.184819135 |
| 100133301 | NM_001310155FAM231B | | family with sequence similarity 231 member B | 73.689731 | -4.163774 | 0.02277515 | 0.184819135 |
| 100188953 | NR_024129 LINC00092 | | long intergenic non-protein coding RNA 92 | 73.689731 | -4.344853 | 0.02277515 | 0.184819135 |
| 100505530 | NR_126057 LOC100505530 | | uncharacterized LOC100505530 | 73.689731 | -4.344853 | 0.02277515 | 0.184819135 |
| 100506305 | NR_038904 LINC00958 | | long intergenic non-protein coding RNA 958 | 73.689731 | -4.344853 | 0.02277515 | 0.184819135 |
| 100874235 | NR_046579 CACNA1C-AS2 | | CACNA1C antisense RNA 2 | 73.689731 | -4.344853 | 0.02277515 | 0.184819135 |
| 100996930 | NR_138043 LINC00621 | | long intergenic non-protein coding RNA 621 | 73.689731 | -3.928596 | 0.02277515 | 0.184819135 |
| 101927811 | NR_110119,NR LOC101927811 | | uncharacterized LOC101927811 | 73.689731 | -3.441504 | 0.02277515 | 0.184819135 |
| 101927931 | NR_110724 LINC01480 | | long intergenic non-protein coding RNA 1480 | 73.689731 | -4.344853 | 0.02277515 | 0.184819135 |
| 101928015 | NR_147054 LINC01985 | | long intergenic non-protein coding RNA 1985 | 73.689731 | -4.344853 | 0.02277515 | 0.184819135 |
| 101928569 | NR_104669 LINC02101 | | long intergenic non-protein coding RNA 2101 | 73.689731 | -4.081068 | 0.02277515 | 0.184819135 |
| 101929111 | NR_144465 LINC01149 | | long intergenic non-protein coding RNA 1149 | 73.689731 | -4.344853 | 0.02277515 | 0.184819135 |
| 101929726 | NM_001315494LOC101929726 | | uncharacterized LOC101929726 | 73.689731 | -4.163774 | 0.02277515 | 0.184819135 |

| 102723849 | NM_001351351SPDYE17 | speedy/RINGO cell cycle regulator family member E17 | 73.689731 | -4.344853 | 0.02277515 | 0.184819135 |
| --- | --- | --- | --- | --- | --- | --- |
| 79092 | NM_001257970CARD14 | caspase recruitment domain family member 14 | 74.729594 | 2.424016 | 7.80509E-09 | 1.42875E-06 |
| 102465252 | NR_106761 MIR6506 | microRNA 6506 | 75.224158 | -1.016239 | 4.79131E-06 | 0.000281099 |
| 9966 | NM_001204344TNFSF15 | TNF superfamily member 15 | 79.732690 | 5.770085 | 1.62261E-09 | 3.7293E-07 |
| 8764 | NM_001297605TNFRSF14 | TNF receptor superfamily member 14 | 80.862798 | 2.022329 | 6.30005E-09 | 1.21791E-06 |
| 4914 | NM_001007792NTRK1 | neurotrophic receptor tyrosine kinase 1 | 81.766368 | -3.726493 | 0.016154467 | 0.146302603 |
| 8431 | NM_021969 NR0B2 | nuclear receptor subfamily 0 group B member 2 | 81.766368 | -4.251697 | 0.016154467 | 0.146302603 |
| 10584 | NM_001324095COLEC10 | collectin subfamily member 10 | 81.766368 | -4.251697 | 0.016154467 | 0.146302603 |
| 11181 | NM_001301065TREH | trehalase | 81.766368 | -4.251697 | 0.016154467 | 0.146302603 |
| 27178 | NM_014439,N IL37 | interleukin 37 | 81.766368 | -4.081273 | 0.016154467 | 0.146302603 |
| 80078 | NR_130915 LCAL1 | lung cancer associated lncRNA 1 | 81.766368 | -3.928795 | 0.016154467 | 0.146302603 |
| 114131 | NM_053049 UCN3 | urocortin 3 | 81.766368 | -4.251697 | 0.016154467 | 0.146302603 |
| 114836 | NM_001184714SLAMF6 | SLAM family member 6 | 81.766368 | -4.251697 | 0.016154467 | 0.146302603 |
| 116535 | NM_001098515MRGPRF | MAS related GPR family member F | 81.766368 | -3.548992 | 0.016154467 | 0.146302603 |
| 125115 | NM_182497 KRT40 | keratin 40 | 81.766368 | -3.790840 | 0.016154467 | 0.146302603 |
| 128218 | NM_001320244TMEM125 | transmembrane protein 125 | 81.766368 | -4.251697 | 0.016154467 | 0.146302603 |
| 149954 | NM_182519 BPIFB4 | BPI fold containing family B member 4 | 81.766368 | -4.251697 | 0.016154467 | 0.146302603 |
| 158798 | NM_001008534AKAP14 | A-kinase anchoring protein 14 | 81.766368 | -4.251697 | 0.016154467 | 0.146302603 |
| 165140 | NM_148962 OXER1 | oxoeicosanoid receptor 1 | 81.766368 | -3.790840 | 0.016154467 | 0.146302603 |
| 285708 | NR_103753,NR LINC00491 | long intergenic non-protein coding RNA 491 | 81.766368 | -2.820324 | 0.016154467 | 0.146302603 |
| 286204 | NM_173689,NRCRB2 | crumbs 2, cell polarity complex component | 81.766368 | -4.003030 | 0.016154467 | 0.146302603 |
| 317716 | NR_026760 BPIFA4P | BPI fold containing family A member 4, pseudogene | 81.766368 | -4.251697 | 0.016154467 | 0.146302603 |
| 400867 | NR_120405 LOC400867 | uncharacterized LOC400867 | 81.766368 | -4.251697 | 0.016154467 | 0.146302603 |
| 414760 | NR_104117 HCG14 | HLA complex group 14 (non-protein coding) | 81.766368 | -3.928795 | 0.016154467 | 0.146302603 |
| 441086 | NR_134288 LOC441086 | uncharacterized LOC441086 | 81.766368 | -3.203742 | 0.016154467 | 0.146302603 |
| 767811 | NR_003238,NR H2BFXP | H2B histone family member X, pseudogene | 81.766368 | -3.790840 | 0.016154467 | 0.146302603 |
| 100506697 | NR_038995 LINC00327 | long intergenic non-protein coding RNA 327 | 81.766368 | -3.441686 | 0.016154467 | 0.146302603 |
| 101927472 | NR_120622 LOC101927472 | uncharacterized LOC101927472 | 81.766368 | -4.251697 | 0.016154467 | 0.146302603 |
| 101927762 | NR_120629 LOC101927762 | uncharacterized LOC101927762 | 81.766368 | -4.251697 | 0.016154467 | 0.146302603 |
| 101928233 | NR_131192 LINC01671 | long intergenic non-protein coding RNA 1671 | 81.766368 | -3.664880 | 0.016154467 | 0.146302603 |
| 101928324 | NR_125952,NR LOC101928324 | uncharacterized LOC101928324 | 81.766368 | -4.251697 | 0.016154467 | 0.146302603 |
| 101928973 | NR_125966 LINC01732 | long intergenic non-protein coding RNA 1732 | 81.766368 | -4.251697 | 0.016154467 | 0.146302603 |
| 102724127 | NM_001330066TP53TG3F | TP53 target 3 family member F | 81.766368 | -3.341776 | 0.016154467 | 0.146302603 |
| 104266958 | NR_126348 LINC01151 | long intergenic non-protein coding RNA 1151 | 81.766368 | -4.251697 | 0.016154467 | 0.146302603 |
| 1472 | NM_001899 CST4 | cystatin S | 84.985969 | 2.276965 | 3.14558E-09 | 6.92194E-07 |
| 150 | NM_000681 ADRA2A | adrenoceptor alpha 2A | 85.128246 | -0.145788 | 7.39036E-08 | 9.49501E-06 |
| 8537 | NM_001316361BCAS1 | breast carcinoma amplified sequence 1 | 88.383904 | 4.959476 | 9.65277E-10 | 2.41062E-07 |
| 246 | NM_001140 ALOX15 | arachidonate 15-lipoxygenase | 89.843005 | -3.928995 | 0.011667115 | 0.118301114 |
| 2134 | NM_004455 EXTL1 | exostosin like glycosyltransferase 1 | 89.843005 | -3.791035 | 0.011667115 | 0.118301114 |
| 3047 | NM_000559 HBG1 | hemoglobin subunit gamma 1 | 89.843005 | -4.164189 | 0.011667115 | 0.118301114 |
| 3751 | NM_012281 KCND2 | potassium voltage-gated channel subfamily D member 2 | 89.843005 | -3.605967 | 0.011667115 | 0.118301114 |
| 9104 | NM_001282848RGN | regucalcin | 89.843005 | -3.605967 | 0.011667115 | 0.118301114 |
| 57624 | NM_020864 NYAP2 | neuronal tyrosine-phosphorylated phosphoinositide-3-kinas | 89.843005 | -3.726685 | 0.011667115 | 0.118301114 |

| 441457 | NM_001045477NUTM2G | NUT family member 2G | 89.843005 | -4.164189 | 0.011667115 | 0.118301114 |
| --- | --- | --- | --- | --- | --- | --- |
| 643406 | NR_029405 LOC643406 | uncharacterized LOC643406 | 89.843005 | -4.164189 | 0.011667115 | 0.118301114 |
| 646730 | NR_136190 LOC646730 | uncharacterized LOC646730 | 89.843005 | -3.726685 | 0.011667115 | 0.118301114 |
| 101928244 | NR_120464 TESC-AS1 | TESC antisense RNA 1 (head to head) | 89.843005 | -3.726685 | 0.011667115 | 0.118301114 |
| 101929134 | NR_134662,NR LOC101929134 | uncharacterized LOC101929134 | 89.843005 | -3.726685 | 0.011667115 | 0.118301114 |
| 4982 | NM_002546 TNFRSF11B | TNF receptor superfamily member 11b | 90.354688 | 0.934074 | 9.87898E-09 | 1.76161E-06 |
| 50853 | NM_015873 VILL | villin like | 94.176193 | 1.820374 | 2.79126E-09 | 6.27579E-07 |
| 55286 | NM_001104629C4orf19 | chromosome 4 open reading frame 19 | 95.234469 | 0.078077 | 3.73323E-08 | 5.32564E-06 |
| 474384 | NM_001007524F8A3 | coagulation factor VIII-associated 3 | 97.190512 | 0.027032 | 3.31062E-08 | 4.82254E-06 |
| 1590 | NR_040090 CYP21A1P | cytochrome P450 family 21 subfamily A member 1, pseudo | 97.919642 | -2.014601 | 0.008564159 | 0.094530216 |
| 3101 | NM_002115 HK3 | hexokinase 3 | 97.919642 | -4.081682 | 0.008564159 | 0.094530216 |
| 3294 | NM_002153 HSD17B2 | hydroxysteroid 17-beta dehydrogenase 2 | 97.919642 | -3.929195 | 0.008564159 | 0.094530216 |
| 7066 | NM_000460,N THPO | thrombopoietin | 97.919642 | -4.081682 | 0.008564159 | 0.094530216 |
| 7634 | NM_007136 ZNF80 | zinc finger protein 80 | 97.919642 | -4.081682 | 0.008564159 | 0.094530216 |
| 10941 | NM_001252274UGT2A1 | UDP glucuronosyltransferase family 2 member A1 complex | 97.919642 | -4.081682 | 0.008564159 | 0.094530216 |
| 133491 | NM_001144954C5orf47 | chromosome 5 open reading frame 47 | 97.919642 | -3.665260 | 0.008564159 | 0.094530216 |
| 147646 | NM_001193623C19orf84 | chromosome 19 open reading frame 84 | 97.919642 | -2.925413 | 0.008564159 | 0.094530216 |
| 155006 | NM_001085429TMEM213 | transmembrane protein 213 | 97.919642 | -3.929195 | 0.008564159 | 0.094530216 |
| 158434 | NR_132344 LOC158434 | uncharacterized LOC158434 | 97.919642 | -4.081682 | 0.008564159 | 0.094530216 |
| 253128 | NR_034140 LINC00612 | long intergenic non-protein coding RNA 612 | 97.919642 | -3.665260 | 0.008564159 | 0.094530216 |
| 255130 | NR_034081 IGFBP7-AS1 | IGFBP7 antisense RNA 1 | 97.919642 | -3.726878 | 0.008564159 | 0.094530216 |
| 259293 | NM_001097643TAS2R30 | taste 2 receptor member 30 | 97.919642 | -3.791230 | 0.008564159 | 0.094530216 |
| 285389 | NR_136179,NR LINC02028 | long intergenic non-protein coding RNA 2028 | 97.919642 | -4.081682 | 0.008564159 | 0.094530216 |
| 286676 | NM_001199799ILDR1 | immunoglobulin like domain containing receptor 1 | 97.919642 | -4.081682 | 0.008564159 | 0.094530216 |
| 317719 | NM_001329595KLHL10 | kelch like family member 10 | 97.919642 | -3.494714 | 0.008564159 | 0.094530216 |
| 342346 | NM_001145011C16orf96 | chromosome 16 open reading frame 96 | 97.919642 | -4.081682 | 0.008564159 | 0.094530216 |
| 406891 | NR_029661 MIRLET7I | microRNA let-7i | 97.919642 | -4.003434 | 0.008564159 | 0.094530216 |
| 643714 | NR_033920 CASC16 | cancer susceptibility 16 (non-protein coding) | 97.919642 | -4.081682 | 0.008564159 | 0.094530216 |
| 100129060 | NR_135301 SEMA3F-AS1 | SEMA3F antisense RNA 1 | 97.919642 | -4.081682 | 0.008564159 | 0.094530216 |
| 100129203 | NR_110295 LOC100129203 | uncharacterized LOC100129203 | 97.919642 | -3.160844 | 0.008564159 | 0.094530216 |
| 100505518 | NR_103853 EDNRB-AS1 | EDNRB antisense RNA 1 | 97.919642 | -4.081682 | 0.008564159 | 0.094530216 |
| 100873955 | NR_046827 LNX1-AS2 | LNX1 antisense RNA 2 | 97.919642 | -3.204086 | 0.008564159 | 0.094530216 |
| 101927102 | NR_110903,NR ITFG1-AS1 | ITFG1 antisense RNA 1 | 97.919642 | -3.929195 | 0.008564159 | 0.094530216 |
| 101927497 | NR_110086,NR LOC101927497 | uncharacterized LOC101927497 | 97.919642 | -3.606155 | 0.008564159 | 0.094530216 |
| 101928907 | NR_135690 LOC101928907 | uncharacterized LOC101928907 | 97.919642 | -3.549364 | 0.008564159 | 0.094530216 |
| 104797536 | NR_126557 LINC01402 | long intergenic non-protein coding RNA 1402 | 97.919642 | -3.858572 | 0.008564159 | 0.094530216 |
| 105372273 | NR_134909 LOC105372273 | uncharacterized LOC105372273 | 97.919642 | -4.081682 | 0.008564159 | 0.094530216 |
| 401546 | NM_001012993C9orf152 | chromosome 9 open reading frame 152 | 98.201996 | 3.224552 | 8.7152E-10 | 2.2256E-07 |
| 7366 | NM_001076 UGT2B15 | UDP glucuronosyltransferase family 2 member B15 | 101.454104 | 2.026358 | 1.58711E-09 | 3.68869E-07 |
| 1080 | NM_000492 CFTR | cystic fibrosis transmembrane conductance regulator | 102.158083 | -0.347509 | 8.25423E-08 | 1.04748E-05 |
| 400746 | NM_001010980NCMAP | non-compact myelin associated protein | 102.767637 | 3.448282 | 6.60086E-10 | 1.77323E-07 |
| 100506551 | NR_103809 LOC100506551 | uncharacterized LOC100506551 | 104.600948 | 0.865231 | 5.9691E-09 | 1.17591E-06 |
| 2207 | NM_004106 FCER1G | Fc fragment of IgE receptor Ig | 105.996278 | -3.494898 | 0.006379424 | 0.076320644 |

| 2212 | NM_001136219FCGR2A | Fc fragment of IgG receptor IIa | 105.996278 | -3.727070 | 0.006379424 | 0.076320644 |
| --- | --- | --- | --- | --- | --- | --- |
| 2953 | NM_000854,N GSTT2 | glutathione S-transferase theta 2 (gene/pseudogene) | 105.996278 | -2.820791 | 0.006379424 | 0.076320644 |
| 3817 | NM_001002231KLK2 | kallikrein related peptidase 2 | 105.996278 | -3.606343 | 0.006379424 | 0.076320644 |
| 8510 | NM_006983 MMP23B | matrix metallopeptidase 23B | 105.996278 | -3.727070 | 0.006379424 | 0.076320644 |
| 11185 | NM_001199219INMT | indolethylamine N-methyltransferase | 105.996278 | -4.003636 | 0.006379424 | 0.076320644 |
| 84677 | NR_026838,NR DSCR8 | Down syndrome critical region 8 (non-protein coding) | 105.996278 | -4.003636 | 0.006379424 | 0.076320644 |
| 121214 | NM_148897 SDR9C7 | short chain dehydrogenase/reductase family 9C member 7 | 105.996278 | -3.606343 | 0.006379424 | 0.076320644 |
| 124773 | NM_181707 C17orf64 | chromosome 17 open reading frame 64 | 105.996278 | -2.854884 | 0.006379424 | 0.076320644 |
| 147872 | NM_144688 CCDC155 | coiled-coil domain containing 155 | 105.996278 | -4.003636 | 0.006379424 | 0.076320644 |
| 151871 | NM_138815 DPPA2 | developmental pluripotency associated 2 | 105.996278 | -4.003636 | 0.006379424 | 0.076320644 |
| 161635 | NR_027320 CSNK1A1P1 | casein kinase 1 alpha 1 pseudogene 1 | 105.996278 | -4.003636 | 0.006379424 | 0.076320644 |
| 284085 | NR_028334 KRT18P55 | keratin 18 pseudogene 55 | 105.996278 | -4.003636 | 0.006379424 | 0.076320644 |
| 285889 | NR_038232 LOC285889 | uncharacterized LOC285889 | 105.996278 | -4.003636 | 0.006379424 | 0.076320644 |
| 389812 | NM_203347 LCN15 | lipocalin 15 | 105.996278 | -4.003636 | 0.006379424 | 0.076320644 |
| 400578 | NR_029392 KRT16P2 | keratin 16 pseudogene 2 | 105.996278 | -3.342309 | 0.006379424 | 0.076320644 |
| 100128569 | NM_001135580C19orf71 | chromosome 19 open reading frame 71 | 105.996278 | -3.606343 | 0.006379424 | 0.076320644 |
| 100874071 | NR_046548 USP12-AS2 | USP12 antisense RNA 2 (head to head) | 105.996278 | -3.204258 | 0.006379424 | 0.076320644 |
| 101927056 | NR_125395,NR LOC101927056 | uncharacterized LOC101927056 | 105.996278 | -3.727070 | 0.006379424 | 0.076320644 |
| 101927571 | NR_110782 LOC101927571 | uncharacterized LOC101927571 | 105.996278 | -3.606343 | 0.006379424 | 0.076320644 |
| 101929688 | NR_109755 LINC01251 | long intergenic non-protein coding RNA 1251 | 105.996278 | -3.665450 | 0.006379424 | 0.076320644 |
| 110091768 | NR_146604 LOC110091768 | uncharacterized LOC110091768 | 105.996278 | -3.665450 | 0.006379424 | 0.076320644 |
| 319 | NM_001638 APOF | apolipoprotein F | 114.072915 | -3.929594 | 0.00481584 | 0.062415821 |
| 1188 | NM_000085,N CLCNKB | chloride voltage-gated channel Kb | 114.072915 | -2.855041 | 0.00481584 | 0.062415821 |
| 1259 | NM_000087,N CNGA1 | cyclic nucleotide gated channel alpha 1 | 114.072915 | -3.727263 | 0.00481584 | 0.062415821 |
| 3316 | NM_001541 HSPB2 | heat shock protein family B (small) member 2 | 114.072915 | -3.929594 | 0.00481584 | 0.062415821 |
| 5450 | NM_006235 POU2AF1 | POU class 2 associating factor 1 | 114.072915 | -3.929594 | 0.00481584 | 0.062415821 |
| 7484 | NM_001320458WNT9B | Wnt family member 9B | 114.072915 | -3.342487 | 0.00481584 | 0.062415821 |
| 51554 | NM_016557,N ACKR4 | atypical chemokine receptor 4 | 114.072915 | -3.929594 | 0.00481584 | 0.062415821 |
| 80352 | NM_025236,N RNF39 | ring finger protein 39 | 114.072915 | -3.665641 | 0.00481584 | 0.062415821 |
| 81033 | NM_001278919KCNH6 | potassium voltage-gated channel subfamily H member 6 | 114.072915 | -3.929594 | 0.00481584 | 0.062415821 |
| 135892 | NM_001281450TRIM50 | tripartite motif containing 50 | 114.072915 | -3.858966 | 0.00481584 | 0.062415821 |
| 171389 | NM_001276700NLRP6 | NLR family pyrin domain containing 6 | 114.072915 | -3.549736 | 0.00481584 | 0.062415821 |
| 283777 | NM_182562 FAM169B | family with sequence similarity 169 member B | 114.072915 | -3.665641 | 0.00481584 | 0.062415821 |
| 285848 | NM_001145716PNPLA1 | patatin like phospholipase domain containing 1 | 114.072915 | -2.755072 | 0.00481584 | 0.062415821 |
| 399717 | NR_024256,NR GATA3-AS1 | GATA3 antisense RNA 1 | 114.072915 | -3.665641 | 0.00481584 | 0.062415821 |
| 414157 | NM_001009997C10orf62 | chromosome 10 open reading frame 62 | 114.072915 | -3.391589 | 0.00481584 | 0.062415821 |
| 101927084 | NR_119383 LINC01359 | long intergenic non-protein coding RNA 1359 | 114.072915 | -3.727263 | 0.00481584 | 0.062415821 |
| 101927282 | NR_110753 LINC02466 | long intergenic non-protein coding RNA 2466 | 114.072915 | -3.391589 | 0.00481584 | 0.062415821 |
| 4069 | NM_000239 LYZ | lysozyme | 114.832443 | 3.685169 | 2.9706E-10 | 9.60107E-08 |
| 145837 | NR_026979 DRAIC | downregulated RNA in cancer, inhibitor of cell invasion and | 115.662036 | 2.648093 | 5.10328E-10 | 1.4265E-07 |
| 185 | NM_000685,N AGTR1 | angiotensin II receptor type 1 | 118.292606 | 3.887393 | 2.40133E-10 | 8.01153E-08 |
| 146336 | NR_027242 SSTR5-AS1 | SSTR5 antisense RNA 1 | 118.569191 | 2.653063 | 4.37476E-10 | 1.25683E-07 |
| 1180 | NM_000083,NRCLCN1 | chloride voltage-gated channel 1 | 122.149552 | -3.495266 | 0.003680029 | 0.051642736 |

| 1638 | NM_001129889DCT | dopachrome tautomerase | 122.149552 | -3.727455 | 0.003680029 | 0.051642736 |
| --- | --- | --- | --- | --- | --- | --- |
| 4975 | NM_006189 OMP | olfactory marker protein | 122.149552 | -3.859163 | 0.003680029 | 0.051642736 |
| 5409 | NM_002686,NRPNMT | phenylethanolamine N-methyltransferase | 122.149552 | -3.859163 | 0.003680029 | 0.051642736 |
| 57573 | NM_001321768ZNF471 | zinc finger protein 471 | 122.149552 | -3.295168 | 0.003680029 | 0.051642736 |
| 83881 | NM_001282402MIXL1 | Mix paired-like homeobox | 122.149552 | -3.078547 | 0.003680029 | 0.051642736 |
| 121506 | NM_001300784ERP27 | endoplasmic reticulum protein 27 | 122.149552 | -3.859163 | 0.003680029 | 0.051642736 |
| 131831 | NM_001308234ERICH6 | glutamate rich 6 | 122.149552 | -3.665831 | 0.003680029 | 0.051642736 |
| 153643 | NM_152548 FAM81B | family with sequence similarity 81 member B | 122.149552 | -3.665831 | 0.003680029 | 0.051642736 |
| 162282 | NM_153228 ANKFN1 | ankyrin repeat and fibronectin type III domain containing 1 | 122.149552 | -2.244801 | 0.003680029 | 0.051642736 |
| 401563 | NM_207511 C9orf139 | chromosome 9 open reading frame 139 | 122.149552 | -3.859163 | 0.003680029 | 0.051642736 |
| 646915 | NM_001304449ZNF806 | zinc finger protein 806 | 122.149552 | -3.442594 | 0.003680029 | 0.051642736 |
| 653316 | NR_038353 FAM153C | family with sequence similarity 153 member C | 122.149552 | -3.606719 | 0.003680029 | 0.051642736 |
| 100506178 | NR_038393 LOC100506178 | uncharacterized LOC100506178 | 122.149552 | -3.119360 | 0.003680029 | 0.051642736 |
| 100506371 | NR_104420 LINC02091 | long intergenic non-protein coding RNA 2091 | 122.149552 | -3.727455 | 0.003680029 | 0.051642736 |
| 101929331 | NR_121587 LOC101929331 | uncharacterized LOC101929331 | 122.149552 | -3.859163 | 0.003680029 | 0.051642736 |
| 104355220 | NR_126400 LINC01219 | long intergenic non-protein coding RNA 1219 | 122.149552 | -3.859163 | 0.003680029 | 0.051642736 |
| 2330 | NM_001144829FMO5 | flavin containing monooxygenase 5 | 122.675752 | 3.869667 | 1.94998E-10 | 6.83651E-08 |
| 130 | NM_000672,N ADH6 | alcohol dehydrogenase 6 (class V) | 124.925565 | 2.644087 | 3.39876E-10 | 1.0493E-07 |
| 10863 | NM_001304351ADAM28 | ADAM metallopeptidase domain 28 | 125.310837 | 3.763294 | 1.83414E-10 | 6.54125E-08 |
| 942 | NM_001206924CD86 | CD86 molecule | 130.226189 | -3.792009 | 0.002843659 | 0.0427381 |
| 3932 | NM_001042771LCK | LCK proto-oncogene, Src family tyrosine kinase | 130.226189 | -3.792009 | 0.002843659 | 0.0427381 |
| 8477 | NM_003608 GPR65 | G protein-coupled receptor 65 | 130.226189 | -3.792009 | 0.002843659 | 0.0427381 |
| 9154 | NM_001287761SLC28A1 | solute carrier family 28 member 1 | 130.226189 | -3.792009 | 0.002843659 | 0.0427381 |
| 9348 | NM_004784,NRNDST3 | N-deacetylase and N-sulfotransferase 3 | 130.226189 | -3.666021 | 0.002843659 | 0.0427381 |
| 55018 | NR_073199 LINC00483 | long intergenic non-protein coding RNA 483 | 130.226189 | -3.792009 | 0.002843659 | 0.0427381 |
| 57047 | NM_001199978PLSCR2 | phospholipid scramblase 2 | 130.226189 | -3.119528 | 0.002843659 | 0.0427381 |
| 57348 | NM_001005367TTYH1 | tweety family member 1 | 130.226189 | -3.792009 | 0.002843659 | 0.0427381 |
| 79258 | NM_033467 MMEL1 | membrane metalloendopeptidase like 1 | 130.226189 | -3.391949 | 0.002843659 | 0.0427381 |
| 80108 | NM_030613 ZFP2 | ZFP2 zinc finger protein | 130.226189 | -3.342842 | 0.002843659 | 0.0427381 |
| 254050 | NM_001098519LRRC43 | leucine rich repeat containing 43 | 130.226189 | -3.000376 | 0.002843659 | 0.0427381 |
| 340090 | NR_134291 LOC340090 | uncharacterized LOC340090 | 130.226189 | -3.161525 | 0.002843659 | 0.0427381 |
| 388574 | NR_029406 RPL23AP87 | ribosomal protein L23a pseudogene 87 | 130.226189 | -2.855355 | 0.002843659 | 0.0427381 |
| 389015 | NM_001011552SLC9A4 | solute carrier family 9 member A4 | 130.226189 | -3.792009 | 0.002843659 | 0.0427381 |
| 643339 | NR_040096 LOC643339 | uncharacterized LOC643339 | 130.226189 | -3.204774 | 0.002843659 | 0.0427381 |
| 693213 | NR_030358 MIR628 | microRNA 628 | 130.226189 | -3.727648 | 0.002843659 | 0.0427381 |
| 100852410 | NR_046217 ZRANB2-AS2 | ZRANB2 antisense RNA 2 (head to head) | 130.226189 | -3.442776 | 0.002843659 | 0.0427381 |
| 101927604 | NR_110681 LOC101927604 | uncharacterized LOC101927604 | 130.226189 | -3.391949 | 0.002843659 | 0.0427381 |
| 101928150 | NR_120634 LOC101928150 | uncharacterized LOC101928150 | 130.226189 | -3.792009 | 0.002843659 | 0.0427381 |
| 6947 | NM_001062 TCN1 | transcobalamin 1 | 134.332801 | 4.140726 | 1.05455E-10 | 4.45169E-08 |
| 362 | NM_001651 AQP5 | aquaporin 5 | 138.302826 | -3.727840 | 0.002220049 | 0.036130384 |
| 433 | NM_001181,N ASGR2 | asialoglycoprotein receptor 2 | 138.302826 | -3.727840 | 0.002220049 | 0.036130384 |
| 1007 | NM_016279 CDH9 | cadherin 9 | 138.302826 | -3.727840 | 0.002220049 | 0.036130384 |
| 1607 | NM_001350705DGKB | diacylglycerol kinase beta | 138.302826 | -2.890432 | 0.002220049 | 0.036130384 |

| 9402 | NM_001291824GRAP2 | GRB2-related adaptor protein 2 | 138.302826 | -3.727840 | 0.002220049 | 0.036130384 |
| --- | --- | --- | --- | --- | --- | --- |
| 26070 | NR_026882 DKFZP434K028 | uncharacterized LOC26070 | 138.302826 | -3.727840 | 0.002220049 | 0.036130384 |
| 27232 | NM_001318865GNMT | glycine N-methyltransferase | 138.302826 | -3.727840 | 0.002220049 | 0.036130384 |
| 63970 | NM_001195194TP53AIP1 | tumor protein p53 regulated apoptosis inducing protein 1 | 138.302826 | -2.926213 | 0.002220049 | 0.036130384 |
| 148345 | NM_001170754C1orf127 | chromosome 1 open reading frame 127 | 138.302826 | -3.442958 | 0.002220049 | 0.036130384 |
| 283575 | NR_110554 LINC02288 | long intergenic non-protein coding RNA 2288 | 138.302826 | -3.550294 | 0.002220049 | 0.036130384 |
| 387990 | NM_207377 TOMM20L | translocase of outer mitochondrial membrane 20 like | 138.302826 | -3.607095 | 0.002220049 | 0.036130384 |
| 100128124 | NM_001129895HGC6.3 | uncharacterized LOC100128124 | 138.302826 | -2.926213 | 0.002220049 | 0.036130384 |
| 100302691 | NR_033927 LINC00184 | long intergenic non-protein coding RNA 184 | 138.302826 | -3.495633 | 0.002220049 | 0.036130384 |
| 101926975 | NR_110558 LINC01844 | long intergenic non-protein coding RNA 1844 | 138.302826 | -3.727840 | 0.002220049 | 0.036130384 |
| 5027 | NM_002562,NRP2RX7 | purinergic receptor P2X 7 | 146.379462 | -2.200589 | 0.0017497 | 0.030261321 |
| 5175 | NM_000442 PECAM1 | platelet and endothelial cell adhesion molecule 1 | 146.379462 | -2.692660 | 0.0017497 | 0.030261321 |
| 5919 | NM_002889 RARRES2 | retinoic acid receptor responder 2 | 146.379462 | -3.343198 | 0.0017497 | 0.030261321 |
| 27151 | NM_015692,NRCPAMD8 | C3 and PZP like, alpha-2-macroglobulin domain containing | 146.379462 | -1.229508 | 0.0017497 | 0.030261321 |
| 54754 | NM_017561 NUTM2F | NUT family member 2F | 146.379462 | -3.666401 | 0.0017497 | 0.030261321 |
| 254956 | NM_001286828MORN5 | MORN repeat containing 5 | 146.379462 | -3.000702 | 0.0017497 | 0.030261321 |
| 83890 | NM_001349303SPATA9 | spermatogenesis associated 9 | 154.456099 | -3.392488 | 0.001391155 | 0.025331018 |
| 196996 | NM_001012642GRAMD2A | GRAM domain containing 2A | 154.456099 | -3.607471 | 0.001391155 | 0.025331018 |
| 339453 | NM_001114748TMEM240 | transmembrane protein 240 | 154.456099 | -3.607471 | 0.001391155 | 0.025331018 |
| 729993 | NM_001145204SHISA9 | shisa family member 9 | 154.456099 | -2.491579 | 0.001391155 | 0.025331018 |
| 100129148 | NR_033999 LOC100129148 | uncharacterized LOC100129148 | 154.456099 | -3.392488 | 0.001391155 | 0.025331018 |
| 105375606 | NR_147077 MNX1-AS2 | MNX1 antisense RNA 2 | 154.456099 | -3.295872 | 0.001391155 | 0.025331018 |
| 924 | NM_006137 CD7 | CD7 molecule | 162.532736 | -3.550851 | 0.001115132 | 0.021383887 |
| 22952 | NR_040249 CYP2G1P | cytochrome P450 family 2 subfamily G member 1, pseudog | 162.532736 | -3.443503 | 0.001115132 | 0.021383887 |
| 24141 | NM_001199897LAMP5 | lysosomal associated membrane protein family member 5 | 162.532736 | -3.550851 | 0.001115132 | 0.021383887 |
| 25834 | NM_001351282MGAT4C | MGAT4 family member C | 162.532736 | -3.205462 | 0.001115132 | 0.021383887 |
| 84467 | NM_001321431FBN3 | fibrillin 3 | 162.532736 | -3.205462 | 0.001115132 | 0.021383887 |
| 145624 | NR_022009 PWAR1 | Prader Willi/Angelman region RNA 1 | 162.532736 | -3.296047 | 0.001115132 | 0.021383887 |
| 201617 | NR_038221 LINC00870 | long intergenic non-protein coding RNA 870 | 162.532736 | -3.162206 | 0.001115132 | 0.021383887 |
| 374569 | NM_001080464ASPG | asparaginase | 162.532736 | -3.550851 | 0.001115132 | 0.021383887 |
| 386627 | NR_037897 SAP30L-AS1 | SAP30L antisense RNA 1 (head to head) | 162.532736 | -2.692960 | 0.001115132 | 0.021383887 |
| 404663 | NR_033383 LINC01194 | long intergenic non-protein coding RNA 1194 | 162.532736 | -2.574571 | 0.001115132 | 0.021383887 |
| 643596 | NM_001190228RNF224 | ring finger protein 224 | 162.532736 | -2.926694 | 0.001115132 | 0.021383887 |
| 100129316 | NR_033912 LOC100129316 | uncharacterized LOC100129316 | 162.532736 | -3.550851 | 0.001115132 | 0.021383887 |
| 100132163 | NR_029379 PHKA2-AS1 | PHKA2 antisense RNA 1 | 162.532736 | -3.343553 | 0.001115132 | 0.021383887 |
| 100507099 | NR_103839 FRY-AS1 | FRY antisense RNA 1 | 162.532736 | -3.550851 | 0.001115132 | 0.021383887 |
| 105376772 | NR_135170 LOC105376772 | uncharacterized LOC105376772 | 162.532736 | -3.550851 | 0.001115132 | 0.021383887 |
| 107985781 | NR_144453,NR FSIP2-AS1 | FSIP2 antisense RNA 1 | 162.532736 | -3.443503 | 0.001115132 | 0.021383887 |
| 11279 | NM_001159296KLF8 | Kruppel like factor 8 | 162.555184 | 1.527987 | 6.94142E-10 | 1.81751E-07 |
| 151258 | NM_001199148SLC38A11 | solute carrier family 38 member 11 | 164.471865 | 0.206260 | 4.8058E-09 | 9.94079E-07 |
| 284950 | NR_038888 LOC284950 | uncharacterized LOC284950 | 170.609373 | -3.120370 | 0.000900684 | 0.018158517 |
| 347736 | NM_001349018NME9 | NME/NM23 family member 9 | 170.609373 | -3.205634 | 0.000900684 | 0.018158517 |
| 653483 | NR_027906 AFDN-AS1 | AFDN antisense RNA 1 (head to head) | 170.609373 | -3.296223 | 0.000900684 | 0.018158517 |

| 101927905 | NR_120454,NR | LINC02449 | long intergenic non-protein coding RNA 2449 | 170.609373 | -3.496368 | 0.000900684 | 0.018158517 |
| --- | --- | --- | --- | --- | --- | --- | --- |
| 51673 | NM_015964,N | TPPP3 | tubulin polymerization promoting protein family member 3 | 172.071572 | 4.683782 | 2.16302E-11 | 1.20924E-08 |
| 1415 | NM_000496 | CRYBB2 | crystallin beta B2 | 178.686010 | -3.250395 | 0.000732646 | 0.01532333 |
| 11249 | NM_007226 | NXPH2 | neurexophilin 2 | 178.686010 | -3.079713 | 0.000732646 | 0.01532333 |
| 285668 | NM_173667,NR | C5orf64 | chromosome 5 open reading frame 64 | 178.686010 | -3.162546 | 0.000732646 | 0.01532333 |
| 100506674 | NR_109862,NR | BRCAT54 | breast cancer-associated transcript 54 | 178.686010 | -3.079713 | 0.000732646 | 0.01532333 |
| 100874074 | NR_046551 | DLEU7-AS1 | DLEU7 antisense RNA 1 | 178.686010 | -3.443866 | 0.000732646 | 0.01532333 |
| 3196 | NM_016170 | TLX2 | T-cell leukemia homeobox 2 | 186.762646 | -2.413759 | 0.00059992 | 0.013229577 |
| 8190 | NM_001202553MIA | | melanoma inhibitory activity | 186.762646 | -3.393206 | 0.00059992 | 0.013229577 |
| 10595 | NM_001308220ERN2 | | endoplasmic reticulum to nucleus signaling 2 | 186.762646 | -3.393206 | 0.00059992 | 0.013229577 |
| 29958 | NM_013391,NRDMGDH | | dimethylglycine dehydrogenase | 186.762646 | -2.963870 | 0.00059992 | 0.013229577 |
| 79656 | NM_001302082BEND5 | | BEN domain containing 5 | 186.762646 | -3.393206 | 0.00059992 | 0.013229577 |
| 644619 | NR_027392 INTS4P2 | | integrator complex subunit 4 pseudogene 2 | 186.762646 | -2.662889 | 0.00059992 | 0.013229577 |
| 343990 | NM_207362 KIAA1211L | | KIAA1211 like | 190.102567 | 1.096814 | 5.72635E-10 | 1.55855E-07 |
| 3026 | NM_001177660HABP2 | | hyaluronan binding protein 2 | 193.195846 | 2.808082 | 3.40969E-11 | 1.77898E-08 |
| 10993 | NM_006843 SDS | | serine dehydratase | 194.839283 | -3.250743 | 0.0004943 | 0.01131223 |
| 55200 | NM_001144856PLEKHG6 | | pleckstrin homology and RhoGEF domain containing G6 | 194.839283 | -2.465680 | 0.0004943 | 0.01131223 |
| 84708 | NM_001126328LNX1 | | ligand of numb-protein X 1 | 194.839283 | -2.693559 | 0.0004943 | 0.01131223 |
| 284632 | NR_027087 LOC284632 | | uncharacterized LOC284632 | 194.839283 | -3.344264 | 0.0004943 | 0.01131223 |
| 100131561 | NR_024013 FKSG29 | | FKSG29 | 194.839283 | -2.927334 | 0.0004943 | 0.01131223 |
| 105180390 | NR_146073,NR SPDYE13P | | speedy/RINGO cell cycle regulator family member E13, pse | 194.839283 | -3.344264 | 0.0004943 | 0.01131223 |
| 55808 | NM_001289107ST6GALNAC1 | | ST6 N-acetylgalactosaminide alpha-2,6-sialyltransferase 1 | 199.146942 | 3.834963 | 1.39155E-11 | 8.22405E-09 |
| 7103 | NM_004616 TSPAN8 | | tetraspanin 8 | 199.557839 | 4.500267 | 9.83461E-12 | 6.56222E-09 |
| 8633 | NM_003728 UNC5C | | unc-5 netrin receptor C | 202.915920 | -3.040501 | 0.00040966 | 0.009751222 |
| 9635 | NM_006536 CLCA2 | | chloride channel accessory 2 | 202.915920 | -3.206322 | 0.00040966 | 0.009751222 |
| 56169 | NM_031415 GSDMC | | gasdermin C | 202.915920 | -2.927494 | 0.00040966 | 0.009751222 |
| 126669 | NM_001010846SHE | | Src homology 2 domain containing E | 202.915920 | -2.465819 | 0.00040966 | 0.009751222 |
| 158326 | NM_001177704FREM1 | | FRAS1 related extracellular matrix 1 | 202.915920 | -3.296926 | 0.00040966 | 0.009751222 |
| 284593 | NR_027055 FAM41C | | family with sequence similarity 41 member C | 202.915920 | -2.822657 | 0.00040966 | 0.009751222 |
| 390213 | NR_033791 DOC2GP | | double C2 domain gamma pseudogene | 202.915920 | -3.040501 | 0.00040966 | 0.009751222 |
| 3957 | NM_006498 LGALS2 | | galectin 2 | 204.544823 | 1.486233 | 1.6953E-10 | 6.19801E-08 |
| 79825 | NM_024768 EFCC1 | | EF-hand and coiled-coil domain containing 1 | 210.992557 | -3.002009 | 0.000341383 | 0.008456898 |
| 114900 | NM_031909 C1QTNF4 | | C1q and TNF related 4 | 210.992557 | -3.251091 | 0.000341383 | 0.008456898 |
| 222545 | NM_001286354GPRC6A | | G protein-coupled receptor class C group 6 member A | 210.992557 | -3.251091 | 0.000341383 | 0.008456898 |
| 9058 | NM_001145975SLC13A2 | | solute carrier family 13 member 2 | 212.159495 | 1.825470 | 7.95932E-11 | 3.50295E-08 |
| 53842 | NM_001111319CLDN22 | | claudin 22 | 219.069194 | -3.080547 | 0.000285964 | 0.007393951 |
| 53919 | NM_001145944SLCO1C1 | | solute carrier organic anion transporter family member 1C1 | 219.069194 | -3.206666 | 0.000285964 | 0.007393951 |
| 146556 | NM_001098514C16orf89 | | chromosome 16 open reading frame 89 | 219.069194 | -3.206666 | 0.000285964 | 0.007393951 |
| 100996583 | NR_121638 LOC100996583 | | uncharacterized LOC100996583 | 219.069194 | -3.206666 | 0.000285964 | 0.007393951 |
| 1187 | NM_001042704CLCNKA | | chloride voltage-gated channel Ka | 227.145830 | -3.163567 | 0.000240716 | 0.006534407 |
| 7475 | NM_006522 WNT6 | | Wnt family member 6 | 227.145830 | -2.892175 | 0.000240716 | 0.006534407 |
| 25858 | NM_001039496CATSPERZ | | catsper channel auxiliary subunit zeta | 227.145830 | -3.163567 | 0.000240716 | 0.006534407 |
| 50856 | NM_016184,N CLEC4A | | C-type lectin domain family 4 member A | 227.145830 | -2.892175 | 0.000240716 | 0.006534407 |

| 56301 | NM_019849 | SLC7A10 | solute carrier family 7 member 10 | 227.145830 | -2.466237 | 0.000240716 | 0.006534407 |
| --- | --- | --- | --- | --- | --- | --- | --- |
| 101927891 | NR_110682 | PKN2-AS1 | PKN2 antisense RNA 1 | 227.145830 | -2.964679 | 0.000240716 | 0.006534407 |
| 117283 | NM_001142883IP6K3 | | inositol hexakisphosphate kinase 3 | 235.222467 | -2.604585 | 0.000203569 | 0.005721223 |
| 100507377 | NR_038300 LOC100507377 | | uncharacterized LOC100507377 | 235.222467 | -2.823278 | 0.000203569 | 0.005721223 |
| 101927459 | NR_125416,NR LINC01608 | | long intergenic non-protein coding RNA 1608 | 235.222467 | -3.121718 | 0.000203569 | 0.005721223 |
| 195977 | NM_001278688ANTXRL | | anthrax toxin receptor like | 243.299104 | -2.292782 | 0.000172911 | 0.005044328 |
| 283796 | NR_024074 GOLGA8IP | | golgin A8 family member I, pseudogene | 243.299104 | -2.790099 | 0.000172911 | 0.005044328 |
| 646074 | NR_024593 POM121L10P | | POM121 transmembrane nucleoporin like 10, pseudogene | 243.299104 | -3.081047 | 0.000172911 | 0.005044328 |
| 90993 | NM_052854 CREB3L1 | | cAMP responsive element binding protein 3 like 1 | 246.224512 | 5.027116 | 2.52217E-12 | 2.08684E-09 |
| 23017 | NM_012306 FAIM2 | | Fas apoptotic inhibitory molecule 2 | 250.273149 | 0.809231 | 3.73278E-10 | 1.13144E-07 |
| 2104 | NM_001134285ESRRG | | estrogen related receptor gamma | 251.375741 | -3.041490 | 0.000147483 | 0.004440584 |
| 23643 | NM_001195797LY96 | | lymphocyte antigen 96 | 251.375741 | -1.869885 | 0.000147483 | 0.004440584 |
| 158248 | NM_001317037TTC16 | | tetratricopeptide repeat domain 16 | 251.375741 | -2.965164 | 0.000147483 | 0.004440584 |
| 392307 | NM_001012446FAM221B | | family with sequence similarity 221 member B | 251.375741 | -3.041490 | 0.000147483 | 0.004440584 |
| 84152 | NM_001242464PPP1R1B | | protein phosphatase 1 regulatory inhibitor subunit 1B | 256.504528 | 0.846673 | 3.21651E-10 | 1.00808E-07 |
| 29760 | NM_001114094BLNK | | B-cell linker | 259.452378 | -3.002989 | 0.000126293 | 0.00394617 |
| 80341 | NM_025227 BPIFB2 | | BPI fold containing family B member 2 | 259.452378 | -3.002989 | 0.000126293 | 0.00394617 |
| 100287284 | NM_001146221MANSC4 | | MANSC domain containing 4 | 259.452378 | -1.785172 | 0.000126293 | 0.00394617 |
| 2823 | NM_001261447GPM6A | | glycoprotein M6A | 267.529014 | -1.571209 | 0.000108555 | 0.003497601 |
| 3814 | NM_002256 KISS1 | | KiSS-1 metastasis-suppressor | 267.529014 | -2.965487 | 0.000108555 | 0.003497601 |
| 100874365 | NR_047506 HOXC-AS3 | | HOXC cluster antisense RNA 3 | 267.529014 | -2.726096 | 0.000108555 | 0.003497601 |
| 5169 | NM_005021,NRENPP3 | | ectonucleotide pyrophosphatase/phosphodiesterase 3 | 275.605651 | -2.858180 | 9.36436E-05 | 0.003104194 |
| 266977 | NM_025048,N ADGRF1 | | adhesion G protein-coupled receptor F1 | 275.605651 | -2.493688 | 9.36436E-05 | 0.003104194 |
| 102724467 | NR_120309 LOC102724467 | | uncharacterized LOC102724467 | 275.605651 | -2.634615 | 9.36436E-05 | 0.003104194 |
| 164684 | NM_152613 WBP2NL | | WBP2 N-terminal like | 283.682288 | -2.664669 | 8.10571E-05 | 0.002744133 |
| 286207 | NM_001012502CFAP157 | | cilia and flagella associated protein 157 | 283.682288 | -2.790869 | 8.10571E-05 | 0.002744133 |
| 401562 | NM_207510 LCNL1 | | lipocalin like 1 | 283.682288 | -2.893285 | 8.10571E-05 | 0.002744133 |
| 388550 | NR_027754 CEACAM22P | | carcinoembryonic antigen related cell adhesion molecule 22 | 291.758925 | -2.576880 | 7.03917E-05 | 0.002430804 |
| 100506898 | NR_049723 MAGOH2P | | mago homolog 2, pseudogene | 291.758925 | -2.605605 | 7.03917E-05 | 0.002430804 |
| 3048 | NM_000184 HBG2 | | hemoglobin subunit gamma 2 | 299.835562 | -2.824522 | 6.13206E-05 | 0.002217511 |
| 8418 | NR_002174,NR CMAHP | | cytidine monophospho-N-acetylneuraminic acid hydroxylas | 299.835562 | -1.998358 | 6.13206E-05 | 0.002217511 |
| 9758 | NM_014728 FRMPD4 | | FERM and PDZ domain containing 4 | 299.835562 | -2.824522 | 6.13206E-05 | 0.002217511 |
| 24150 | NM_016212,N TP53TG3 | | TP53 target 3 | 299.835562 | -2.037041 | 6.13206E-05 | 0.002217511 |
| 644050 | NR_104176 LINC01529 | | long intergenic non-protein coding RNA 1529 | 299.835562 | -2.824522 | 6.13206E-05 | 0.002217511 |
| 729355 | NM_001099687TP53TG3B | | TP53 target 3B | 299.835562 | -2.270490 | 6.13206E-05 | 0.002217511 |
| 100288486 | NR_034090,NR DDX11L9 | | DEAD/H-box helicase 11 like 9 | 299.835562 | -2.548857 | 6.13206E-05 | 0.002217511 |
| 161829 | NM_001286441EXD1 | | exonuclease 3'-5' domain containing 1 | 307.912198 | -2.225282 | 5.35781E-05 | 0.001996869 |
| 388503 | NR_027300 C3P1 | | complement component 3 precursor pseudogene | 307.912198 | -2.791331 | 5.35781E-05 | 0.001996869 |
| 100533105 | NM_001204173C8orf44-SGK3 | | C8orf44-SGK3 readthrough | 307.912198 | -2.577169 | 5.35781E-05 | 0.001996869 |
| 7053 | NM_003245 TGM3 | | transglutaminase 3 | 314.983615 | 1.171654 | 9.15526E-11 | 3.94534E-08 |
| 3827 | NM_000893,N KNG1 | | kininogen 1 | 315.988835 | -2.758885 | 4.69471E-05 | 0.001818542 |
| 5023 | NM_002558 P2RX1 | | purinergic receptor P2X 1 | 315.988835 | -2.758885 | 4.69471E-05 | 0.001818542 |
| 101929215 | NR_109930 CTD-2201I18.1 | | uncharacterized LOC101929215 | 315.988835 | -2.758885 | 4.69471E-05 | 0.001818542 |

| 3834 | NM_005355,N KIF25 | kinesin family member 25 | 324.065472 | -2.577458 | 4.12497E-05 | 0.001637714 |
| --- | --- | --- | --- | --- | --- | --- |
| 7412 | NM_001078,N VCAM1 | vascular cell adhesion molecule 1 | 324.065472 | -1.924299 | 4.12497E-05 | 0.001637714 |
| 200810 | NM_001015050ALG1L | ALG1, chitobiosyldiphosphodolichol beta-mannosyltransfera | 324.065472 | -1.998711 | 4.12497E-05 | 0.001637714 |
| 340895 | NM_001142308MALRD1 | MAM and LDL receptor class A domain containing 1 | 324.065472 | -2.577458 | 4.12497E-05 | 0.001637714 |
| 7434 | NM_001304522VIPR2 | vasoactive intestinal peptide receptor 2 | 332.142109 | -2.696103 | 3.6339E-05 | 0.001482588 |
| 102723927 | NR_110592 LINC01238 | long intergenic non-protein coding RNA 1238 | 332.142109 | -2.271012 | 3.6339E-05 | 0.001482588 |
| 142680 | NM_001177316SLC34A3 | solute carrier family 34 member 3 | 340.218746 | -2.665707 | 3.20938E-05 | 0.001357587 |
| 100534589 | NR_037940 HOXA10-HOXA9 | HOXA10-HOXA9 readthrough | 340.218746 | -2.665707 | 3.20938E-05 | 0.001357587 |
| 115110 | NR_037844 LOC115110 | uncharacterized LOC115110 | 348.295382 | -2.366458 | 2.84133E-05 | 0.001211813 |
| 2568 | NM_001291985GABRP | gamma-aminobutyric acid type A receptor pi subunit | 356.372019 | -2.606770 | 2.52136E-05 | 0.001095679 |
| 388284 | NM_001012984C16orf86 | chromosome 16 open reading frame 86 | 356.372019 | -2.606770 | 2.52136E-05 | 0.001095679 |
| 1361 | NM_001278541CPB2 | carboxypeptidase B2 | 364.448656 | -2.366727 | 2.24245E-05 | 0.000995387 |
| 1551 | NM_000765 CYP3A7 | cytochrome P450 family 3 subfamily A member 7 | 364.448656 | -2.578179 | 2.24245E-05 | 0.000995387 |
| 2867 | NM_005306 FFAR2 | free fatty acid receptor 2 | 364.448656 | -2.037999 | 2.24245E-05 | 0.000995387 |
| 7941 | NM_001168357PLA2G7 | phospholipase A2 group VII | 364.448656 | -2.018521 | 2.24245E-05 | 0.000995387 |
| 221303 | NM_001085480FAM162B | family with sequence similarity 162 member B | 364.448656 | -2.416773 | 2.24245E-05 | 0.000995387 |
| 5224 | NM_000290 PGAM2 | phosphoglycerate mutase 2 | 372.525293 | -2.550144 | 1.9987E-05 | 0.000910643 |
| 285596 | NM_173663,NRFAM153A | family with sequence similarity 153 member A | 372.525293 | -2.550144 | 1.9987E-05 | 0.000910643 |
| 654466 | NR_003670 FGF7P3 | fibroblast growth factor 7 pseudogene 3 | 380.601930 | -2.366996 | 1.78517E-05 | 0.00082241 |
| 100131814 | NR_026805 LINC00271 | long intergenic non-protein coding RNA 271 | 396.755203 | -2.204533 | 1.4326E-05 | 0.000679665 |
| 102724362 | NR_146466,NR SPINT1-AS1 | SPINT1 antisense RNA 1 | 396.755203 | -2.318879 | 1.4326E-05 | 0.000679665 |
| 101928287 | NR_110393 LINC01285 | long intergenic non-protein coding RNA 1285 | 412.908477 | -1.770833 | 1.15831E-05 | 0.000574573 |
| 50619 | NM_022047 DEF6 | DEF6, guanine nucleotide exchange factor | 420.985114 | -1.722827 | 1.04431E-05 | 0.000530748 |
| 64922 | NM_022901 LRRC19 | leucine rich repeat containing 19 | 420.985114 | -2.392485 | 1.04431E-05 | 0.000530748 |
| 100132923 | NR_027425 FAM66D | family with sequence similarity 66 member D | 429.061750 | -2.058707 | 9.43113E-06 | 0.000487707 |
| 6359 | NM_032965 CCL15 | C-C motif chemokine ligand 15 | 437.138387 | -2.343539 | 8.53121E-06 | 0.000449028 |
| 9381 | NM_001287489OTOF | otoferlin | 437.138387 | -2.058827 | 8.53121E-06 | 0.000449028 |
| 83661 | NM_031457 MS4A8 | membrane spanning 4-domains A8 | 445.215024 | -2.319674 | 7.72941E-06 | 0.000416361 |
| 101927117 | NR_109889 PLCG1-AS1 | PLCG1 antisense RNA 1 | 445.215024 | -2.183464 | 7.72941E-06 | 0.000416361 |
| 27035 | NM_001271815NOX1 | NADPH oxidase 1 | 448.718578 | 1.644780 | 1.03402E-11 | 6.68396E-09 |
| 1018 | NM_001258 CDK3 | cyclin dependent kinase 3 | 461.368298 | -1.739219 | 5.8006E-06 | 0.00032963 |
| 101928812 | NR_120557,NR LOC101928812 | uncharacterized LOC101928812 | 469.444934 | -1.517855 | 5.28636E-06 | 0.000303746 |
| 407011 | NR_029664 MIR23B | microRNA 23b | 477.521571 | -2.205806 | 4.82427E-06 | 0.000281099 |
| 3384 | NM_000873,N ICAM2 | intercellular adhesion molecule 2 | 485.598208 | -1.290430 | 4.40838E-06 | 0.000262033 |
| 2524 | NM_000511,N FUT2 | fucosyltransferase 2 | 493.674845 | -2.079702 | 4.03352E-06 | 0.000242539 |
| 130106 | NM_001029881CIB4 | calcium and integrin binding family member 4 | 501.751482 | -1.788424 | 3.69514E-06 | 0.000225599 |
| 4246 | NM_002407 SCGB2A1 | secretoglobin family 2A member 1 | 509.828118 | -2.141755 | 3.38925E-06 | 0.00021053 |
| 148545 | NM_001143989NBPF4 | NBPF member 4 | 509.828118 | -1.926934 | 3.38925E-06 | 0.00021053 |
| 349136 | NM_001284260WDR86 | WD repeat domain 86 | 517.904755 | -1.788641 | 3.11235E-06 | 0.000196277 |
| 84630 | NM_032538 TTBK1 | tau tubulin kinase 1 | 525.981392 | -1.963933 | 2.86135E-06 | 0.000183811 |
| 653689 | NM_001080843GSTT2B | glutathione S-transferase theta 2B (gene/pseudogene) | 525.981392 | -1.518532 | 2.86135E-06 | 0.000183811 |
| 53405 | NM_001114086CLIC5 | chloride intracellular channel 5 | 534.058029 | -2.080309 | 2.63354E-06 | 0.000171304 |
| 6564 | NM_005073 SLC15A1 | solute carrier family 15 member 1 | 542.134666 | -0.547716 | 2.4265E-06 | 0.000158335 |

| 9068 | NM_004673 ANGPTL1 | angiopoietin like 1 | 542.134666 | -2.060395 | 2.4265E-06 | 0.000158335 |
| --- | --- | --- | --- | --- | --- | --- |
| 84218 | NM_032258,N TBC1D3F | TBC1 domain family member 3F | 554.855705 | 1.365461 | 3.44013E-11 | 1.77898E-08 |
| 10017 | NM_001306168BCL2L10 | BCL2 like 10 | 558.287939 | -1.983142 | 2.06647E-06 | 0.000137003 |
| 6523 | NM_000343,N SLC5A1 | solute carrier family 5 member 1 | 582.517850 | -1.709235 | 1.63627E-06 | 0.000115516 |
| 220082 | NM_001286341SPERT | spermatid associated | 582.517850 | -1.575071 | 1.63627E-06 | 0.000115516 |
| 284417 | NM_001085488TMEM150B | transmembrane protein 150B | 582.517850 | -1.964747 | 1.63627E-06 | 0.000115516 |
| 3208 | NM_002143 HPCA | hippocalcin | 590.594486 | -1.505669 | 1.51666E-06 | 0.000109693 |
| 3818 | NM_000892,N KLKB1 | kallikrein B1 | 590.594486 | -1.946355 | 1.51666E-06 | 0.000109693 |
| 6997 | NM_001174136TDGF1 | teratocarcinoma-derived growth factor 1 | 614.824397 | -1.892545 | 1.2143E-06 | 9.30287E-05 |
| 80736 | NM_001178044SLC44A4 | solute carrier family 44 member 4 | 622.238493 | 2.133315 | 1.3734E-12 | 1.3528E-09 |
| 127 | NM_000670,N ADH4 | alcohol dehydrogenase 4 (class II), pi polypeptide | 630.977670 | -1.857755 | 1.0515E-06 | 8.14614E-05 |
| 51297 | NM_001243193BPIFA1 | BPI fold containing family A member 1 | 630.977670 | -1.857755 | 1.0515E-06 | 8.14614E-05 |
| 59341 | NM_001177428TRPV4 | transient receptor potential cation channel subfamily V me | 630.977670 | -1.857755 | 1.0515E-06 | 8.14614E-05 |
| 554225 | NR_146078 STRCP1 | stereocilin pseudogene 1 | 630.977670 | -1.806765 | 1.0515E-06 | 8.14614E-05 |
| 101101775 | NR_073455,NR TMEM220-AS1 | TMEM220 antisense RNA 1 | 630.977670 | -1.575666 | 1.0515E-06 | 8.14614E-05 |
| 136306 | NM_001139456SVOPL | SVOP like | 655.207581 | -1.575963 | 8.52417E-07 | 6.83421E-05 |
| 729668 | NR_120609 GOLGA2P6 | golgin A2 pseudogene 6 | 663.284218 | -1.741876 | 7.96041E-07 | 6.43207E-05 |
| 284749 | NR_026958 LINC00494 | long intergenic non-protein coding RNA 494 | 671.360854 | -1.664253 | 7.43944E-07 | 6.10654E-05 |
| 100132159 | NR_120496,NR CALML3-AS1 | CALML3 antisense RNA 1 | 671.360854 | -1.427369 | 7.43944E-07 | 6.10654E-05 |
| 100528016 | NR_037646 TMX2-CTNND1 | TMX2-CTNND1 readthrough (NMD candidate) | 671.360854 | -0.808980 | 7.43944E-07 | 6.10654E-05 |
| 80070 | NM_025003 ADAMTS20 | ADAM metallopeptidase with thrombospondin type 1 motif | 695.590765 | -1.281031 | 6.09843E-07 | 5.14882E-05 |
| 84239 | NM_032279 ATP13A4 | ATPase 13A4 | 703.667402 | -1.649603 | 5.71537E-07 | 4.86512E-05 |
| 388813 | NM_001256579LOC388813 | uncharacterized protein ENSP00000383407-like | 703.667402 | -1.710809 | 5.71537E-07 | 4.86512E-05 |
| 9153 | NM_004212 SLC28A2 | solute carrier family 28 member 2 | 711.744038 | -1.695368 | 5.35993E-07 | 4.65841E-05 |
| 440335 | NM_001253790SMIM22 | small integral membrane protein 22 | 711.744038 | -1.605436 | 5.35993E-07 | 4.65841E-05 |
| 441204 | NR_015364 LOC441204 | uncharacterized LOC441204 | 711.744038 | -1.634798 | 5.35993E-07 | 4.65841E-05 |
| 63910 | NM_001302643SLC17A9 | solute carrier family 17 member 9 | 752.127222 | -1.480675 | 3.92535E-07 | 3.53026E-05 |
| 161497 | NM_153700 STRC | stereocilin | 768.280496 | -1.591673 | 3.48017E-07 | 3.18528E-05 |
| 101060321 | NM_001291462TBC1D3G | TBC1 domain family member 3G | 792.403051 | 2.636029 | 3.06085E-13 | 3.95711E-10 |
| 202134 | NM_001265615FAM153B | family with sequence similarity 153 member B | 800.587043 | -1.138389 | 2.75404E-07 | 2.63738E-05 |
| 110384692 | NM_001352000LOC110384692 | complement C4A (Rodgers blood group)-like | 800.587043 | -1.317465 | 2.75404E-07 | 2.63738E-05 |
| 64092 | NM_001256370SAMSN1 | SAM domain, SH3 domain and nuclear localization signals | 832.893590 | -1.481625 | 2.19791E-07 | 2.22862E-05 |
| 6512 | NM_001287595SLC1A7 | solute carrier family 1 member 7 | 849.046864 | -1.455376 | 1.96936E-07 | 2.02668E-05 |
| 10417 | NM_001128325SPON2 | spondin 2 | 865.200138 | -1.429596 | 1.76792E-07 | 1.86579E-05 |
| 157855 | NM_001031836KCNU1 | potassium calcium-activated channel subfamily U member | 865.200138 | -1.379014 | 1.76792E-07 | 1.86579E-05 |
| 202658 | NM_001199119TRIM39-RPP21 | TRIM39-RPP21 readthrough | 865.200138 | -0.897117 | 1.76792E-07 | 1.86579E-05 |
| 1003 | NM_001795 CDH5 | cadherin 5 | 881.353411 | -1.047379 | 1.59E-07 | 1.73101E-05 |
| 9071 | NM_001160100CLDN10 | claudin 10 | 881.353411 | -1.192843 | 1.59E-07 | 1.73101E-05 |
| 119467 | NM_152311 CLRN3 | clarin 3 | 881.353411 | -1.354549 | 1.59E-07 | 1.73101E-05 |
| 649264 | NR_037839 CES5AP1 | carboxylesterase 5A pseudogene 1 | 881.353411 | -1.404268 | 1.59E-07 | 1.73101E-05 |
| 80258 | NM_025184 EFHC2 | EF-hand domain containing 2 | 905.583322 | -0.264947 | 1.3606E-07 | 1.54638E-05 |
| 93432 | NM_001293626MGAM2 | maltase-glucoamylase 2 (putative) | 918.198801 | 4.762161 | 2.88859E-15 | 7.46881E-12 |
| 374899 | NM_001037232ZNF829 | zinc finger protein 829 | 945.966506 | -0.197712 | 1.05822E-07 | 1.258E-05 |

| 730338 | NR_134575 | LOC730338 | uncharacterized LOC730338 | 945.966506 | -0.828217 | 1.05822E-07 | 1.258E-05 |
| --- | --- | --- | --- | --- | --- | --- | --- |
| 126 | NM_000669,NRADH1C alcohol dehydrogenase 1C (class I), gamma polypeptide | | | 956.653356 | 3.674529 | 1.20731E-14 | 2.7748E-11 |
| 79370 | NM_030766,N BCL2L14 BCL2 like 14 | | | 978.273053 | -1.238254 | 8.71577E-08 | 1.08606E-05 |
| 400618 | NR_103737,NR SOX9-AS1 SOX9 antisense RNA 1 | | | 978.273053 | -1.108901 | 8.71577E-08 | 1.08606E-05 |
| 147409 | NM_001134453DSG4 desmoglein 4 | | | 1034.809510 | -0.683538 | 6.29289E-08 | 8.1867E-06 |
| 154215 | NM_001040214NKAIN2 sodium/potassium transporting ATPase interacting 2 | | | 1034.809510 | -0.854709 | 6.29289E-08 | 8.1867E-06 |
| 3158 | NM_001166107HMGCS2 3-hydroxy-3-methylglutaryl-CoA synthase 2 | | | 1042.886147 | -1.172794 | 6.01494E-08 | 7.92478E-06 |
| 285905 | NR_146905,NR INTS4P1 integrator complex subunit 4 pseudogene 1 | | | 1050.962784 | -0.283257 | 5.75113E-08 | 7.62577E-06 |
| 101928738 | NR_110851 LINC01978 long intergenic non-protein coding RNA 1978 | | | 1067.116058 | -0.907865 | 5.26273E-08 | 7.0688E-06 |
| 54363 | NM_017545 HAO1 hydroxyacid oxidase 1 | | | 1115.575878 | -1.079628 | 4.06222E-08 | 5.71612E-06 |
| 101928100 | NR_120430 LOC101928100 uncharacterized LOC101928100 | | | 1155.959062 | -0.707766 | 3.29982E-08 | 4.82254E-06 |
| 407019 | NR_029665 MIR27B microRNA 27b | | | 1164.035699 | -0.963635 | 3.16803E-08 | 4.71445E-06 |
| 445328 | NM_001003702ARHGEF35 Rho guanine nucleotide exchange factor 35 | | | 1164.035699 | -0.177218 | 3.16803E-08 | 4.71445E-06 |
| 26191 | NM_001193431PTPN22 protein tyrosine phosphatase, non-receptor type 22 | | | 1172.112336 | -0.890958 | 3.04232E-08 | 4.59346E-06 |
| 566 | NM_001700 AZU1 azurocidin 1 | | | 1236.725430 | -0.708419 | 2.22065E-08 | 3.50643E-06 |
| 5244 | NM_000443,N ABCB4 ATP binding cassette subfamily B member 4 | | | 1252.878704 | -0.874037 | 2.05755E-08 | 3.32502E-06 |
| 28959 | NM_001101311TMEM176B transmembrane protein 176B | | | 1252.878704 | -0.882829 | 2.05755E-08 | 3.32502E-06 |
| 407977 | NM_172089 TNFSF12-TNFSF1 TNFSF12-TNFSF13 readthrough | | | 1293.261888 | -0.419232 | 1.70706E-08 | 2.84985E-06 |
| 7113 | NM_001135099TMPRSS2 transmembrane protease, serine 2 | | | 1349.798346 | -0.655586 | 1.28034E-08 | 2.22554E-06 |
| 79820 | NM_024764 CATSPERB cation channel sperm associated auxiliary subunit beta | | | 1470.947898 | -0.649017 | 7.7188E-09 | 1.42557E-06 |
| 25850 | NM_001242472ZNF345 zinc finger protein 345 | | | 1543.637629 | 0.127230 | 5.80428E-09 | 1.15444E-06 |
| 100129046 | NR_034091 LOC100129046 uncharacterized LOC100129046 | | | 1575.944176 | -0.466767 | 5.13437E-09 | 1.04122E-06 |
| 55 | NM_001099,N ACPP acid phosphatase, prostate | | | 1648.633907 | -0.408929 | 3.92952E-09 | 8.21032E-07 |
| 400935 | NM_001001694IL17REL interleukin 17 receptor E like | | | 1664.787181 | -0.396386 | 3.70844E-09 | 7.82745E-07 |
| 140683 | NM_001319164BPIFA2 BPI fold containing family A member 2 | | | 1680.940454 | -0.507771 | 3.50165E-09 | 7.46717E-07 |
| 7097 | NM_001318787TLR2 toll like receptor 2 | | | 1721.323638 | 0.626038 | 3.0406E-09 | 6.76289E-07 |
| 167838 | NM_153235 TXLNB taxilin beta | | | 2020.159200 | 0.637292 | 1.16888E-09 | 2.74753E-07 |
| 10050 | NM_001286121SLC17A4 solute carrier family 17 member 4 | | | 2036.312474 | -0.237885 | 1.11439E-09 | 2.64956E-07 |
| 8470 | NM_001145670SORBS2 sorbin and SH3 domain containing 2 | | | 2076.695658 | -0.052910 | 9.90586E-10 | 2.41062E-07 |
| 100499483 | NM_001348010CCDC180 coiled-coil domain containing 180 | | | 2084.772294 | 0.780702 | 9.67783E-10 | 2.41062E-07 |
| 148170 | NM_145057 CDC42EP5 CDC42 effector protein 5 | | | 2270.534941 | -0.000334 | 5.67145E-10 | 1.55855E-07 |
| 2331 | NM_002023,NRFMOD fibromodulin | | | 2329.929629 | 5.343952 | 2.72673E-17 | 1.88008E-13 |
| 7032 | NM_005423 TFF2 trefoil factor 2 | | | 2415.914403 | 0.003654 | 3.90654E-10 | 1.13812E-07 |
| 81031 | NM_030777 SLC2A10 solute carrier family 2 member 10 | | | 2423.991040 | 0.008379 | 3.8289E-10 | 1.13144E-07 |
| 100127888 | NR_024470 SLCO4A1-AS1 SLCO4A1 antisense RNA 1 | | | 2423.991040 | 0.050601 | 3.8289E-10 | 1.13144E-07 |
| 363 | NM_001652 AQP6 aquaporin 6 | | | 2504.757408 | 0.082189 | 3.14344E-10 | 1.00034E-07 |
| 192134 | NM_138706 B3GNT6 UDP-GlcNAc:betaGal beta-1,3-N-acetylglucosaminyltransfer | | | 2771.286422 | 0.231163 | 1.70794E-10 | 6.19801E-08 |
| 22914 | NM_007360 KLRK1 killer cell lectin like receptor K1 | | | 2876.282701 | 0.407194 | 1.364E-10 | 5.40596E-08 |
| 4065 | NM_002349 LY75 lymphocyte antigen 75 | | | 2932.819158 | 0.406951 | 1.21241E-10 | 4.91738E-08 |
| 721 | NM_001002029C4B complement C4B (Chido blood group) | | | 2940.895795 | 0.460125 | 1.19239E-10 | 4.91738E-08 |
| 4113 | NM_002364 MAGEB2 MAGE family member B2 | | | 3263.961267 | 2.564390 | 6.24305E-11 | 2.80734E-08 |
| 54553 | NR_033797 LINC02486 long intergenic non-protein coding RNA 2486 | | | 3360.880909 | 1.075977 | 5.22835E-11 | 2.45792E-08 |
| 440107 | NM_001004330PLEKHG7 pleckstrin homology and RhoGEF domain containing G7 | | | 3385.110819 | 0.565752 | 5.00546E-11 | 2.40786E-08 |

| 3683 | NM_001114380ITGAL integrin subunit alpha L | 3562.796829 | 0.706837 | 3.66925E-11 | 1.85118E-08 |
| --- | --- | --- | --- | --- | --- |
| 340706 | NM_001272046VWA2 von Willebrand factor A domain containing 2 | 3918.168848 | 0.702622 | 2.03225E-11 | 1.1677E-08 |
| 342035 | NM_001330297GLDN gliomedin | 4233.157683 | 0.822931 | 1.26959E-11 | 7.72399E-09 |
| 4101 | NM_001282501MAGEA2 MAGE family member A2 | 4297.770777 | 1.920687 | 1.15769E-11 | 7.25658E-09 |
| 266740 | NM_001321400MAGEA2B MAGE family member A2B | 4580.453065 | 1.830028 | 7.85063E-12 | 5.41301E-09 |
| 2348 | NM_000802,N FOLR1 folate receptor 1 | 4798.522259 | 1.092259 | 5.84951E-12 | 4.17231E-09 |
| 91894 | NM_080659 C11orf52 chromosome 11 open reading frame 52 | 5121.587731 | 1.317818 | 3.93018E-12 | 2.90342E-09 |
| 54328 | NM_018969 GPR173 G protein-coupled receptor 173 | 5258.890557 | 1.282439 | 3.3436E-12 | 2.66009E-09 |
| 100289574 | NR_109773 HERC2P4 hect domain and RLD 2 pseudogene 4 | 5581.956029 | 1.196354 | 2.30158E-12 | 1.98367E-09 |
| 5284 | NM_002644 PIGR polymeric immunoglobulin receptor | 5590.032665 | 1.586248 | 2.28133E-12 | 1.98367E-09 |
| 121838 | NR_026955 LINC00284 long intergenic non-protein coding RNA 284 | 5735.412128 | 1.283222 | 1.94998E-12 | 1.83342E-09 |
| 720 | NM_001252204C4A complement C4A (Rodgers blood group) | 6462.309440 | 1.687785 | 9.32057E-13 | 9.6398E-10 |
| 54848 | NM_001242729ARHGEF38 Rho guanine nucleotide exchange factor 38 | 7560.732045 | 1.643332 | 3.53665E-13 | 4.30327E-10 |
| 155465 | NM_176813 AGR3 anterior gradient 3, protein disulphide isomerase family me | 7851.490969 | 1.685118 | 2.80566E-13 | 3.95711E-10 |
| 4129 | NM_000898 MAOB monoamine oxidase B | 8618.771465 | 2.442553 | 1.57322E-13 | 2.50323E-10 |
| 200765 | NM_145702 TIGD1 tigger transposable element derived 1 | 9159.906131 | 2.723315 | 1.07627E-13 | 1.85521E-10 |
| 414059 | NM_001001417TBC1D3B TBC1 domain family member 3B | 11397.134524 | 2.532611 | 2.78041E-14 | 5.22843E-11 |
| 92747 | NM_033197 BPIFB1 BPI fold containing family B member 1 | 17850.367327 | 2.868585 | 1.70416E-15 | 5.8751E-12 |
| 7033 | NM_003226 TFF3 trefoil factor 3 | 24238.987036 | 3.349642 | 2.53194E-16 | 1.18401E-12 |
| 100533483 | NR_037923 DNAAF4-CCPG1 DNAAF4-CCPG1 readthrough (NMD candidate) | 36806.233897 | 4.295477 | 1.87474E-17 | 1.88008E-13 |
| 100532737 | NR_037853 ATP6V1G2-DDX3 ATP6V1G2-DDX39B readthrough (NMD candidate) | 71834.607697 | 4.871225 | 2.88438E-19 | 5.96634E-15 |
