# Supplementary material for: Noggin contributes to brain metastatic colonization of lung cancer cells
Source: Cancer Cell Int. 2023 Nov 28;23:299. doi: 10.1186/s12935-023-03155-7 (PMC10683317; doi:10.1186/s12935-023-03155-7)
Supplement: Supplementary file 2 — Additional file 2: Table S2. qRT-PCR primer sequences used in this study. [file 12935_2023_3155_MOESM2_ESM.docx]

**Supplementary Table 2.** qRT-PCR primer sequences used in this study.

| **genes** | **forward (5' → 3')** | **reverse (5' → 3')** |
| --- | --- | --- |
| *RPL32* | ACAAAGCACATGCTGCCCAGTG | TTCCACGATGGCTTTGCGGTTC |
| *CRB3* | CTTCTGCAAATGAGAATAGCACTG | GACCACGATGATAGCAGTGATGG |
| *CDH1* | GCCTCCTGAAAAGAGAGTGGAAG | TGGCAGTGTCTCTCCAAATCCG |
| *PARD3* | CGGTCAAAAGAGAACCACGCAG | CATTCACCCGAAGCCTTCCATC |
| *PATJ* | ACAAGGCAGATTTGACGACCTGG | CTTTGAGCCACAACAGGAAGGTC |
| *SNAI1* | CTGAGGCCAAGGATCTCCAG | TGCAGTATTTGCAGTTGAAGGC |
| *TWIST2* | GCAAGATCCAGACGCTCAAGCT | ACACGGAGAAGGCGTAGCTGAG |
| *ZEB1* | GGCATACACCTACTCAACTACGG | TGGGCGGTGTAGAATCAGAGTC |
| *TWIST1* | GCCAGGTACATCGACTTCCTCT | TCCATCCTCCAGACCGAGAAGG |
| *FN1* | ACAACACCGAGGTGACTGAGAC | GGACACAACGATGCTTCCTGAG |
| *VIM* | AGGCAAAGCAGGAGTCCACTGA | ATCTGGCGTTCCAGGGACTCAT |
| *CDH2* | ATCTCGGGTCAGCTGTCGG | GGCTATCTGCTCGCGATCC |
| *SNAI2* | ATCTGCGGCAAGGCGTTTTCCA | GAGCCCTCAGATTTGACCTGTC |
| *ZEB2* | AATGCACAGAGTGTGGCAAGGC | CTGCTGATGTGCGAACTGTAGG |
| *NOG* | GCCAGCACTATCTCCACATCCG | AGCAGCGTCTCGTTCAGATCCT |
