# Supplementary material for: Noggin contributes to brain metastatic colonization of lung cancer cells
Source: Cancer Cell Int. 2023 Nov 28;23:299. doi: 10.1186/s12935-023-03155-7 (PMC10683317; doi:10.1186/s12935-023-03155-7)
Supplement: Supplementary file 3 — Additional file 3. Method and figures. [file 12935_2023_3155_MOESM3_ESM.pdf]

Supplementary Information

Noggin contributes to brain metastatic colonization of lung cancer cells

Jung Eun Lee, Jihye Park, Eun Ju Kim, Yoon Ho Ko, Soon Auck Hong, Seung Ho Yang, Young-Ho Ahn

Supplementary Method

Immunohistochemistry

The study received approval from the Institutional Review Board at St. Vincent's Hospital, The Catholic University of Korea (No. VC21SISI0159). Immunohistochemistry was conducted on brain metastasis and primary lung cancer tissues obtained from the same patients following craniotomy and bronchoscopy procedures, respectively. Lung cancer tissue sections were sliced to a thickness of 4 μm. These sections were deparaffinized, immersed in methanol with 3% hydrogen peroxide, blocked, and then subjected to immunostaining using a Noggin antibody (1:200, #ab16054; Abcam, Cambridge, MA, USA) through the avidin–biotin–peroxidase complex method with diaminobenzidine as the label, following the manufacturer’s instructions. Slides were counterstained with hematoxylin. The immunohistochemical staining was analyzed by a board-certified pathologist (SAH). Noggin immunohistochemical results were semi-quantitatively scored on a four-point scale: 0 for no immunoreaction, 1+ for faint immunoreaction in less than 20% of cells, 2+ for strong immunoreaction in 20–50% of cells, and 3+ for strong immunoreaction in more than 50% of cells. This immunohistochemistry was performed on two matched tumors, with Noggin expression observed as 2+ in brain metastasis tissues and 3+ in primary lung cancer tissues, respectively.

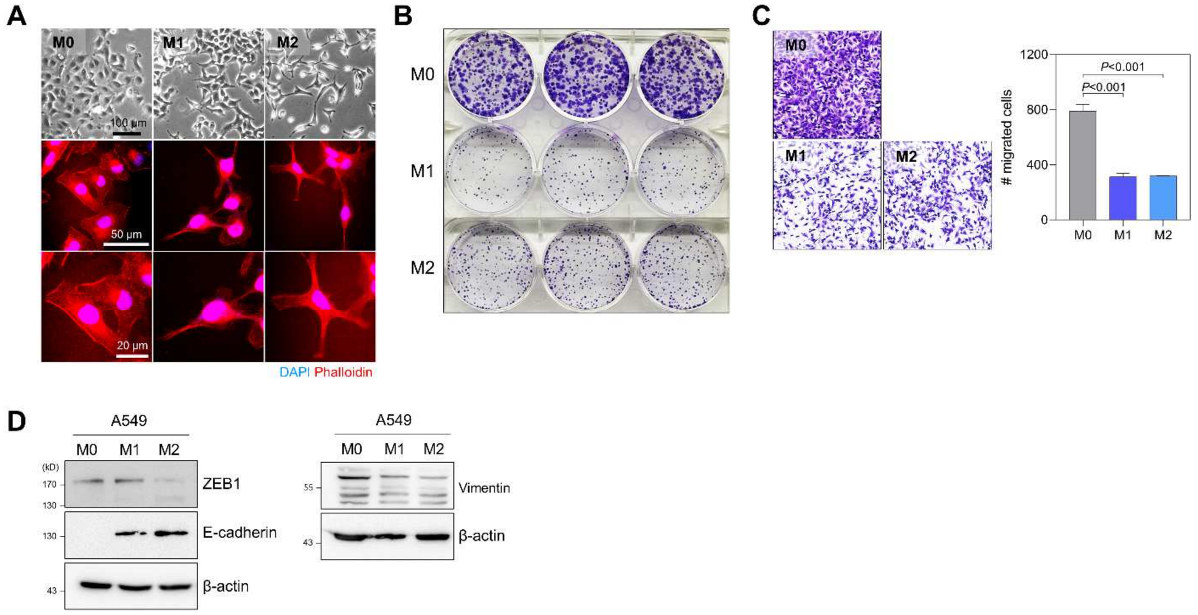

Supplementary Figure 1. Brain colonization diminishes the growth and migration of metastatic lung cancer cells.

A. Cell morphology of A549-M0, M1, and M2 cells. Representative phase-contrast and fluorescence microscopic images of A549-M0, M1, and M2 cells. Cells were stained with phalloidin conjugated to Alexa 594 (red) and DAPI (blue). To facilitate a valid comparison among three cells, we have presented identical images of M0 and M2 cells as shown in Figure 1B.

B. Colonogenic assays of M0, M1, and M2 cells. Colonies were stained with crystal violet at 6 days after seeding.

C. Transwell migration assays of M0, M1, and M2 cells. Cells ($1{\times}10^5$/insert) were cultured in upper wells for 24 h. Migrated cells were counted after staining with crystal violet. Mean ± SD (n = 3). *P*, unpaired two-tailed Student’s t-test.

D. Western blots of ZEB1, E-cadherin, and Vimentin in M0, M1, and M2 cells. β-actin was used as a loading control.

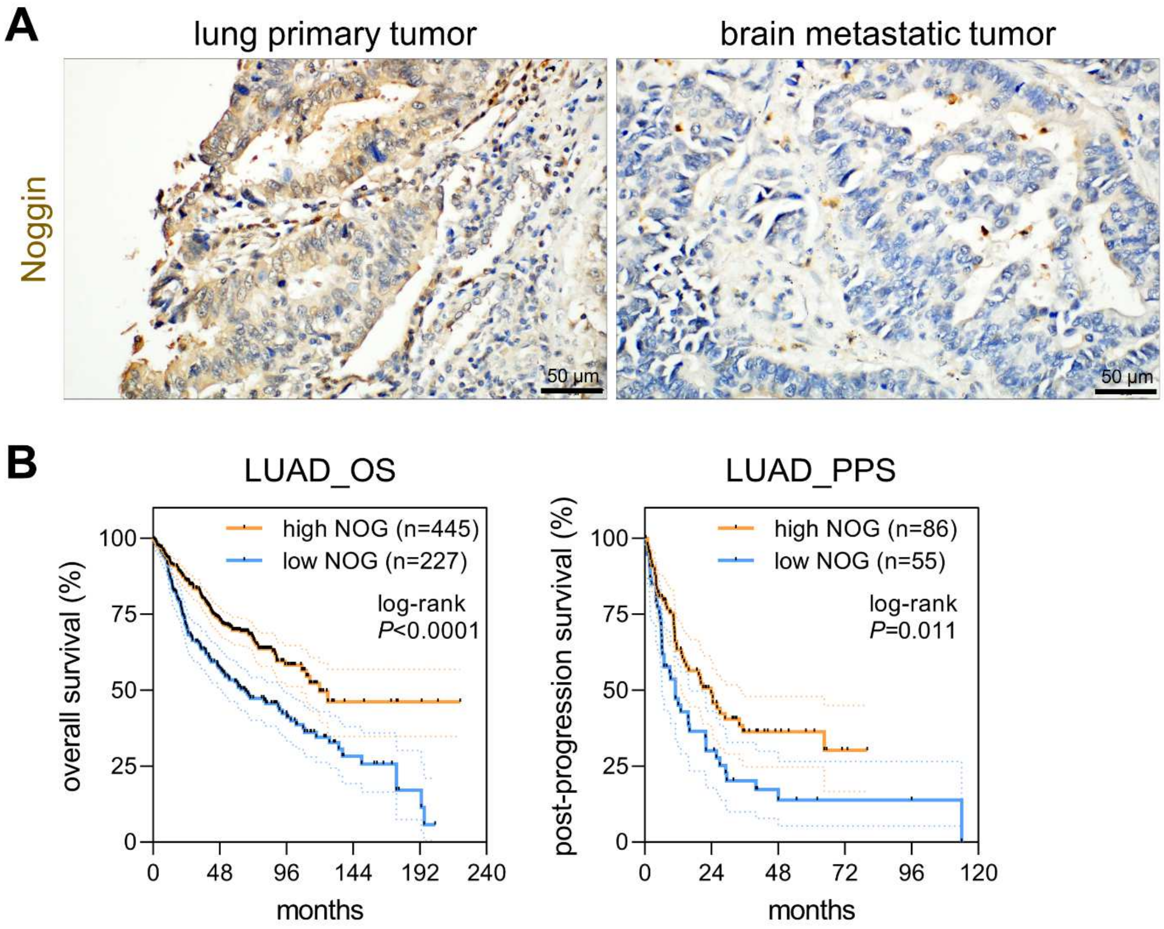

Supplementary Figure 2. Reduced Noggin expression is associated with brain metastasis and worse patient survival.

A. Noggin immunohistochemical staining of primary lung cancer specimen (*left*) and brain metastasis specimen (*right*) (×400).

B. Kaplan–Meier plots showing the overall survival (*left*) and post-progression survival rates (*right*) of patients with LUAD. Patients were divided into two groups (high and low) based on their Noggin expression levels. Data were obtained from the KM plotter (http://kmplot.com). *P*, log-rank test.
